# Viviparous Reptile Regarded to Have Temperature-Dependent Sex Determination Has Old XY Chromosomes

**DOI:** 10.1093/gbe/evaa104

**Published:** 2020-05-20

**Authors:** Paola Cornejo-Páramo, Duminda S B Dissanayake, Andrés Lira-Noriega, Mónica L Martínez-Pacheco, Armando Acosta, Ciro Ramírez-Suástegui, Fausto R Méndez-de-la-Cruz, Tamás Székely, Araxi O Urrutia, Arthur Georges, Diego Cortez

**Affiliations:** e1 Center for Genome Sciences, UNAM, Cuernavaca, México; e2 Department of Biology and Biochemistry, Milner Centre for Evolution, University of Bath, United Kingdom; e3 Institute for Applied Ecology, University of Canberra, Australia; e4 CSIRO, Australian National Wildlife Collection, Canberra, Australia; e5 CONACYT Research Fellow, Red de Estudios Moleculares Avanzados, Instituto de Ecología, A.C. Carretera antigua a Coatepec 351, Xalapa, Veracruz, México; e6 Biology Institute, UNAM, Mexico City, México; e7 Department of Evolutionary Zoology and Human Biology, University of Debrecen, Hungary; e8 Institute of Ecology, UNAM, Mexico City, Mexico

**Keywords:** temperature-dependent sex determination, viviparous reptiles, genetic sex determination systems, water skinks, *Eulamprus heatwolei*

## Abstract

The water skinks *Eulamprus tympanum* and *Eulamprus heatwolei* show thermally induced sex determination where elevated temperatures give rise to male offspring. Paradoxically, *Eulamprus* species reproduce in temperatures of 12–15 °C making them outliers when compared with reptiles that use temperature as a cue for sex determination. Moreover, these two species are among the very few viviparous reptiles reported to have thermally induced sex determination. Thus, we tested whether these skinks possess undetected sex chromosomes with thermal override. We produced transcriptome and genome data for *E. heatwolei*. We found that *E. heatwolei* presents XY chromosomes that include 14 gametologs with regulatory functions. The Y chromosomal region is 79–116 Myr old and shared between water and spotted skinks. Our work provides clear evidence that climate could be useful to predict the type of sex determination systems in reptiles and it also indicates that viviparity is strictly associated with sex chromosomes.

## Introduction

### *Eulamprus tympanum* and *Eulamprus heatwolei* Reproduce in Colder Conditions Compared with Other Species with Temperature-Dependent Sex Determination

Vertebrates exhibit two major classes of sex determination systems. Genotypic sex determination (GSD), where genetic components guide the development of the gonads, and temperature-dependent sex determination (TSD), where specific incubation temperatures define the sex of the embryos (Bachtrog et al. 2014). TSD in reptiles is thought to have evolved when external conditions that enhance either male or female offspring fitness could influence the sex of the embryos (Charnov and Bull 1977; Shine 1999). For this reason, the discovery of TSD in a viviparous skink was particularly notable (Robert and Thompson 2001). In viviparous species, the external conditions have little effect because embryonic development and hatchling occur inside the mother’s womb in a relatively stable environment.

The viviparous water skinks *Eulamprus tympanum* and *E. heatwolei* (family *Scincidae*) are classified as TSD species (Tree of Sex 2014) because cytogenetic analyses found no evidence of heteromorphic sex chromosomes and female *Eulamprus* skinks give rise to male offspring when they are kept at warm temperatures (32 °C) during pregnancy (Robert and Thompson 2001). Three features, however, make this classification of *Eulamprus* as TSD suspect: 1) These two species inhabit alpine habitats in southeastern Australia (Cogger 2000), whereas most reptiles with TSD systems inhabit lowland areas; 2) Uniquely, although all known viviparous reptiles have genetic sex determination systems, *E. tympanum* and *E. heatwolei* are the only known viviparous reptiles classified as TSD; and 3) Several studies have found 1:1 sex ratios in *E. heatwolei* at mild temperatures, both in the laboratory and in the field (Schwarzkopf and Shine 1991; Robert and Thompson 2001; Allsop et al. 2006). Taken together, these features implied either a GSD system with thermal override or, although less likely, an atypical TSD system.

**Fig. 1.**
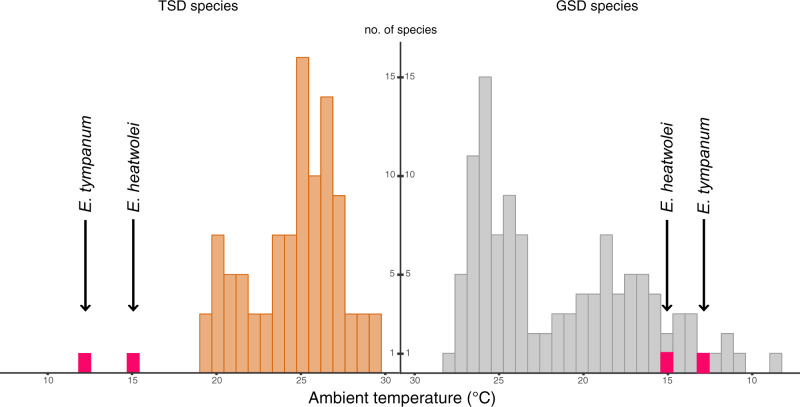
Distribution of average ambient temperature in geographical ranges during breeding seasons for reptile species with TSD (*n* = 101) and species with GSD (*n* = 99). Labeled bars in red correspond to average ambient temperature for *Eulamprus heatwolei* and *Eulamprus tympanum*.

We first examined whether ambient temperatures in areas inhabited by *E. tympanum* and *E. heatwolei* during breeding seasons were unusual compared with reptile species with TSD or GSD. For this, we mapped 30 years of ambient temperatures onto the geographic ranges of 101 species with TSD and 99 species with GSD during their breeding season (fig. 1). Average ambient temperatures for *E. heatwolei* and *E. tympanum* during their breeding seasons are 15 and 12.4 °C, respectively (fig. 1). Thus, *E. heatwolei* and *E. tympanum* are clear outliers when considered as TSD species, located at 3 and 4 SDs away from the mean of the distribution, respectively (fig. 1). In contrast, *Eulamprus* species are found within the distribution of species with GSD (fig. 1). These results are suggestive of the presence of previously undetected sex chromosomes in these two species.

### *Eulamprus heatwolei* Has XY Chromosomes

**Fig. 2.**
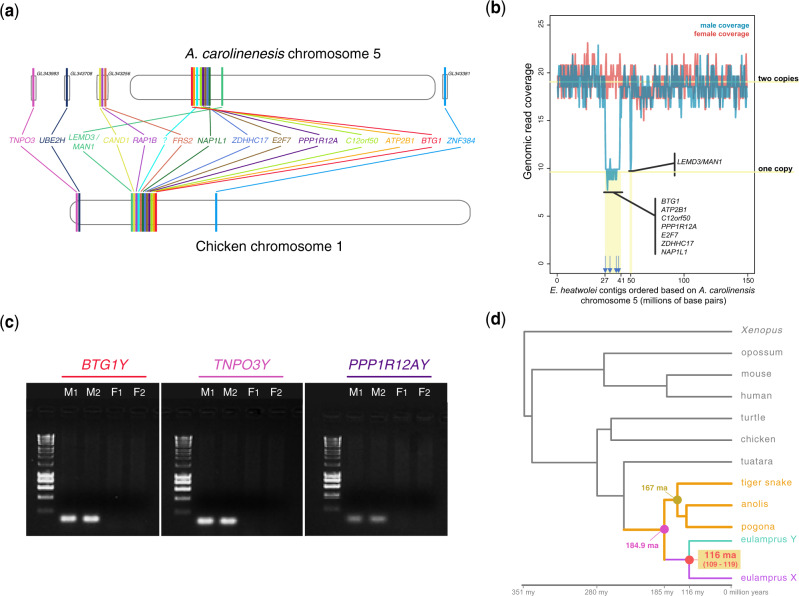
(*a*) Synteny of the 14 XY gametologs in other species. (*b*) Male (blue) and female (red) genomic coverage along the chromosome 5 of *Eulamprus heatwolei*. A syntenic region shows half of the coverage in males (one copy) but regular coverage in females (two copies). XY gametologs map to this region. Blue arrows show the matching locations of Y-linked markers from *Niveoscincus ocellatus*. (*c*) PCR screenings of two males and two females using primers designed to amplify three Y-linked genes (seven males and seven females were screened in total; see supplementary fig. 3, Supplementary Material online). (*d*) Time-calibrated synonymous substitution tree used to estimate the age of the XY chromosomes in *E. heatwolei*. Branch lengths represent millions of years.

To test for the presence of previously unidentified sex chromosomes in skinks, RNAseq data were generated from brain, liver, and gonads of one adult male and one adult female *E. heatwolei*. We then applied a subtraction approach (Cortez et al. 2014; Marin et al. 2017) to the male and female transcriptomic data of *E. heatwolei*. Specifically, we assembled a male-restricted transcriptome and used male and female genomic reads to uncover Y-linked transcripts (see Materials and Methods). We identified Y-linked transcripts from 14 protein-coding genes with known orthologous genes located on a single syntenic block on chromosome 5 in *Anolis carolinensis* and chromosome 1 in chicken (fig. 2*a* andsupplementary table 3, Supplementary Material online). Additionally, we performed a male and female genomic read coverage analysis of six chromosomes of *E. heatwolei* (see Materials and Methods). We found a region on chromosome 5 where the male shows only half of the coverage (i.e., one genomic copy, fig. 2*b*, andsupplementary fig. 1, Supplementary Material online). XY gametologs map to this specific region on chromosome 5 (fig. 2*a* and *b*) and analysis of their genomic coverage is consistent with two X gametologs in females but one X and one Y gametolog in males (supplementary fig. 2, Supplementary Material online). Lastly, we screened the genomes of seven males and seven females using standard PCRs and found that we could only amplify Y-linked sequences in males (fig. 2*c* andsupplementary fig. 3, Supplementary Material online). In summary, the results reveal the presence of sex chromosomes in *E. heatwolei.*

Functions associated to the identified Y-linked genes (retrieved from the GeneCards database; www.genecards.org, last accessed May 25, 2020) include ubiquitination (*UBE2H* and *CAND1*), signaling pathways (*LEMD3*/*MAN1* and *FRS2*), cell cycle, cell growth and differentiation (*PPP1R12A*, *E2F7*, *RAP1B*, and *BTG1*), transcription regulation (*ZNF384*), ion transport (*ATP2B1*), fatty acid metabolism (*ZDHHC17*), and DNA replication (*NAP1L1*). Many of the identified Y chromosome-linked genes have known regulatory functions. Examining the list of putative Y-linked genes, *PPP1R12A* is of particular interest. The protein coded by this gene is part of the PPP1C protein complex that catalyzes many protein dephosphorylation reactions in the cell and is essential for male fertility in mice (Silva et al. 2015). Another member of the PPP1C complex, the *PPP1CC* gene, is one of the oldest genes on the Y chromosome of pleurodonts (Marin et al. 2017), a group that diverged from the skink lineage 184.9 Ma (data retrieved from the TimeTree database; www.timetree.org/, last accessed May 25, 2020). The convergent co-option of genes forming part of the same molecular pathways (*PPP1R12A* and *PPP1CC* are probably involved in spermatogenesis) on the Y and W chromosomes is a frequent phenomenon in vertebrates (Marshall Graves and Peichel 2010; O’Meally et al. 2012).

To obtain an estimate for the origin of the male-specific region on the Y chromosome (MSY) in the *E. heatwolei* lineage, we used *d*_S_ trees based on the nucleotide sequences of the XY gametologs in *E. heatwolei* and orthologous sequences from other species (see Materials and Methods). From the synonymous substitution rates of the concatenated sequences of the XY gametologs, we estimated that *E. heatwolei* sex chromosomes originated ∼116 Ma (95% confident intervals: 109.45–119.28 Ma; values derived from 100 bootstrap rounds; fig. 2*d*). Moreover, estimates obtained using BEAST resulted in a sex chromosome age of ∼93 Ma (supplementary fig. 4, Supplementary Material online). Next, we retrieved Y-linked markers reported for the spotted skink, *Niveoscincus ocellatus* (Hill et al. 2018). These sequences are short (17–70 bp) and likely represent repeated, intergenic or intronic regions of the MSY. Only nine Y markers aligned to the *E. heatwolei* and *A. carolinensis* genomes; four mapped to multiple genomic locations (i.e., likely repeated sequences), one mapped to chromosome 3, and four mapped to chromosome 5, exactly within the MSY of *E. hetawolei* (fig. 2, blue arrows; supplementary table 3, Supplementary Material online). This association is highly significant (Fisher exact test, *P *<* *0.001) and indicative that water and spotted skinks likely share a common MSY, which originated >79 Ma (divergence time between the two groups of skinks; data retrieved from TimeTree; http://www.timetree.org/, last accessed May 25, 2020).

### Conclusions

Our work identified the MSY locus in *E. heatwolei*’s chromosome 5 and, importantly, it provided evidence that climate could be a good predictor of sex determination systems in reptiles. We can now reclassify *E. heatwolei* (and probably *E. tympanum*) as a viviparous skink showing GSD with thermally induced sex reversal at elevated temperatures (Shine et al. 2002; Quinn et al. 2007; Radder et al. 2008; Holleley et al. 2015). In the past, also the viviparous skink, *N. ocellatus* was assumed to have TSD on a lowland population (Pen et al. 2010). Here, we found that *E. heatwolei* and *N. ocellatus* share Y-linked sequences. We estimated that the sex-linked locus originated ∼79–116 Ma. Note that other species in the *Scincidae* family also have XY chromosomes (supplementary fig. 5, Supplementary Material online), so perhaps all skink species share the same GSD system.

Formerly, reptiles were thought to either have GSD or TSD systems. However, various studies have shown that in several species, including the viviparous *E. heatwolei* (Robert and Thompson 2001) (and this work), the viviparous *N. ocellatus* (Hill et al. 2018), the oviparous *Pogona vitticeps* (Quinn et al. 2007; Holleley et al. 2015), and the oviparous *Bassiana duperreyi* (Shine et al. 2002; Radder et al. 2008), certain incubation temperatures can override the signaling cascade initiated by sex-linked genes and influence the fate of the embryonic gonads. These thermally induced sex reversal mechanisms may represent retained elements of ancestral TSD systems. Further analyses in *E. heatwolei* and related species could help answer this question.

We know that viviparity has evolved from oviparity >100 times (Sites et al. 2011; Pyron and Burbrink 2014) and it is strongly correlated with the colonization of cold alpine environments (Lambert and Wiens 2013). The *Eulamprus* species were the last viviparous reptiles classified as TSD (Tree of Sex 2014). Our results indicate, for the moment, that viviparity in reptiles is strictly associated with GSD systems.

## Materials and Methods

### Data Generation

One adult male (Euhea_18_05) and one adult female individual (Euhea_18_03) of *E. heatwolei* species were captured from a population that inhabits Woods Reserve, Corin Road, ACT, Australia (−35.480751, 148.940398). Both individuals were sacrificed by intraperitoneal injection of pentobarbitone following the standard operating procedures specified by the animal ethics committee of the University of Canberra. We generated DNA-seq libraries for a male and female *E. heatwolei* from liver tissue using the Illumina TruSeq DNA protocol for short insert size (400–450 nt). We generated strand-specific RNA-seq libraries (using the Illumina TruSeq Stranded mRNA Library protocol) for a total of six samples obtained from brain, liver, and gonads for a male and female *E. heatwolei*. All libraries were sequenced on Illumina HiSeq 2500 sequencers at the University of Canberra. We generated 262–269 million 150-nt paired-end DNAseq reads. We generated 82–95 million 125-nt paired-end RNAseq reads. Further details in supplementary table 4, Supplementary Material online. Quality of the reads was verified using FastQC (http://www.bioinformatics.babraham.ac.uk/projects/fastqc, last accessed May 25, 2020) and the remaining adaptors were removed with Trimmomatic (Bolger et al. 2014).

### Assembly of Y-Linked Transcripts

To assemble Y-linked transcripts in *E. heatwolei*, we used a subtraction approach based on male and female RNAseq data (Cortez et al. 2014; Marin et al. 2017; Acosta et al. 2019). Briefly, male RNA-seq reads were aligned onto the de novo reconstructed female transcriptome from *E. heatwolei* using Hisat2 (v2.0.2) (Kim et al. 2015); no mismatches allowed; reads not mapping were selected. We also removed male RNA-seq reads sharing k-mers with the female transcriptome (Akagi et al. 2014). The selected reads were passed to Trinity (v2.0.2, default k-mer of 25 bp) (Grabherr et al. 2011) to assemble transcripts that were only present in male tissues. We obtained 21,249 transcripts that were subsequently aligned to the male and female genomic reads using BlastN (Altschul et al. 1990); at a 100–99% identity threshold. We selected those transcripts showing 4×–14× of averaged coverage of male genomic reads and zero averaged coverage of female genomic reads (supplementary table 3, Supplementary Material online). To establish Y gene identity, we searched NCBI GenBank (Reptile taxa only; http://www.ncbi.nlm.nih.gov/genbank, last accessed May 25, 2020) with BlastN and BlastX for the closest homologs and identified transcripts that coded for 14 proteins (supplementary table 3, Supplementary Material online). BlastX searches also allowed the identification of CDS regions. For these 14 Y-linked protein-coding genes, we performed BlastN searches against the de novo reconstructed female transcriptome from *E. heatwolei* to find the X gametologs (best match over the entire sequence; 95–97% identity). We verified the X gametologs identity using coverage analyses of male and female genomic reads and GenBank searches (same gene identity as Y gametologs). XY gametologs in *E. heatwolei* were searched against the *A. carolinensis* and chicken genomes using the sequence search engine at the ENSEMBL webpage (https://www.ensembl.org/Multi/Tools/Blast, last accessed May 25, 2020) to establish whether they formed a syntenic block. We validated the presence of a Y chromosome by PCR screenings using genomic DNA obtained from tails snips of seven males and seven females. Additional information can be found in the extended Materials and Methods section in the Supplementary Material online. We retrieved the Y-linked markers in *N. ocellatus* (Hill et al. 2018) and used BlastN (e-value < 0.01) to map these sequences onto the reconstructed *E. heatwolei* chromosomes and the *A. carolinenesis* reference genome downloaded from the Ensembl database (https://www.ensembl.org/, last accessed May 25, 2020; v.97). More details in supplementary table 3, Supplementary Material online.

### Genomic Coverage Analyses

We followed a methodology previously published (Vicoso et al. 2013). Briefly, the male and female genomic reads were assembled into contigs. The contigs were subsequently aligned and ordered based on the *A. carolinensis* reference genome. We used bowtie2 (Langmead and Salzberg 2012) to align the DNA-seq reads from the male and female *E. heatwolei* onto the reconstructed chromosomes. Coverage along the chromosomes was calculated using BEDtools (Quinlan and Hall 2010), bins of 100,000 nucleotides. Additional information can be found in the extended Materials and Methods section in the Supplementary Material online.

### Data Collection

Full list of reptiles with known TSD system was obtained from the Tree of Sex database (Tree of Sex 2014) and literature searches. We searched the literature and dedicated databases for the duration and month intervals of the breeding seasons. We collected information for 101 species with TSD (supplementary tables 1 and 2, Supplementary Material online). Temperature data from the entire surface of the planet were downloaded from the Climatic Research Unit (http://catalogue.ceda.ac.uk/uuid/3df7562727314bab963282e6a0284f24, last accessed May 25, 2020; version 3.24.01). Additional information can be found in the extended Materials and Methods section in the Supplementary Material online.

### Geographical Ranges

Shapefiles for 29 species where downloaded from the RedList database (http://www.iucnredlist.org/, last accessed May 25, 2020; version 3; supplementary table 1, Supplementary Material online). For 72 additional species (supplementary table 2, Supplementary Material online) we generated geographic ranges using the ecological niche modeling routines applying the maximum entropy algorithm in Maxent (Phillips et al. 2006) using the R package kuenm (Cobos et al. 2019). Additional information can be found in the extended Materials and Methods section in the Supplementary Material online.

### Mapping Climate Data to the Species Distribution

We matched the climate data with the species shapefiles using a dedicated R package built by Dr Anna Krystalli as part of the Newton Advanced Fellowship program (https://github.com/annakrystalli/IUCNextractR, last accessed May 25, 2020). We recovered the median temperature (ambient temperature) of all months comprised in the breeding season. Additional information can be found in the extended Materials and Methods section in the Supplementary Material online.

### Synonymous Substitution Analyses

To assess the age at which the XY system was originated in *E. heatwolei*, we followed a previous procedure (Cortez et al. 2014; Marin et al. 2017; Acosta et al. 2019). Briefly, we aligned using PRANK (Loytynoja and Goldman 2005) the coding sequences of XY gametologs in *E. heatwolei* and coding sequences of 1–1 orthologous in other reptiles, mammalian and *Xenopus* species downloaded from the Ensembl database (https://www.ensembl.org/, last accessed May 25, 2020; v.97). We obtained the species’ tree from the TimeTree database (http://www.timetree.org/, last accessed May 25, 2020). We concatenated the alignments and calculated synonymous substitution rates (*d*_S_) using codeml (Yang 1997) and a bootstrap approach. Branch lengths on the species’ tree were used to obtain an ultrametric, time-calibrated, tree using the *chronos* library (*ape* package in R, v5.0) (Paradis and Schliep 2019). The age of the sex chromosomes was obtained from the calibrated branch lengths just before and after the split of the XY gametologs and the time since *E. heatwolei* diverged from the *Snake–Pogona–Anolis* lineage (divergence data retrieved from TimeTree; http://www.timetree.org/, last accessed May 25, 2020). We also calculated the age of the sex chromosomes using BEAST v1.10.4 (http:/beast.bio.ed.ac.uk/), which resulted in an age estimate of ∼93 Ma. We used the relaxed clock and calibrated the tree based on the reptile/mammalian divergence time. We ran the analyses two independent times for 100,000,000 generations, sampling every 1,000 generations. Additional information can be found in the extended Materials and Methods section in the Supplementary Material online. 

## Supplementary Material

evaa104_Supplementary_Data

## Notes

**Data deposition:** This project has been deposited at NCBI-SRA database (www.ncbi.nlm.nih.gov/sra, last accessed May 25, 2020) under the accession BioProject PRJNA573688. Y-linked sequences found in *Eulamprus heatwolei* are available in Supplementary Material online. Shapefiles of geographical ranges of reptiles are available in the figshare platform at the following link https://figshare.com/articles/Reptile_shapefiles/7416638 (last accessed May 25, 2020). The program to map climatic data onto geographical ranges is available in the GitHub platform at the following link https://github.com/annakrystalli/IUCNextractR (last accessed May 25, 2020).
