# Supplementary material for: Viviparous Reptile Regarded to Have Temperature-Dependent Sex Determination Has Old XY Chromosomes
Source: Genome Biol Evol. 2020 May 20;12(6):924–30. doi: 10.1093/gbe/evaa104 (PMC7313667; doi:10.1093/gbe/evaa104)
Supplement: evaa104_Supplementary_Data [file evaa104_supplementary_data.zip › Cornejo.et.al.full.methods.pdf]

Full Methods.

Viviparous reptile regarded to have temperature-dependent sex determination has >90 million-year-old XY chromosomes.

Paola Cornejo-Páramo, Duminda S.B. Dissanayake, Andrés Lira-Noriega, Mónica L. Martínez-Pacheco, Armando Acosta, Ciro Ramírez-Suástegui, Fausto R. Méndez-de-la-Cruz, Tamás Székely, Araxi O. Urrutia, Arthur Georges & Diego Cortez.

Genome and transcriptome data generation

One adult male (Euhea_18_05) and one adult female individual (Euhea_18_03) of *Eulamprus heatwolei* species were captured from a population that inhabits Woods Reserve, Corin Road, ACT, Australia (-35.480751, 148.940398). Both individuals were sacrificed by intraperitoneal injection of pentobarbitone in accordance with the standard operating procedures specified by the animal ethics committee of the University of Canberra. We generated DNA-seq libraries for a male and female *Eulamprus* from liver tissue using the Illumina TruSeq DNA protocol for short insert size (400–450 nt). We generated strand-specific RNA-seq libraries (using the Illumina TruSeq Stranded mRNA Library protocol) for a total of 6 samples obtained from brain, liver, and gonads for a male and female *Eulamprus*. All libraries were sequenced on Illumina HiSeq 2500 sequencers at the University of Canberra. We generated 262-269 million 150-nt paired-end DNAseq reads for each of the male and female genomes. We generated 82-95 million 125-nt paired-end RNAseq reads for each of the male and female tissues. Quality of the reads was verified using FastQC (http://www.bioinformatics.babraham.ac.uk/projects/fastqc) and the remaining adaptors were removed with Trimmomatic (Bolger, et al. 2014). Genomic sequencing coverage (~19X) was estimated based on the *A. carolinensis* genome (1.7 Gb) and later confirmed by the genomic coverage analysis (see below). More details in Supplementary Table 4.

Assembly of Y-linked transcripts and validation

To assemble Y-linked transcripts in *E. heatwolei* we used a subtraction approach based on male and female RNAseq data applied previously in two studies in mammals/birds (Cortez, et al. 2014), the green anole *A. carolinesis* (Marin, et al. 2017), and casque-headed lizards (Acosta, et al. 2019).

We first removed all reads with ambiguous nucleotides (N). Next, we concatenated male RNA-seq reads from the three tissues of *E. heatwolei* into one file and aligned these reads onto the *de novo* reconstructed female transcriptome from *E. heatwolei* using Hisat2 (v2.0.2) (Kim, et al. 2015); reads not mapping were selected; female transcriptome was obtained for all tissues combined (brain, liver, ovary) using Trinity (v2.0.2, default k-mer of 25 bp). We also used the female RNA-seq reads from *E. heatwolei* to build an index of 35 base pairs (bp) k-mers; following a previous procedure (Akagi, et al. 2014). During the development of previous studies, we found that a k-mer size of 35 provided good results at a transcriptome level that allowed the accurate recovering of many Y-linked and W-linked reads in a variety of mammals, birds and reptile species. Larger k-mers resulted in fewer male-specific reads. Smaller k-mers increased the number of false-positive results and potential XY chimeric transcripts. We calculated the frequency of these 35 bp k-mers and removed those showing frequencies below ten; we did not consider rare k-mers as part of the overall signature of the female transcriptome. Rare k-mers are most likely due to erroneous sequencing. We used Bowtie2 (2.1.0) (Langmead and Salzberg 2012) to align the more abundant 35 bp k-mers to the male reads that did not align onto the *E. heatwolei* female transcriptome (with no mismatches and no indels allowed); we selected those male reads with no successful alignments. We obtained only pairs of reads that did not align against the female data and with these few remaining paired-end reads we assembled with Trinity (v2.0.2, default k-mer of 25 bp) (Grabherr, et al. 2011) a transcriptome that were only present in male tissues. We obtained 21,249 transcripts expressed in male tissues (male-biased expression). These transcripts could potentially belong to any chromosomal class (autosomes) and may not necessarily represent Y-linked transcripts. To test for the presence of a Y chromosome, we used blastN (Altschul, et al. 1990) to perform sequence searches in the male and female genomic reads we produced for this project at a 100% identity threshold. Short genomic reads (150bp) may only partially contain exons but would mostly be constituted of intergenic and intronic sequences. blastN searches only report the portion of the genomic read that aligns to the transcripts. That is, blastN only report the exon segment present on the genomic read. All intronic and intergenic sequences that cannot map to our transcripts are left out. To expedite the analysis, we searched the transcripts (fewer sequences) onto the genomic raw reads, which were used to generate the blastN database. Male transcripts were marked as Y-linked when showing 4X-14X of averaged coverage of male genomic reads and zero averaged coverage of female genomic reads (Supplementary Table 3). Out of 21,249 transcripts, 350 transcripts were present in both the male transcriptome and the male

genome (Supplementary Table 3) and were considered as Y-linked.

Initially, we did not know whether *E. heatwolei* presented XY or ZW chromosomes. So, in parallel, we ran the methodology starting with female RNA-seq data to determine the presence of potential W-linked sequences. We found 27,705 transcripts with female-biased expression. All the 27,705 transcripts aligned perfectly to both male and female genomic reads. So, they belong to genes located on autosomes or the X chromosome, which are chromosomes shared between males and females.

To establish Y gene identity, we searched NCBI GenBank (http://www.ncbi.nlm.nih.gov/genbank) with blastN and blastX for the closest homologs in the Reptile taxa and identified transcripts that coded for 14 proteins (Supplementary Table 3). BlastX searches also allowed the identification of CDS regions. We searched the 350 Y-linked transcripts. Of these, around 52 transcripts mapped to the same 14 protein-coding genes (Supplementary Table 3). Other transcripts (with several isoforms each) did not show significant matches and could be long-non-coding RNAs or species-specific repeated elements or ampliconic elements (testis-specific Y transcripts).

For these 14 Y-linked protein-coding genes, we performed blastN (Altschul, et al. 1990) searches against the *de novo* reconstructed female transcriptome from *E. heatwolei* to obtain the best match (95-97% identity over the entire sequence), which we considered as X gametologs. We verified the X gametologs based on coverage analyses using male and female genomic reads and GenBank searches that resulted in the same gene identity previously established for Y genes. Visual inspection of complete XY gametologs alignments is very helpful to detect inconsistencies in the XY sequences.

The 14 XY gametologs in *E. heatwolei* were also searched against the *A. carolinensis* and chicken genomes using the sequence search engine at the ENSEMBL webpage (https://www.ensembl.org/Multi/Tools/Blast) to establish whether they formed a syntenic block.

To further reinforce our results, we decided to amplify a subset of three Y-linked genes by standard PCR approach using whole genomic DNA from *E. heatwolei* (n = 14; seven males and seven females). Seven males and seven females tail snips were collected from a population that inhabits Woods Reserve, Corin Road, ACT, Australia (-35.480751, 148.940398). Sex was determined by manual eversion of hemipenes. DNA was extracted using a commercial kit (Gentra

Puregene, QIAGEN) following manufacturer protocols, including RNase A treatment. DNA was eluted in 100 µL AE buffer (QIAGEN), preheated to 65°C. DNA concentration assessed for purity using a NanoDrop 1000 spectrophotometer (NanoDrop Technologies, Wilmington, 19810, USA) and quantified using Qubit 2.0 fluorometer (Invitrogen, Life technologies, Sydney, NSW, Australia). The primers used were: *COL1A1* (autosomal/control) forward: TCACACTGGCAGTTGGGGAAAGAC, reverse: ATTCCTGGTCTGGGGCACCAAC. Whereas Y-linked genes were: *BTG1,* forward: AAAACAACAGCACTGTCG, reverse: CACAGTTTTGAAGATGTGGCAC. *BTG1,* forward: CAACAGCACTGTCGCTATA, reverse: GTCACAGTTTTGAAGATGTGG. *BTG1,* forward: TCTTGTGCCACATCTTCAAAAC, reverse: GTAGTTCTTACTTTGAGTGGGCT. *TNPO3,* forward: TTGGAAATCCTGATAGTTCTGC reverse: ATTGGCTCCAATGCGTAAGG. *PPP1R12A,* forward: CATCACTACAGAGCAACTTC, reverse: TAGAACTTGTGGCAGTTG. These primers were designed to specifically amplify Y sequences. Nucleotide sequences for XY gametologs (transcripts) were aligned using Muscle (Edgar 2004) and the mismatches were marked (lowercase) on the Y sequence. Hundreds of primers were then designed for the Y sequence using AmplifX2 v.2.0.6 a program designed by Nicolas Jullien (https://inp.univ-amu.fr/en/amplifx-manage-test-and-design-your-primers-for-pcr). We then selected those pairs of primers that contained the maximum number of mismatches between XY sequences and that would amplify a genomic region of ~100nt within the same exon. Exon/intron boundaries were obtained from the *A. carolinensis* reference genome. Three Y genes met the PCR products size restriction. The PCR screening process was conducted in three stages. Initially, we first screened the two sequenced individuals to confirm presence of an amplified fragment in the male and the absence of an amplified fragment in the female individual. Subsequently, in the second stage, we screened two males and two females. Finally, we performed a panel of 7 males and 7 females. Each PCR reaction contained 1x My Taq HS Red mix (Bioline, cat. BIO-25047), 1µl of each of the forward and reverse primers at 4µM and 2µl of template DNA at approximately 40 ng/µl. The PCR cycling conditions used a touchdown phase to increase specificity of annealing: denaturing at 95˚C, annealing temperature stepping down from 70˚C by 0.5˚C for 10 cycles, extension at 72˚C. This was followed by 40 cycles at 59.3˚C annealing and 72˚C extension. PCR reactions were run on agarose gels (2%).

We retrieved the Y-linked markers in *N. ocellatus* (Hill, et al. 2018) and used blastN (e-value < 0.01) to map these sequences onto the reconstructed *E. heatwolei* chromosomes and the *A. carolinenesis* reference genome downloaded from the Ensembl database (https://www.ensembl.org/; v.97). More details in Supplementary Table 3. We calculated the

Fisher-exact test using the total number of Y markers, the number of Y markers with successful alignments and how many of these Y markers landed on the *Eulamprus* MSY.

Genomic coverage analyses

The quality of the reads was verified using FastQC (http://www.bioinformatics.babraham.ac.uk/projects/fastqc) and the remaining adaptors were removed with Trimmomatic (Bolger, et al. 2014). We then followed a methodology previously applied to the analysis of sex chromosomes in snakes (Vicoso, et al. 2013). Specifically, the male and female raw genomic reads were assembled into scaffolds using SOAP *de novo* (v.2; default parameters) (Luo, et al. 2012), and the resulting scaffolds were aligned against the *A. carolinensis* reference genome using BWA (bwa-mem) (Li, et al. 2009). The selected scaffolds in the male and female eulamprus genomes were required to align over 30% of their sequence length and above 70% of identity against the *A. carolinensis* genome. We ordered the eulamprus scaffolds following the sequence of the chromosomes in *A. carolinensis*. We used bowtie2 (Langmead and Salzberg 2012) to align the DNA-seq reads from the male and female eulamprus onto the reconstructed chromosomes. The aligned reads were sorted using SAMtools (Li, et al. 2009). Coverage along the chromosomes were calculated using BEDtools (Quinlan and Hall 2010). To compute the final data illustrated in the figures, we averaged the coverage using windows of 100,000 nucleotides

We also used the XY gametolog nucleotide sequences as queries to perform blastN (Altschul, et al. 1990) searches against the male and female raw DNA-seq reads. From the resulting alignments we calculated the median coverage for each gametolog for the male genomic reads and the female genomic reads. We repeated the male and female coverage analysis for 1,000 randomly selected autosomal genes.

Data collection

We collected the full list of reptiles with known TSD system from the Tree of Sex database (Tree of Sex 2014) and literature searches. We then searched the literature and dedicated reptile databases for the duration and month intervals of the breeding seasons. We collected information for 101 species with TSD and 99 species with GSD (Supplementary Tables 1-2). Temperature data from the entire surface of the planet was downloaded from the Climatic Research Unit (http:// http://catalogue.ceda.ac.uk/uuid/3df7562727314bab963282e6a0284f24; version 3.24.01).

Geographical ranges

We collected the full list of reptiles with a known sex determination system from the Tree of Sex database (Tree of Sex 2014). We divided the sex determination systems of the species into two categories: genotypic sex determination (GSD) and temperature-dependent sex determination (TSD). We then searched the literature and dedicated reptile databases for the duration and month intervals of the breeding seasons for all reptile species with known sex determination systems. The detailed records for the species with the collected information and their corresponding references are listed in Supplementary Tables 1-2. When multiple breeding seasons were found for a given species, the consensus period was selected. Several species names reported in the Tree of Sex database have changed (the species have been reclassified) and did not follow the current species classification (*e.g.* different genus). Therefore, we worked with all synonyms obtained from the RedList database (http://www.iucnredlist.org/; version 3), the Reptile Database (http://www.reptile-database.org/) and the Mexican Collection of Reptiles (http://www.ib.unam.mx/zoologia/#colecciones-zoologicas-nacionales) to solve the inconsistencies. We only used the current names for each species in strict agreement with the names used in the RedList database. The list of synonyms can be found in Supplementary Tables 1-2. Lastly, for each species, we downloaded the shapefiles of their distributions from the RedList database (http://www.iucnredlist.org/; version 3). For 128 species we successfully collected the sex-determination system and the shapefile of their geographical distribution. Of these, 99 species show GSD, and 39 species have TSD. Temperature and precipitation data from the entire surface of the planet were downloaded from the Climatic Research Unit (http:// http://catalogue.ceda.ac.uk/uuid/3df7562727314bab963282e6a0284f24; version 3.24.01), a database that has month-by-month variations in climate over the period 1901-2015, on high-resolution (0.5x0.5 degree) grids.

Generation of distributional ranges for additional species

We generated shapefiles for 72 species with TSD. To characterize the species' geographic ranges, we implemented ecological niche modeling routines using the maximum entropy algorithm in Maxent (Phillips, et al. 2006). This was done by searching for best candidate models using the R package kuenm developed by Dr Marlon E. Cobos (https://github.com/marlonecobos/kuenm). We first compiled the species' occurrences through several databases including the Global Biodiversity Information Facility (GBIF; https://www.gbif.org/), Biodiversity Information Serving Our Nation

(BISON; https://bison.usgs.gov/#home), Berkeley Ecoinformatics Engine (Ecoengine; https://ecoengine.berkeley.edu/), Integrated Digitized Biocollections (iDigBio; https://www.idigbio.org/tags/database), Atlas of Living Australia (ALA; https://www.ala.org.au/), iNaturalist (https://www.inaturalist.org/), VertNet (http://vertnet.org/), and Ocean Biogeographic Information System (OBIS; http://www.iobis.org/). All the occurrences per species were cleaned from obvious georeferencing errors or taxonomic misidentifications through careful inspection in a GIS (Geographic Information System) and based on information gathered from The Reptile Database, which contains updated taxonomic information (http://reptile-database.reptarium.cz/). The number of occurrences per species ranged between 1 and 13192, although the majority (76 species; 89.4%) had more than 25 occurrences. Only seven species had less than 12 occurrences and were treated as data deficient species; for four of these data deficient species we decided to use alternative geographic range estimations rather than the outcome of a niche model (see below) as follows: for two of these data deficient species (*Mauremys nigricans*, *Podocnemis erythrocephala*) we decided to use the ecoregions (Olson, et al. 2001) that intersected with their occurrences as a proxy of their distribution after carefully inspecting that these ranges corresponded to the species known distributions, and for two other data deficient species (*Phelsuma guentheri*, *Phelsuma guimbeaui*) that are located in the Mauritius island, we used the polygon of the island as a proxy of their geographic range. For the rest of the data deficient species (*Geochelone elephantopus*, *Mauremys annamensis*, *Eurotestudo hermanni*) we conducted niche modeling as described as follows. For the species with more than 12 occurrences, the points representing the locations of each species were then used to intersect the terrestrial ecoregions of the world (Olson, et al. 2001), to which we then added a 1-degree buffer to use as the accessibility area (Barve, et al. 2011); this area was implemented to mask the environmental layers that were then needed to calibrate the models. All occurrences were spatially filtered with a 20 km radius to avoid spatial autocorrelation and overfitting the models. Models were calibrated with the seven least correlated bioclimatic layers from the WorldClim database (http://www.worldclim.org/) at a spatial resolution of 2.5 minutes: Annual Mean Temperature; Mean Diurnal Range (Mean of monthly; max temp - min temp); Max Temperature of Warmest Month; Min Temperature of Coldest Month; Annual Precipitation; Precipitation of Wettest Month; Precipitation of Driest Month. Candidate models were explored setting up the maximum entropy algorithm in Maxent (Phillips, et al. 2006) in the R package kuenm (Cobos, et al. 2019) (https://github.com/marlonecobos/kuenm) with all possible combination of features and

regularization multipliers of 0.1, 0.5, 1, 2, 3, and 4. This generated 348 models per species, which were then subjected to evaluation to select the best model parameterization based on statistical significance according to the partial Receiver Operative Characteristic (Peterson, et al. 2008) (ROC), omission rate (Peterson, et al. 2008) (i.e., a user-selected proportion of occurrence data that may present meaningful errors), and model complexity (Akaike Information Criterion for small samples (AICc)). Evaluation parameters were set to 10 percent for omission rate, with 50 percent randomly sampled occurrence points from the test data for bootstrapping, and 500 iterations for bootstrapping; such procedure produced a table with the best models corresponding to those with highest mean AUC ratio from the partial ROC, lower omission rate and AICc, as well as number of parameters per model. When more than one best model was obtained, we selected the best one based on the highest value of the AUC mean ratio. Finally, the specific parameterization from the best model was used to re-run the Maxent procedure and generate the final model, which represented the best estimate of the species' geographic range. The minimum criteria regarding the outcome of model evaluation was the statistical significance of the model ($P < 0.001$) and an AUC ratio > 1.4, considering that values of AUC ratio that depart upwards from one perform better than random(Peterson, et al. 2008). The final model per species was based on the raster of the median of 10 model projections of that best model in each species accessible area. To depict the geographic potential distribution of each species, each raster was thresholded based on the 10-percentile training presence. The geographic projection of the resulting binary map was then converted to a shapefile. The resulting shapefiles are available in the figshare platform at the following link https://figshare.com/articles/Reptile_shapefiles/7416638.

Mapping climate data to the species distribution

Shapefiles downloaded from the RedList database (http://www.iucnredlist.org/; version 3) and shapefiles generated for this project contain polygons with geographical coordinates (latitude and longitude) representing the species distribution. The environmental data from CRU (http:// http://catalogue.ceda.ac.uk/uuid/3df7562727314bab963282e6a0284f24; version 3.24.01) has climate surfaces gridded at a spatial resolution of 0.5x0.5 degrees. We matched the climate data with the species shapefiles using a dedicated R package built by Dr. Anna Krystalli as part of the Newton Advanced Fellowship program. The R package used in the study is available at https://github.com/annakrystalli/IUCNextractR. Briefly, the R package extracted the climate grids

that overlap with the species' polygons and returned, for each shapefiles, the average monthly temperature for a given time period. In our case, we selected a 30-year time period of climate data, 1960-1990. The beginning and end months of the breeding season were recorded in a numerical format, where 1 was January, 2 was February, etc., until 12 represented December. We then recovered the median temperature (ambient temperature) of all months comprised in the breeding season for 1960-1990.

Synonymous substitution analyses

To assess the age at which the novel XY system was originated, we followed a previous procedure (Cortez, et al. 2014; Marin, et al. 2017). Specifically, we aligned using PRANK (codon-based option) (Loytynoja and Goldman 2005) the coding sequences of XY genes in *E. heatwolei* and the coding sequences from one-to-one orthologous genes in *A. carolinensis*, *Pogona vitticeps*, the tiger snake (*Notechis scutatus*), the tuatara, chicken, the soft-shell Chinese turtle, opossum, mouse, human and *Xenopus;* orthologous coding sequences were downloaded from the Ensembl database (https://www.ensembl.org/; v.97). We obtained the species' tree from the TimeTree database (http://www.timetree.org/). We concatenated the individual gene alignments and applied a bootstrap approach (100 rounds). For each round, we calculated the synonymous substitutions for all branches of the species' tree using codeml (implemented in PAML (Yang 1997)). The average branch lengths from the 100 bootstraps were standardized to produce an ultrametric, time-calibrated, tree using the *chronos* library (*ape* package in R, v5.0) (Paradis and Schliep 2019). We then retrieved the branch lengths just before and after the split of the XY gametologs and the time since *E. heatwolei* diverged from the *Snake-Pogona-Anolis* lineage (out-group species). We calculated the age of the sex chromosomes base on these values. That is, we distributed the time since these species diverged proportionally according to the branch lengths just before and after the XY split. The divergence time between species was retrieved from TimeTree (http://www.timetree.org/). We also calculated the age of the sex chromosomes using BEAST v1.10.4 (http:/beast.bio.ed.ac.uk/). For this analysis, we used the relaxed clock and calibrated the tree based on the reptile/mammalian divergence time, GTR+I+G for the substitution model (estimated for the dataset), and a normal distribution with SD of 1 as priors on the calibration nodes to accommodate calibration uncertainty. Gene boundaries were marked in the concatenated alignment. We only used an old node for time calibration (the reptile/mammalian node; data retrieved from the TimeTree database; http://www.timetree.org/) because we did not

want to fix recent nodes that could constrain the analysis close to the origin of the sex chromosomes. We ran the analyses two independent times for 100,000,000 generations, sampling every 1,000 generations. The average age estimate of the two independent runs resulted in a XY split of ~93 million years old (Supplementary Figure 4).

References

Acosta A, Suarez-Varon G, Rodriguez-Miranda LA, Lira-Noriega A, Aguilar-Gomez D, Gutierrez-Mariscal M, Hernandez-Gallegos O, Mendez-de-la-Cruz F, Cortez D. 2019. Corytophanids Replaced the Pleurodont XY System with a New Pair of XY Chromosomes. Genome Biol Evol 11:2666-2677.
Akagi T, Henry IM, Tao R, Comai L. 2014. Plant genetics. A Y-chromosome-encoded small RNA acts as a sex determinant in persimmons. Science 346:646-650.
Altschul SF, Gish W, Miller W, Myers EW, Lipman DJ. 1990. Basic local alignment search tool. J Mol Biol 215:403-410.
Barve N, Barve V, Jimenez-Valverde A, Lira-Noriega A, Maher SP, Peterson AT, Soberon J, Villalobos F. 2011. The crucial role of the accessible area in ecological niche modeling and species distribution modeling. Ecological Modelling 222:1810-1819.
Bolger AM, Lohse M, Usadel B. 2014. Trimmomatic: a flexible trimmer for Illumina sequence data. Bioinformatics 30:2114-2120.
Cobos ME, Peterson AT, Barve N, Osorio-Olvera L. 2019. kuenm: an R package for detailed development of ecological niche models using Maxent. Peerj 7:e6281.
Cortez D, Marin R, Toledo-Flores D, Froidevaux L, Liechti A, Waters PD, Grutzner F, Kaessmann H. 2014. Origins and functional evolution of Y chromosomes across mammals. Nature 508:488-493.
Edgar RC. 2004. MUSCLE: multiple sequence alignment with high accuracy and high throughput. Nucleic Acids Res 32:1792-1797.
Grabherr MG, Haas BJ, Yassour M, Levin JZ, Thompson DA, Amit I, Adiconis X, Fan L, Raychowdhury R, Zeng Q, et al. 2011. Full-length transcriptome assembly from RNA-Seq data without a reference genome. Nat Biotechnol 29:644-652.
Hill PL, Burridge CP, Ezaz T, Wapstra E. 2018. Conservation of Sex-Linked Markers among Conspecific Populations of a Viviparous Skink, *Niveoscincus ocellatus*, Exhibiting Genetic and Temperature-Dependent Sex Determination. Genome Biol Evol 10:1079-1087.
Kim D, Langmead B, Salzberg SL. 2015. HISAT: a fast spliced aligner with low memory requirements. Nat Methods 12:357-360.
Langmead B, Salzberg SL. 2012. Fast gapped-read alignment with Bowtie 2. Nat Methods 9:357-359.
Li H, Handsaker B, Wysoker A, Fennell T, Ruan J, Homer N, Marth G, Abecasis G, Durbin R, Genome Project Data Processing S. 2009. The Sequence Alignment/Map format and SAMtools. Bioinformatics 25:2078-2079.
Loytynoja A, Goldman N. 2005. An algorithm for progressive multiple alignment of sequences with insertions. Proc Natl Acad Sci U S A 102:10557-10562.
Luo R, Liu B, Xie Y, Li Z, Huang W, Yuan J, He G, Chen Y, Pan Q, Liu Y, et al. 2012. SOAPdenovo2: an empirically improved memory-efficient short-read de novo assembler. Gigascience 1:18.
Marin R, Cortez D, Lamanna F, Pradeepa MM, Leushkin E, Julien P, Liechti A, Halbert J, Bruning T, Mossinger K, et al. 2017. Convergent origination of a Drosophila-like dosage compensation mechanism in a reptile lineage. Genome Res 27:1974-1987.

Olson DM, Dinerstein E, Wikramanayake ED, Burgess ND, Powell GVN, Underwood EC, D'Amico JA, Itoua I, Strand HE, Morrison JC, et al. 2001. Terrestrial ecoregions of the worlds: A new map of life on Earth. Bioscience 51:933-938.
Paradis E, Schliep K. 2019. ape 5.0: an environment for modern phylogenetics and evolutionary analyses in R. Bioinformatics 35:526-528.
Peterson AT, Papes M, Soberon J. 2008. Rethinking receiver operating characteristic analysis applications in ecological niche modeling. Ecological Modelling 213:63-72.
Phillips SJ, Anderson RP, Schapire RE. 2006. Maximum entropy modeling of species geographic distributions. Ecological Modelling 190:231-259.
Quinlan AR, Hall IM. 2010. BEDTools: a flexible suite of utilities for comparing genomic features. Bioinformatics 26:841-842.
Tree of Sex C. 2014. Tree of Sex: a database of sexual systems. Sci Data 1:140015.
Vicoso B, Emerson JJ, Zektser Y, Mahajan S, Bachtrog D. 2013. Comparative sex chromosome genomics in snakes: differentiation, evolutionary strata, and lack of global dosage compensation. PLoS Biol 11:e1001643.
Yang Z. 1997. PAML: a program package for phylogenetic analysis by maximum likelihood. Comput Appl Biosci 13:555-556.
