# Supplementary material for: Viviparous Reptile Regarded to Have Temperature-Dependent Sex Determination Has Old XY Chromosomes
Source: Genome Biol Evol. 2020 May 20;12(6):924–30. doi: 10.1093/gbe/evaa104 (PMC7313667; doi:10.1093/gbe/evaa104)
Supplement: evaa104_Supplementary_Data [file evaa104_supplementary_data.zip › Fastq_reports_DNAseq.RNAseq.RAWdata_compressed.pdf]

FastQC Report

Wed 9 Oct 2019
EHF_18_05_S2_R1_001.fastq.gz

Summary

- Basic Statistics
- Per base sequence quality
- Per tile sequence quality
- Per sequence quality scores
- Per base sequence content
- Per sequence GC content
- Per base N content
- Sequence Length Distribution
- Sequence Duplication Levels
- Overrepresented sequences
- Adapter Content

Basic Statistics

Measure	Value
Filename	EHF_18_05_S2_R1_001.fastq.gz
File type	Conventional base calls
Encoding	Sanger / Illumina 1.9
Total Sequences	134702419
Sequences flagged as poor quality	0
Sequence length	35-151
%GC	47

Per base sequence quality

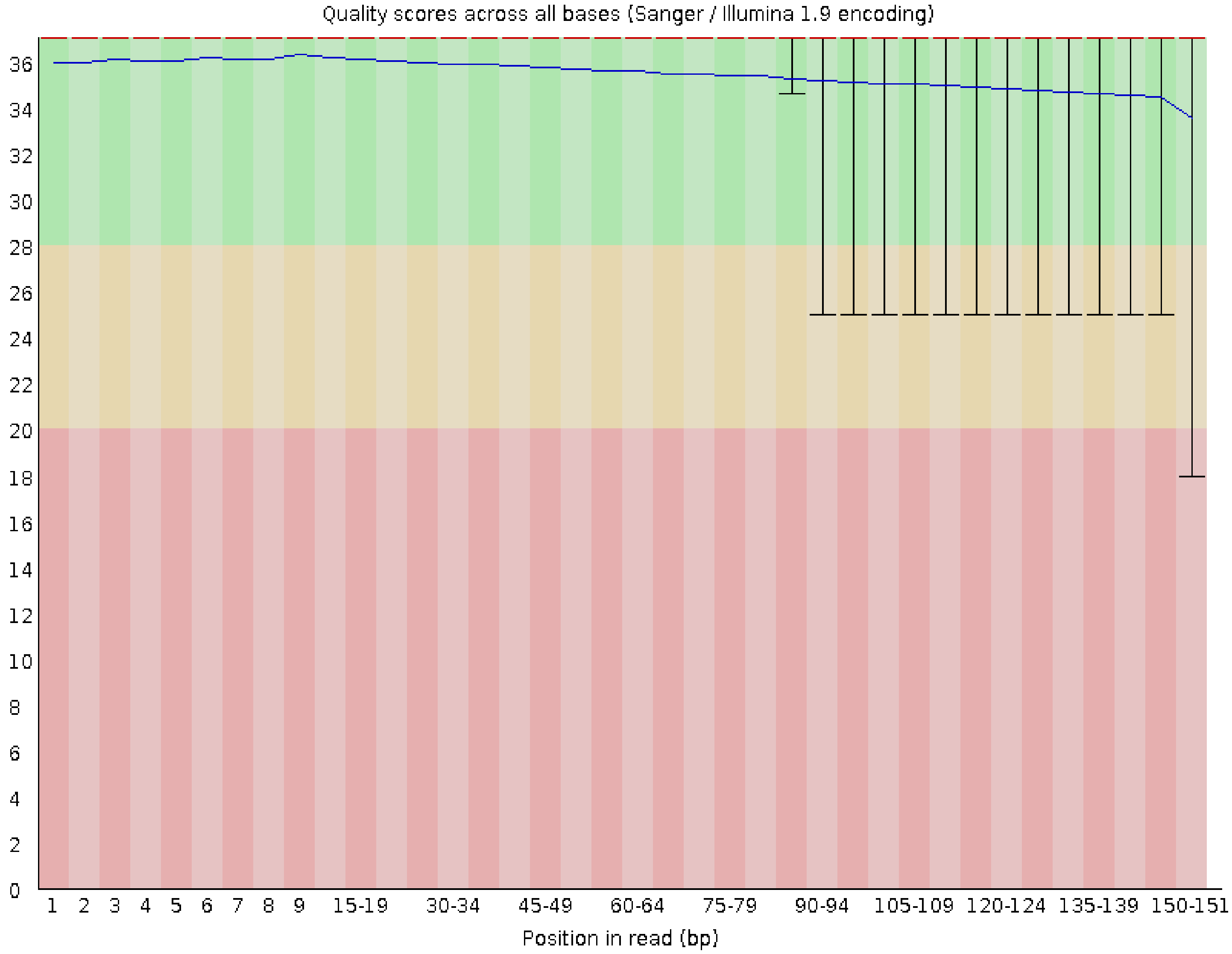

Per tile sequence quality

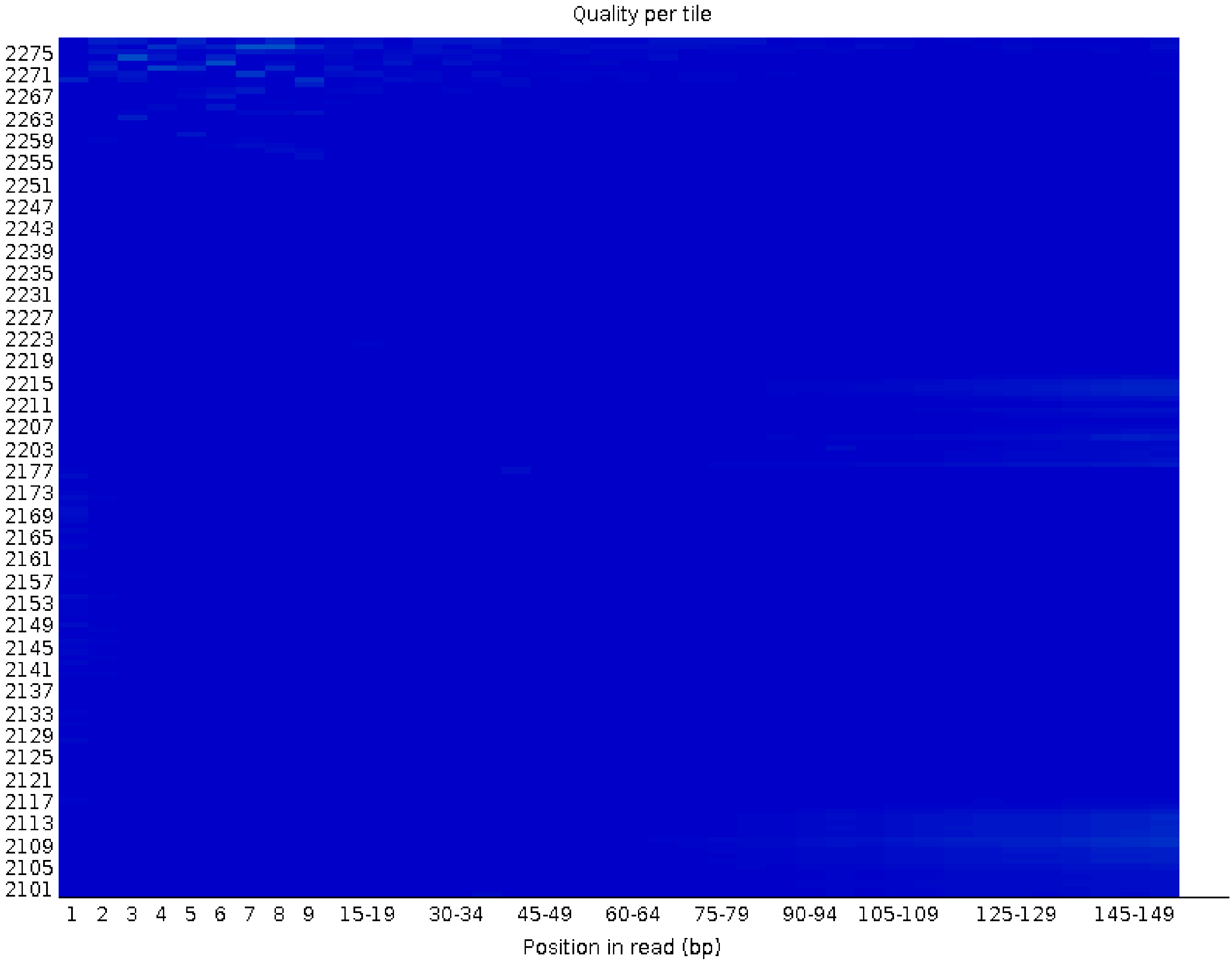

Per sequence quality scores

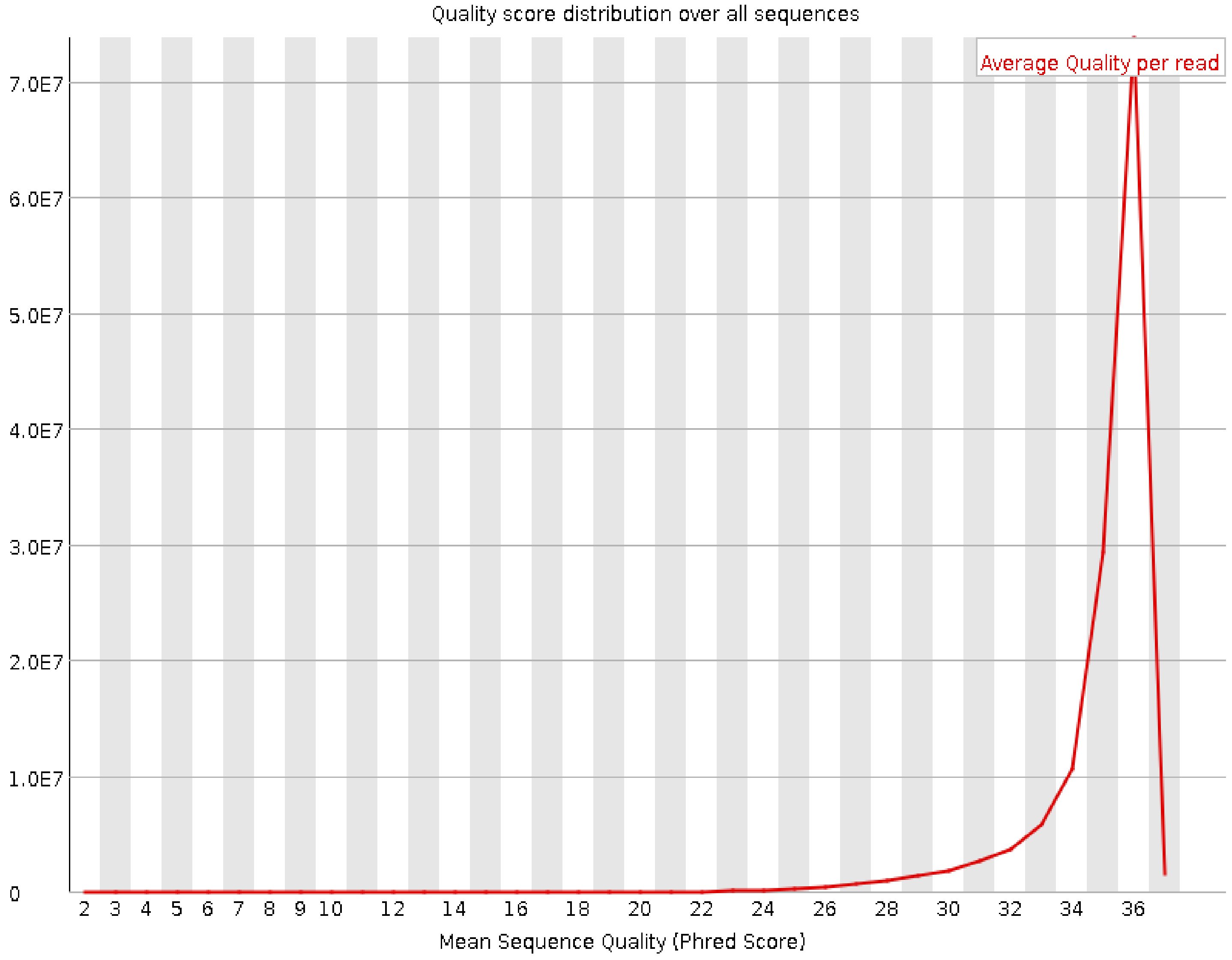

Per base sequence content

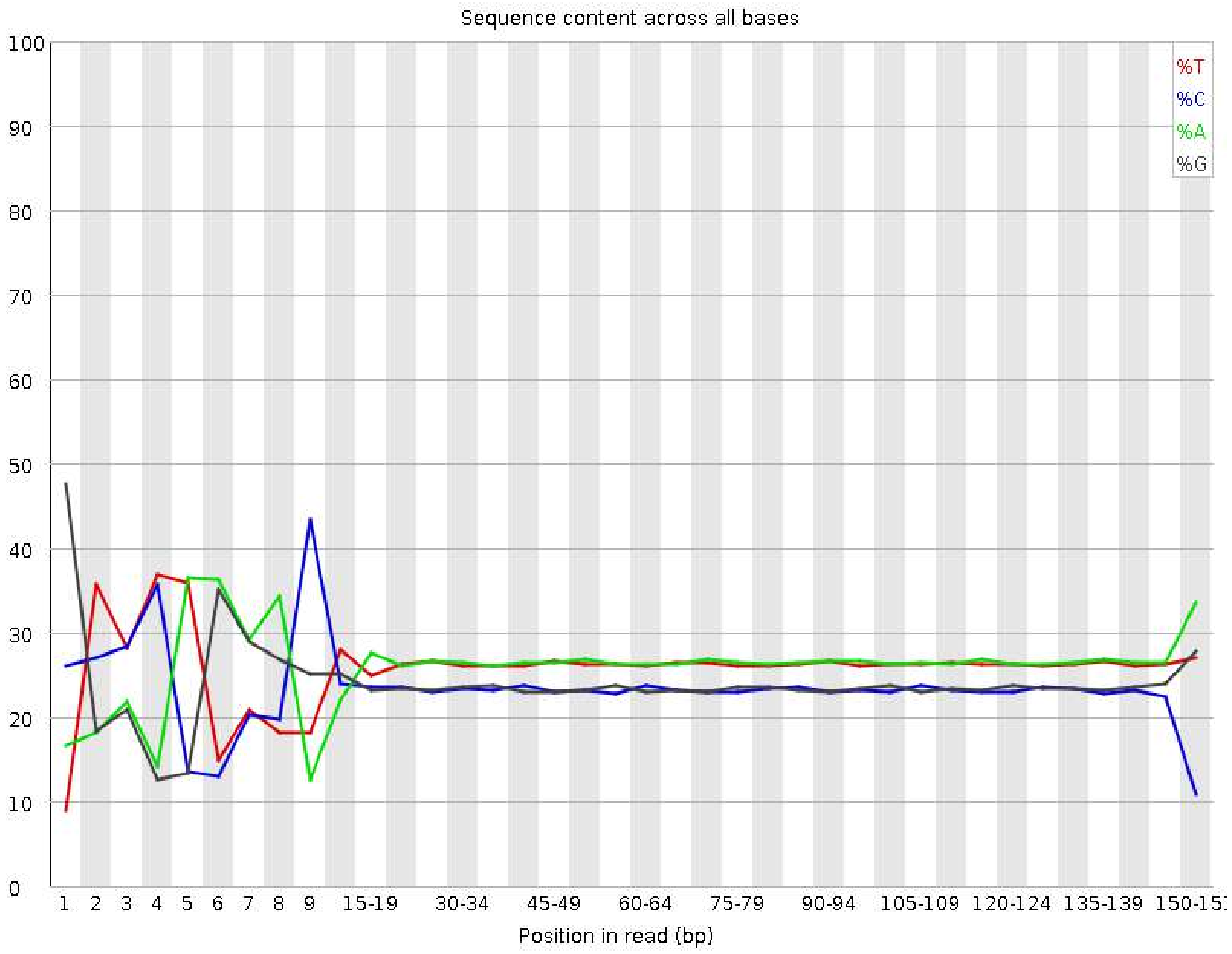

Per sequence GC content

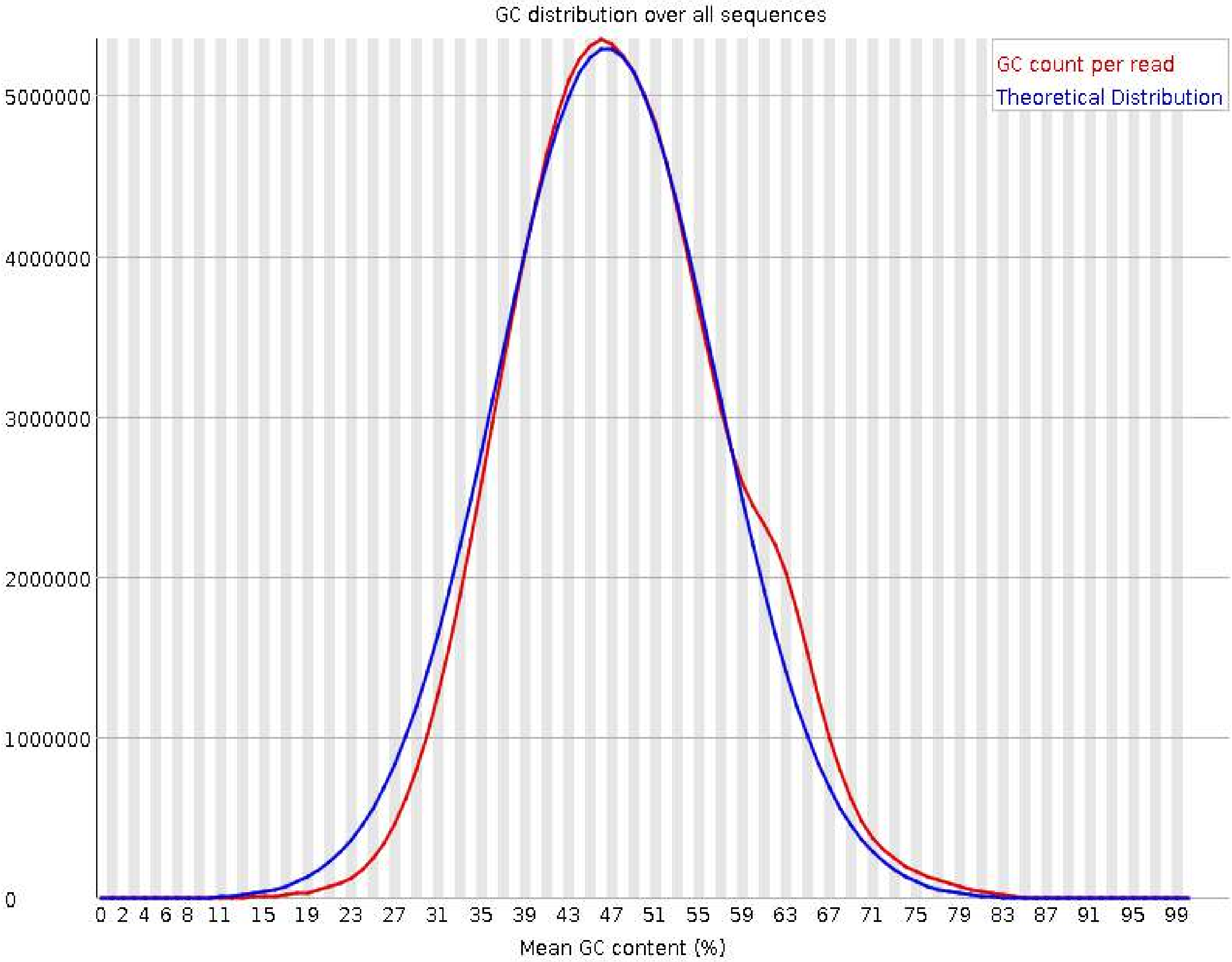

Per base N content

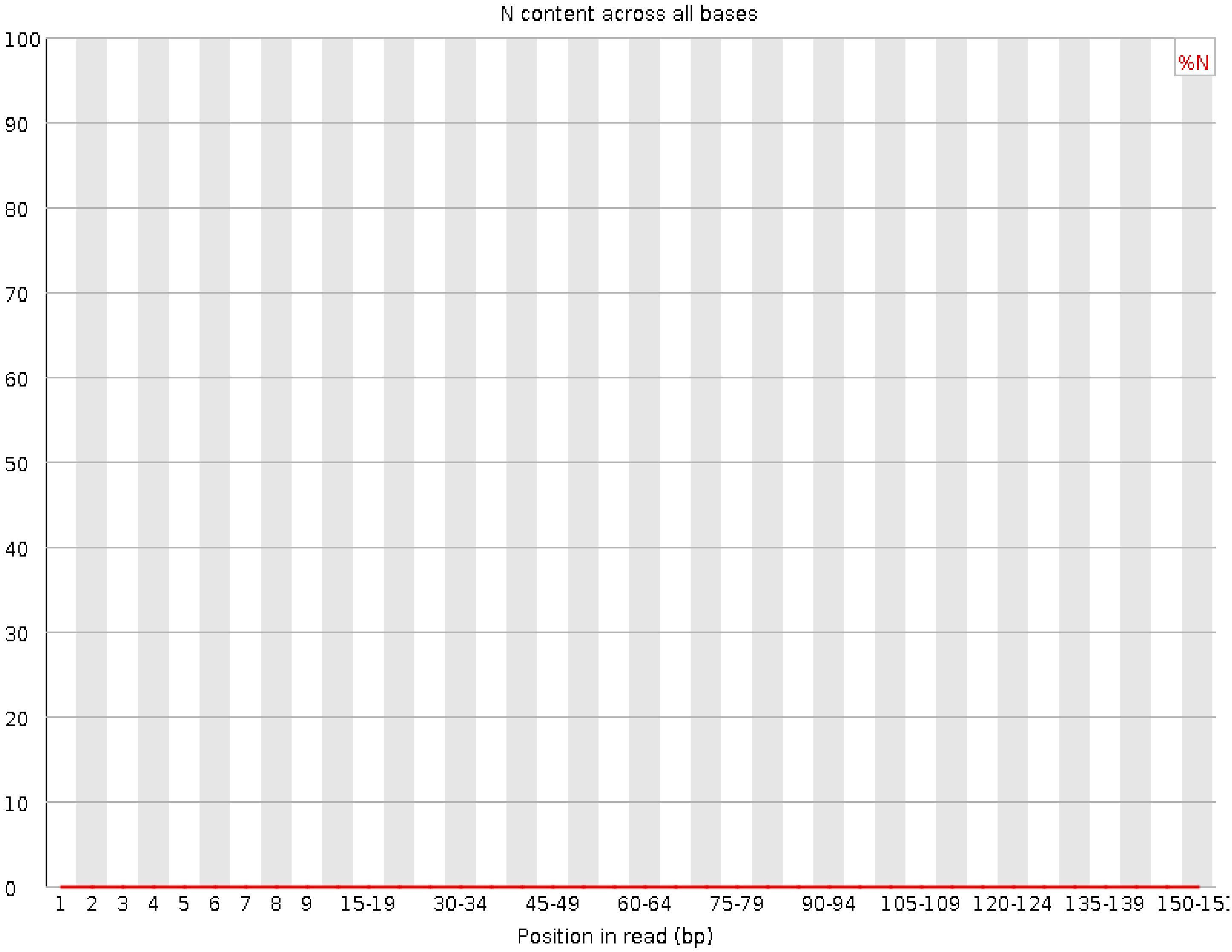

Sequence Length Distribution

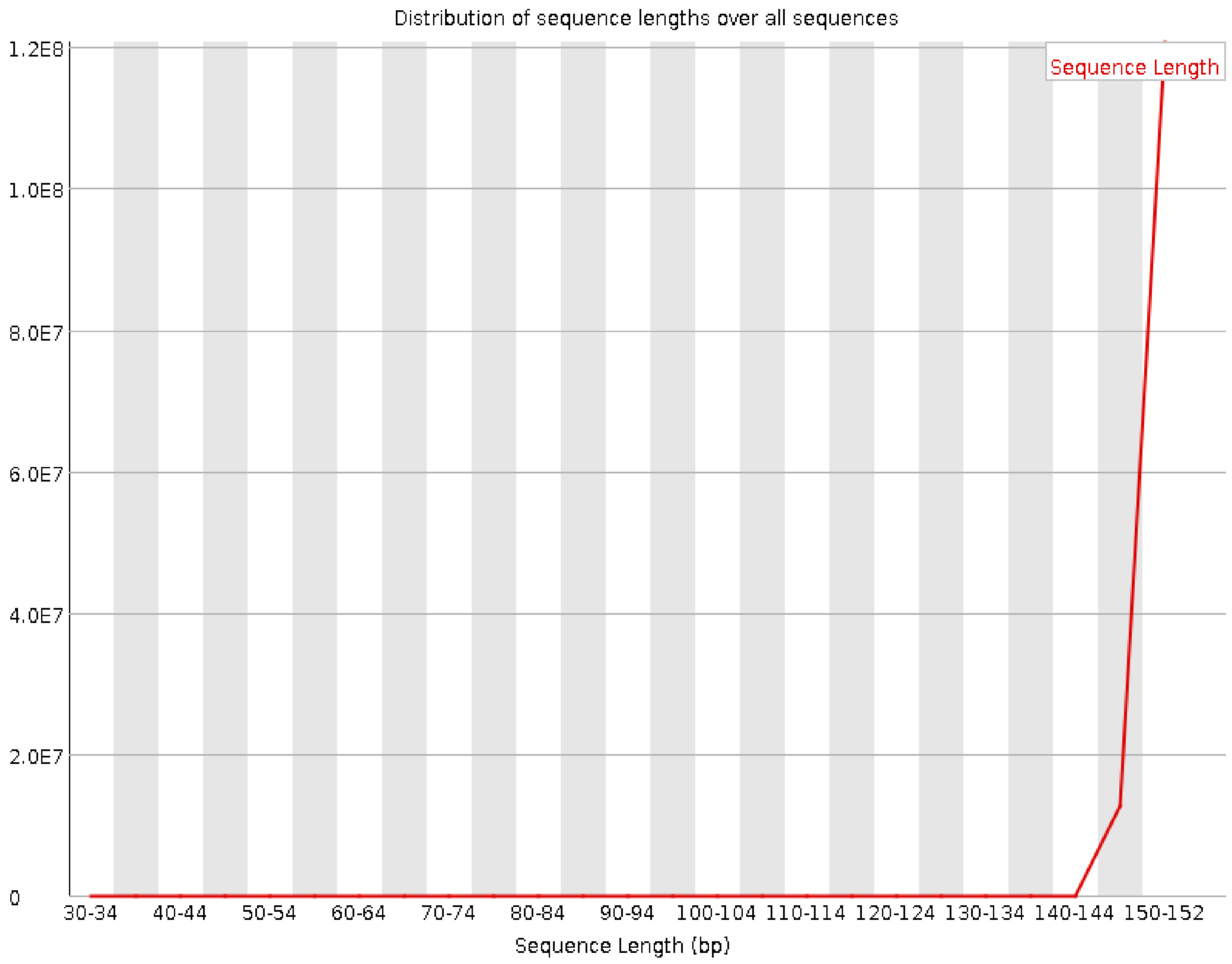

Sequence Duplication Levels

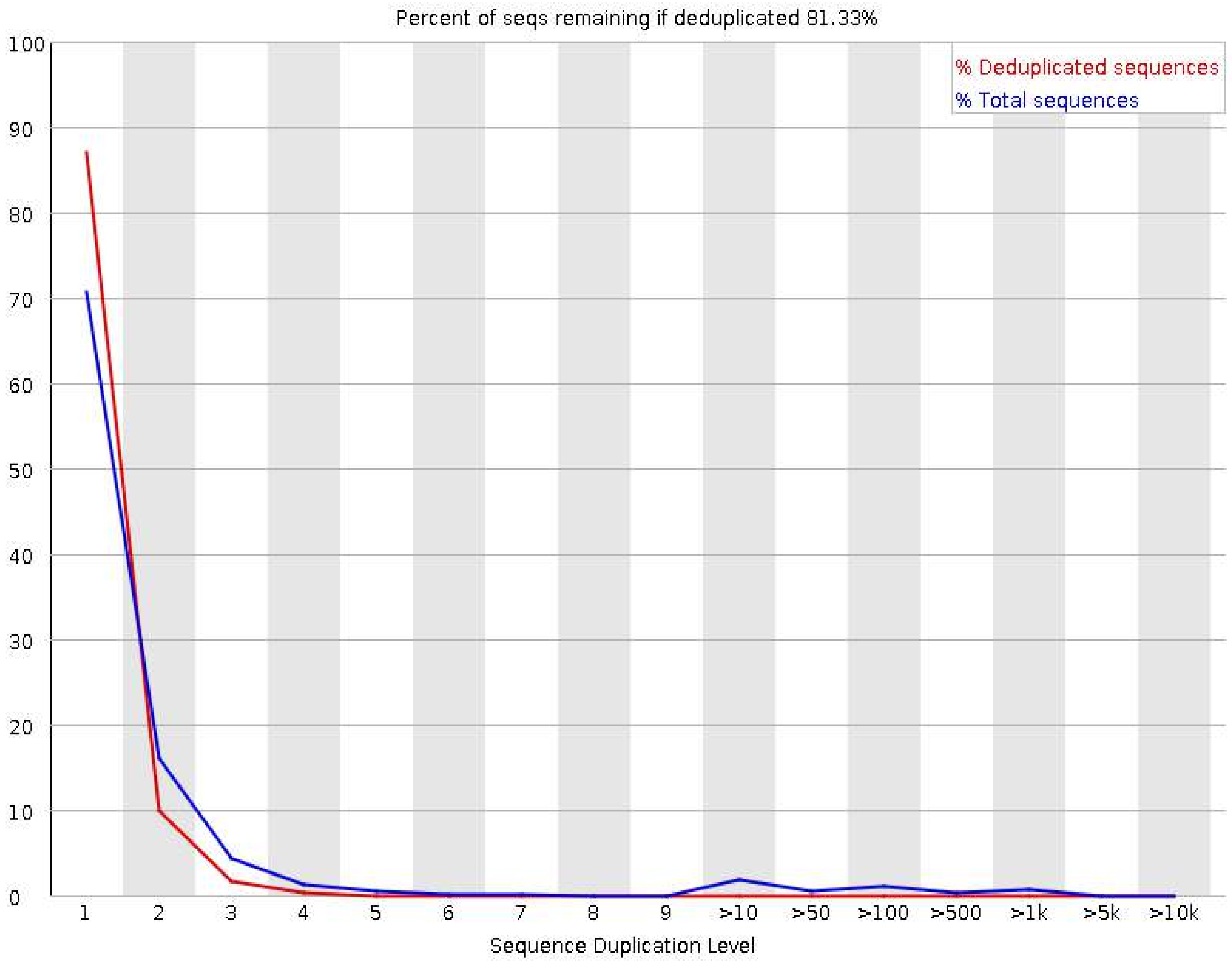

Overrepresented sequences

No overrepresented sequences

Adapter Content

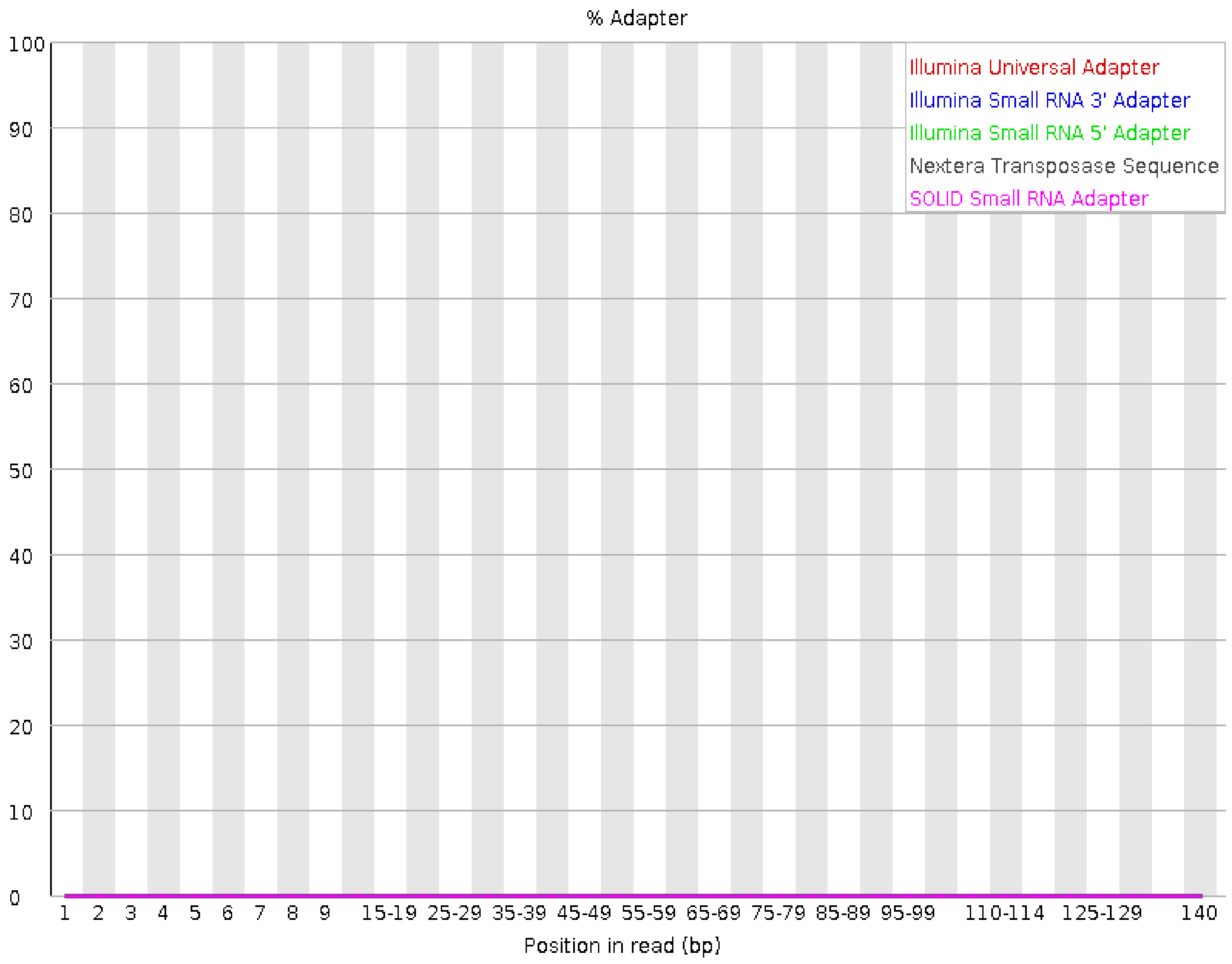

Produced by FastQC (version 0.11.8)

FastQC Report

Wed 9 Oct 2019
EHF_18_05_S2_R2_001.fastq.gz

Summary

- Basic Statistics
- Per base sequence quality
- Per tile sequence quality
- Per sequence quality scores
- Per base sequence content
- Per sequence GC content
- Per base N content
- Sequence Length Distribution
- Sequence Duplication Levels
- Overrepresented sequences
- Adapter Content

Basic Statistics

Measure	Value
Filename	EHF_18_05_S2_R2_001.fastq.gz
File type	Conventional base calls
Encoding	Sanger / Illumina 1.9
Total Sequences	134702419
Sequences flagged as poor quality	0
Sequence length	35-151
%GC	47

Per base sequence quality

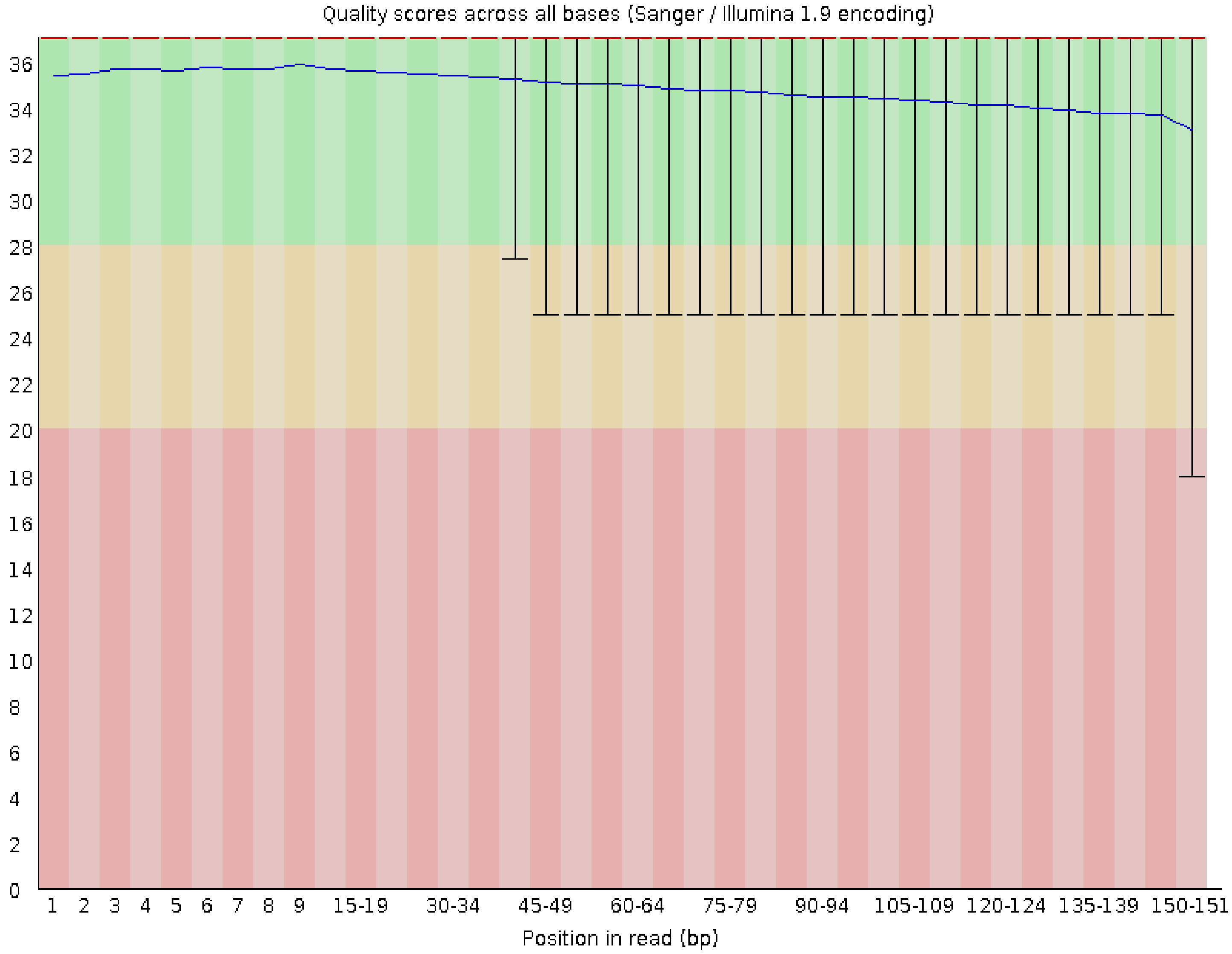

Per tile sequence quality

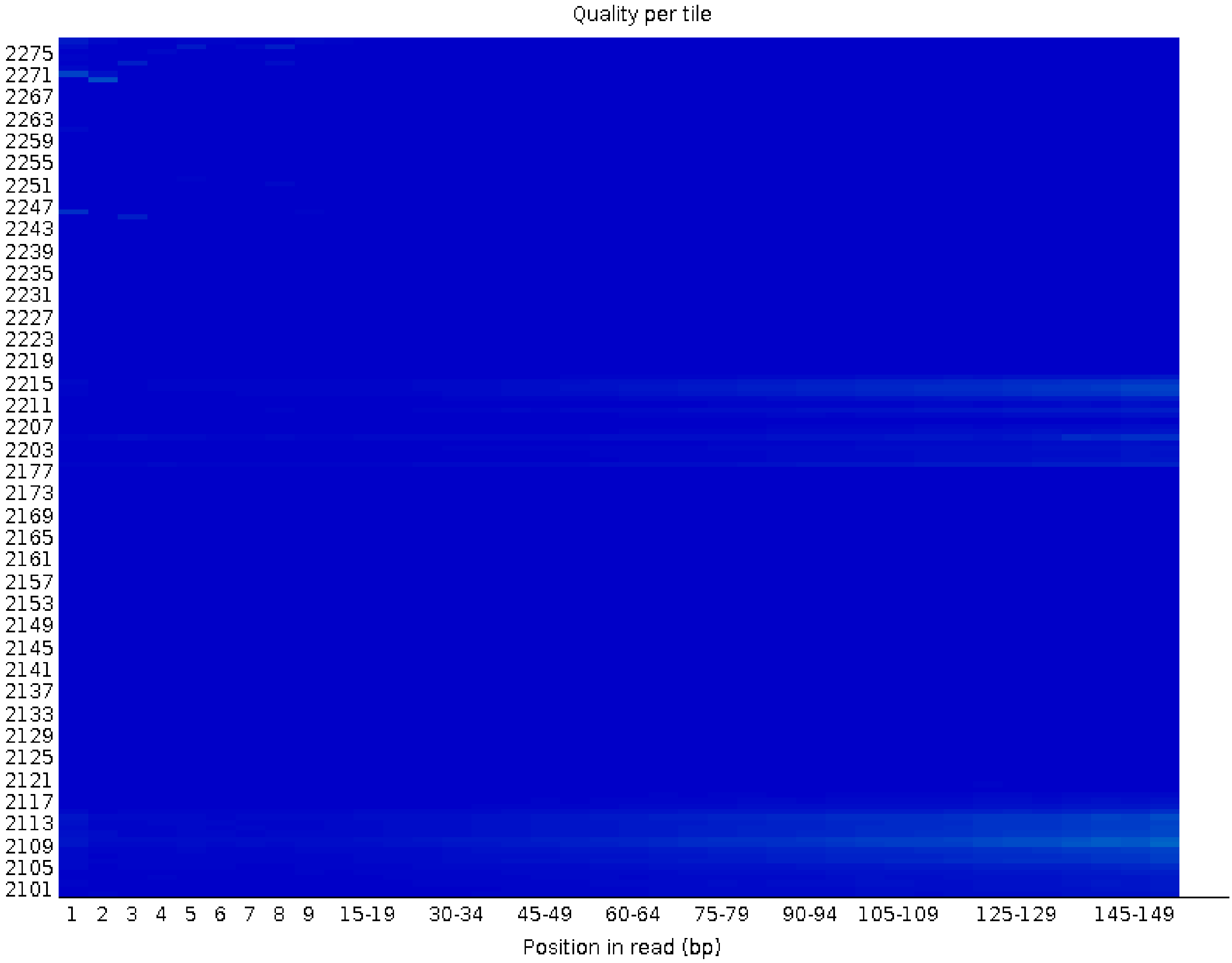

Per sequence quality scores

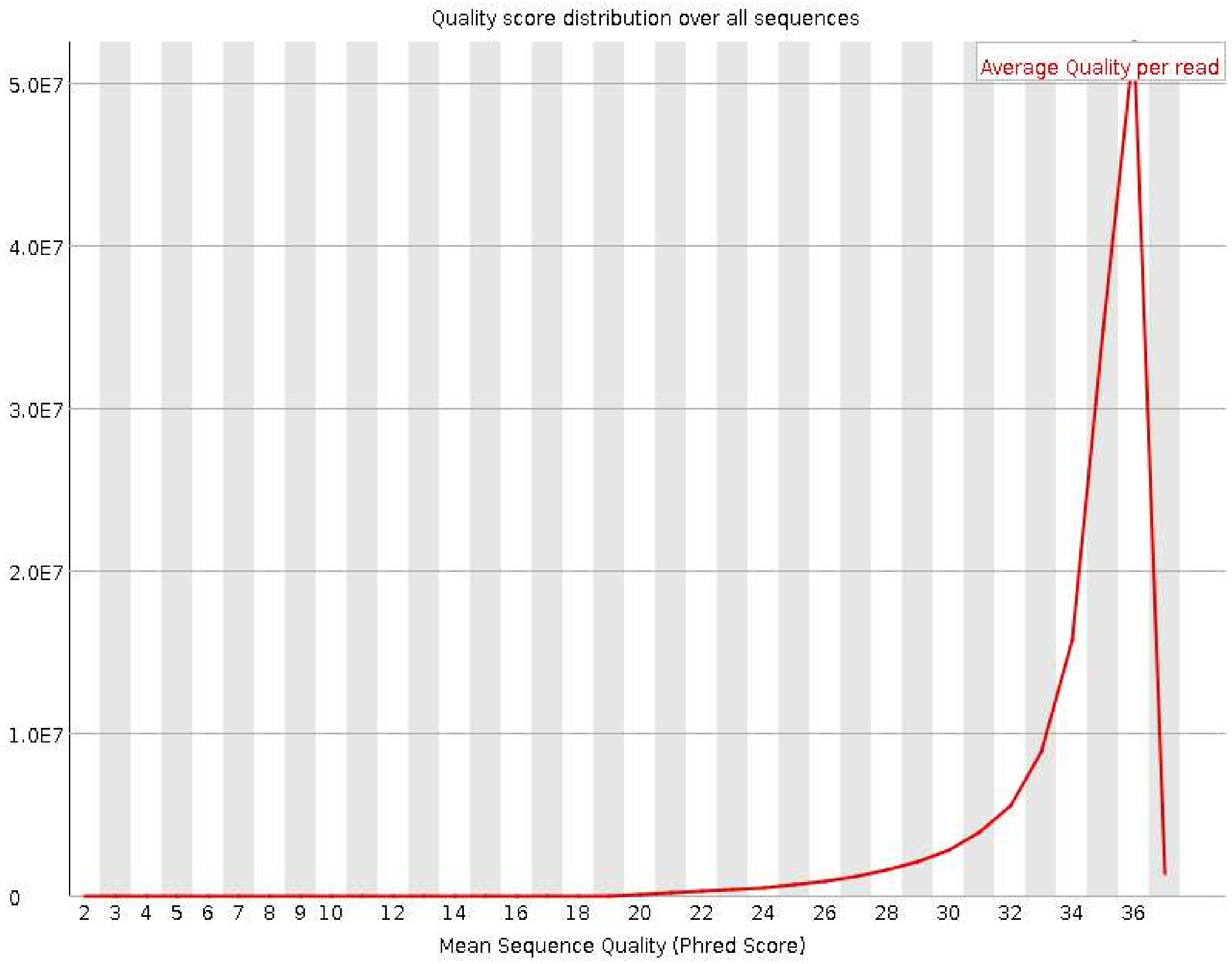

Per base sequence content

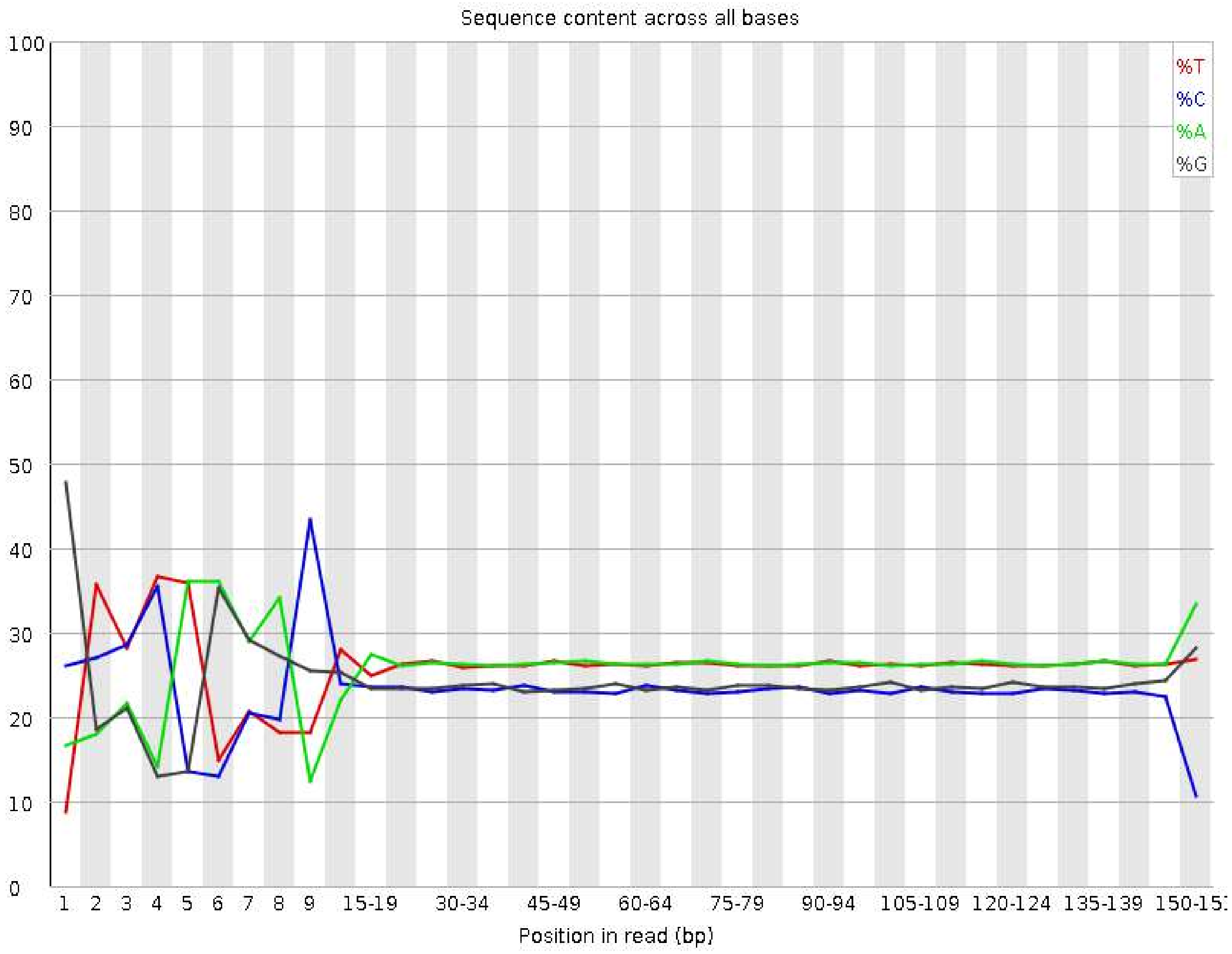

Per sequence GC content

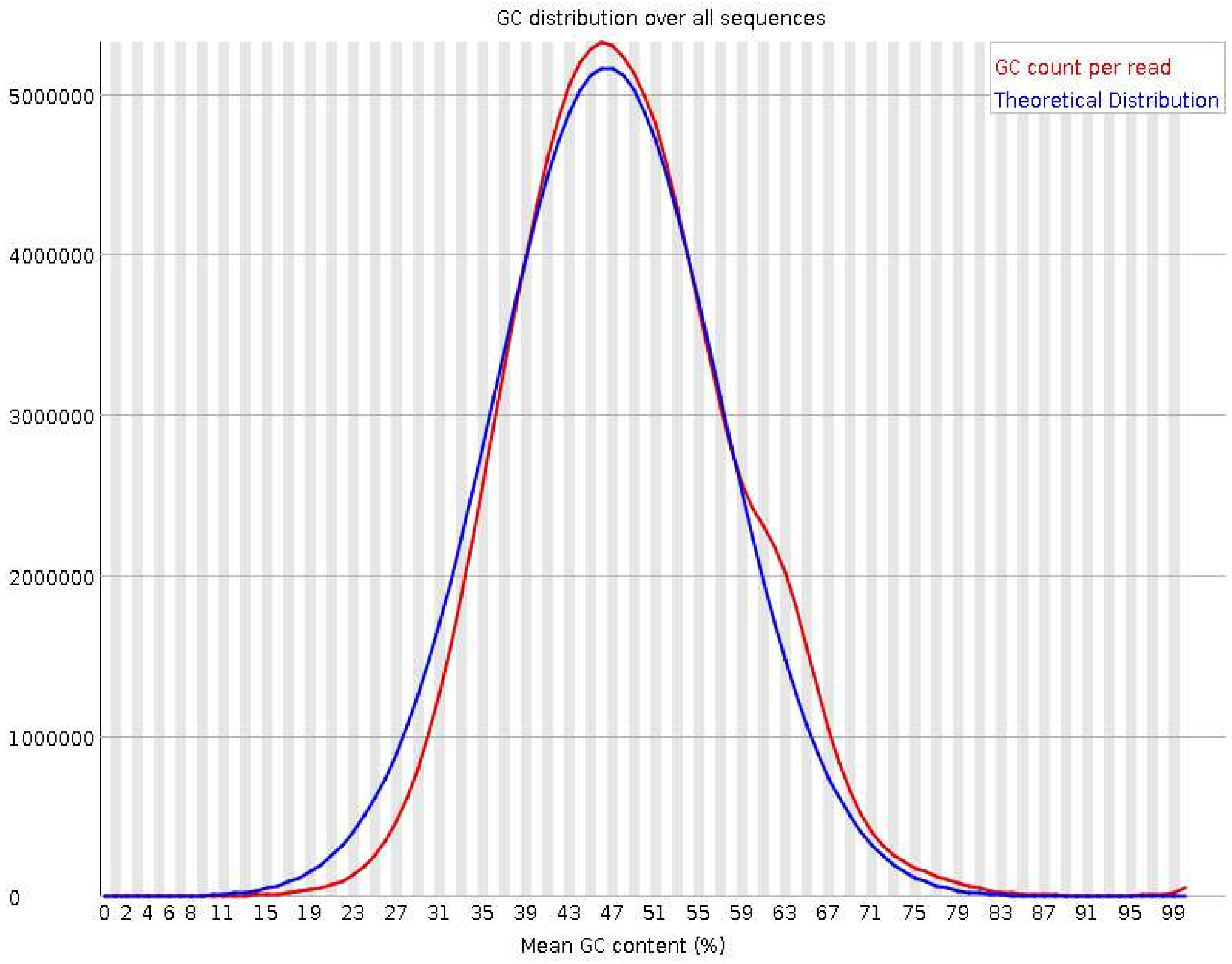

Per base N content

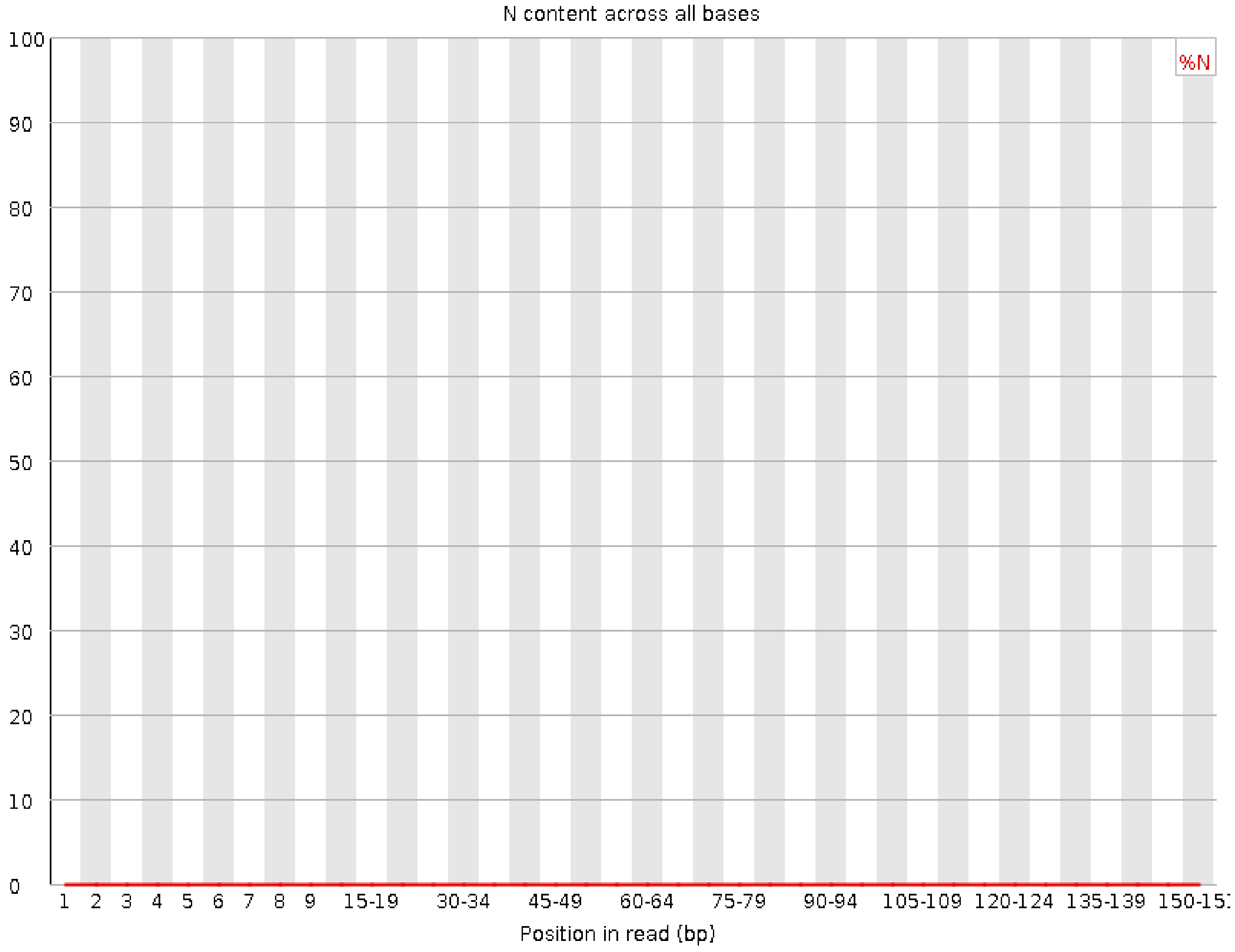

Sequence Length Distribution

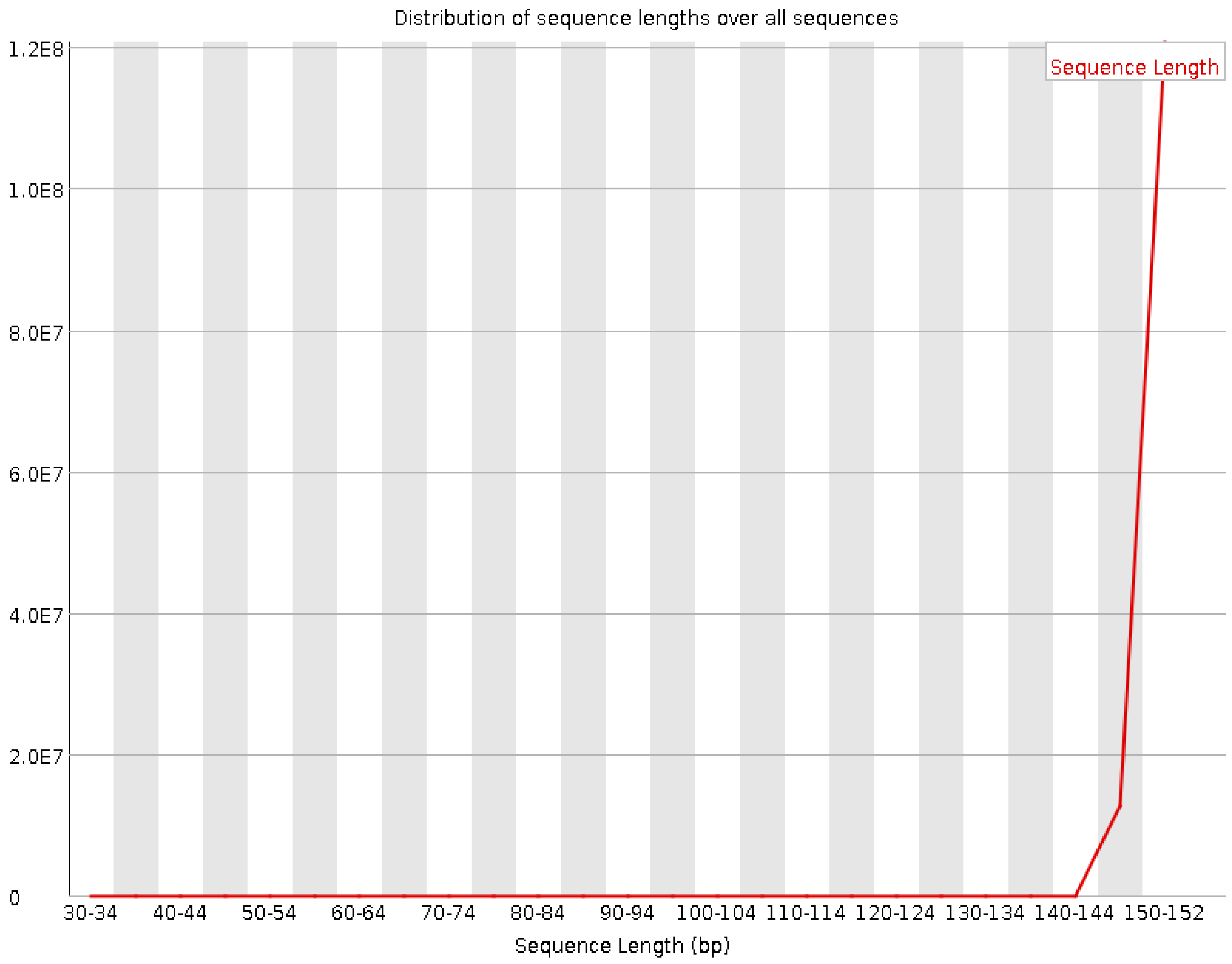

Sequence Duplication Levels

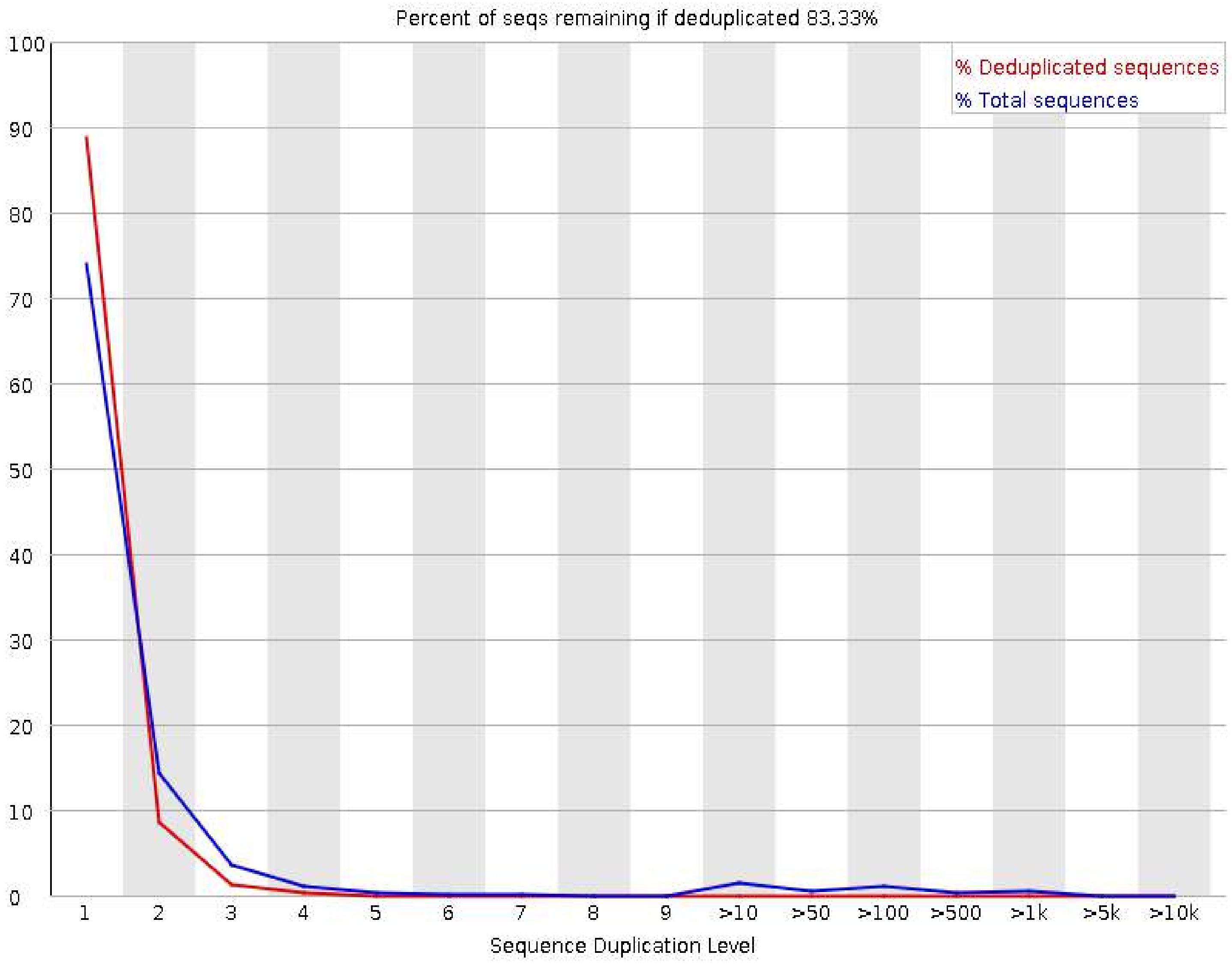

Overrepresented sequences

No overrepresented sequences

Adapter Content

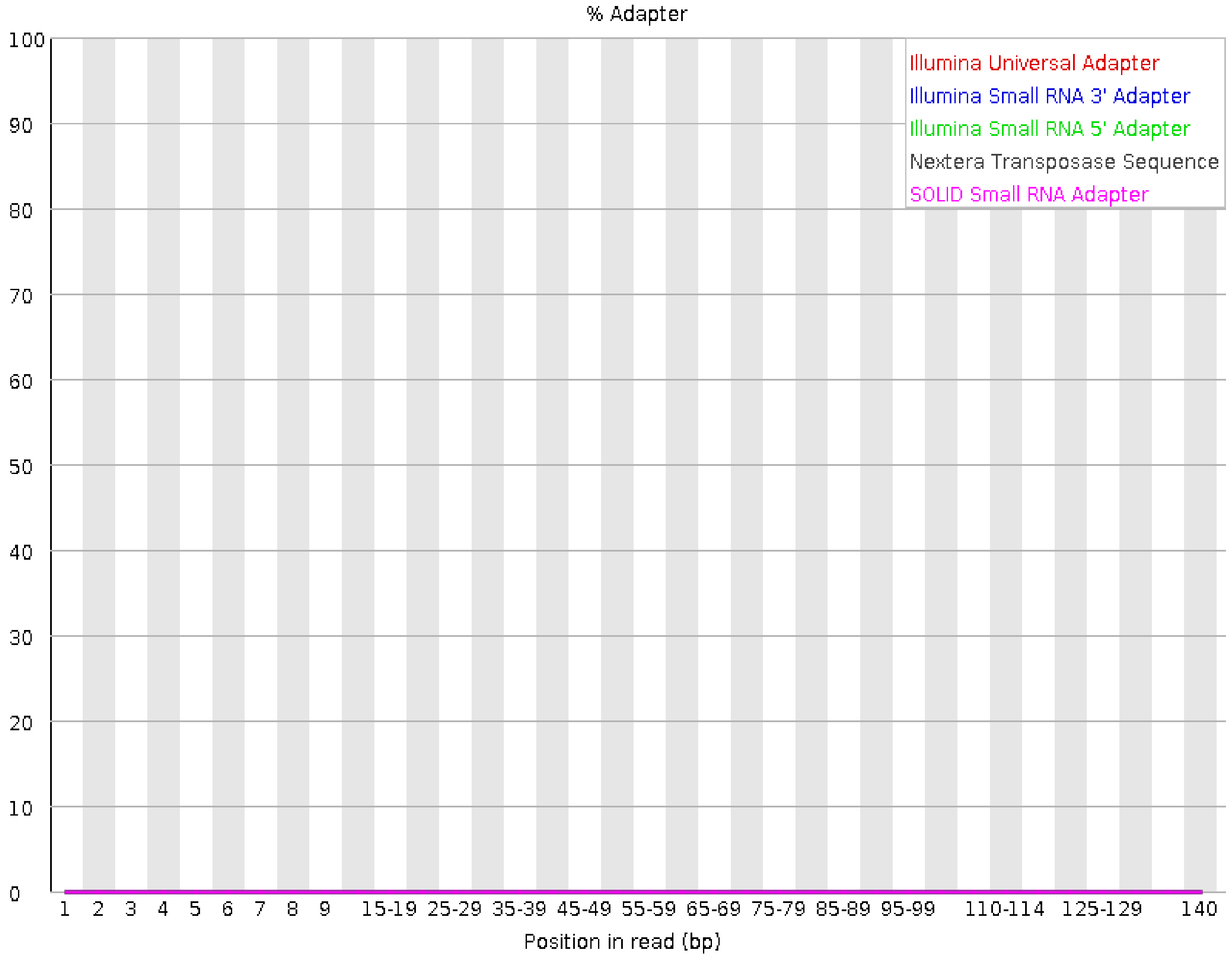

Produced by FastQC (version 0.11.8)

FastQC Report

Wed 9 Oct 2019
EHM_18_03_S5_R1_001.fastq.gz

Summary

- Basic Statistics
- Per base sequence quality
- Per tile sequence quality
- Per sequence quality scores
- Per base sequence content
- Per sequence GC content
- Per base N content
- Sequence Length Distribution
- Sequence Duplication Levels
- Overrepresented sequences
- Adapter Content

Basic Statistics

Measure	Value
Filename	EHM_18_03_S5_R1_001.fastq.gz
File type	Conventional base calls
Encoding	Sanger / Illumina 1.9
Total Sequences	131423503
Sequences flagged as poor quality	0
Sequence length	35-151
%GC	47

Per base sequence quality

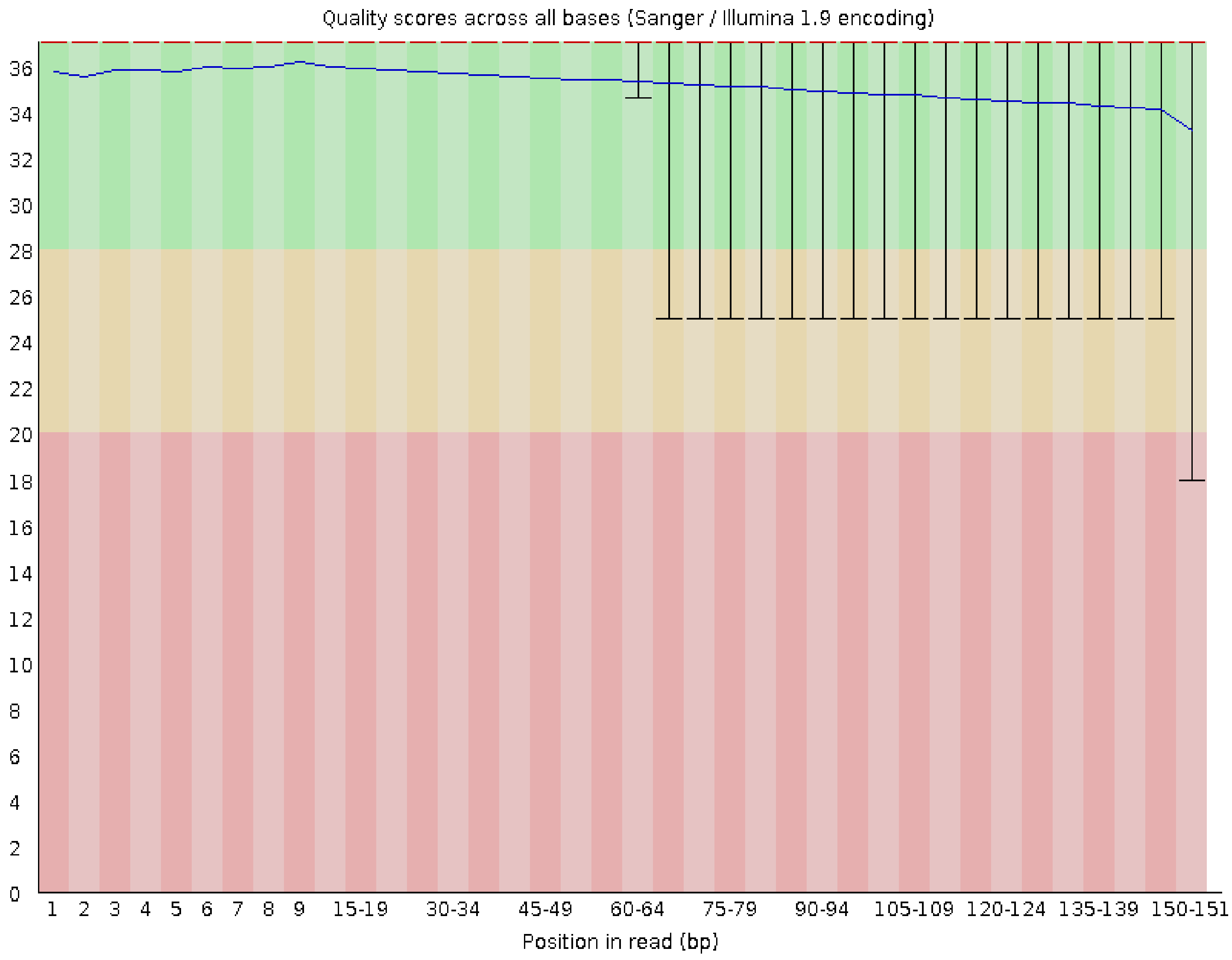

Per tile sequence quality

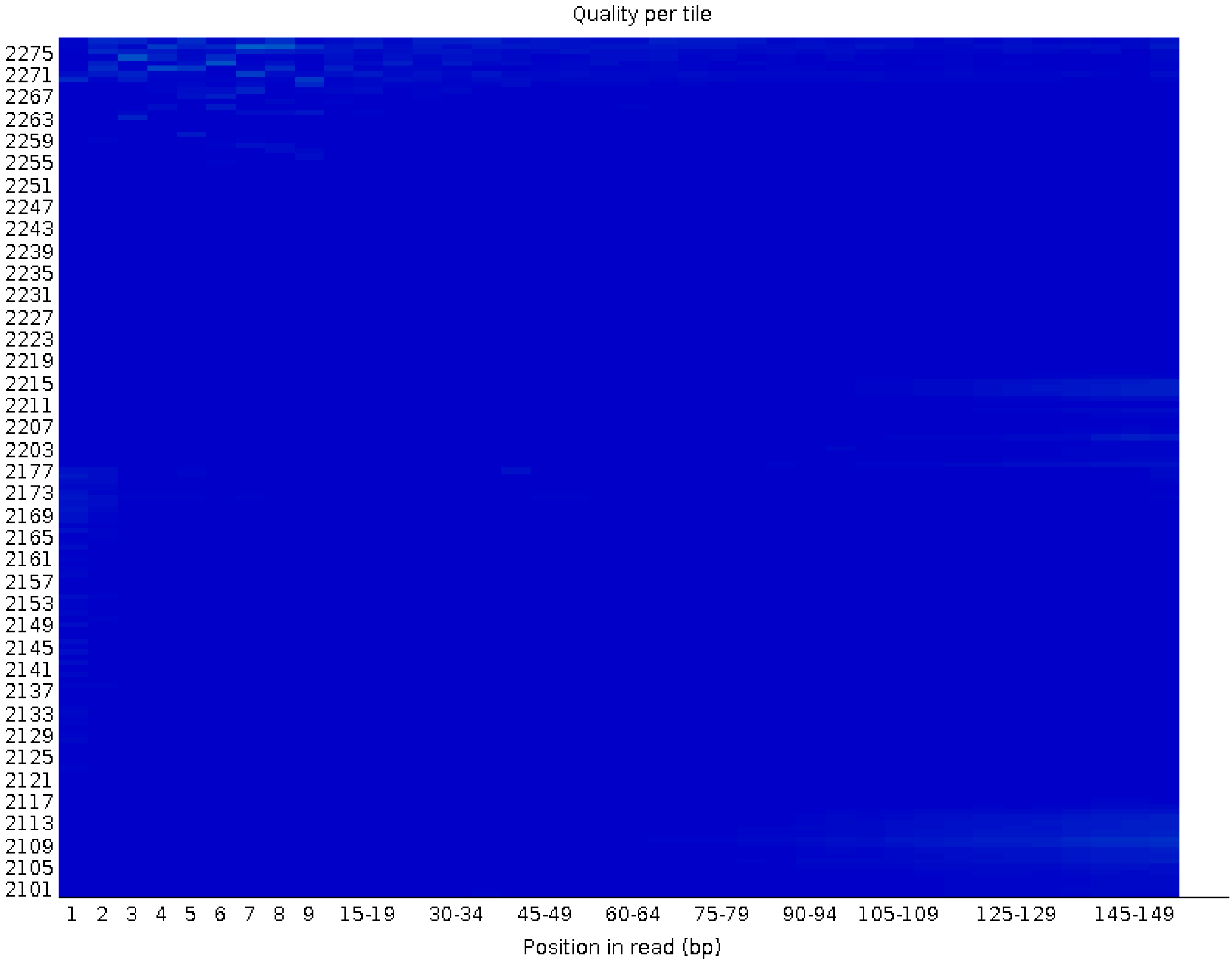

Per sequence quality scores

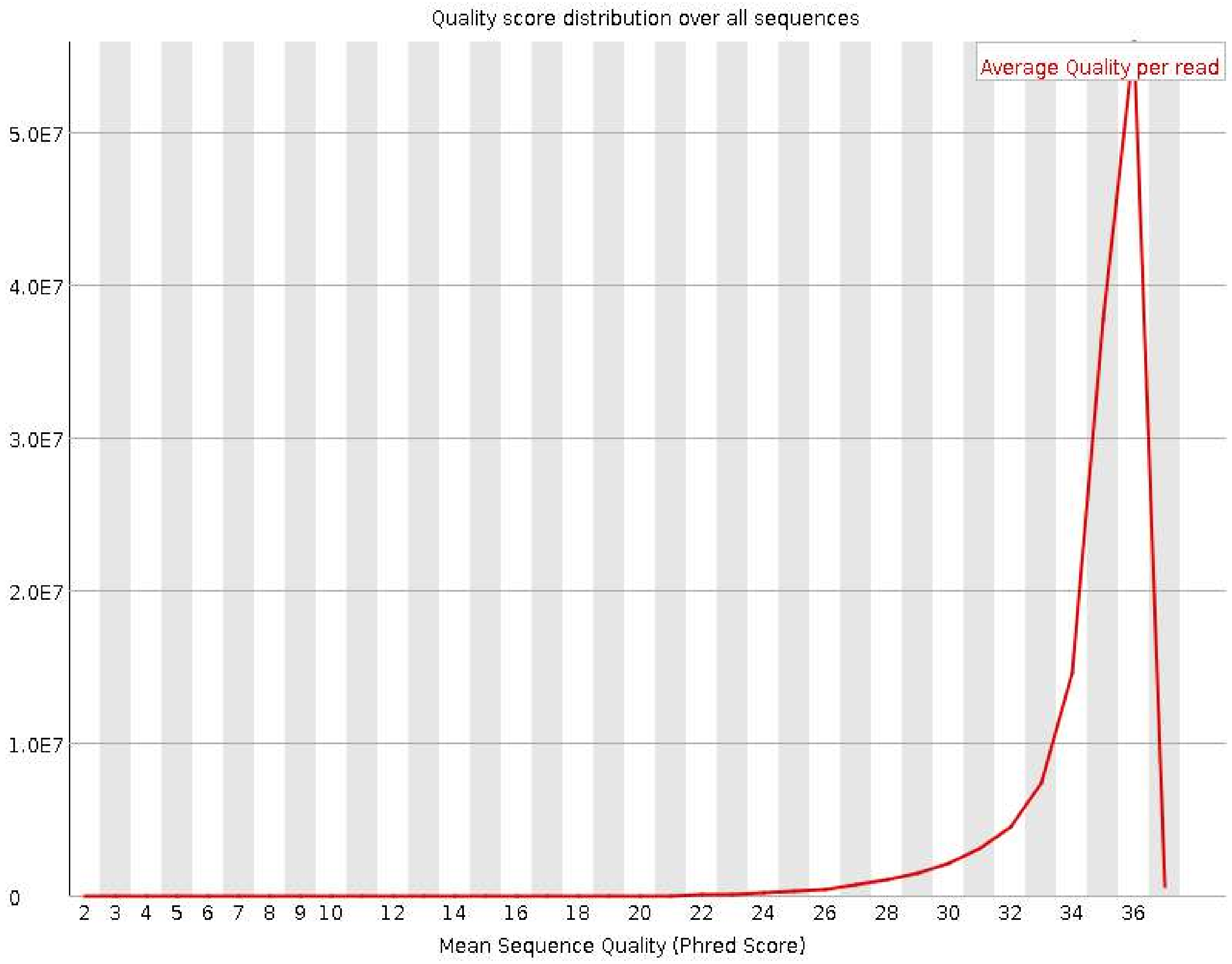

Per base sequence content

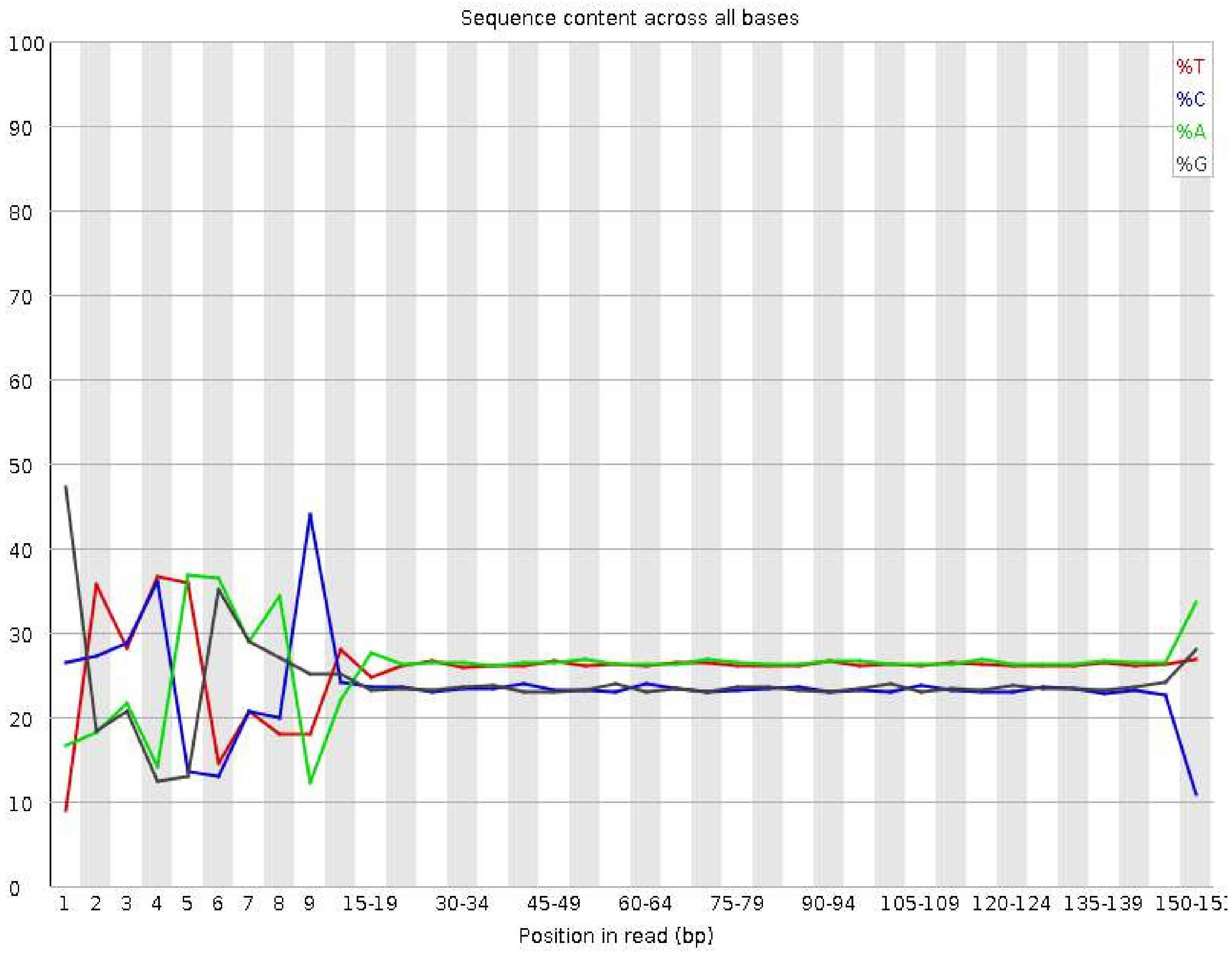

Per sequence GC content

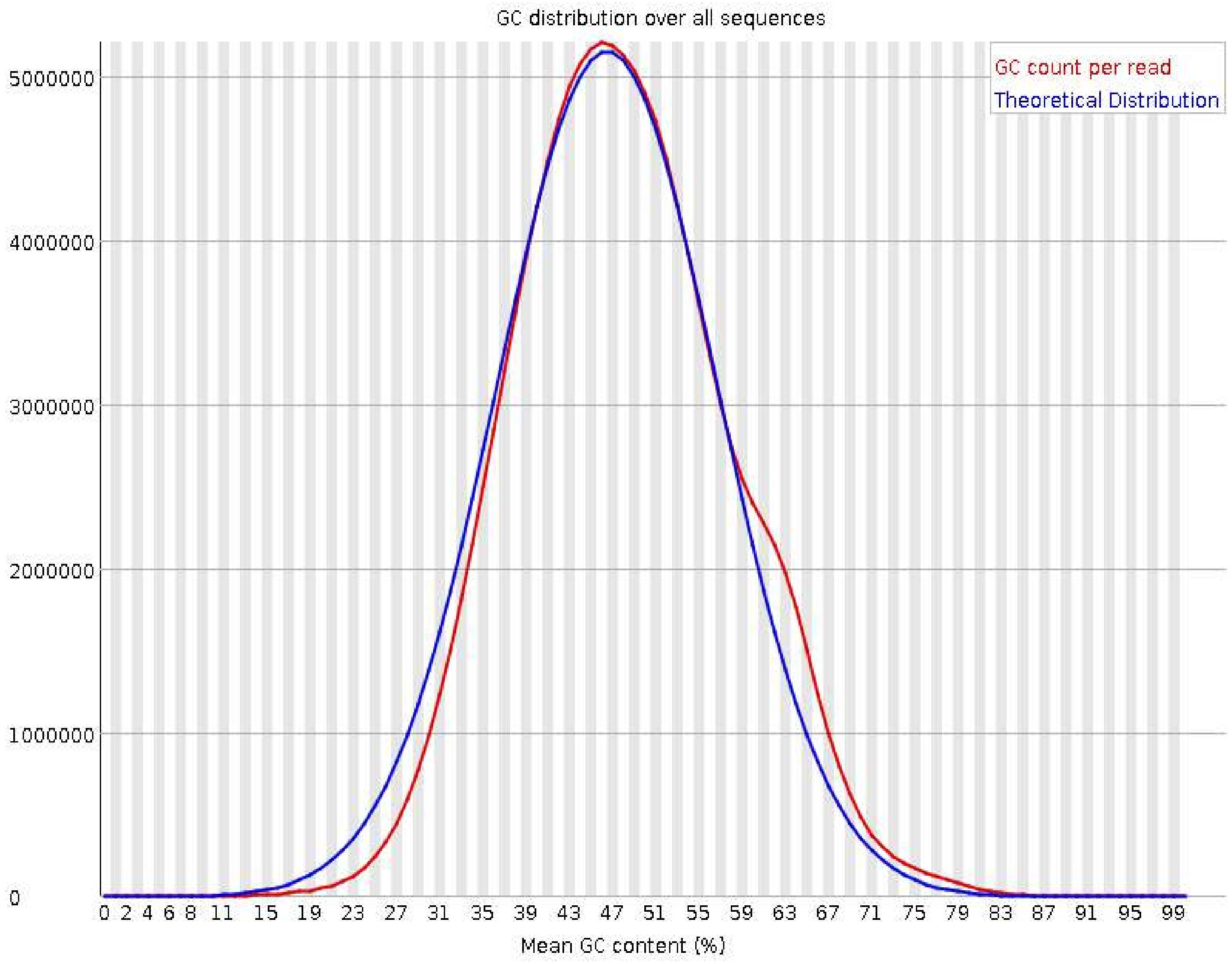

Per base N content

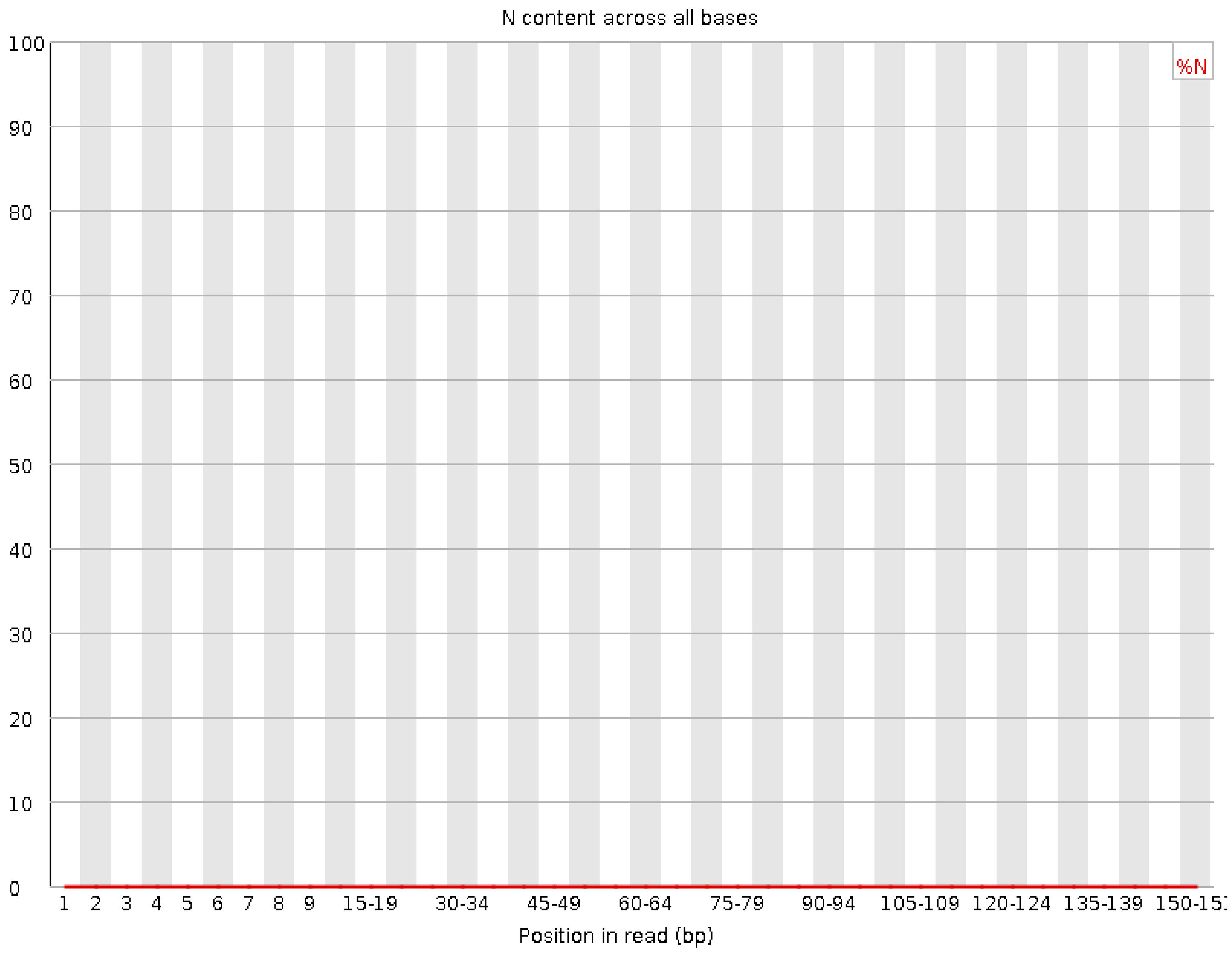

Sequence Length Distribution

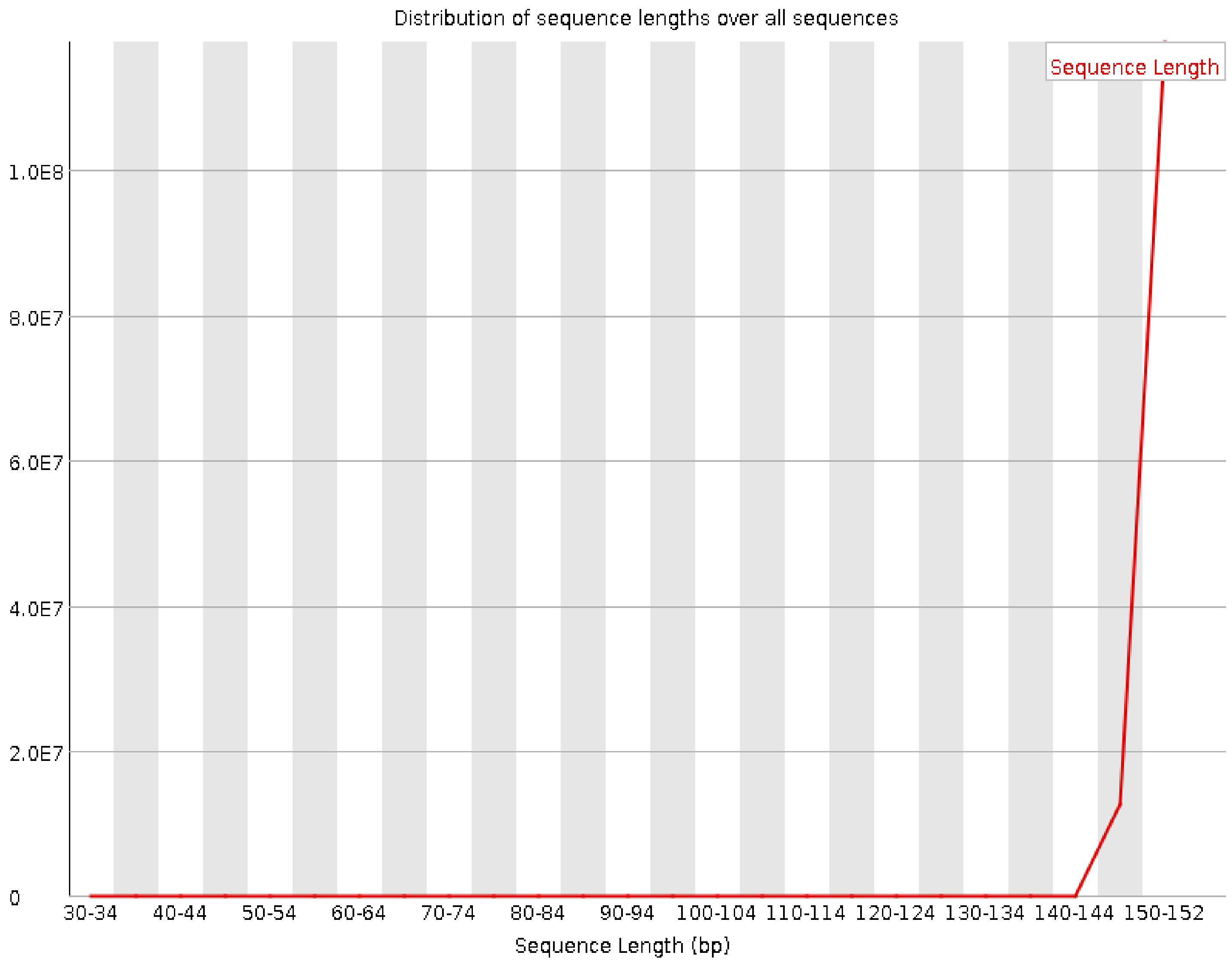

Sequence Duplication Levels

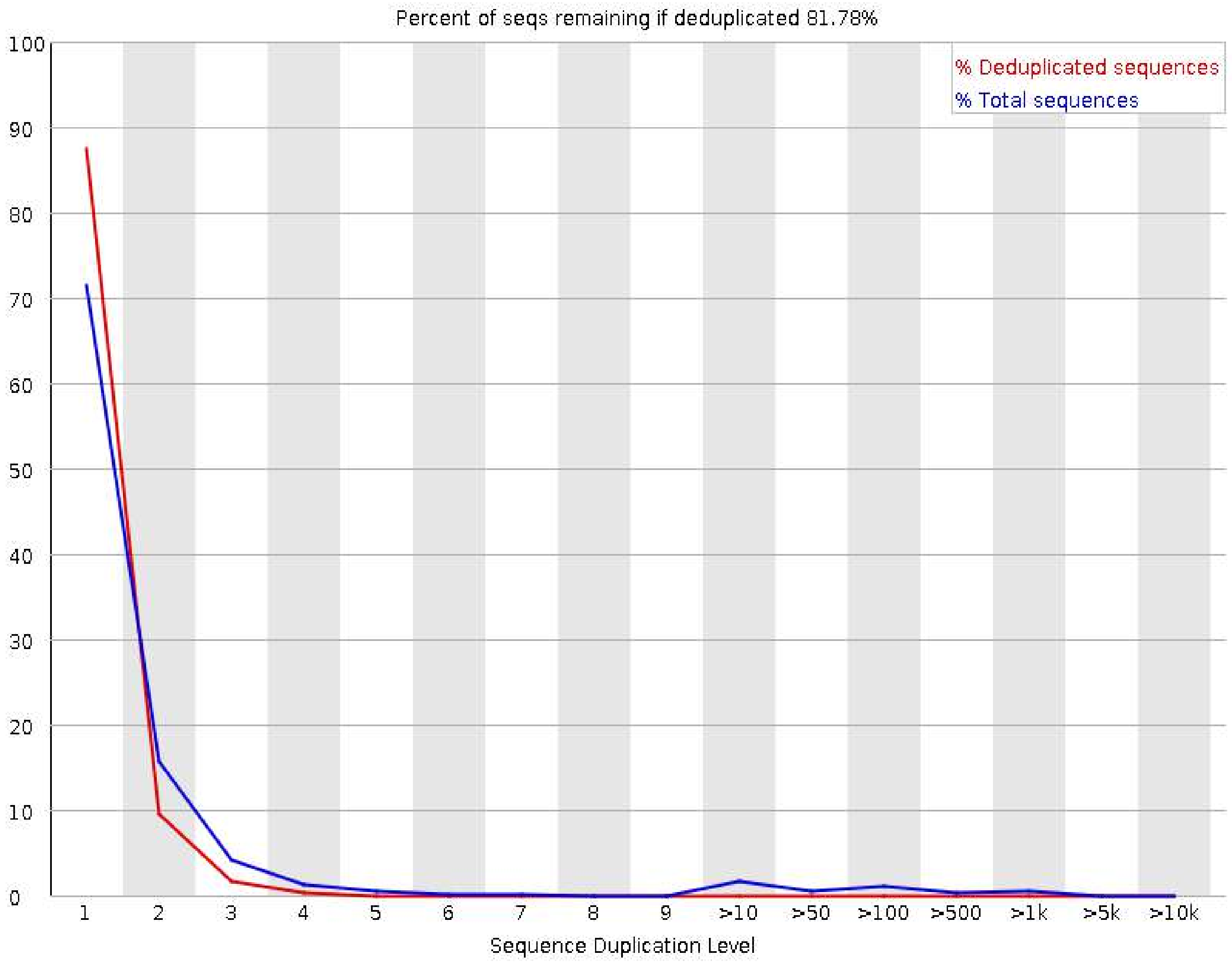

Overrepresented sequences

No overrepresented sequences

Adapter Content

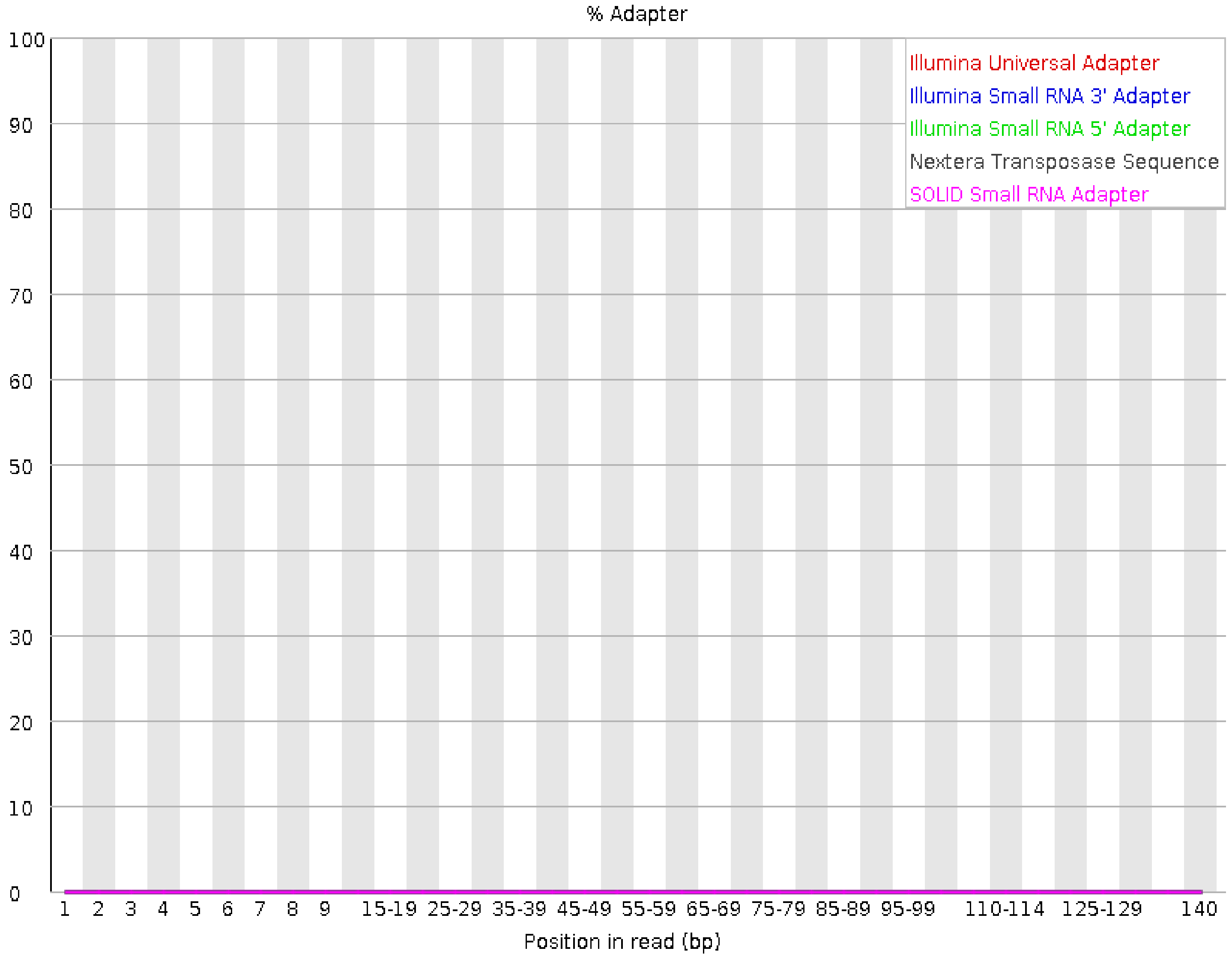

Produced by FastQC (version 0.11.8)

FastQC Report

Wed 9 Oct 2019
EHM_18_03_S5_R2_001.fastq.gz

Summary

- Basic Statistics
- Per base sequence quality
- Per tile sequence quality
- Per sequence quality scores
- Per base sequence content
- Per sequence GC content
- Per base N content
- Sequence Length Distribution
- Sequence Duplication Levels
- Overrepresented sequences
- Adapter Content

Basic Statistics

Measure	Value
Filename	EHM_18_03_S5_R2_001.fastq.gz
File type	Conventional base calls
Encoding	Sanger / Illumina 1.9
Total Sequences	131423503
Sequences flagged as poor quality	0
Sequence length	35-151
%GC	47

Per base sequence quality

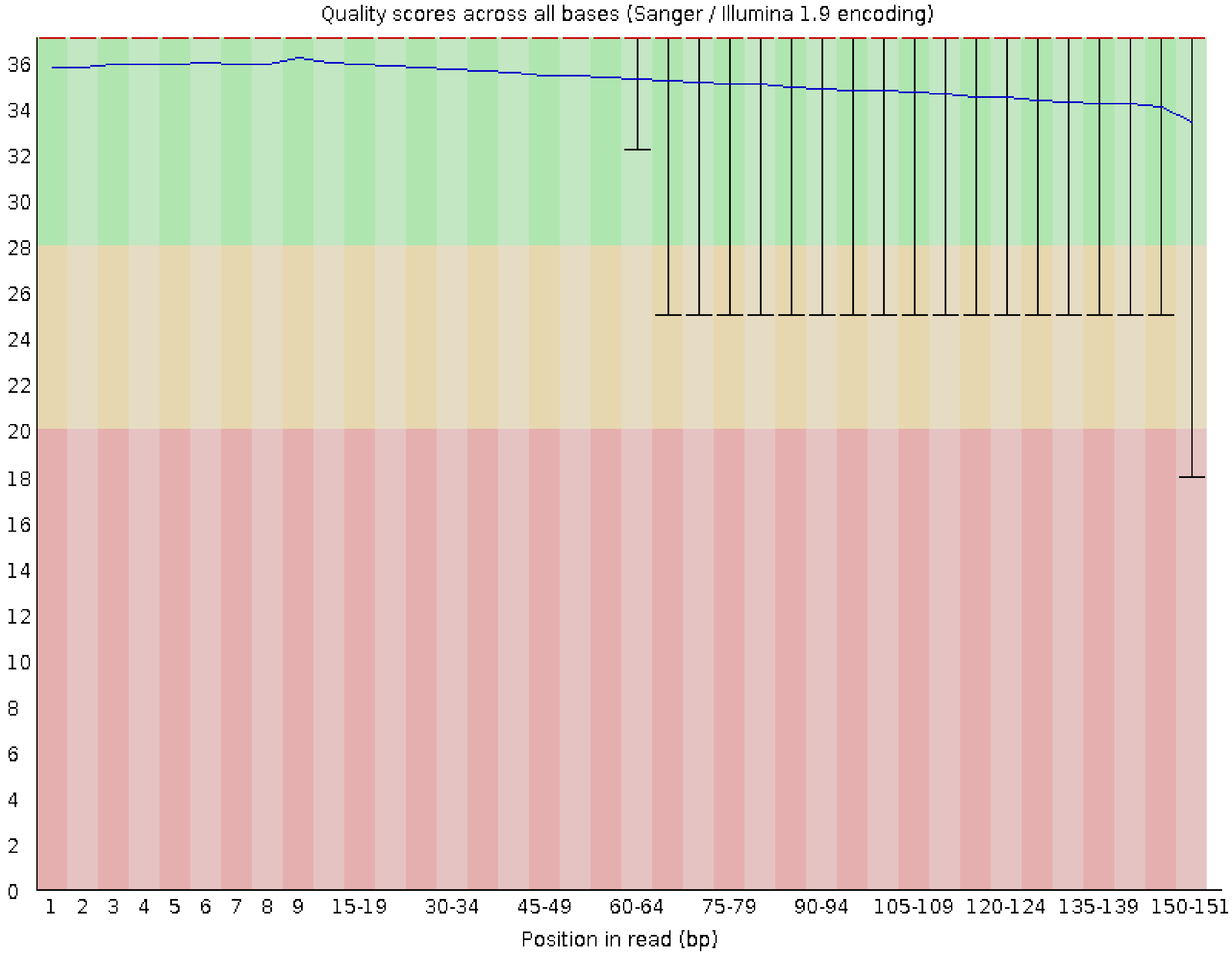

Per tile sequence quality

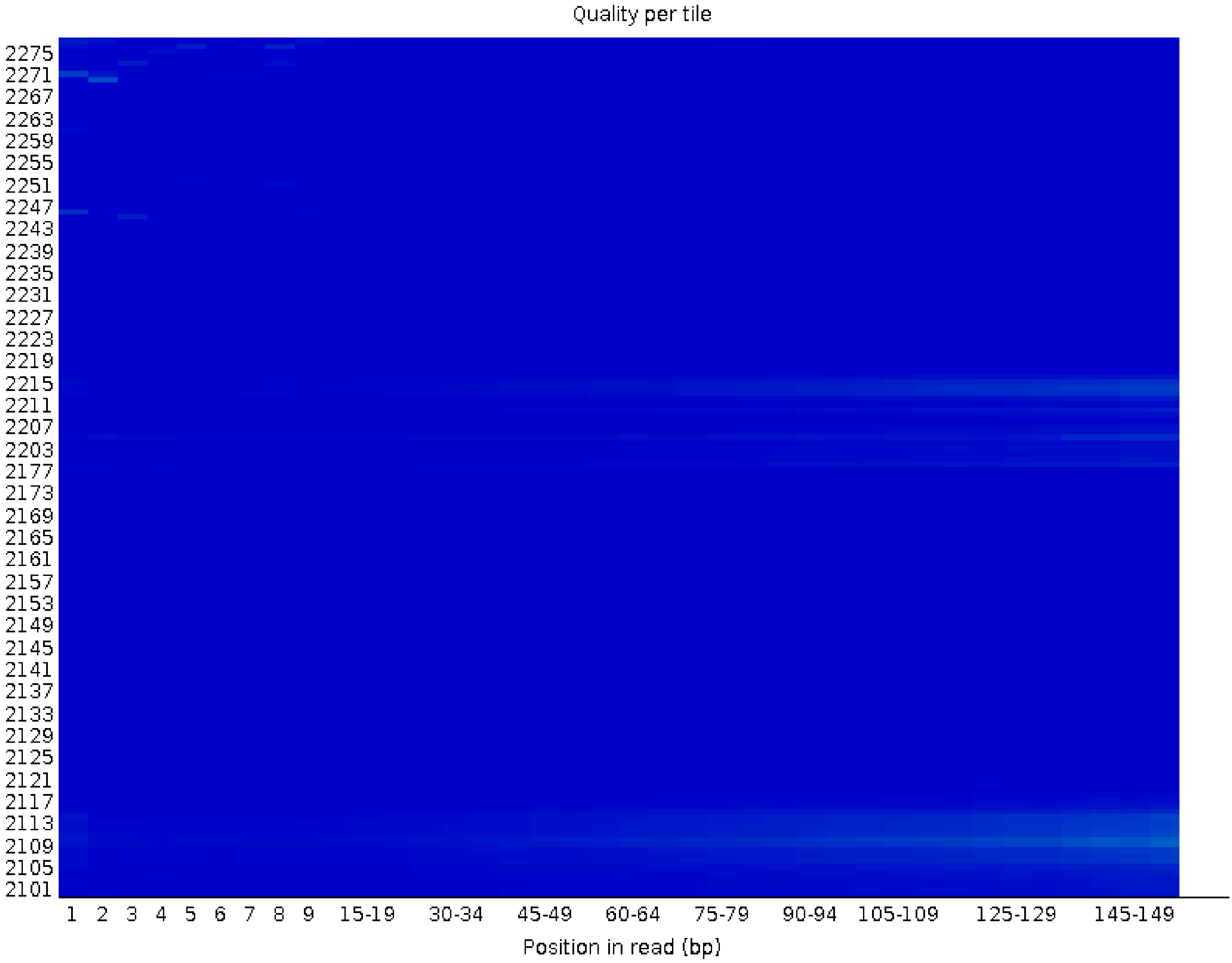

Per sequence quality scores

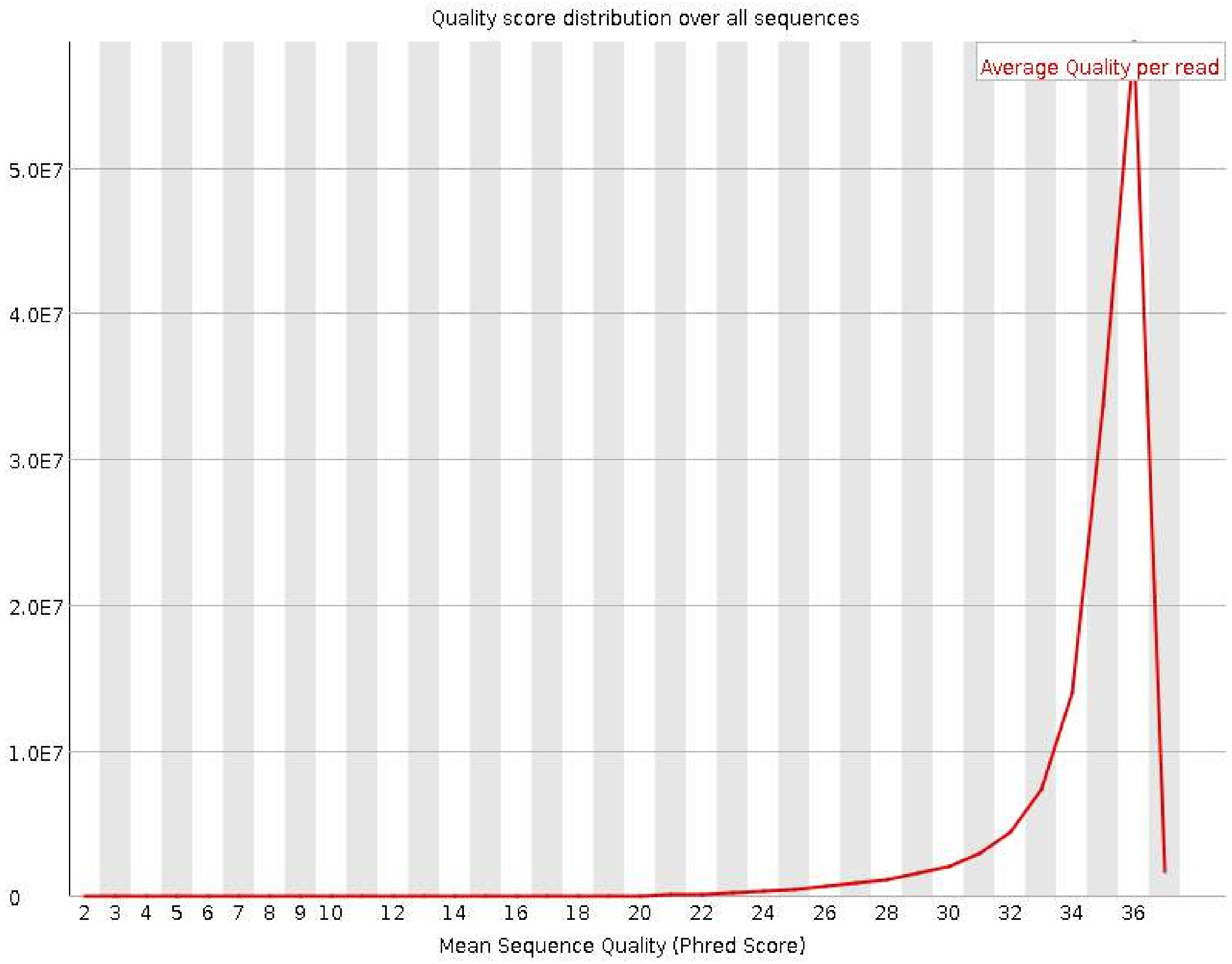

Per base sequence content

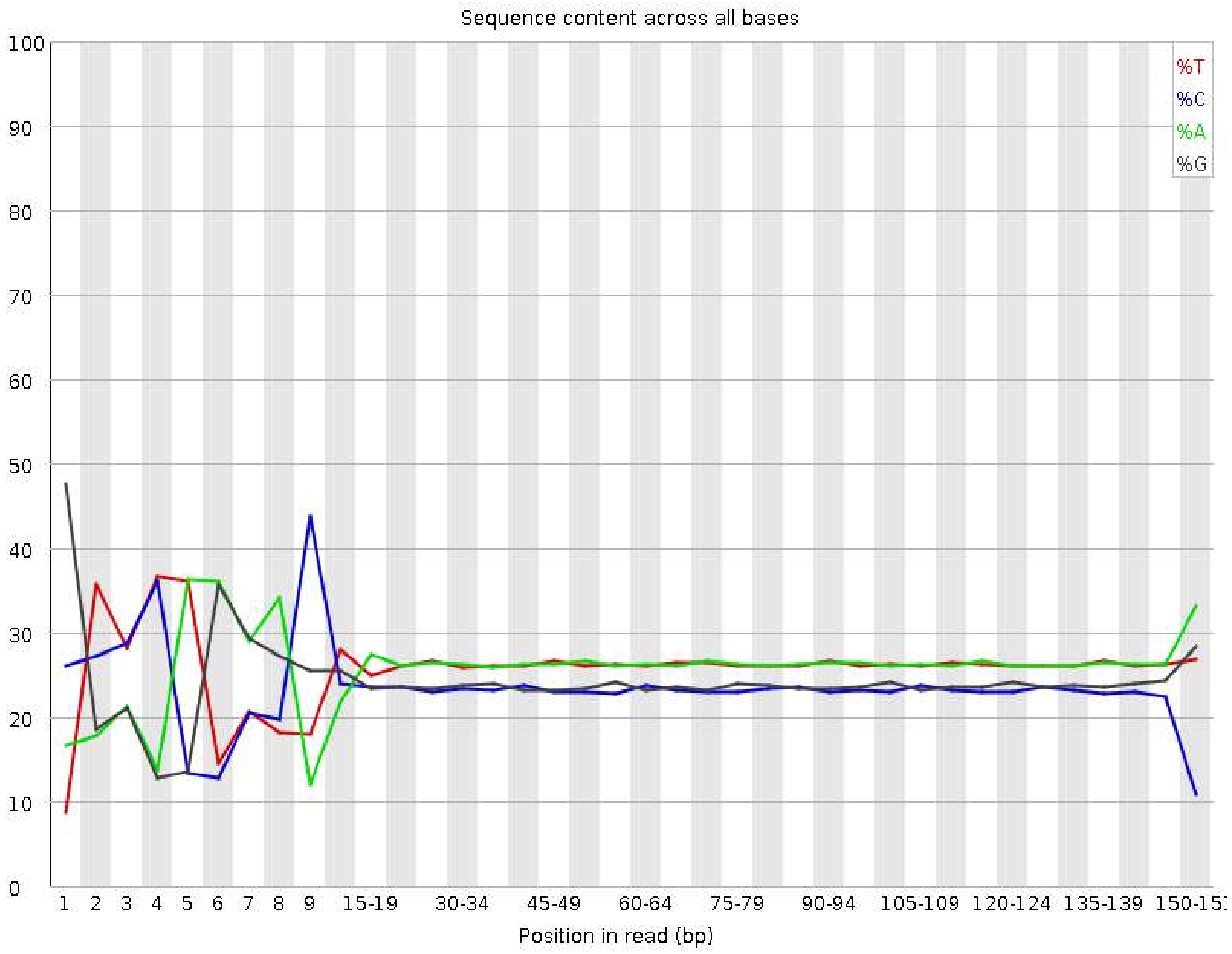

Per sequence GC content

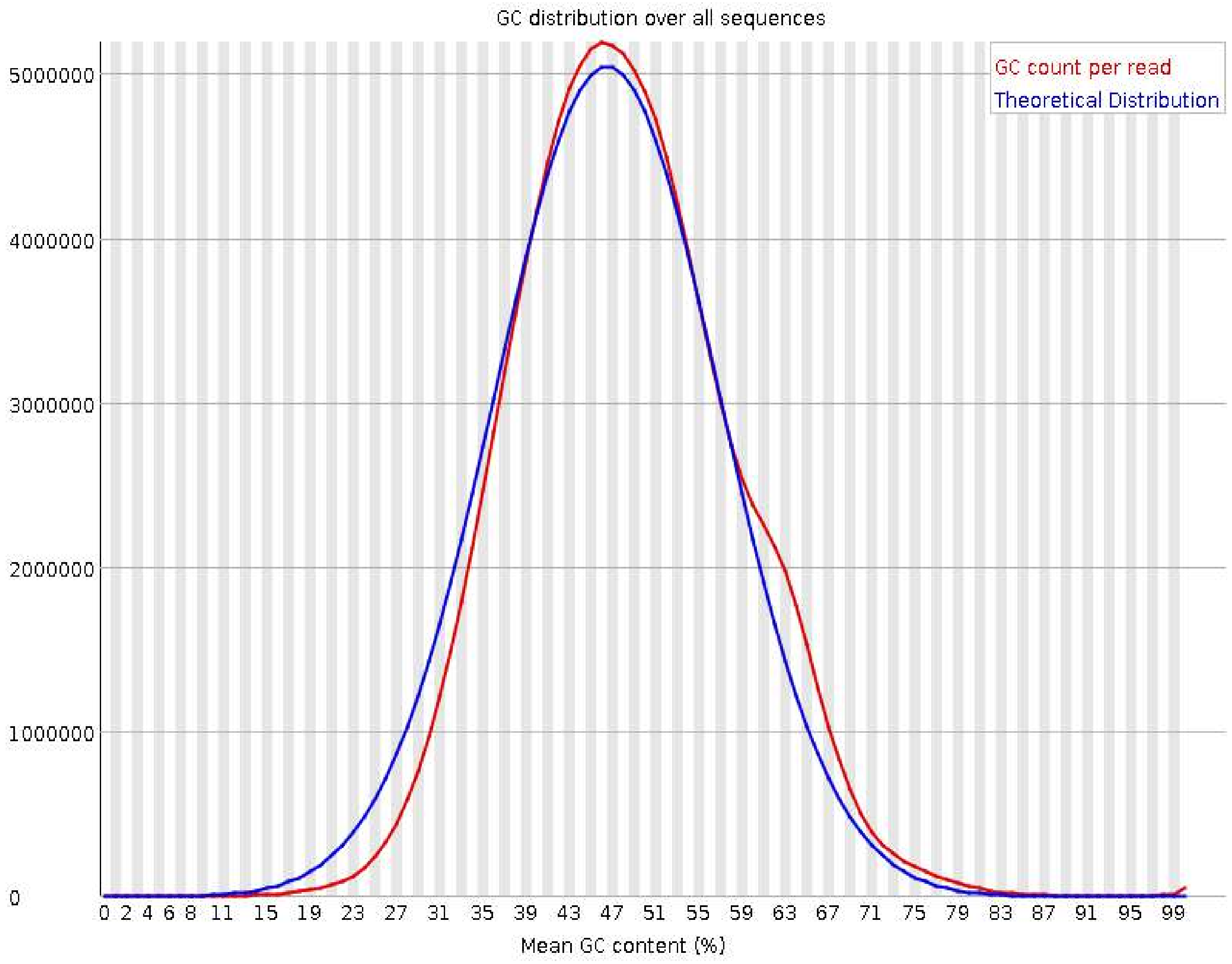

Per base N content

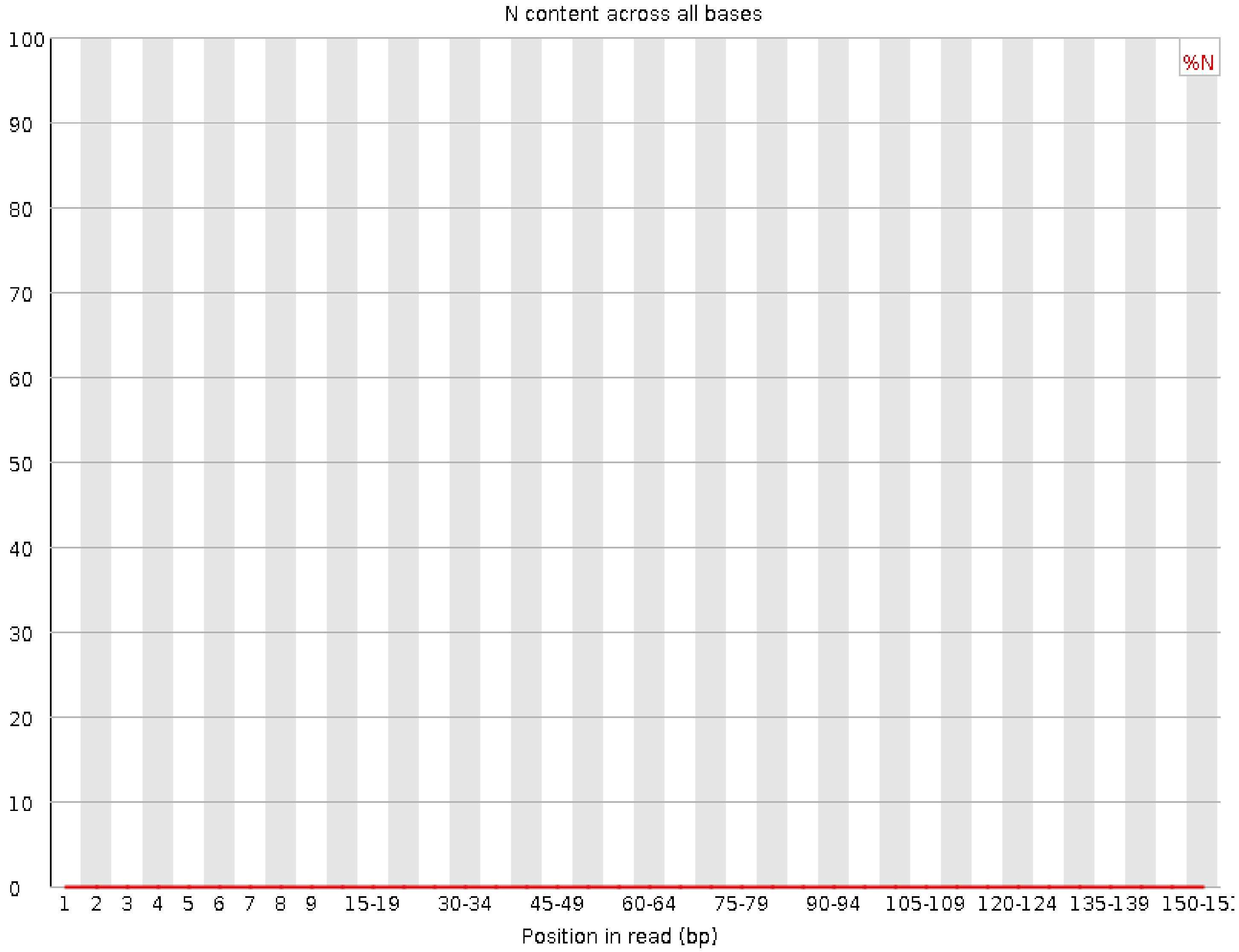

Sequence Length Distribution

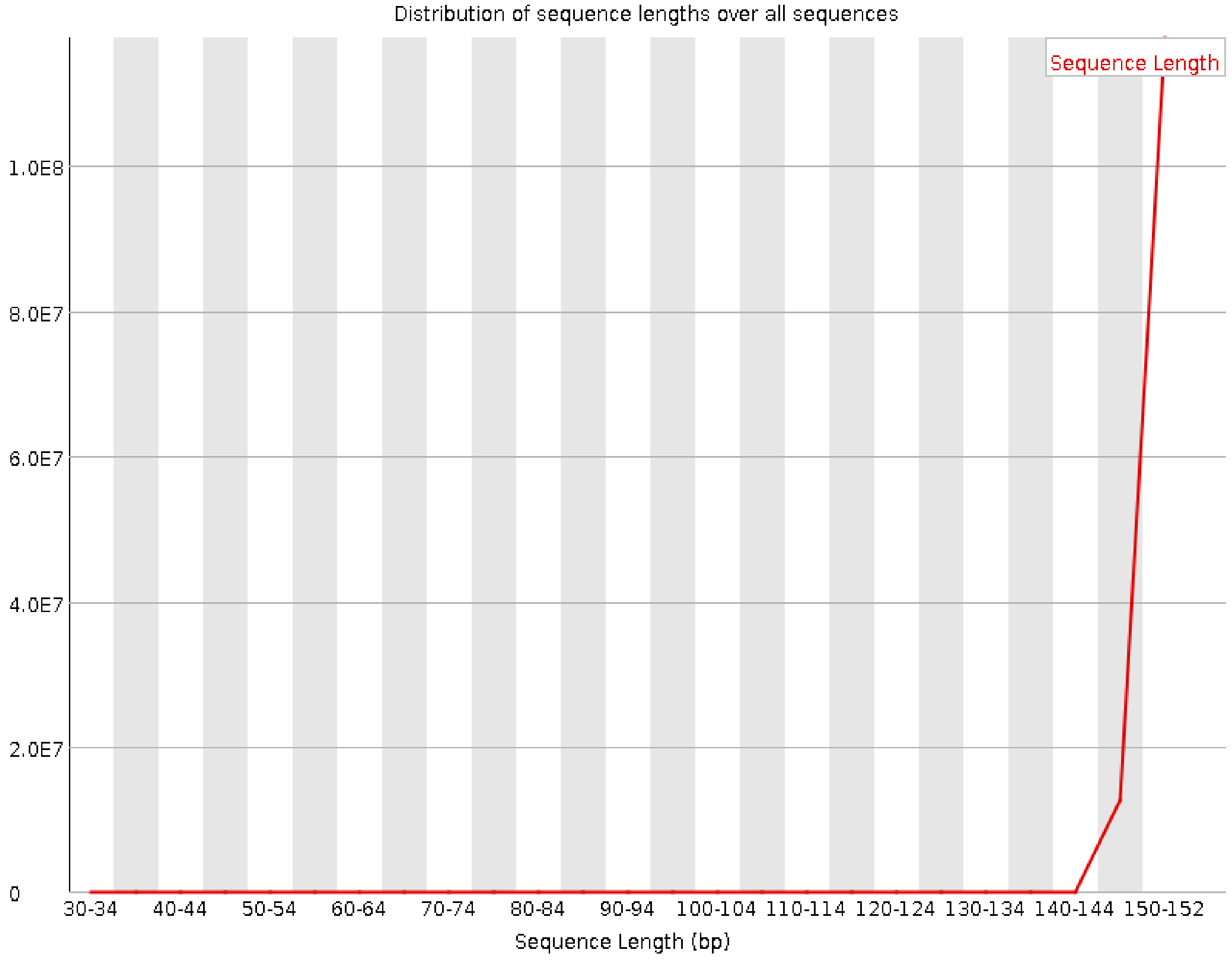

Sequence Duplication Levels

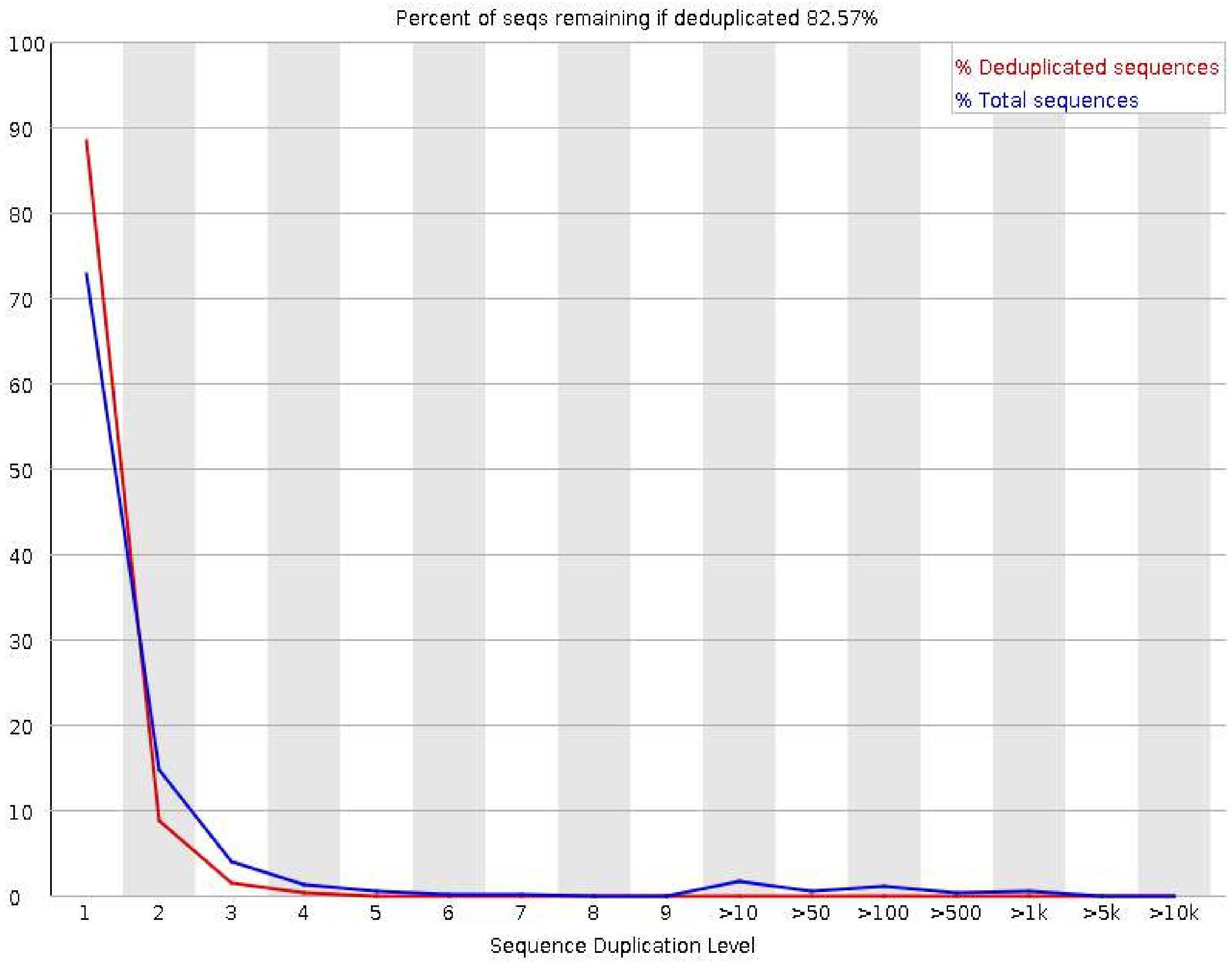

Overrepresented sequences

No overrepresented sequences

Adapter Content

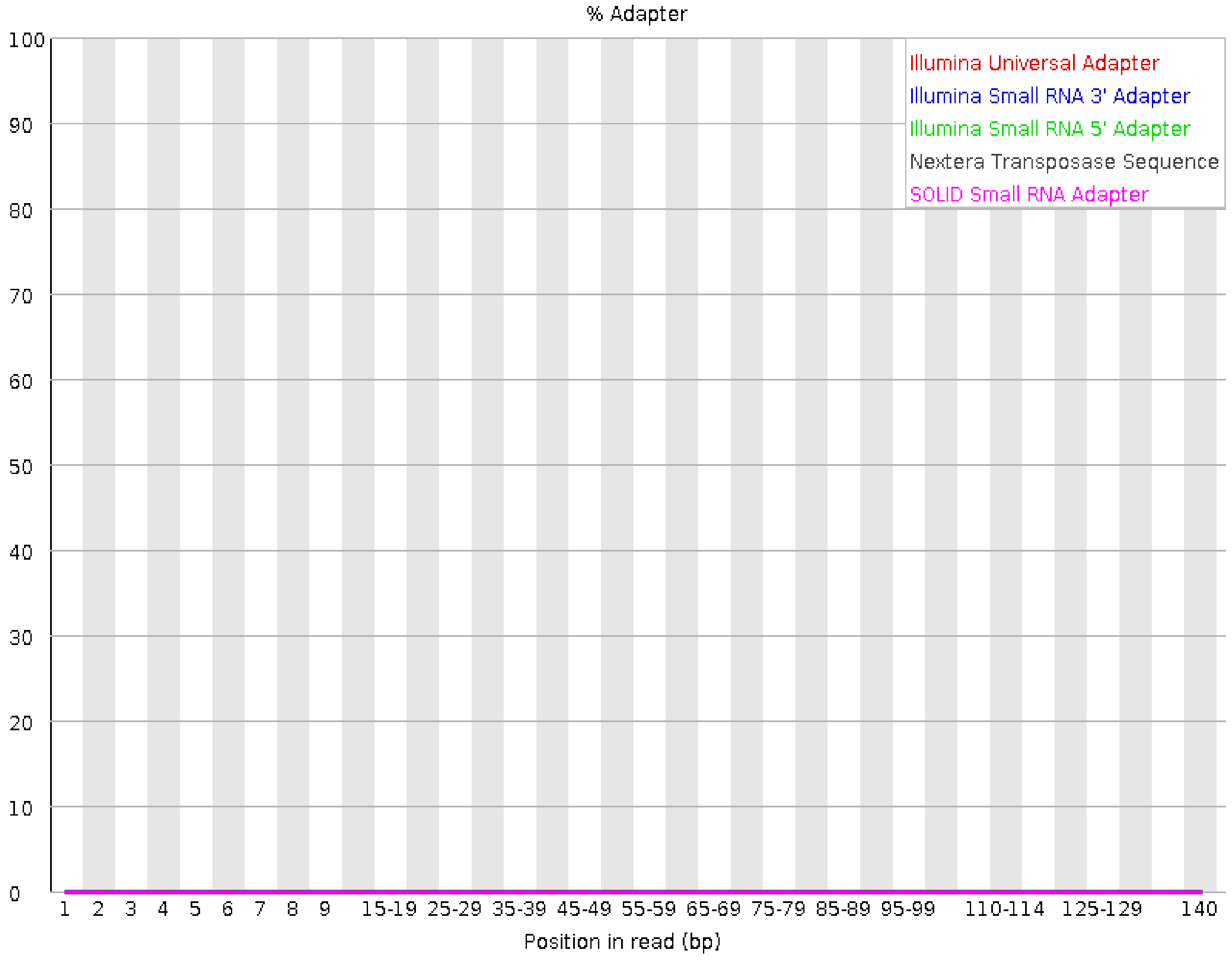

Produced by FastQC (version 0.11.8)

FastQC Report

Wed 9 Oct 2019
Eulamprus.Female.Brain_R1.fastq.gz

Summary

- Basic Statistics
- Per base sequence quality
- Per tile sequence quality
- Per sequence quality scores
- Per base sequence content
- Per sequence GC content
- Per base N content
- Sequence Length Distribution
- Sequence Duplication Levels
- Overrepresented sequences
- Adapter Content

Basic Statistics

Measure	Value
Filename	Eulamprus.Female.Brain_R1.fastq.gz
File type	Conventional base calls
Encoding	Sanger / Illumina 1.9
Total Sequences	46491070
Sequences flagged as poor quality	0
Sequence length	125
%GC	46

Per base sequence quality

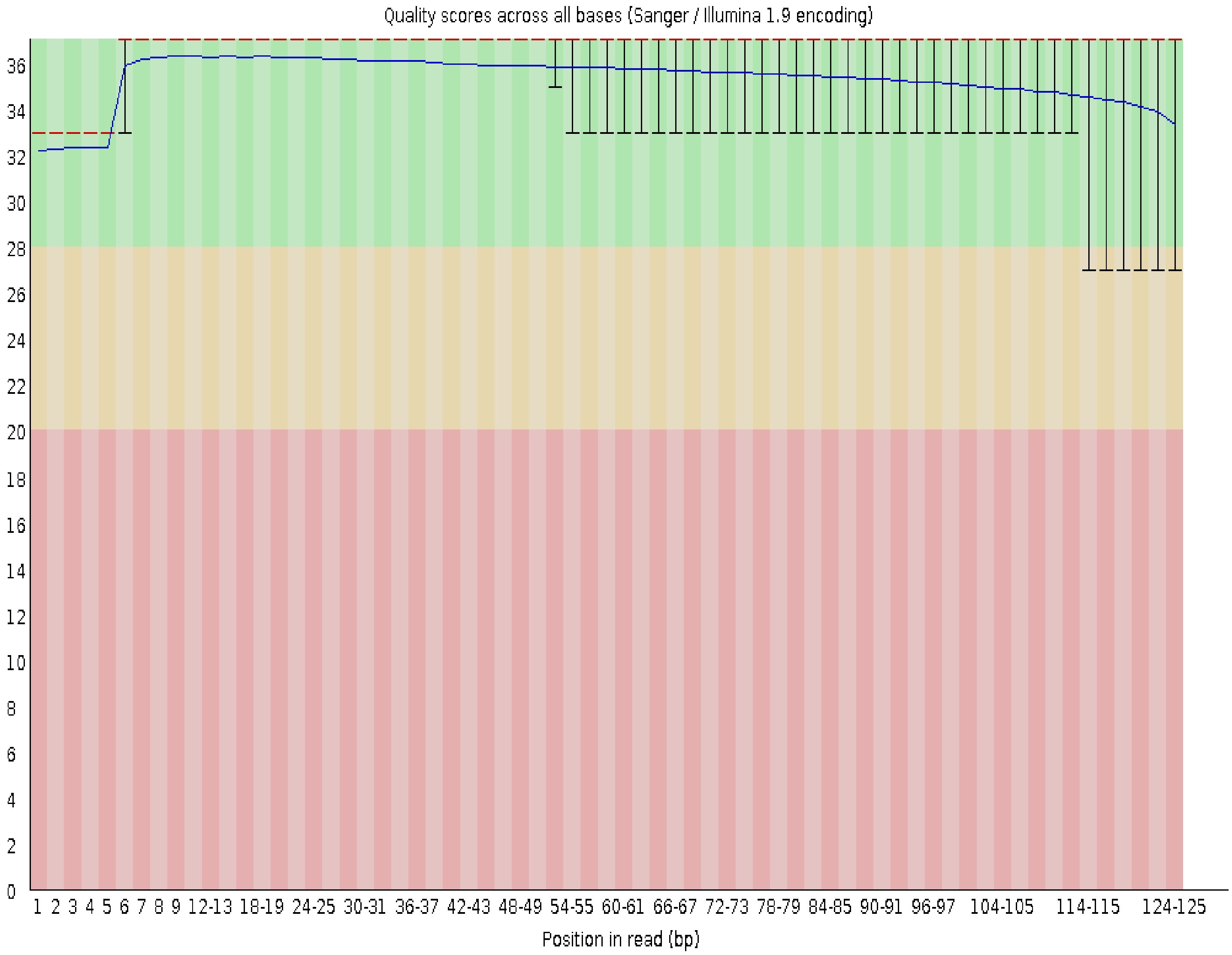

Per tile sequence quality

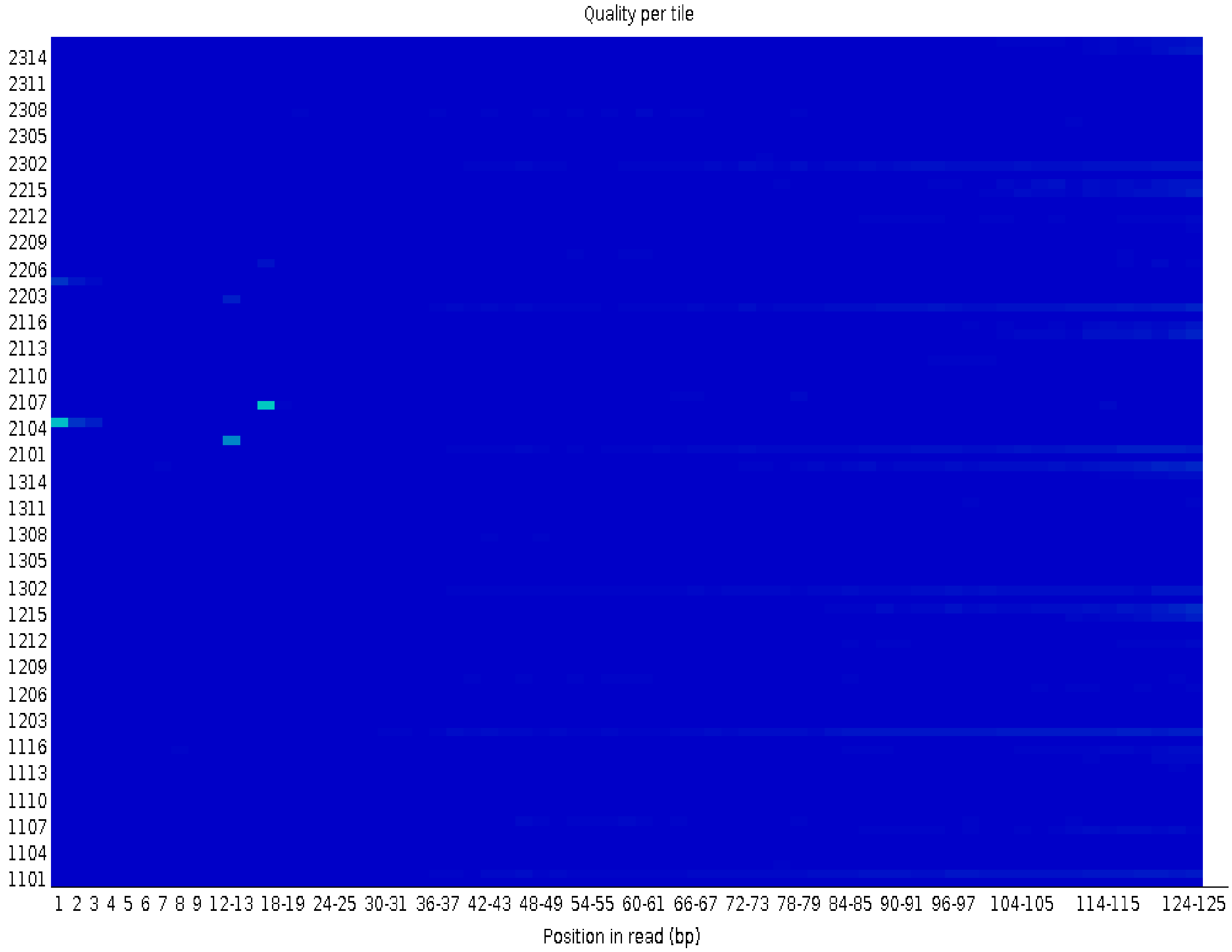

Per sequence quality scores

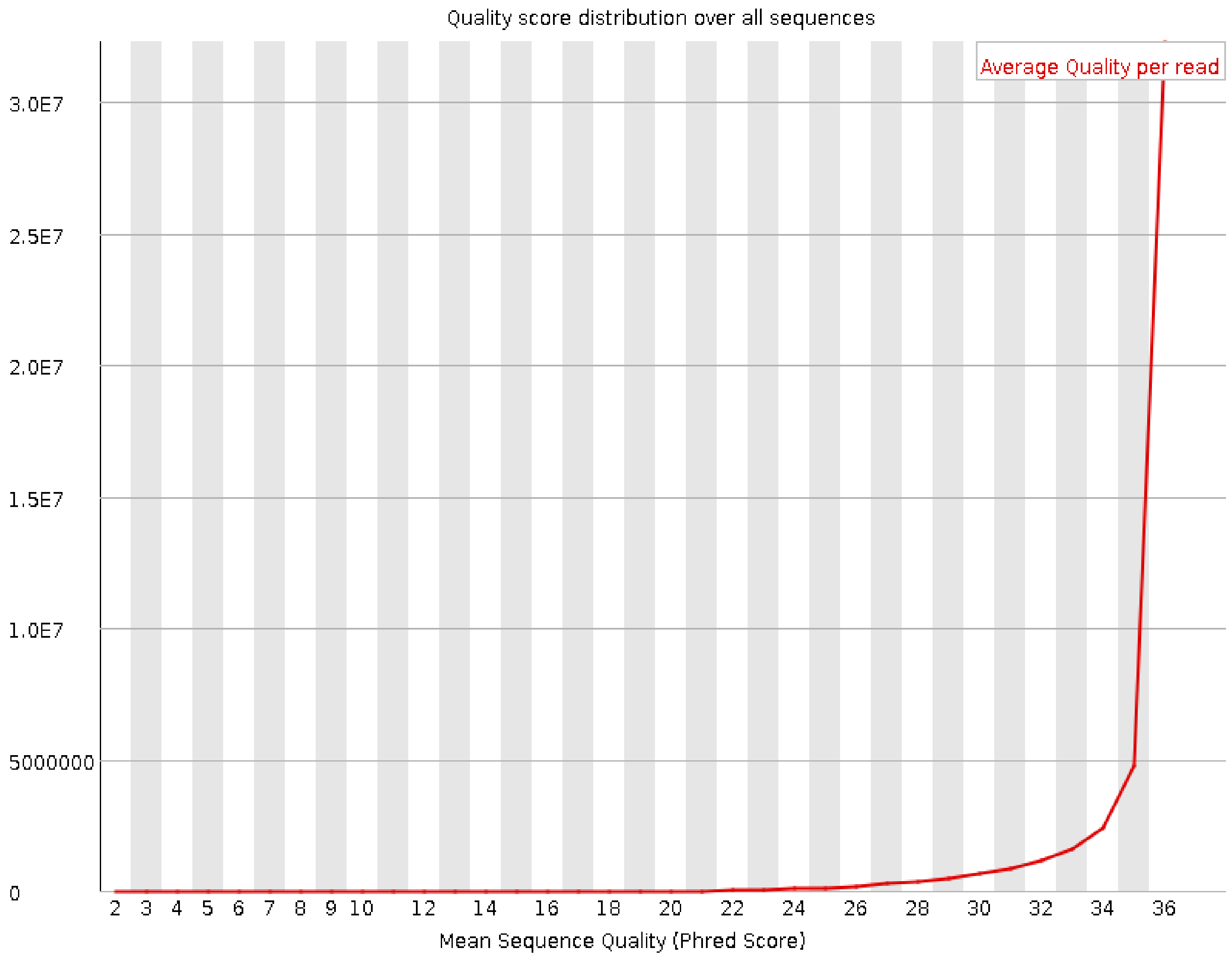

Per base sequence content

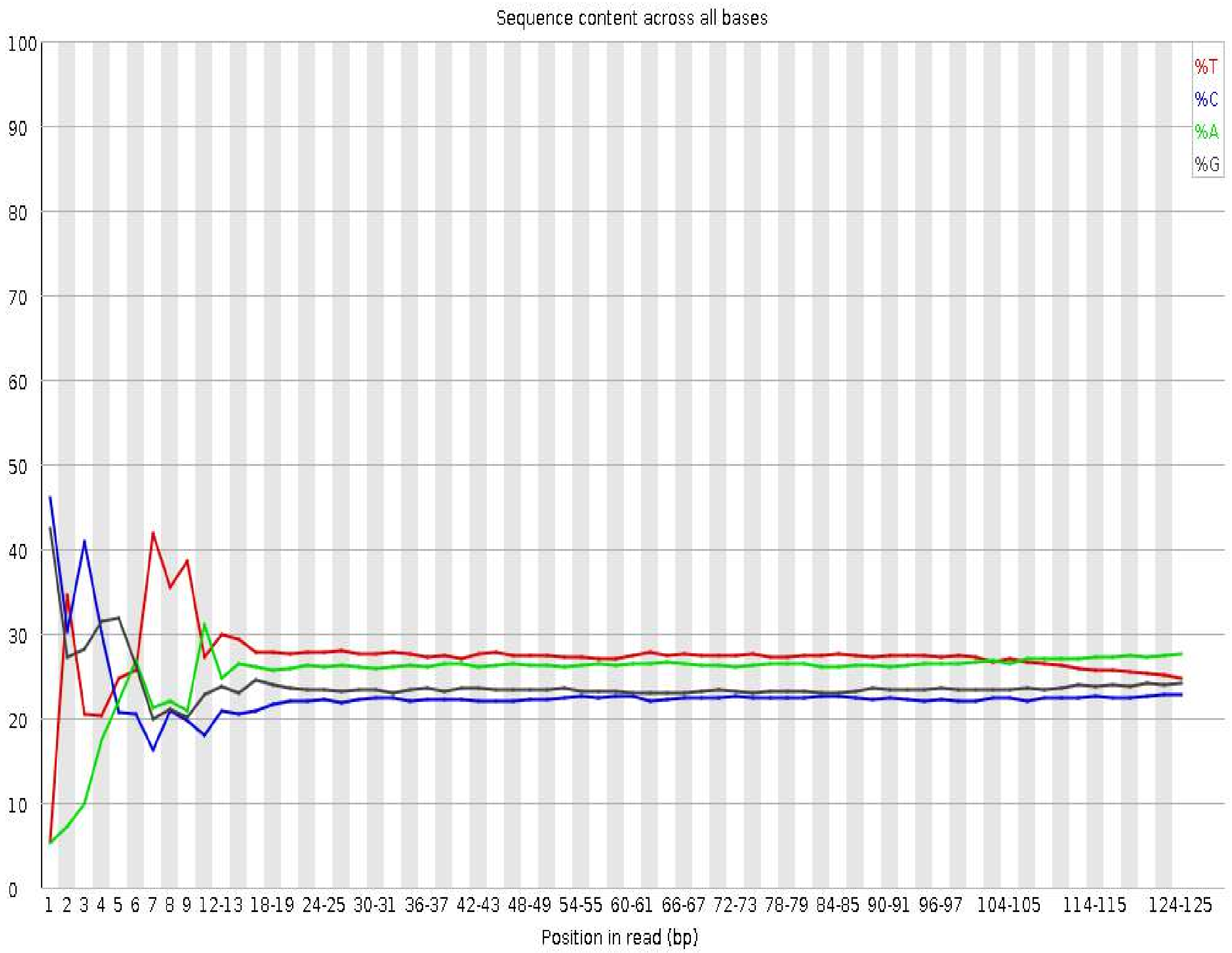

Per sequence GC content

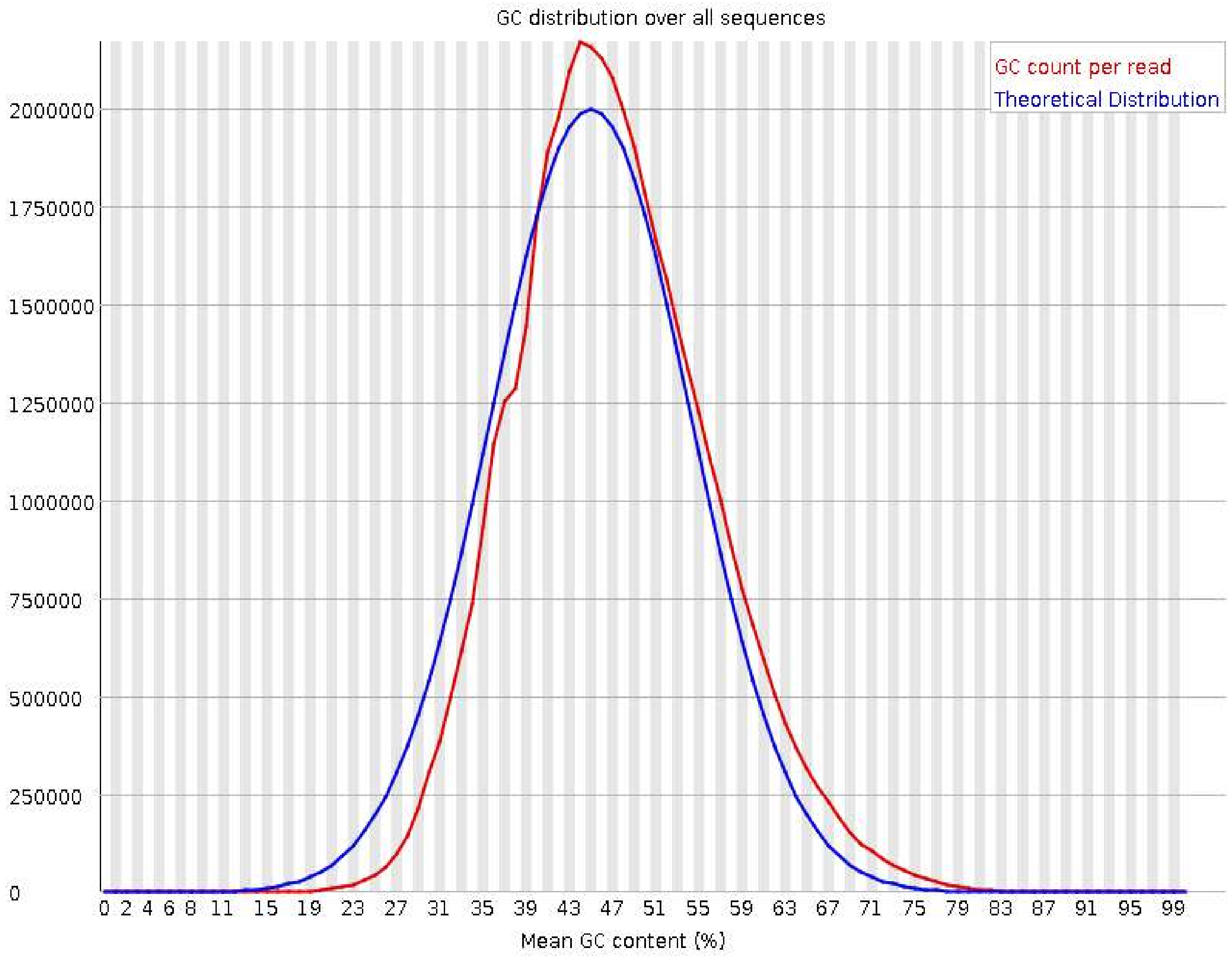

Per base N content

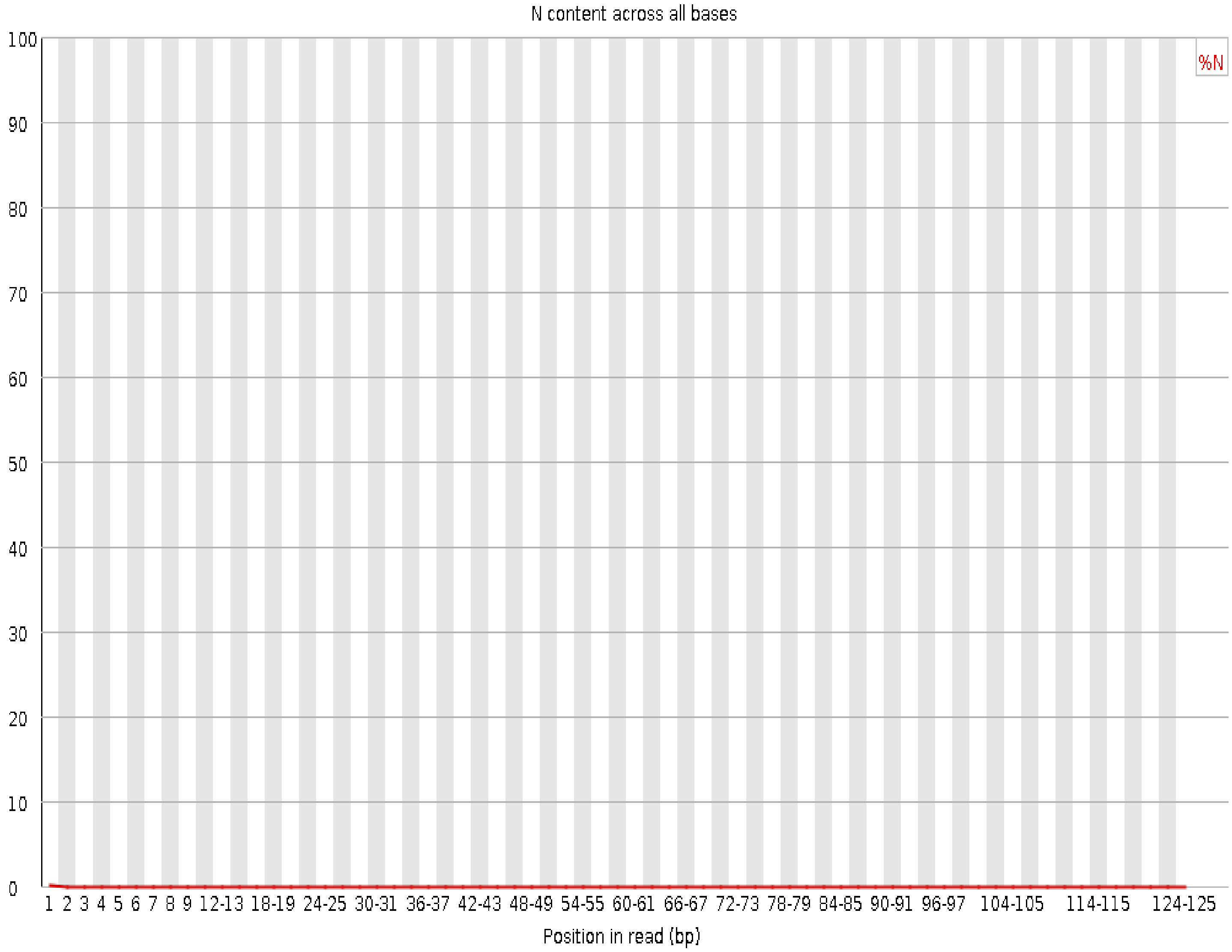

Sequence Length Distribution

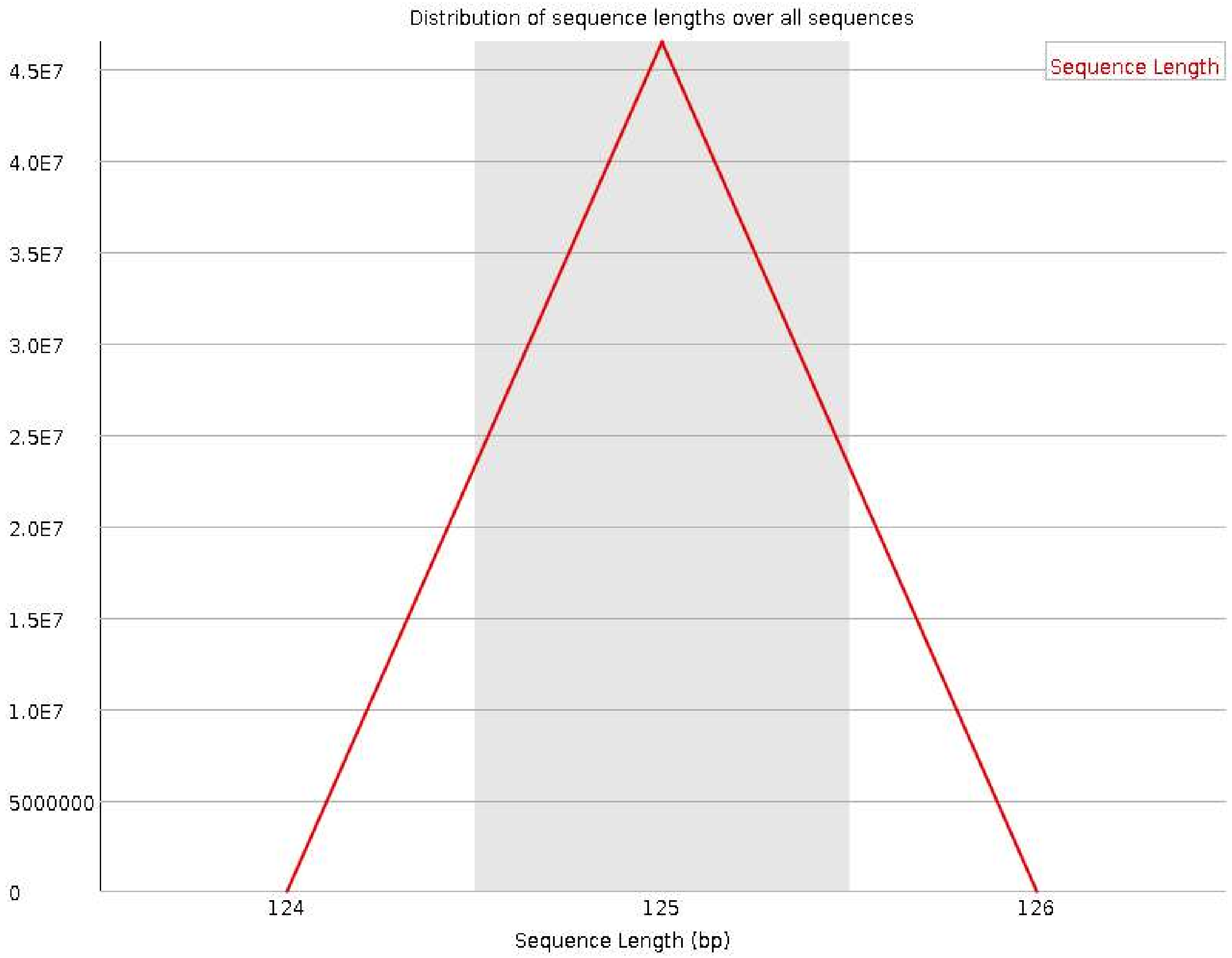

Sequence Duplication Levels

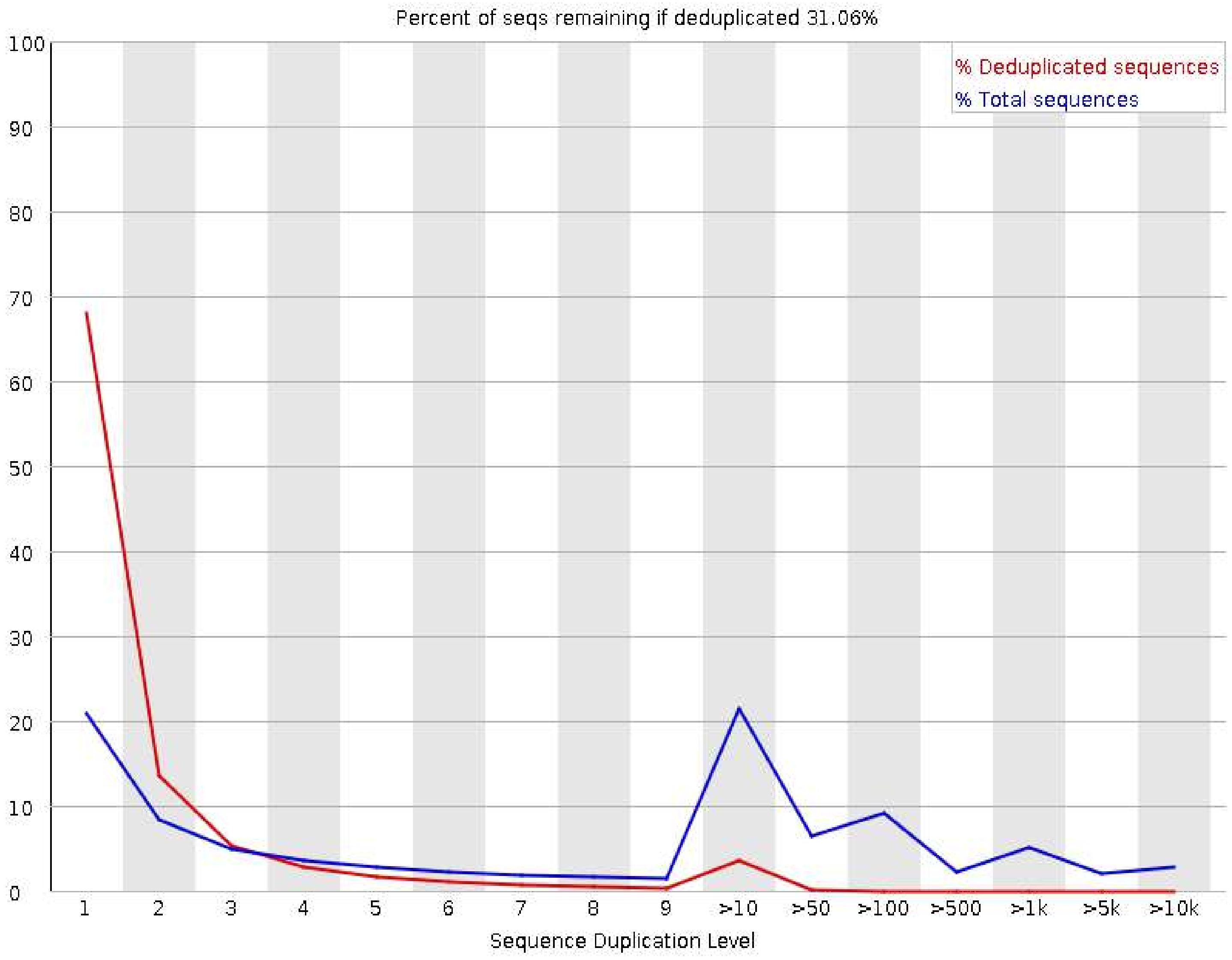

Overrepresented sequences

Sequence	Count	Percentage	Possible Source
GTCGGTTGTAAAGTGGTATAAGACTTGTCGTAATAGGCAAATAATTAAGA	65756	0.14143791485117463	No Hit

Adapter Content

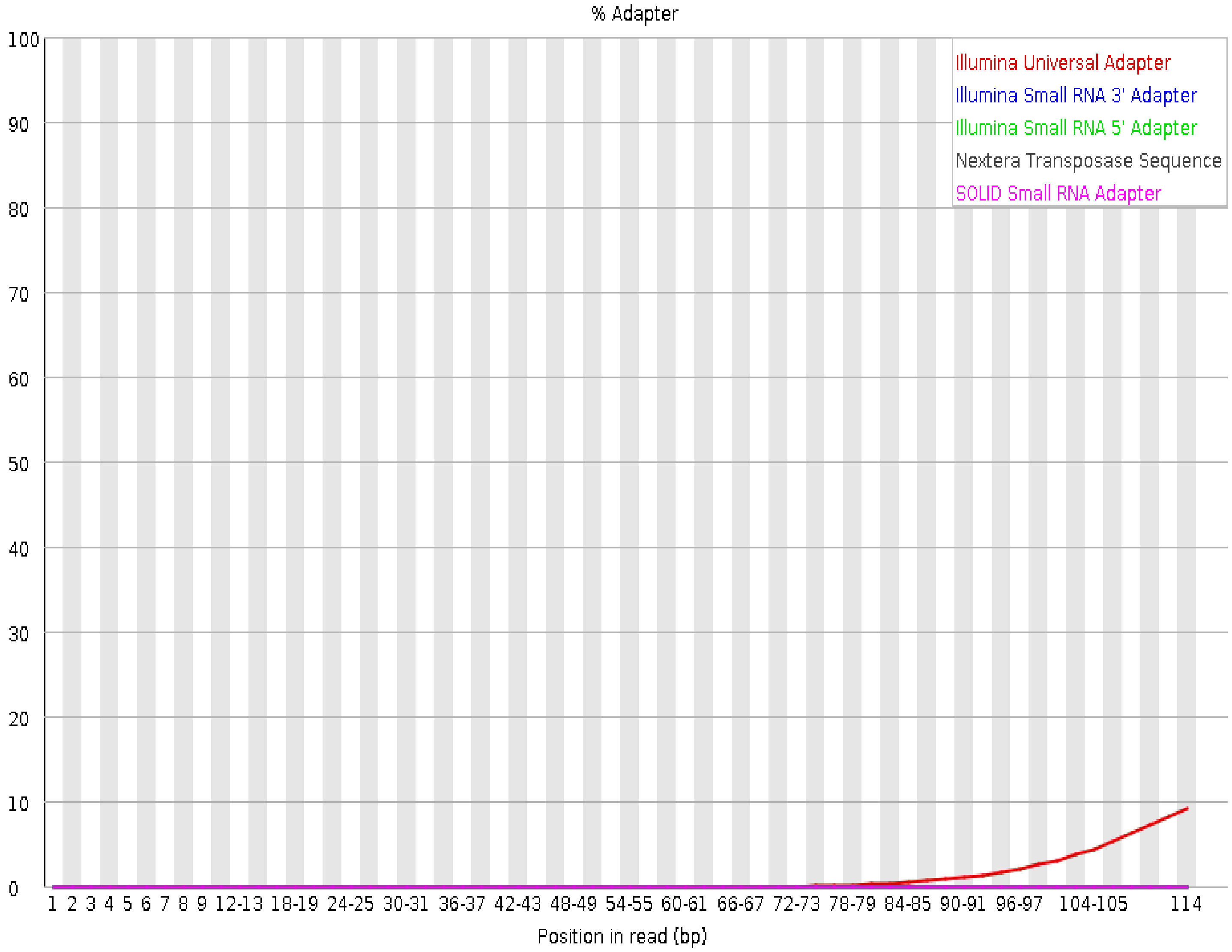

Produced by FastQC (version 0.11.8)

FastQC Report

Wed 9 Oct 2019
Eulamprus.Female.Brain_R2.fastq.gz

Summary

- Basic Statistics
- Per base sequence quality
- Per tile sequence quality
- Per sequence quality scores
- Per base sequence content
- Per sequence GC content
- Per base N content
- Sequence Length Distribution
- Sequence Duplication Levels
- Overrepresented sequences
- Adapter Content

Basic Statistics

Measure	Value
Filename	Eulamprus.Female.Brain_R2.fastq.gz
File type	Conventional base calls
Encoding	Sanger / Illumina 1.9
Total Sequences	46491070
Sequences flagged as poor quality	0
Sequence length	125
%GC	46

Per base sequence quality

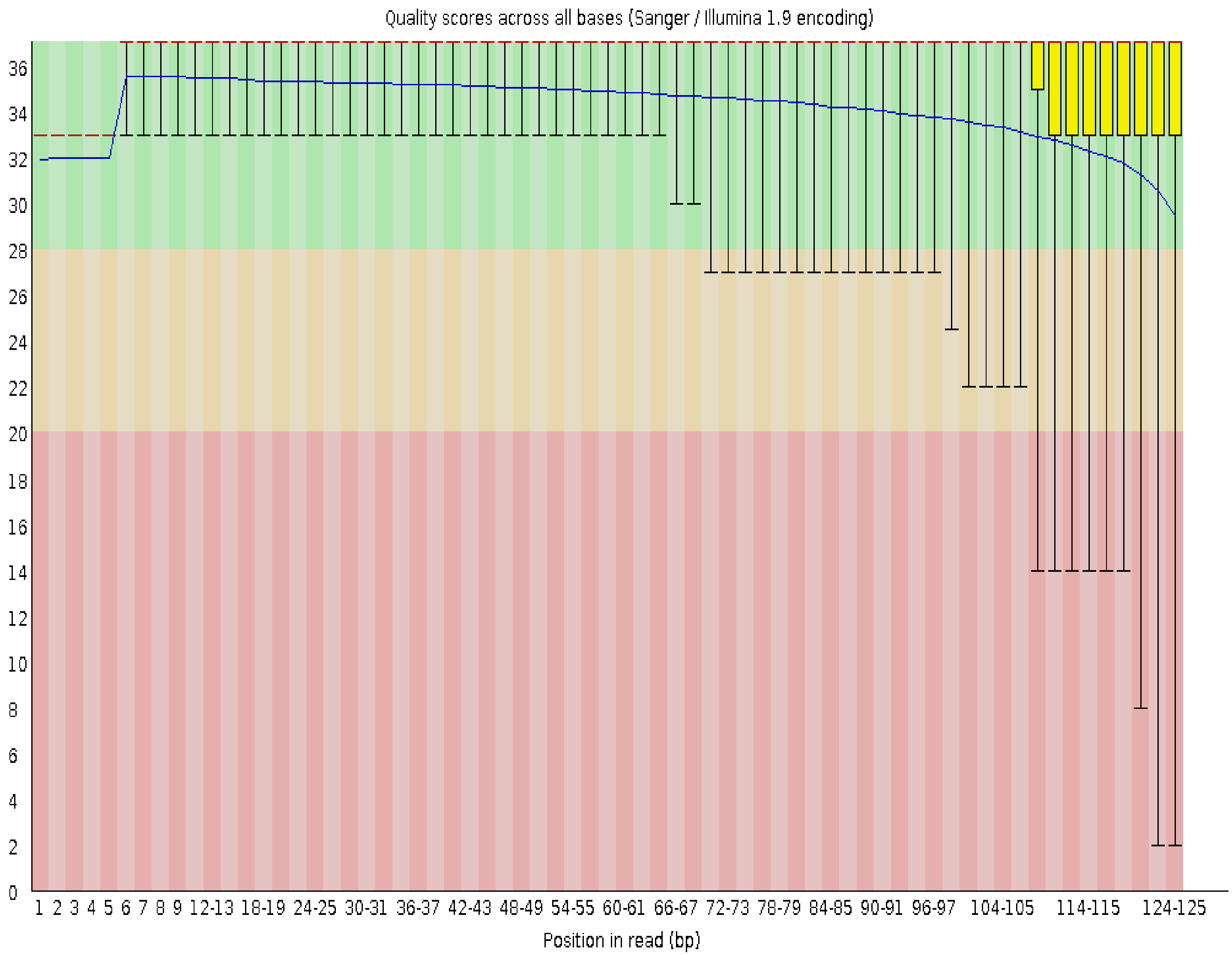

Per tile sequence quality

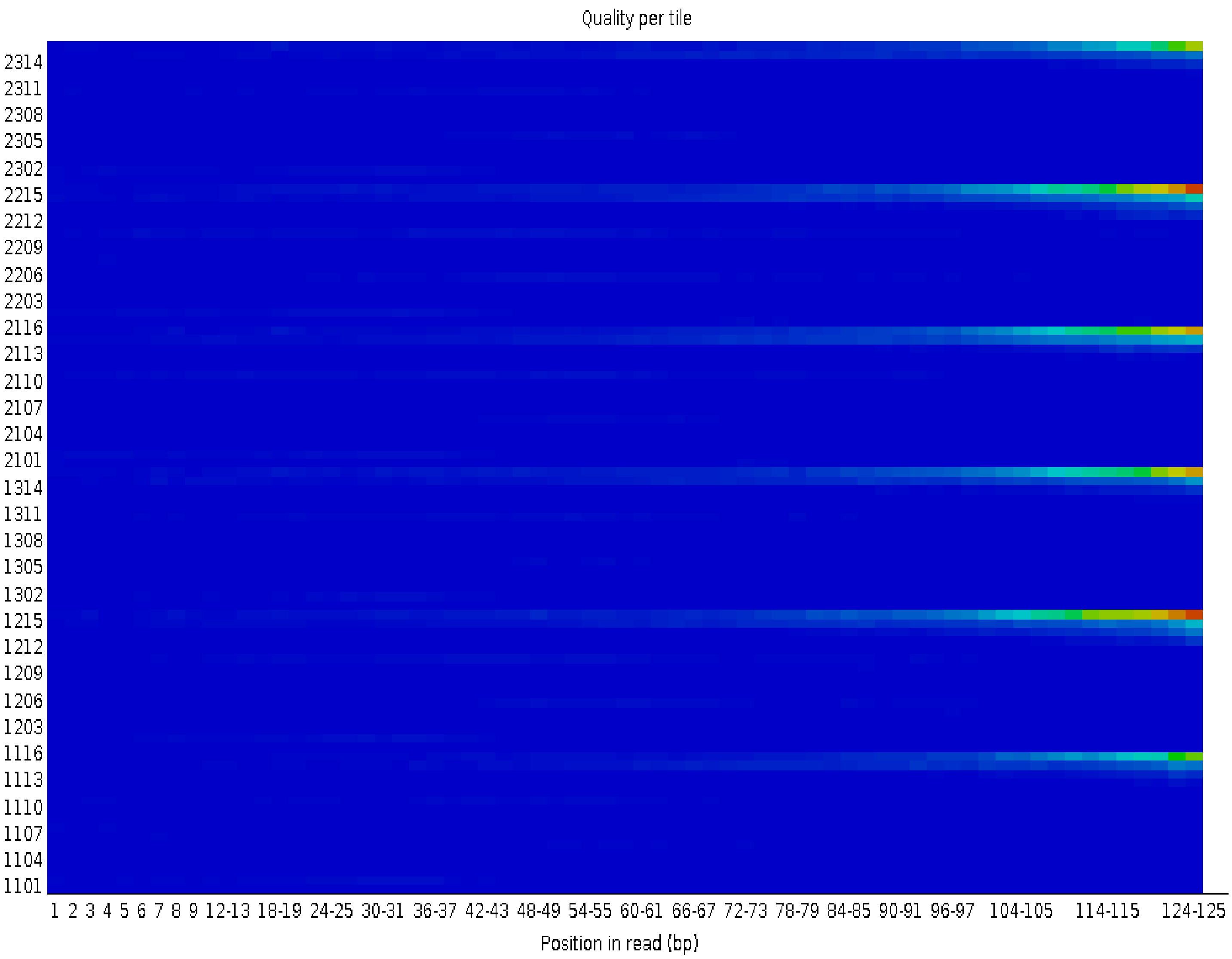

Per sequence quality scores

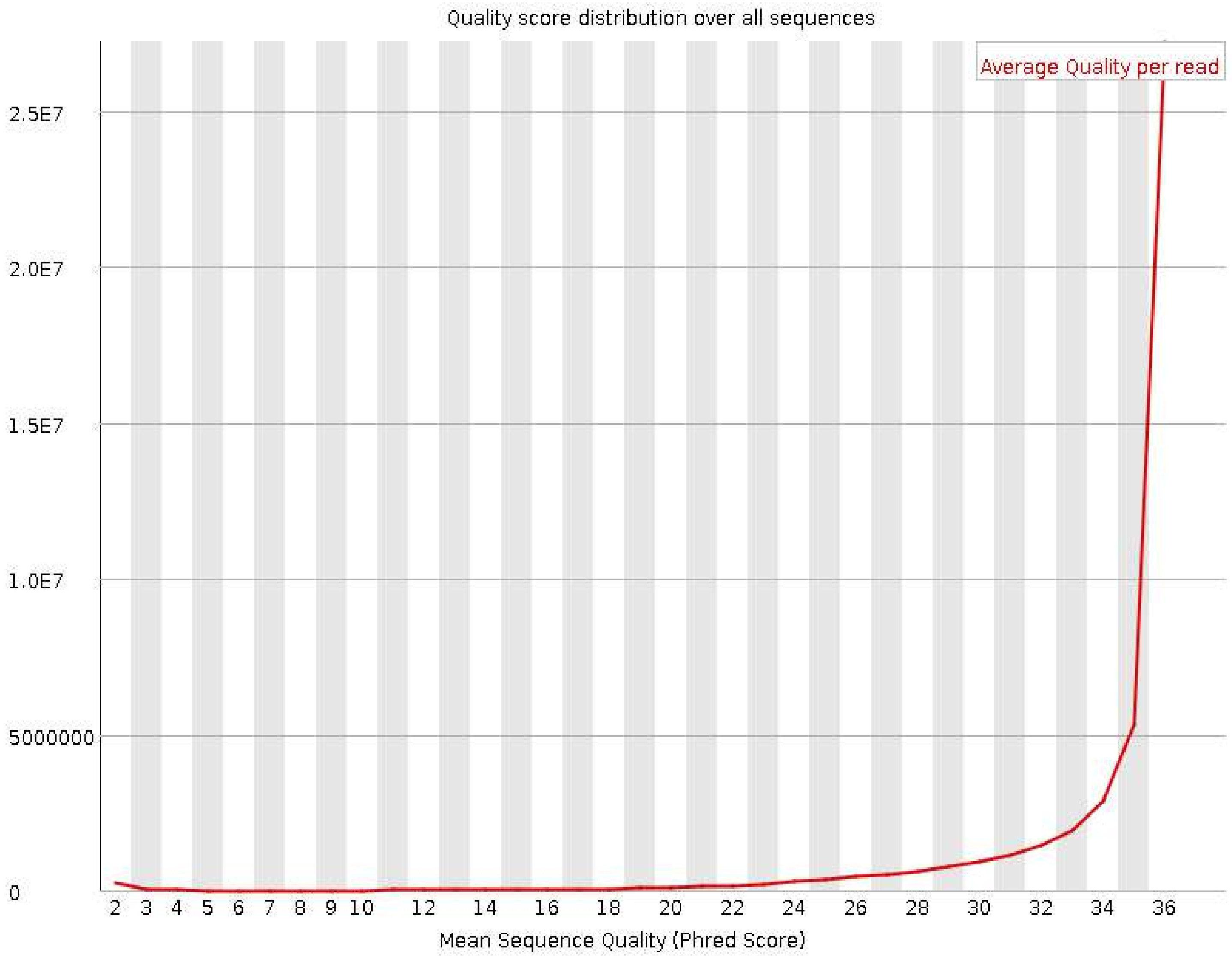

Per base sequence content

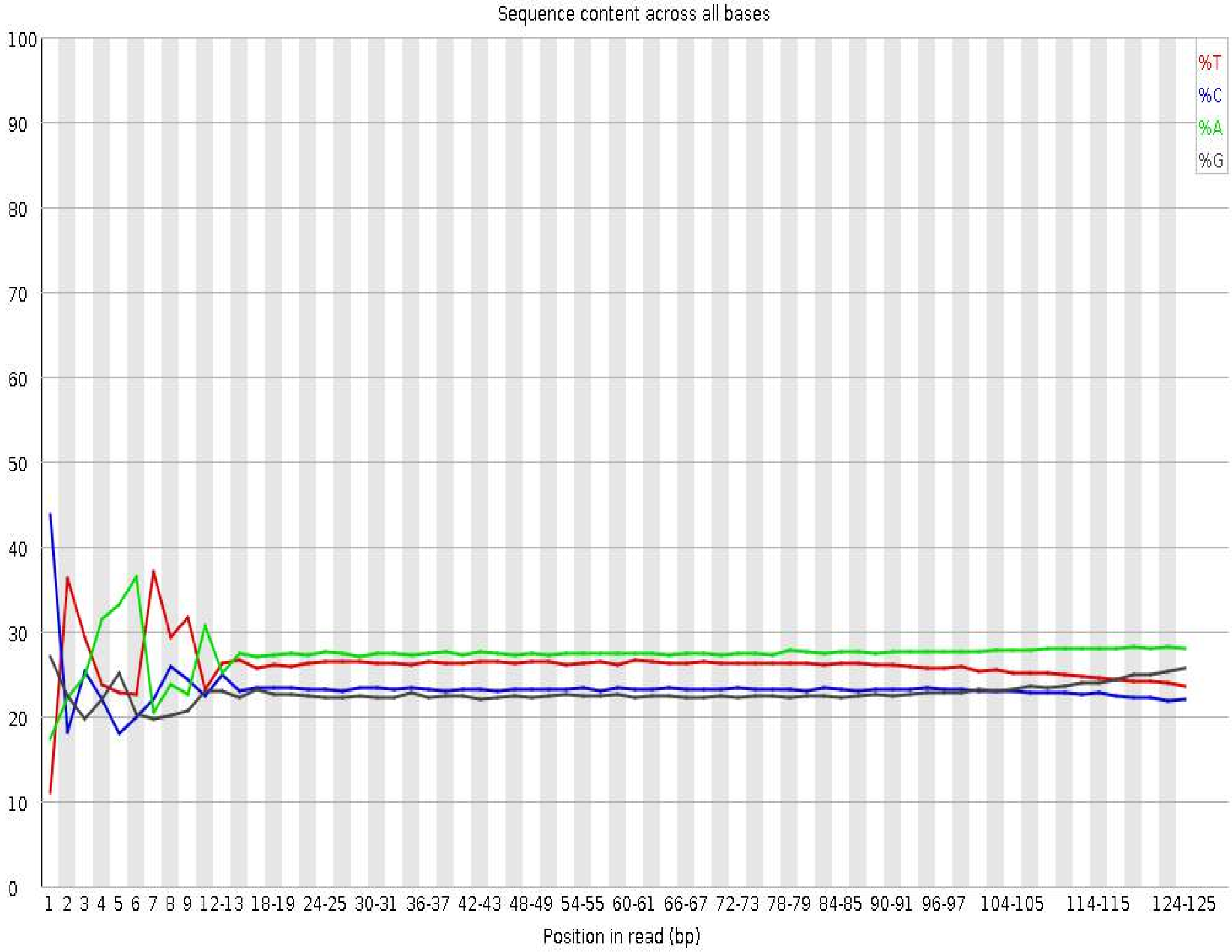

Per sequence GC content

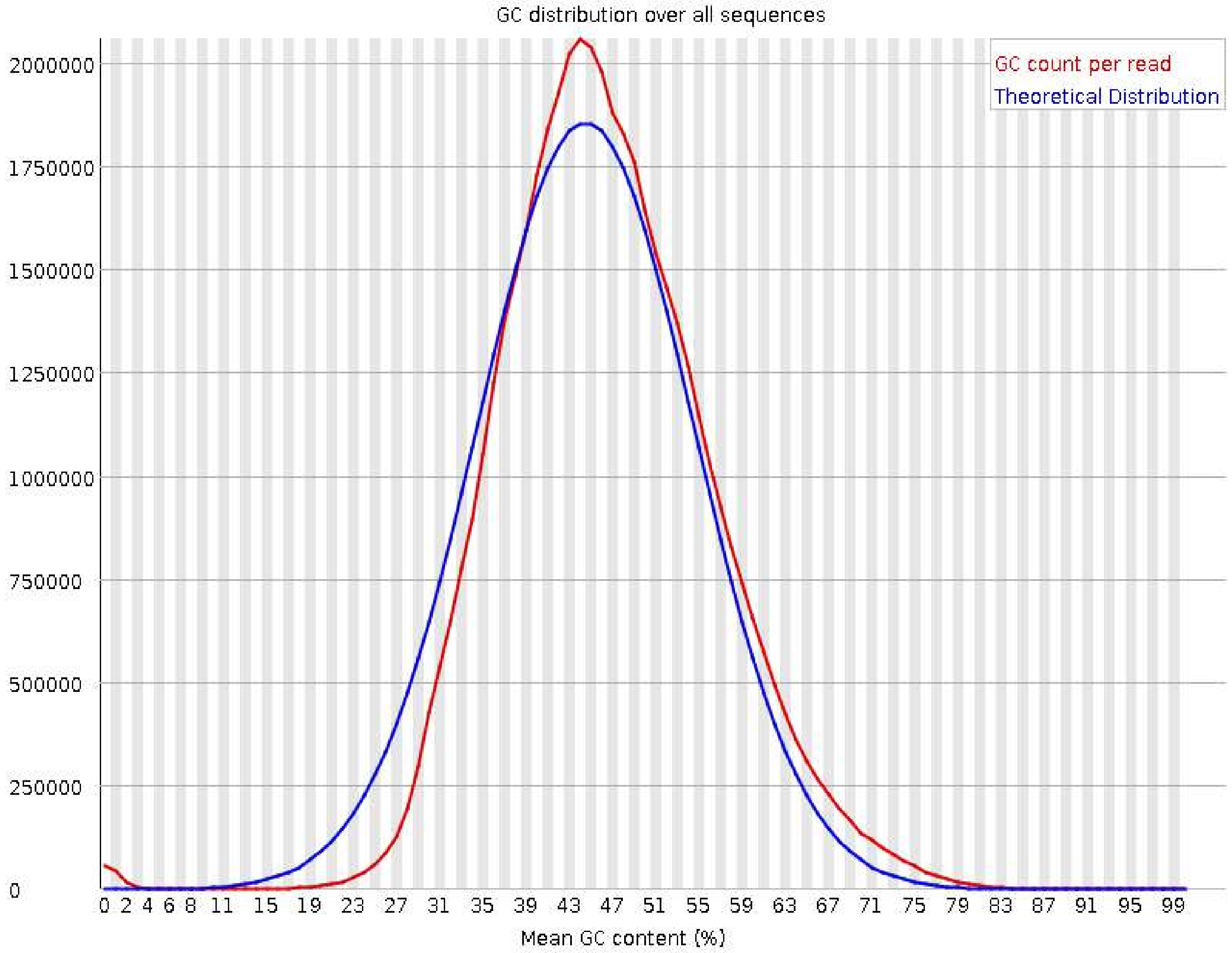

Per base N content

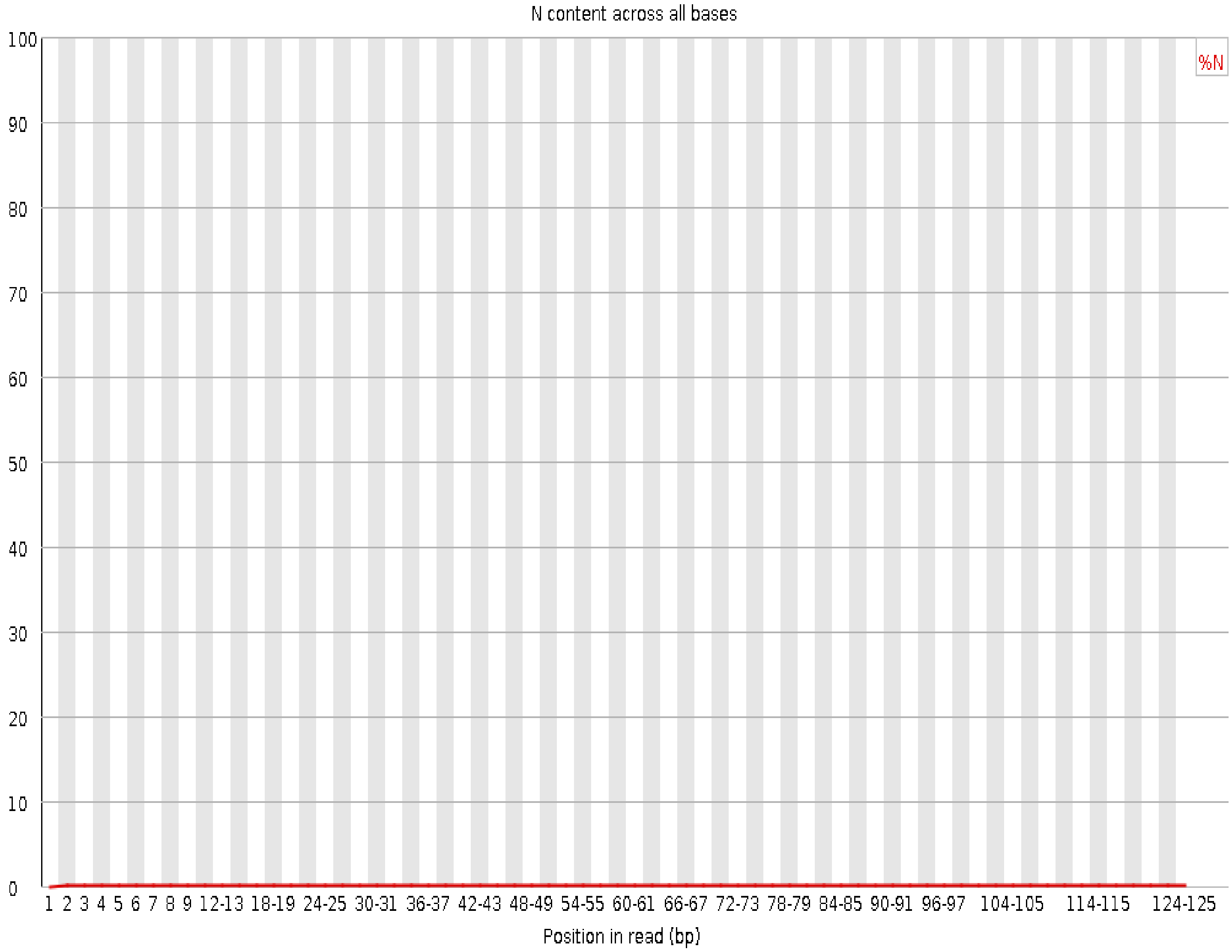

Sequence Length Distribution

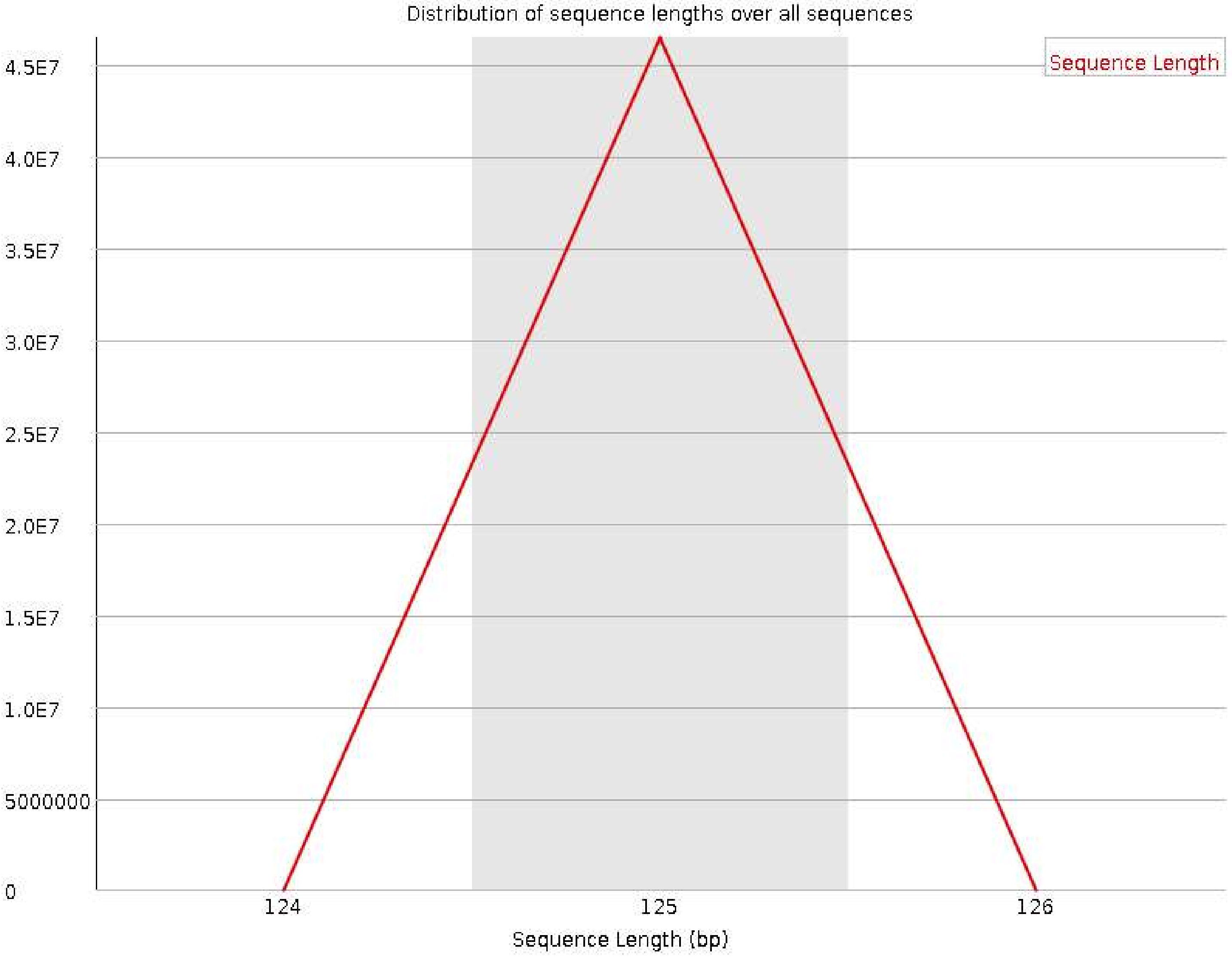

Sequence Duplication Levels

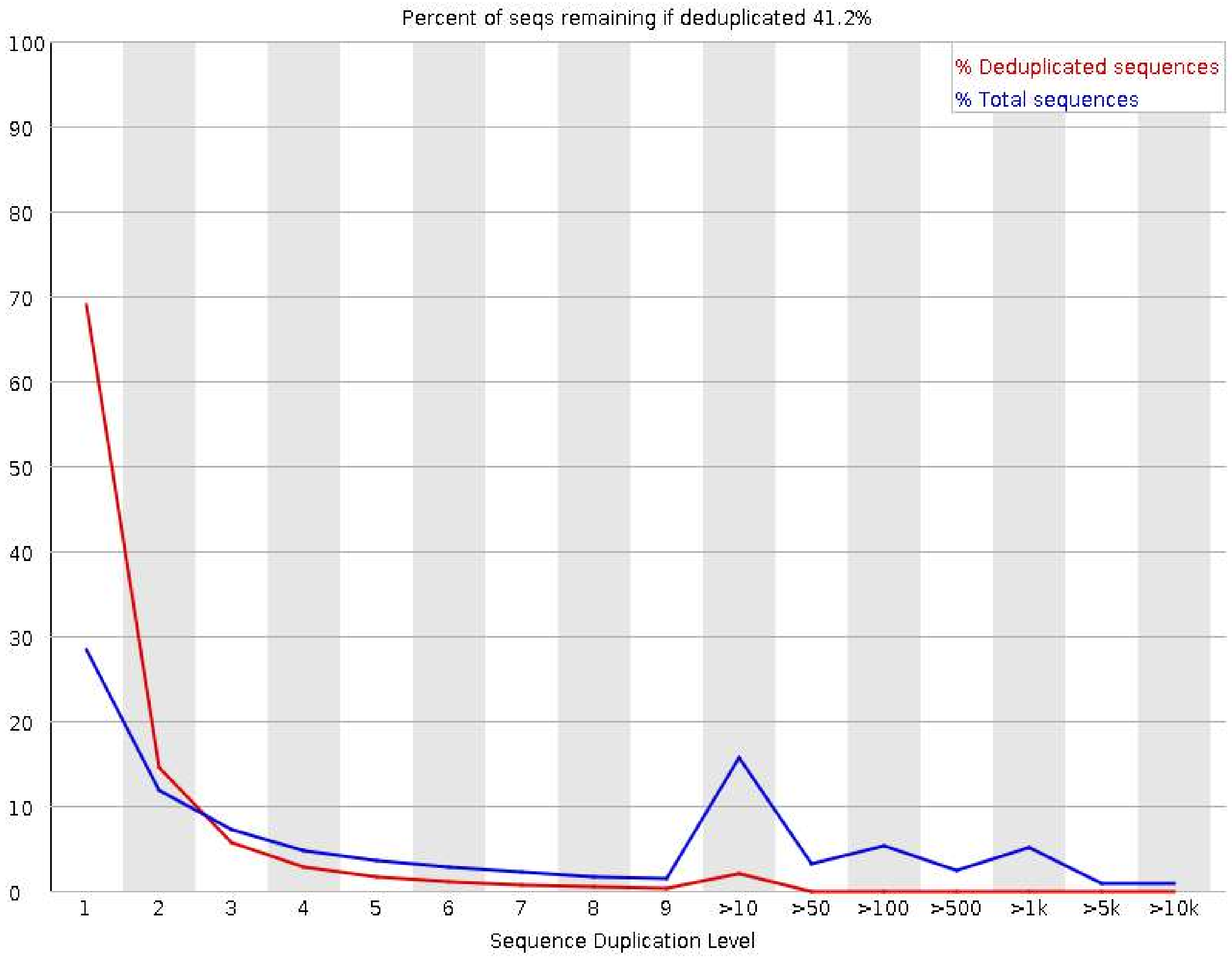

Overrepresented sequences

Sequence	Count	Percentage	Possible Source
CTCAGTTCTCTTGAAACCTACATAAATTCGCAATTATGACAATATATTAC	67000	0.14411369753374142	No Hit

Adapter Content

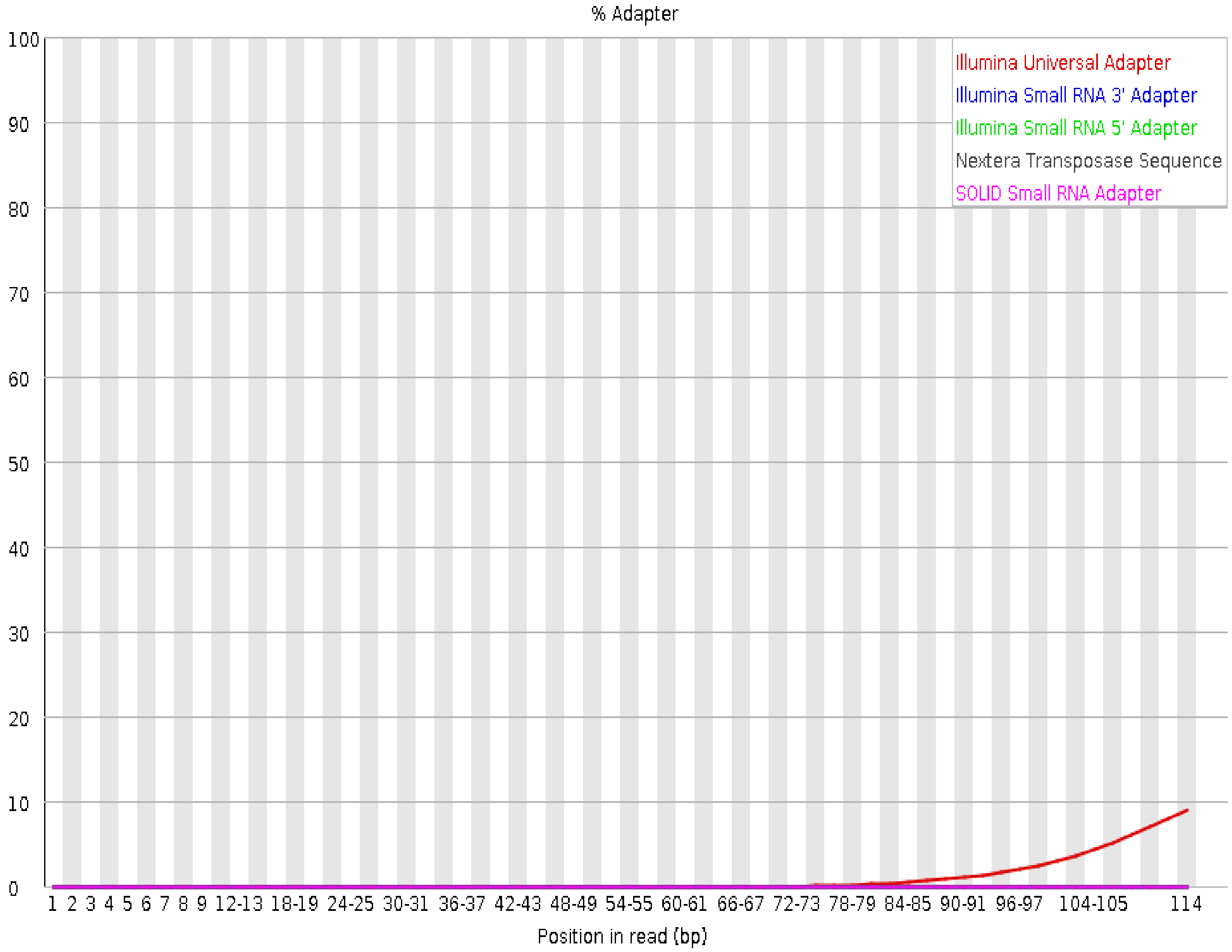

Produced by FastQC (version 0.11.8)

FastQC Report

Wed 9 Oct 2019
Eulamprus.Female.Liver_R1.fastq.gz

Summary

- Basic Statistics
- Per base sequence quality
- Per tile sequence quality
- Per sequence quality scores
- Per base sequence content
- Per sequence GC content
- Per base N content
- Sequence Length Distribution
- Sequence Duplication Levels
- Overrepresented sequences
- Adapter Content

Basic Statistics

Measure	Value
Filename	Eulamprus.Female.Liver_R1.fastq.gz
File type	Conventional base calls
Encoding	Sanger / Illumina 1.9
Total Sequences	41185030
Sequences flagged as poor quality	0
Sequence length	125
%GC	46

Per base sequence quality

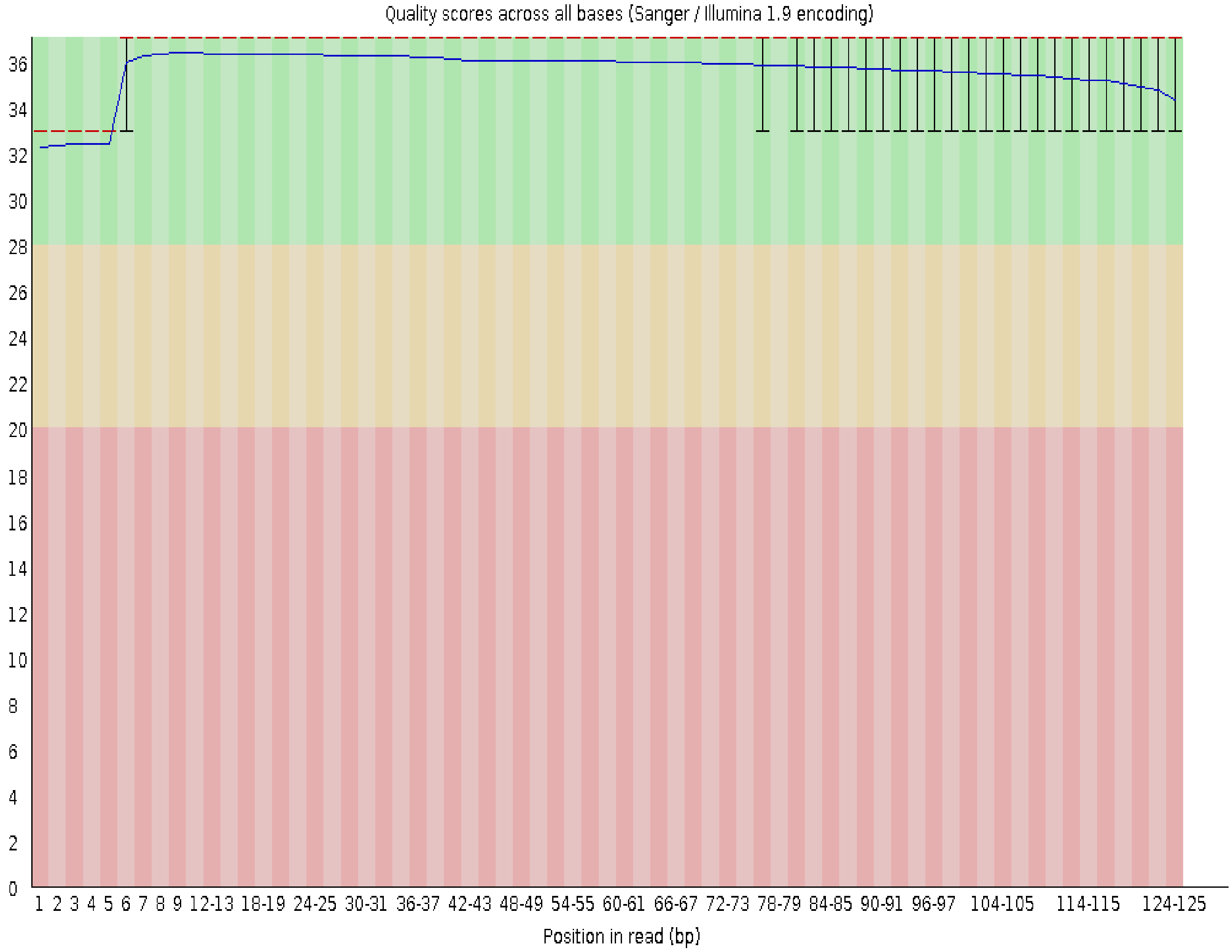

Per tile sequence quality

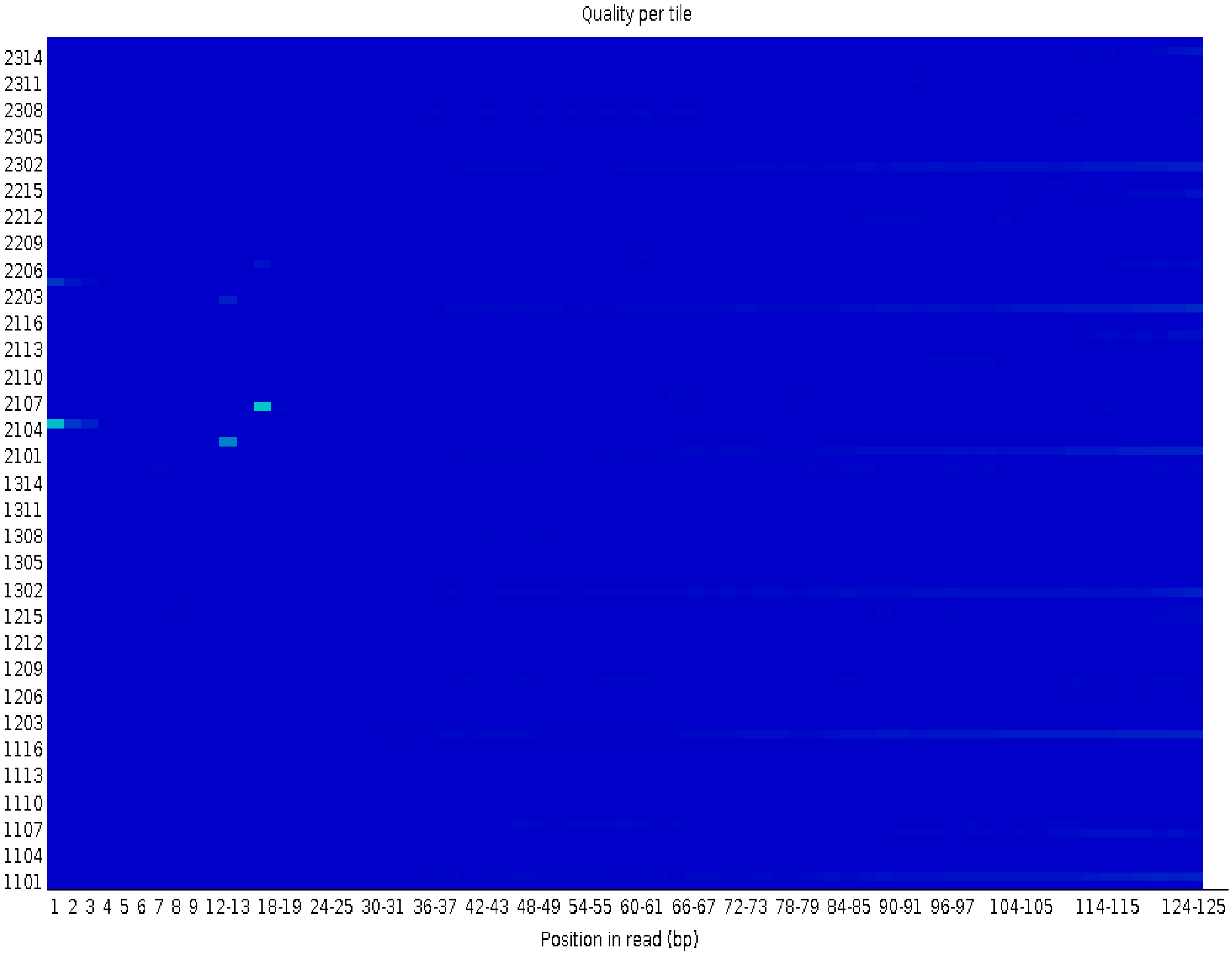

Per sequence quality scores

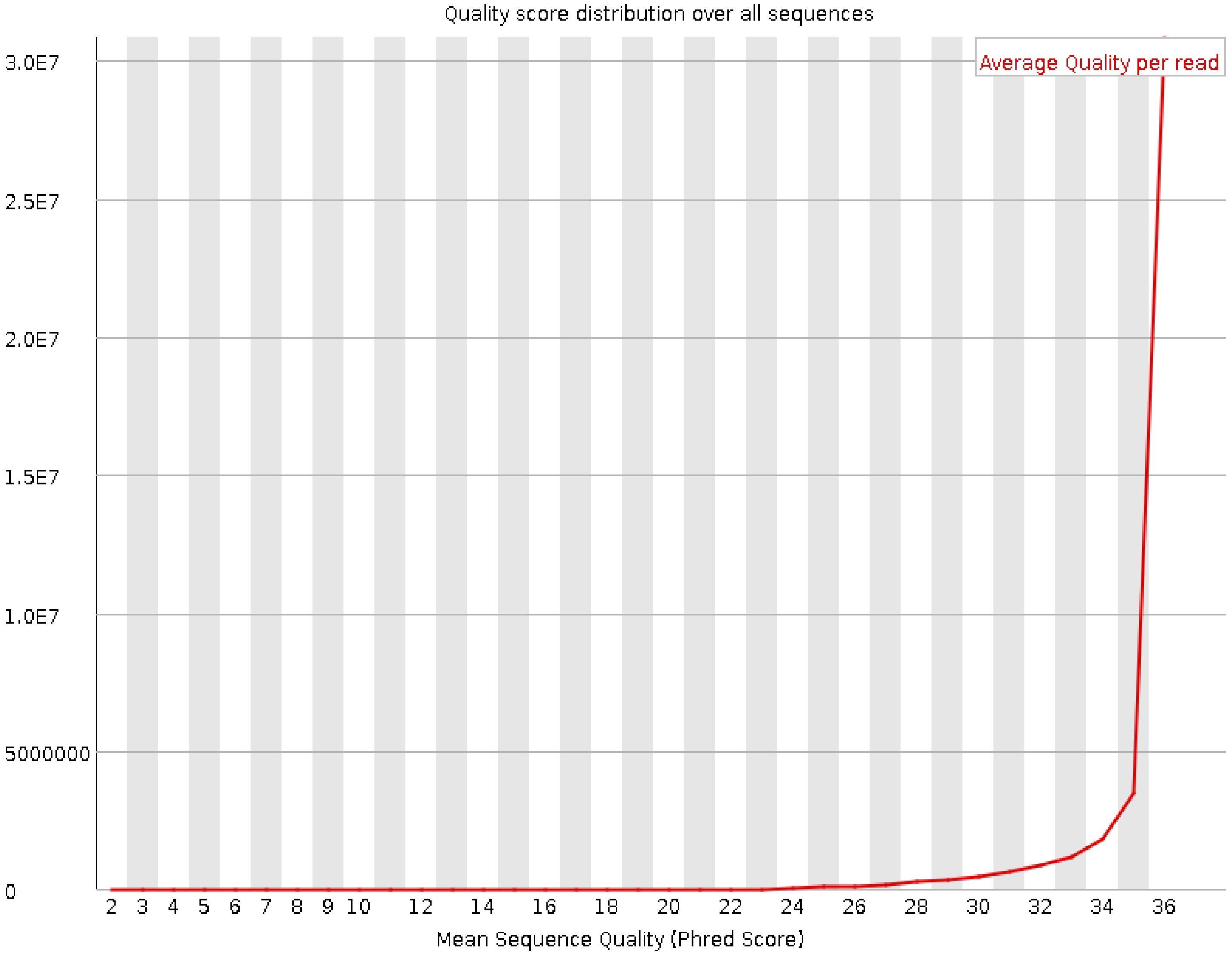

Per base sequence content

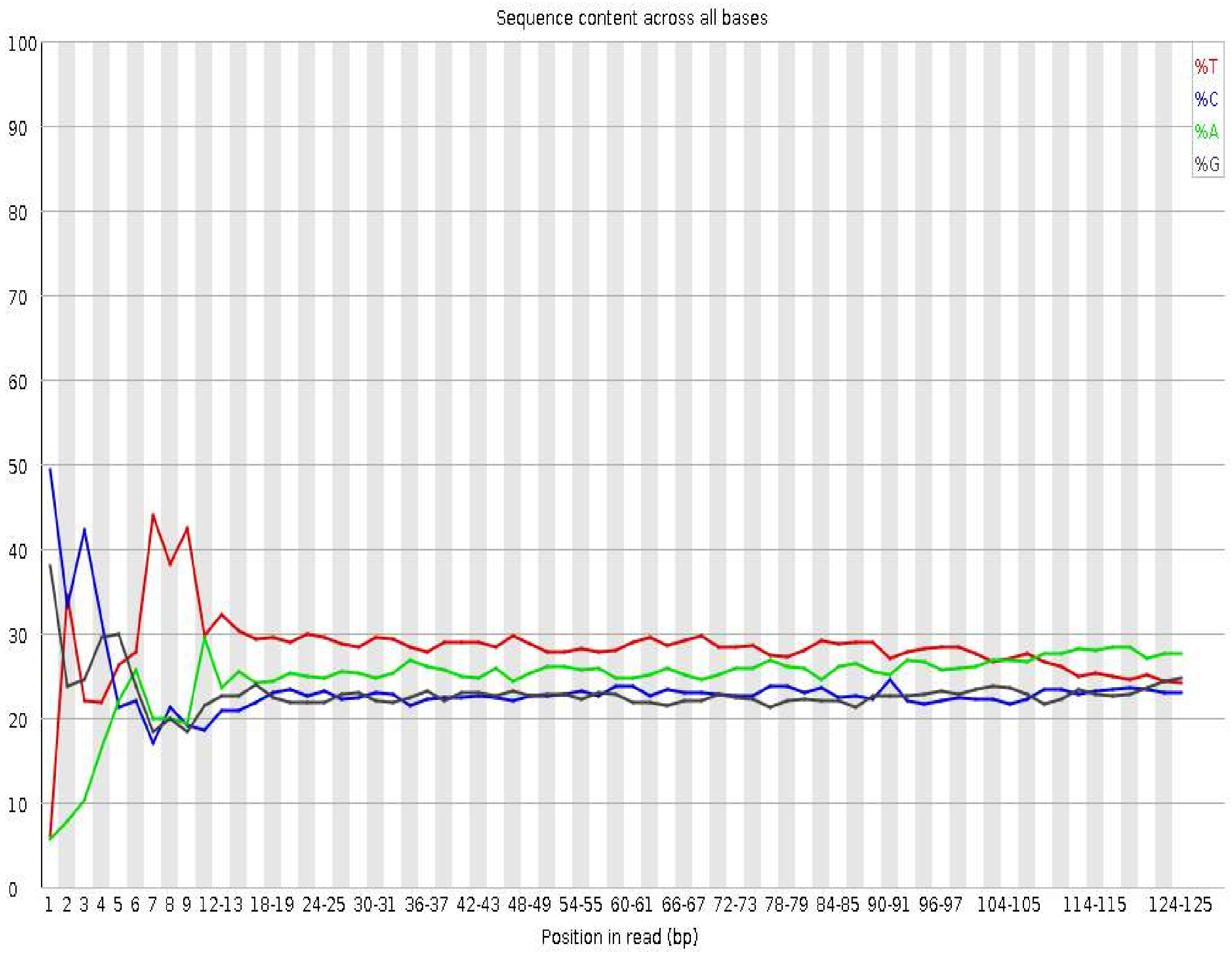

Per sequence GC content

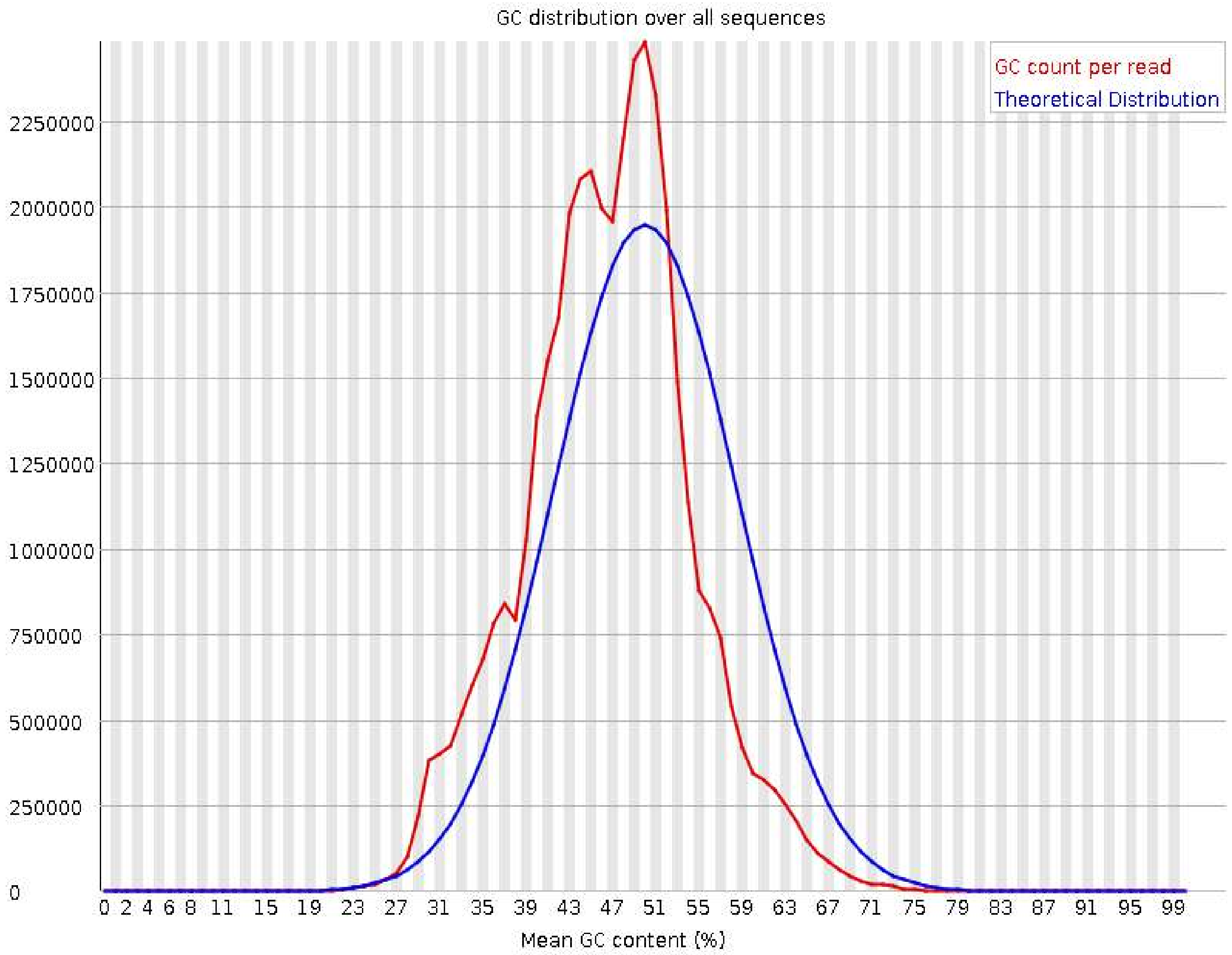

Per base N content

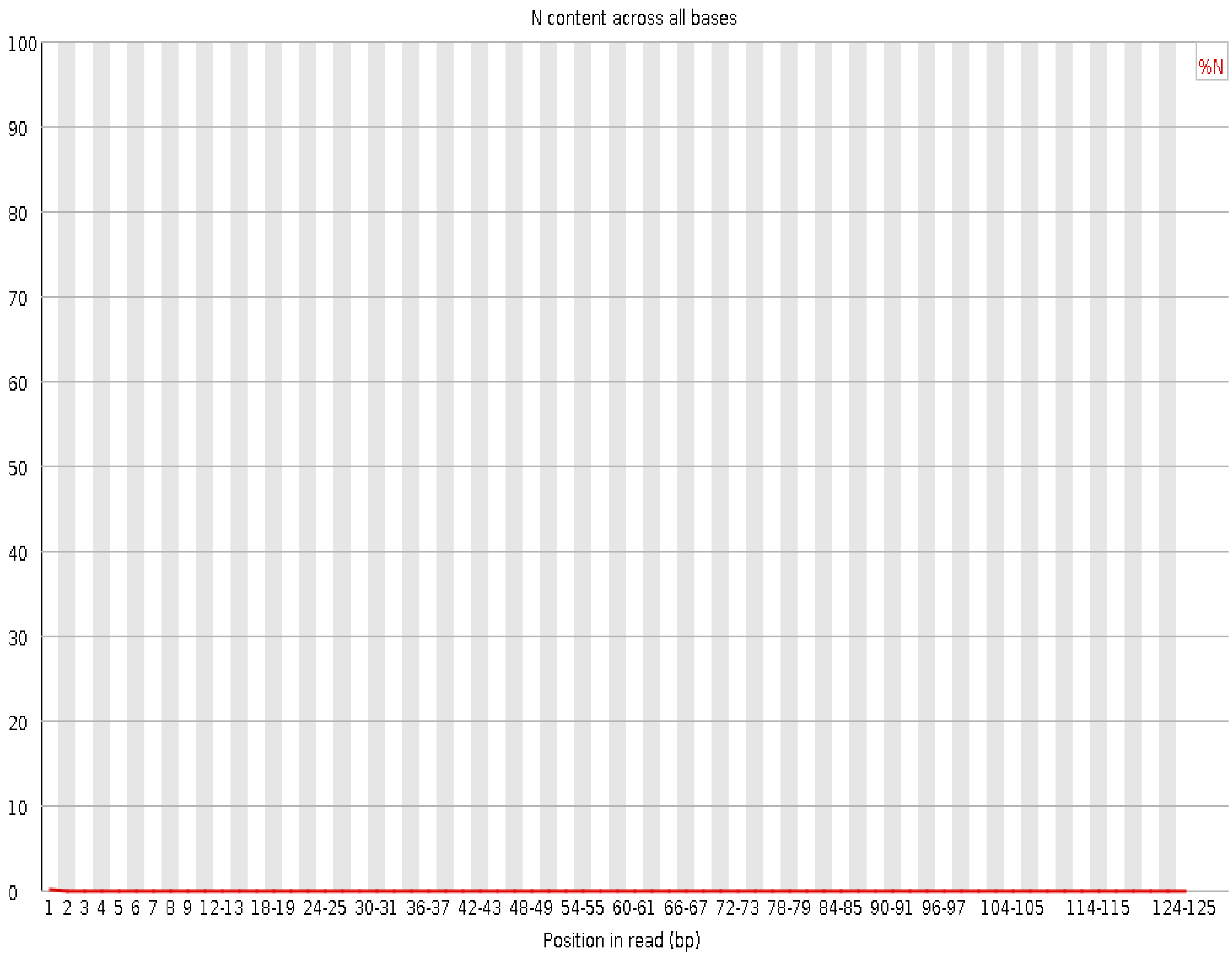

Sequence Length Distribution

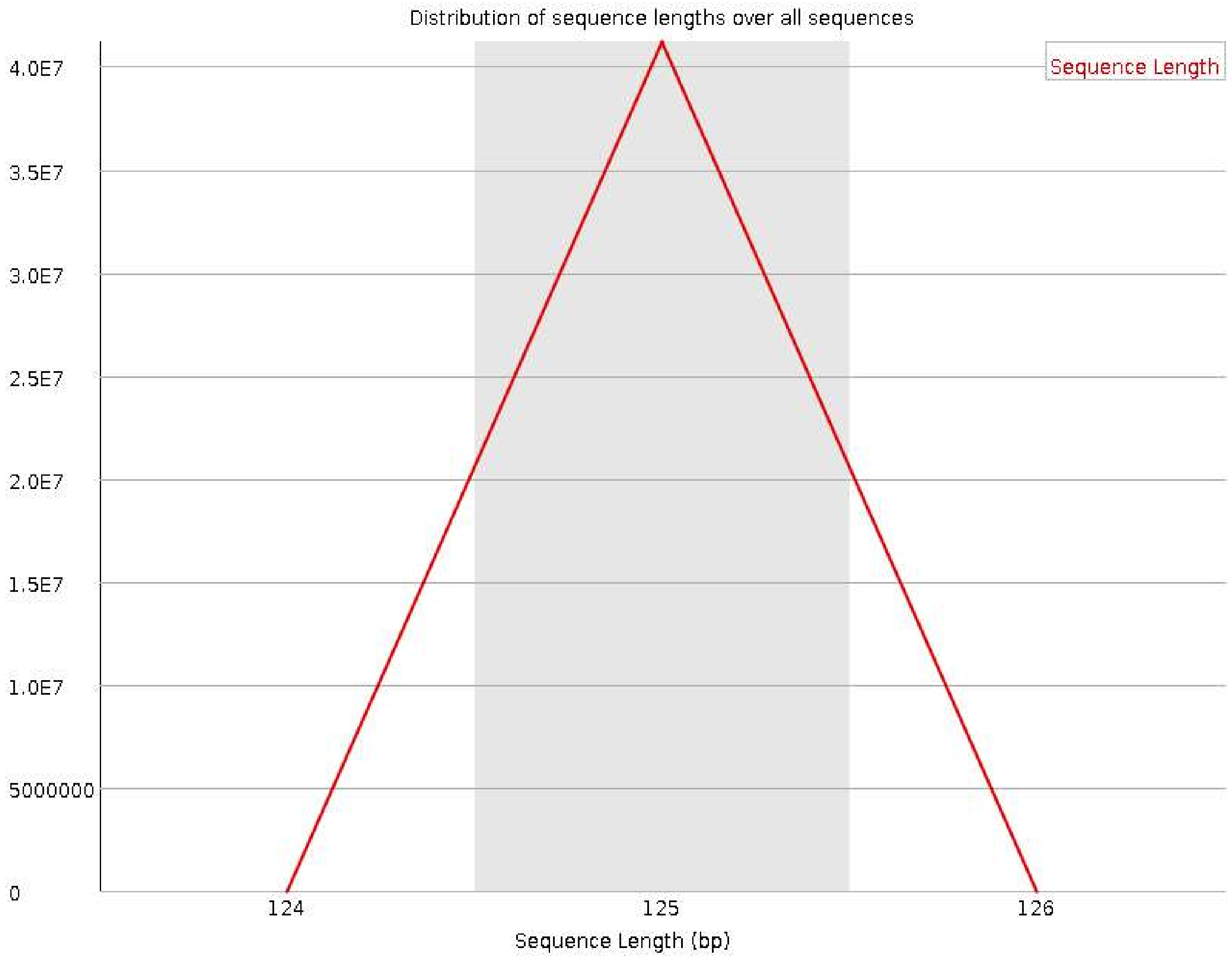

Sequence Duplication Levels

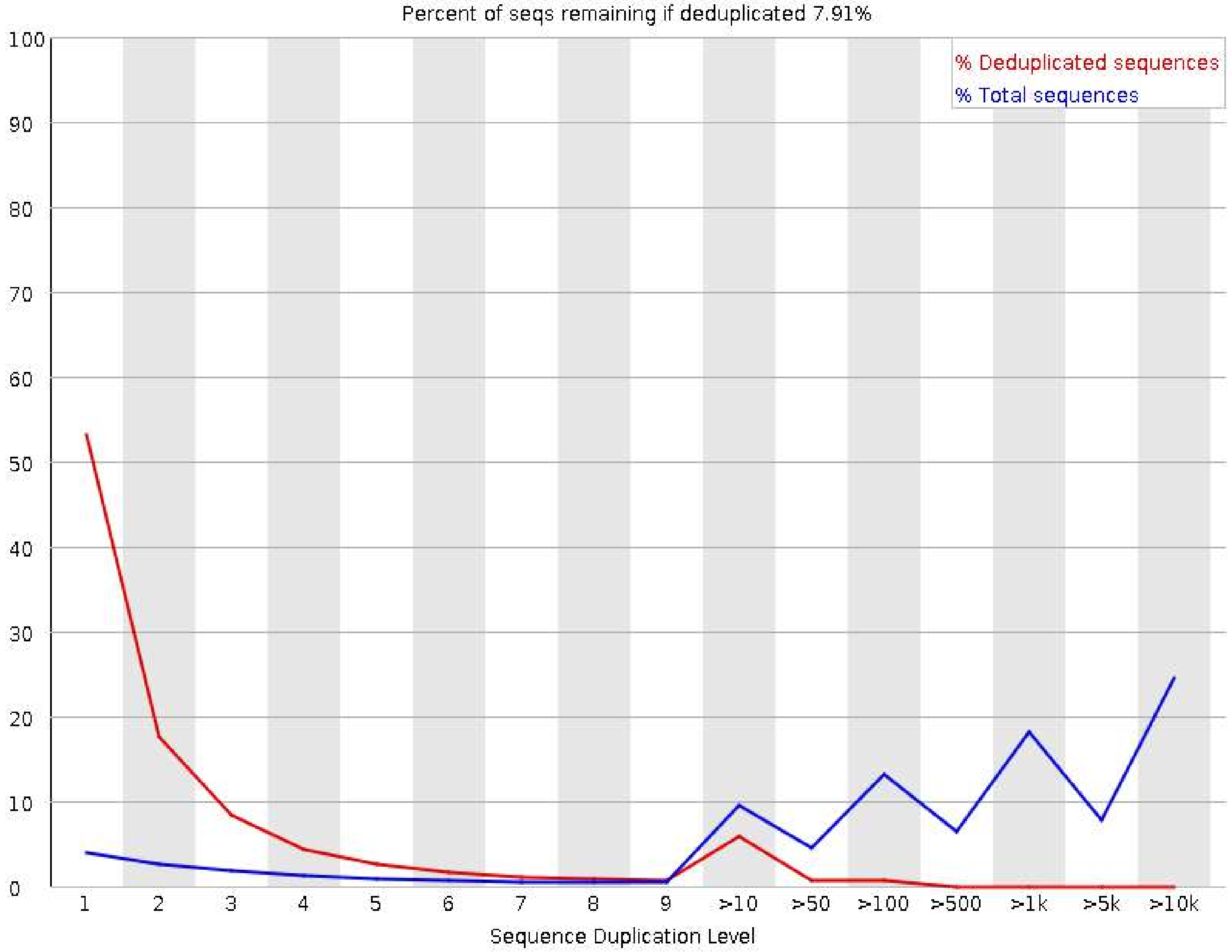

Overrepresented sequences

Sequence	Count	Percentage	Possible Source
GCTTGGTGTTTTGGTGTATTCGAGCTGCTTTAATAAACTTCTTTATGAGA	189427	0.45994139132592593	No Hit
GTAAGGATTTAAGAAAGGTTCTTATGAGGATGGTAAGGTAGGGTAGGGTA	173109	0.42032019886837524	No Hit
TAAGGATTTAAGAAAGGTTCTTATGAGGATGGTAAGGTAGGGTAGGGTAA	152163	0.36946191370990866	No Hit
GGGTAGGGTAACTCCAGGCACTTACTAGCTGTTGTCCTGAATTAATTTGT	144271	0.35029961129080156	No Hit
GTCGTCTTTGTCGTGGCCGGGACTAGGTTATCTAAGTAAGTATCGTTCGG	139324	0.3382879653116678	No Hit
GCTGGTGCTAAGTGCATGTTGGGCGAAGGTAAGTGGTATGGAGATGATAG	120331	0.29217169442392055	No Hit
CCCTGCTGGAGTTGGGCTACTATACCCCAATATTTCATCTTGAACTTGGC	103197	0.250569199536822	No Hit
GGCTTGGTGTTTTGGTGTATTCGAGCTGCTTTAATAAACTTCTTTATGAG	98522	0.2392179877008709	No Hit
GTCCACAACGATCAGTAACAGTACACCGCCCTCCATAGAGGCAATGGTAA	97037	0.23561230864709823	No Hit
GTGGCTTTTCGGATGTGCATACCCTTAATCGCTTGGGCTGTCTCCCGTGT	96512	0.2343375736280877	No Hit
CGGGCTTGGTGTTTTGGTGTATTCGAGCTGCTTTAATAAACTTCTTTATG	92838	0.22541685656171673	No Hit
CTTGGTGTTTTGGTGTATTCGAGCTGCTTTAATAAACTTCTTTATGAGAC	92589	0.2248122679527003	No Hit

Sequence	Count	Percentage	Possible Source
GCCGGATGTAATGGTCTCCACCATTTCGTTGCCTACTATTTCCTGTTCAT	87879	0.2133760737821485	No Hit
GGGGGATTAGAAAAAGTATAACTCTTTACTTTGTGTCATCCTAGGCTGTC	82728	0.20086910219562787	No Hit
CTCTGCCTTTTTTCCTCTATACTCCTATCTCTTTAACAGCACTCATTGAT	82175	0.19952638130893677	No Hit
CCGGCACTTAGATTCCTTCACAGTCCAAACGAATGAGCAGCTCTTCCTCC	74217	0.18020382648743974	No Hit
GTCGATTTAAGAGTTAGATACCTAAACGGGCTTGGTGTTTTGGTGTATTC	74140	0.1800168653513182	No Hit
GTCAGATCTGTATCTATTATTGTTGAAGATATCTAGGGAGTGAGTGCATA	72676	0.1764621756983059	No Hit
CTCCTTCCTAATATCTTTCGTTTATGTAGTCGATTTAAGAGTTAGATACC	72106	0.1750781776776659	No Hit
CCTCTGCCTTTTTTCCTCTATACTCCTATCTCTTTAACAGCACTCATTGA	71653	0.1739782634612625	No Hit
CACAACGATCAGTAACAGTACACCGCCCTCCATAGAGGCAATGGTAACAC	70875	0.172089227566424	No Hit
CCCAGGGCAAATGTATTCGTGACTGATTGGTTGGGAGTTTTGGATGTGAT	69982	0.16992096400075465	No Hit
CTCTGTCTCTGTGAAGTGGCCAACCATGCTAGCACAGTAGTCCCAGTTAC	68015	0.16514495679619512	No Hit
GTTGGGAGTTTTGGATGTGATGACAAAGGTGTTCCCGGTTTGCTGGATCT	67778	0.16456950498761322	No Hit
GGGCTTGGTGTTTTGGTGTATTCGAGCTGCTTTAATAAACTTCTTTATGA	67591	0.16411545651417517	No Hit
GTTGCCGAGAGCGAGTGTGATTGATGTAGGAATCGCTGCACGTTTAGATG	64233	0.1559620085259134	No Hit
GGGGATTAGAAAAAGTATAACTCTTTACTTTGTGTCATCCTAGGCTGTCT	63278	0.15364320482466565	No Hit
CCCGCCCGTACCATATTAGCCGTTTTATTGACAATTCTATAAAGAGCGAT	60759	0.14752690480011793	No Hit
GTTTGATTGAGCATCCAAGCCATTAGCCGTCTAGTTGGTCAGATCTGTAT	56088	0.13618540523097836	No Hit
GTCGTTTGTAGTTCCTTTAAGGTCTTAACAGAAGTTCGGTCGTCTTTGTC	56077	0.13615869649724668	No Hit
GGGCTCATTACAATTGGATGCAAGAACGCAAAGGTGGGCTTGGCGTTTGT	55429	0.13458530927378223	No Hit
CTGGTGCTAAGTGCATGTTGGGCGAAGGTAAGTGGTATGGAGATGATAGT	54311	0.1318707306999655	No Hit
CCCGGTTTGCTGGATCTCTGTGATGGGCTTAATGTCTTTGGCAGCTTTGA	53242	0.12927512739458974	No Hit
CCCGTAGTTCACCTACCAGAACCTTTTCACTGGCTATACAGCAACACTCT	53105	0.12894248225629554	No Hit
GTCCTGAATTAATTTGTGGTTTGATTGAGCATCCAAGCCATTAGCCGTCT	52901	0.1284471566489086	No Hit
GTTTTGGTGTATTCGAGCTGCTTTAATAAACTTCTTTATGAGACATTATA	51059	0.12397465778220874	No Hit
CCTGAATTAATTTGTGGTTTGATTGAGCATCCAAGCCATTAGCCGTCTAG	49482	0.12014559659177133	No Hit
GGGAGTTTCAGTTCTTCAGATTACGCATGATGGTGGAAGTTGCAAGCTTC	47983	0.11650592460415836	No Hit
CCACAACGATCAGTAACAGTACACCGCCCTCCATAGAGGCAATGGTAACA	47710	0.11584306239427286	No Hit
CCTGACTTTCTTCATCCTGTTCTTGCGTTCCTTACGCTGTTTTCTAGAAG	47375	0.11502966004880899	No Hit
GCTCATTACAATTGGATGCAAGAACGCAAAGGTGGGCTTGGCGTTTGTCT	46983	0.11407785790128112	No Hit
CTCATCTTTCTCCTTTTGCGCTTCAGTCTGCGCATACGCTTCTTTCGCCA	46746	0.11350240609269921	No Hit
GGGGAGTTTCAGTTCTTCAGATTACGCATGATGGTGGAAGTTGCAAGCTT	45915	0.11148468266260823	No Hit
CTTGGCGTTTGTCTAAGGCTTTGGCAGCTTCACGAATTCCACGGGCTAAC	45419	0.11028036157798113	No Hit
CCCCATTTGAGTCAATATCAAAGTTGGCCACCACATTGTCCCGAAGAAAC	44215	0.10735696926771694	No Hit
GTGCTGGTGCTAAGTGCATGTTGGGCGAAGGTAAGTGGTATGGAGATGAT	44191	0.10729869566684788	No Hit
GGTGGCTTTTCGGATGTGCATACCCTTAATCGCTTGGGCTGTCTCCCGTG	43537	0.10571074004316616	No Hit
AAGGATTTAAGAAAGGTTCTTATGAGGATGGTAAGGTAGGGTAGGGTAAC	43155	0.10478321856266706	No Hit
CCCTGAGTTAGTTGGGATATACTCCACAAGCTAGAGCTTAATTCCTTCTA	42787	0.10388969001600824	No Hit
GCCTCTGCCTTTTTTCCTCTATACTCCTATCTCTTTAACAGCACTCATTG	42705	0.10369058854637231	No Hit

Sequence	Count	Percentage	Possible Source
CCCTGTCATACCTGCATGCTAGAGTATCAGCTGAGGGTTGAGCGTTTCCT	42620	0.10348420287662774	No Hit
GCCGTCTAGTTGGTCAGATCTGTATCTATTATTGTTGAAGATATCTAGGG	42421	0.10300101760275518	No Hit
GCACGTTTAGATGTGGATAGTGGTGTAAGGATTTAAGAAAGGTTCTTATG	42143	0.1023260150593553	No Hit
CCCGTTGTTTCCCTTAGTATTCAGAATAGCCGACATACTTTATCTATCTT	42083	0.10218033105718266	No Hit

Adapter Content

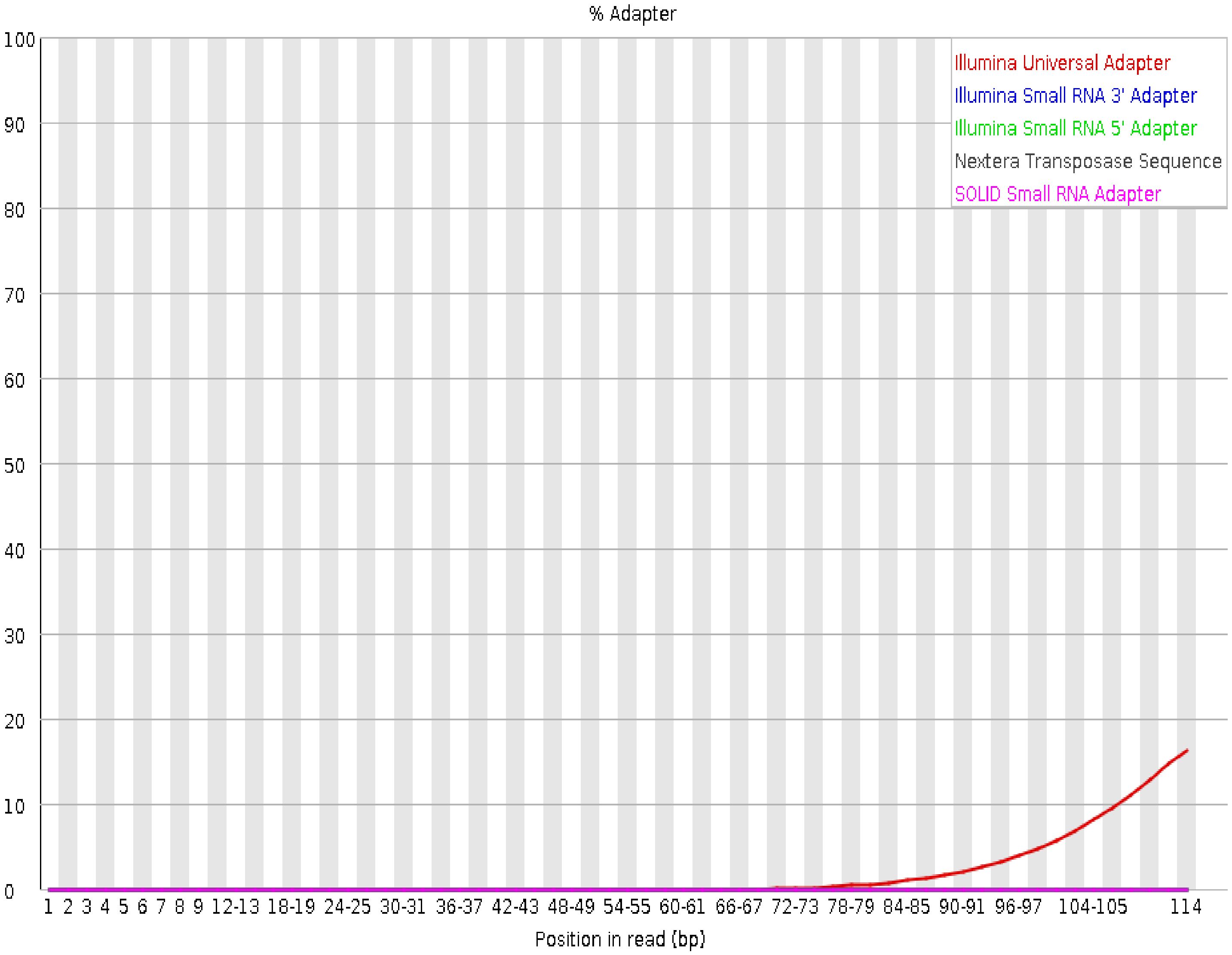

Produced by FastQC (version 0.11.8)

FastQC Report

Wed 9 Oct 2019
Eulamprus.Female.Liver_R2.fastq.gz

Summary

- Basic Statistics
- Per base sequence quality
- Per tile sequence quality
- Per sequence quality scores
- Per base sequence content
- Per sequence GC content
- Per base N content
- Sequence Length Distribution
- Sequence Duplication Levels
- Overrepresented sequences
- Adapter Content

Basic Statistics

Measure	Value
Filename	Eulamprus.Female.Liver_R2.fastq.gz
File type	Conventional base calls
Encoding	Sanger / Illumina 1.9
Total Sequences	41185030
Sequences flagged as poor quality	0
Sequence length	125
%GC	46

Per base sequence quality

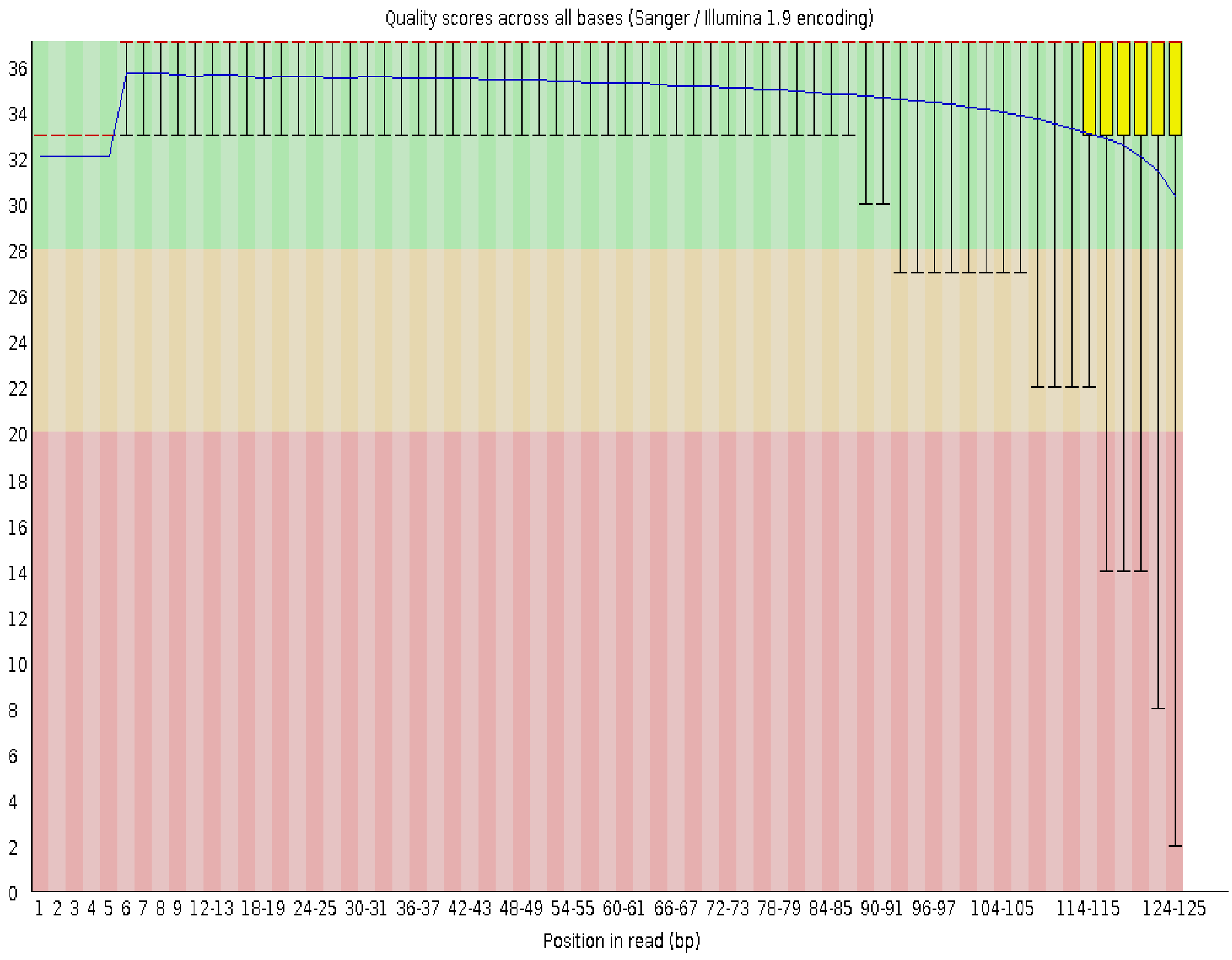

Per tile sequence quality

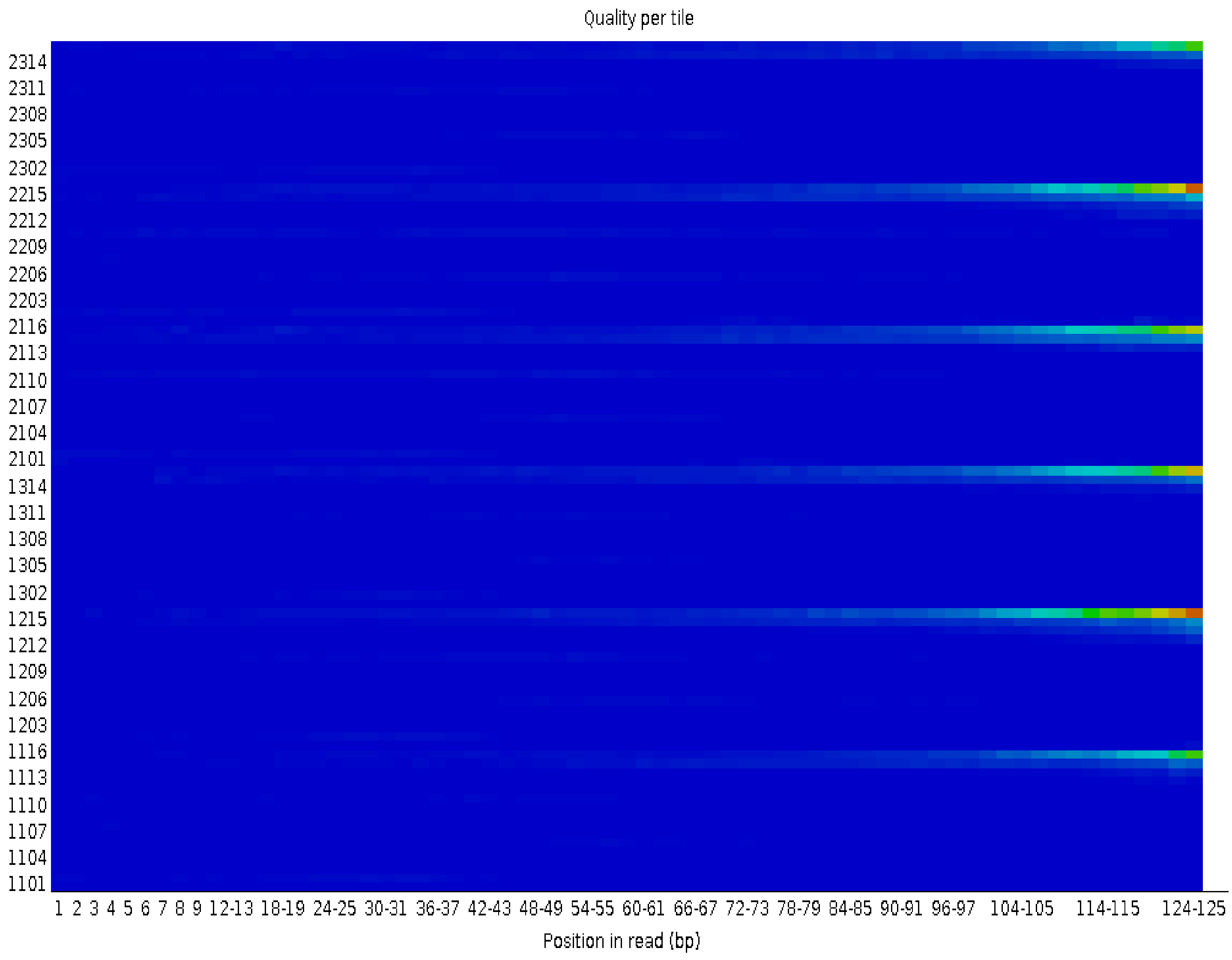

Per sequence quality scores

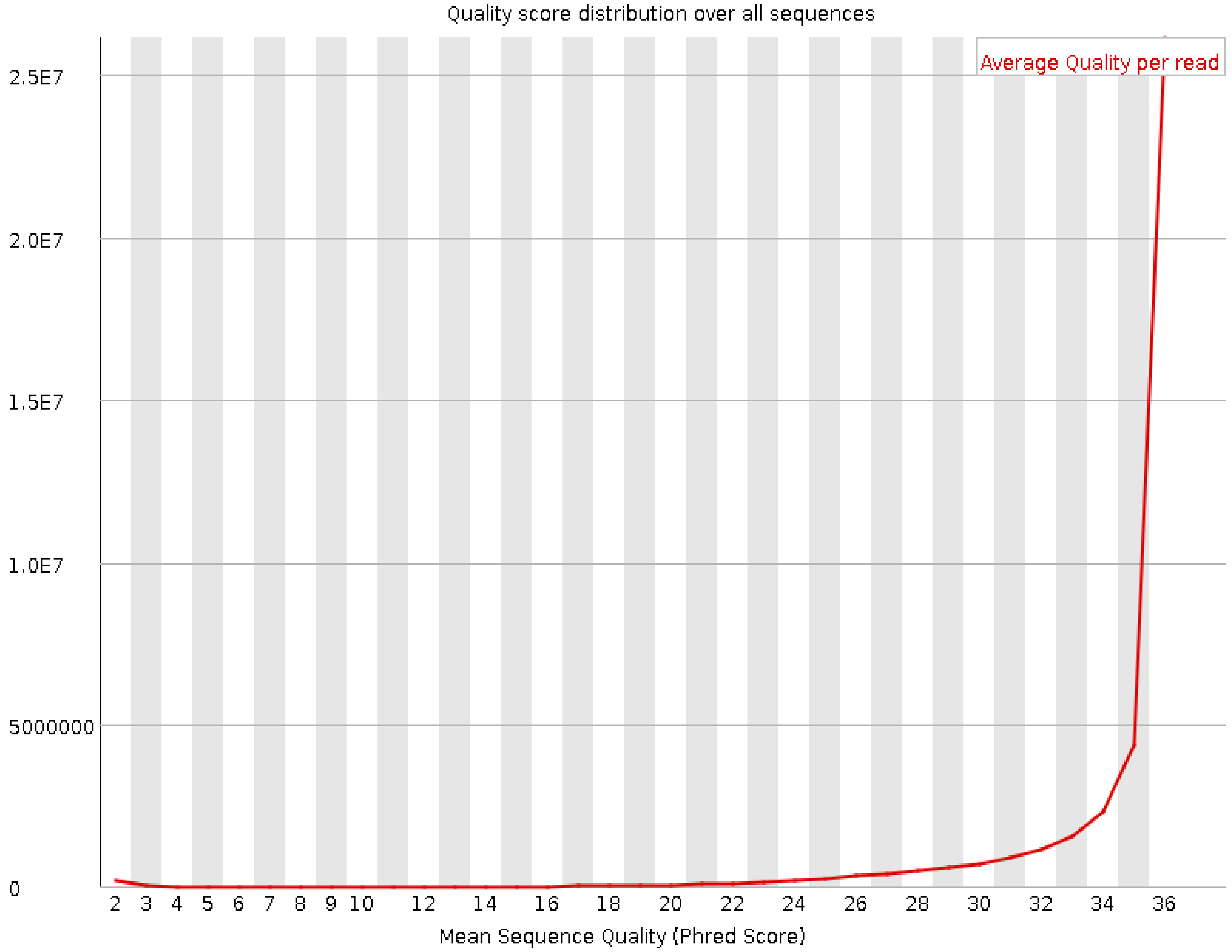

Per base sequence content

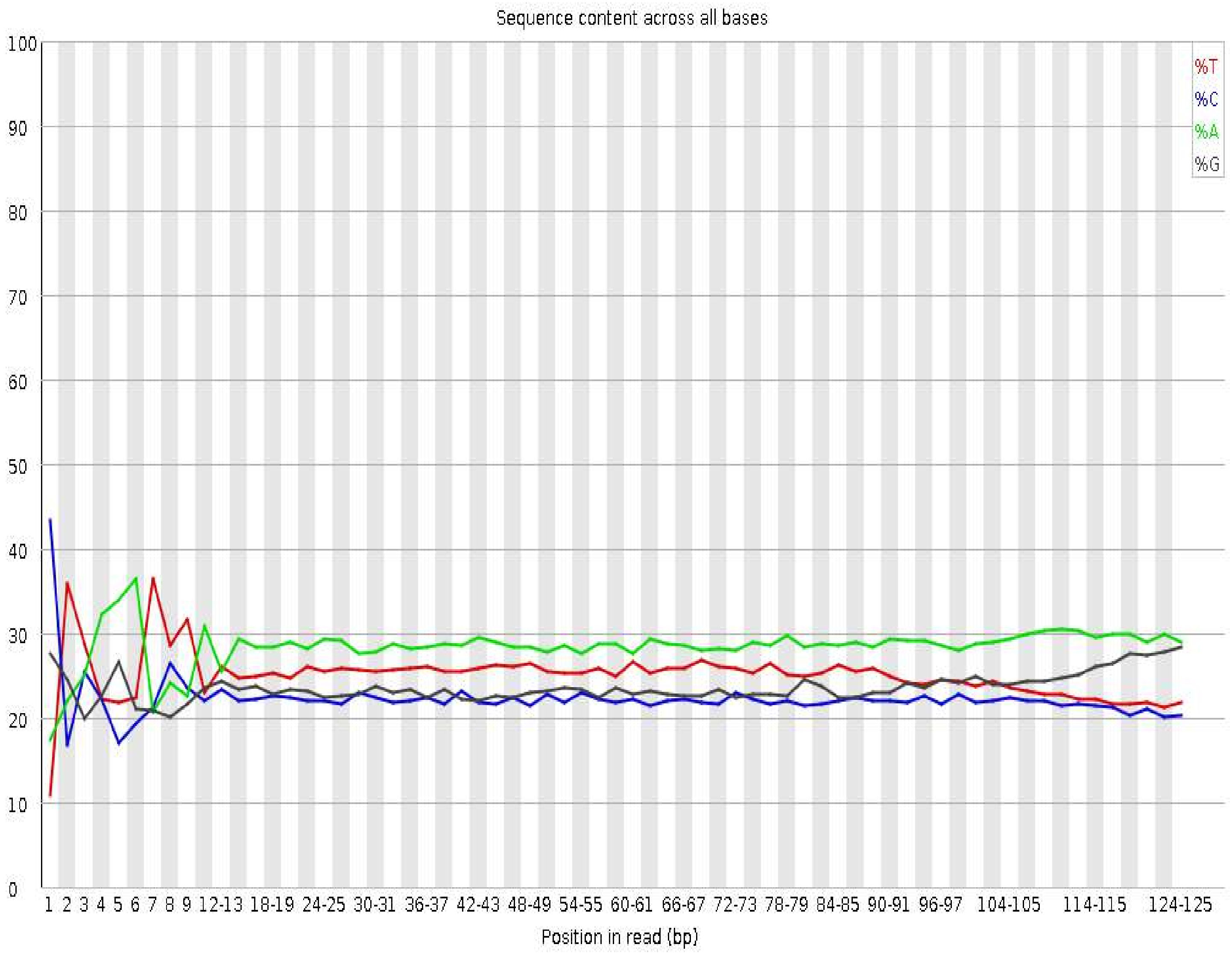

Per sequence GC content

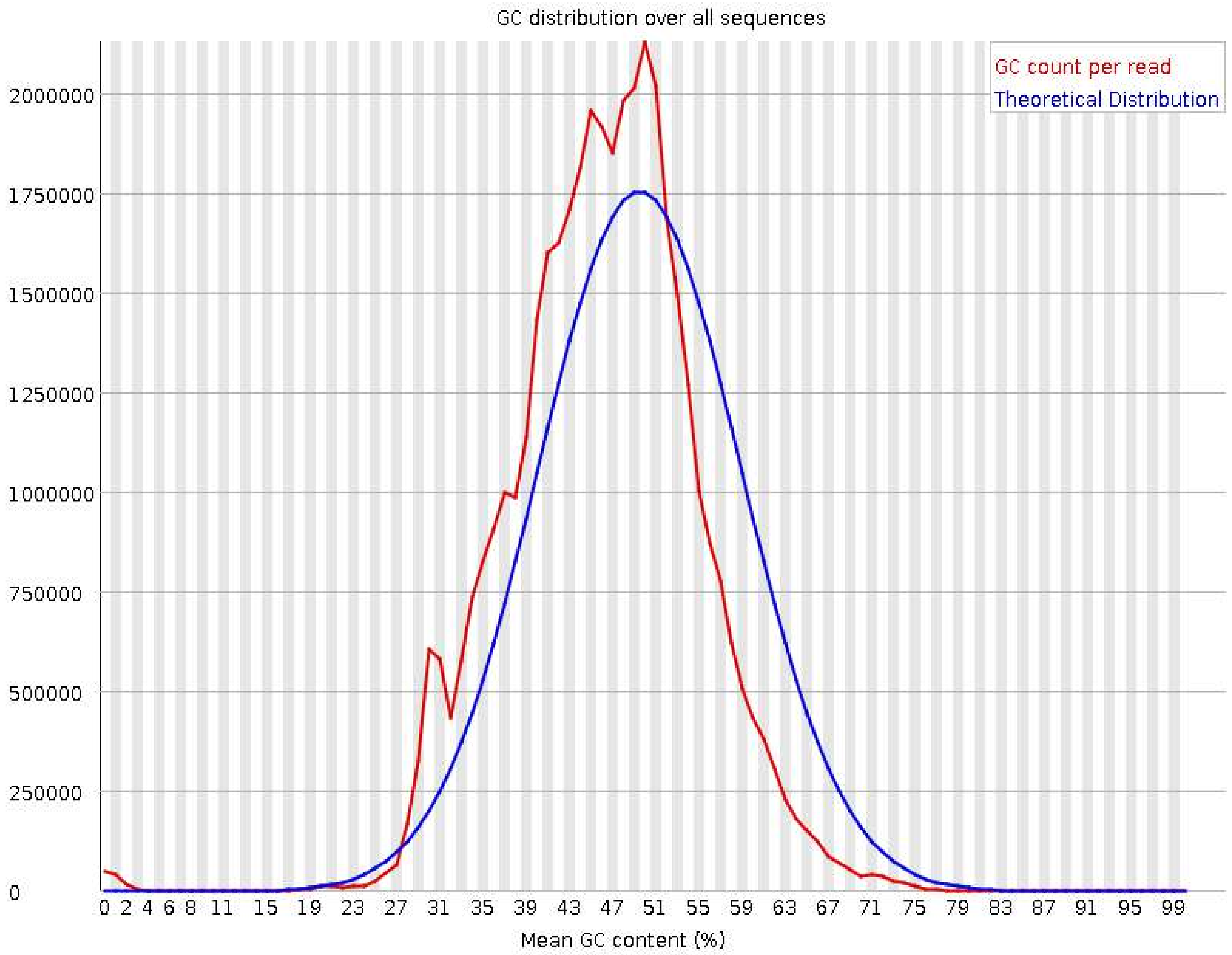

Per base N content

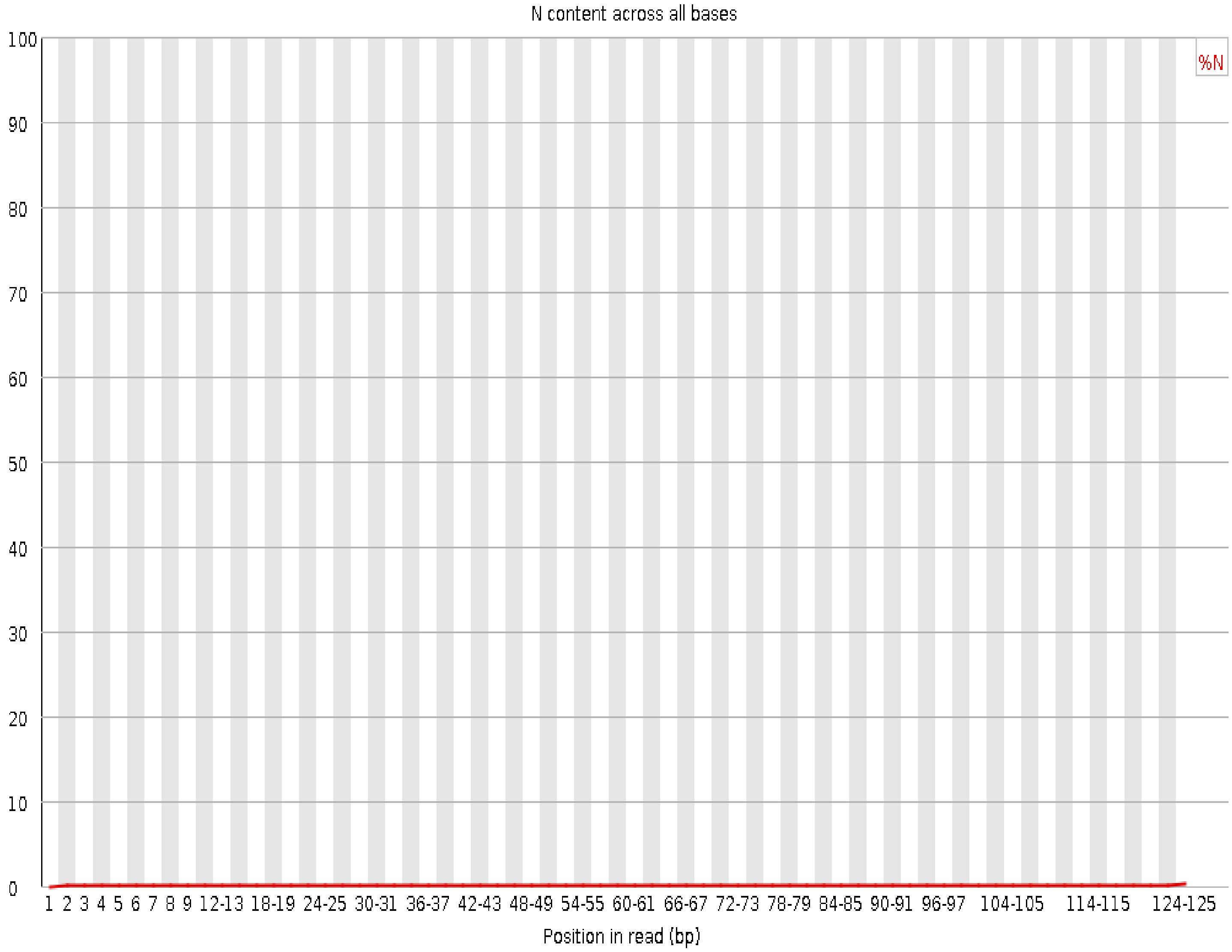

Sequence Length Distribution

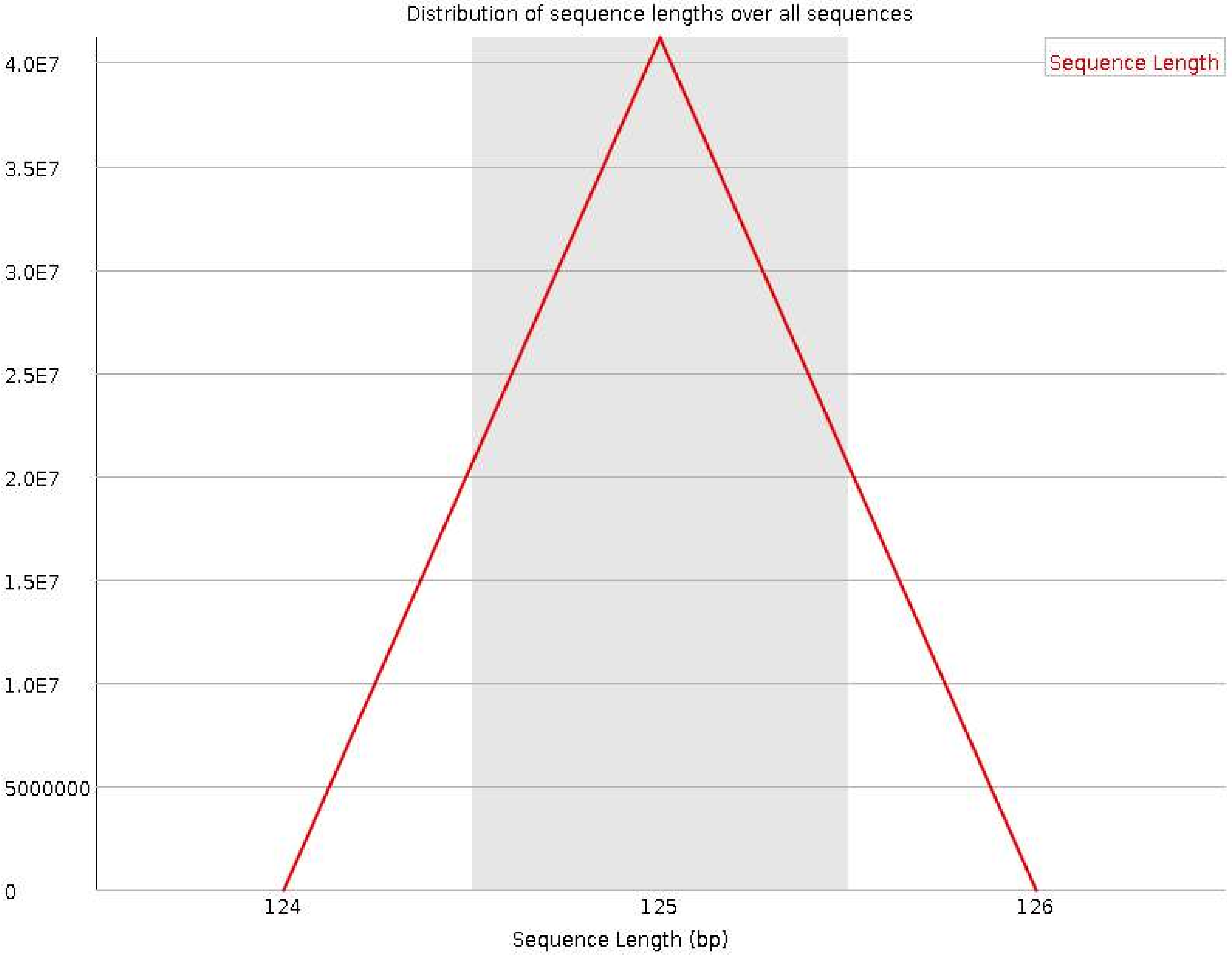

Sequence Duplication Levels

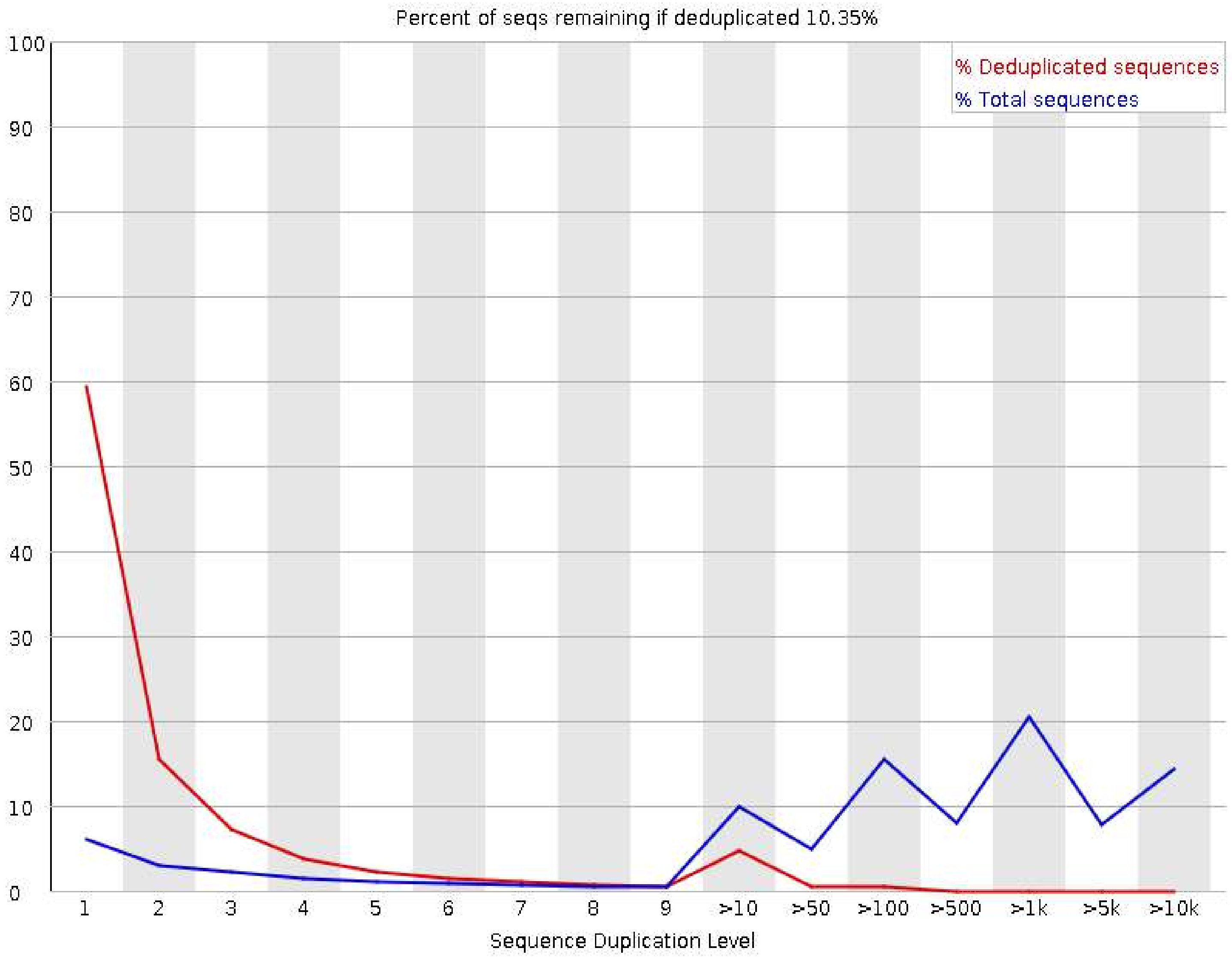

Overrepresented sequences

Sequence	Count	Percentage	Possible Source
CTCAGTTCTCTTGAAACCTACATAAATTCGCAATTATGACAATATATTAC	527921	1.2818274018496527	No Hit
CTTCAACAATAATAGATACAGATCTGACCAACTAGACGGCTAATGGCTTG	165965	0.40297409034302023	No Hit
GTGGACGTTCGGAGGTAAGCTCTGAAAATGGTCCGCTACTCACTTGATCC	98306	0.23869352529304944	No Hit
GTTCTCTTGAAACCTACATAAATTCGCAATTATGACAATATATTACTAGC	92348	0.22422710387730688	No Hit
CTGAACTACATAAGGCTAAGTACATTCCAGGTCATACACAAAAATGAGGA	80010	0.19426961689720756	No Hit
CTTTTATATTGTTCAACTTCTCTCTTAATAGCTATGCACTCACTCCCTAG	73524	0.17852117626234582	No Hit
AGTGGACGTTCGGAGGTAAGCTCTGAAAATGGTCCGCTACTCACTTGATC	72339	0.17564391721943629	No Hit
CGAAGAGTTCCTGAAAGGCATCGGCTTGCCAGATGACCTCATCAAAGCTG	68493	0.16630557268017043	No Hit
CTTGAAACCTACATAAATTCGCAATTATGACAATATATTACTAGCTAAAG	59448	0.14434370935264587	No Hit
GGACGTTCGGAGGTAAGCTCTGAAAATGGTCCGCTACTCACTTGATCCAG	57534	0.13969638968333883	No Hit
GTCGATTTTCACAATATACTTTTATATTGTTCAACTTCTCTCTTAATAGC	54889	0.13327415325422853	No Hit
GTCTAGTTCTTTATATAATCACATTCTTTTTCGTCCTTTATATACGCTAC	51972	0.12619148268193564	No Hit

Sequence	Count	Percentage	Possible Source
GGTCCACTCAGTTCTCTTGAAACCTACATAAATTCGCAATTATGACAATA	50153	0.12177482934940195	No Hit
GGGCTGCCCATTTCGTCGTTTCCCAGGCTCCCAACATGGCATTCAATGGC	47997	0.11653991753799862	No Hit
GCCCATTTCGTCGTTTCCCAGGCTCCCAACATGGCATTCAATGGCATGTG	45892	0.11142883712844205	No Hit
GTTCTTTATATAATCACATTCTTTTTCGTCCTTTATATACGCTACCGTGC	45712	0.11099178512192416	No Hit
CTTTAATCTACATCTACTGATTGCAAATCAGACACTTTAATTAAGCTAAA	44214	0.10735454120101406	No Hit
CAGTTCTCTTGAAACCTACATAAATTCGCAATTATGACAATATATTACTA	42893	0.10414706508651322	No Hit
CTCGTTTACATCATCAAAGAATAGGGGTACTTCCTATTGAAGAGCTGTAT	41201	0.10003877622524494	No Hit

Adapter Content

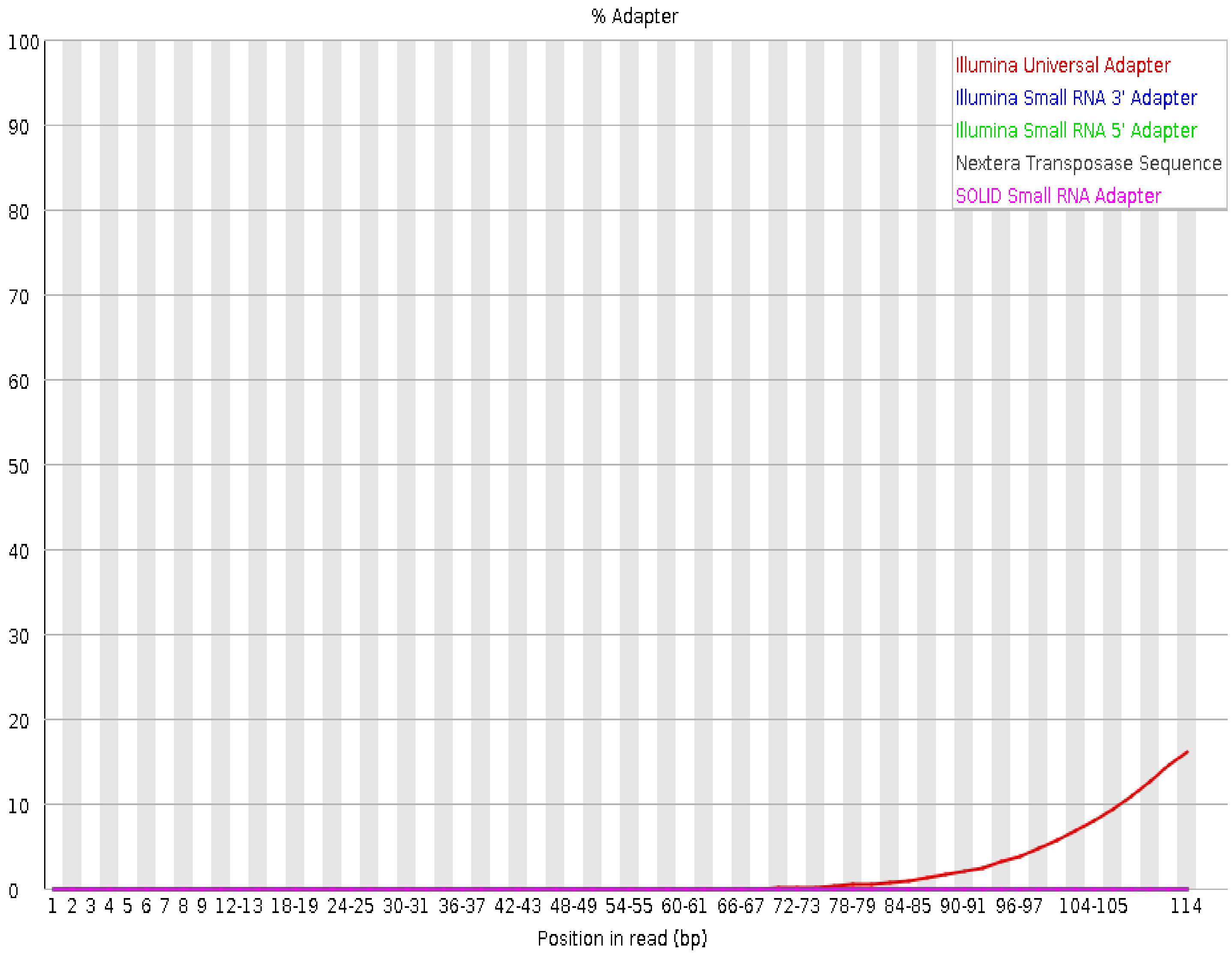

Produced by FastQC (version 0.11.8)

FastQC Report

Wed 9 Oct 2019
Eulamprus.Female.Ovary_R1.fastq.gz

Summary

- Basic Statistics
- Per base sequence quality
- Per tile sequence quality
- Per sequence quality scores
- Per base sequence content
- Per sequence GC content
- Per base N content
- Sequence Length Distribution
- Sequence Duplication Levels
- Overrepresented sequences
- Adapter Content

Basic Statistics

Measure	Value
Filename	Eulamprus.Female.Ovary_R1.fastq.gz
File type	Conventional base calls
Encoding	Sanger / Illumina 1.9
Total Sequences	47862449
Sequences flagged as poor quality	0
Sequence length	125
%GC	47

Per base sequence quality

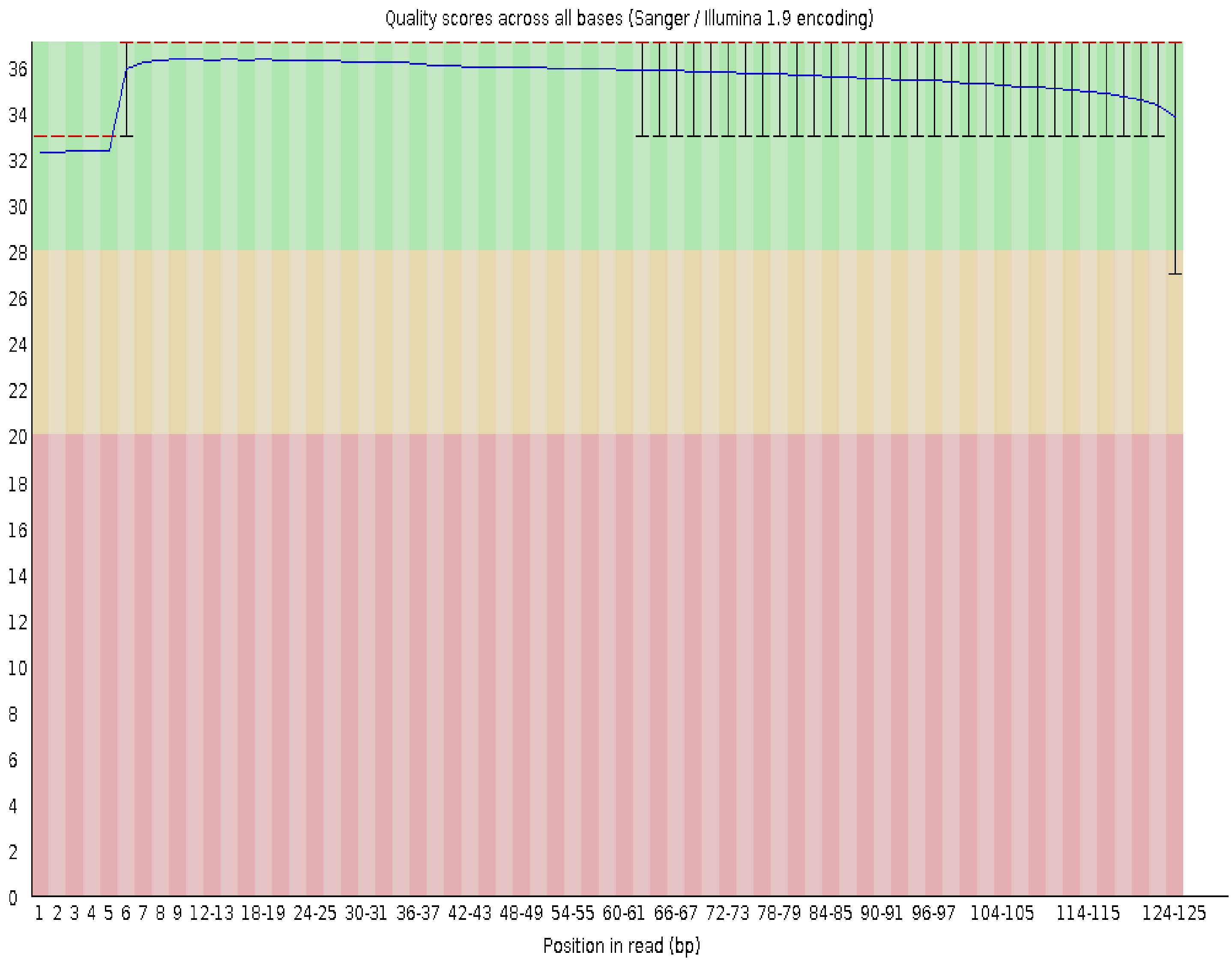

Per tile sequence quality

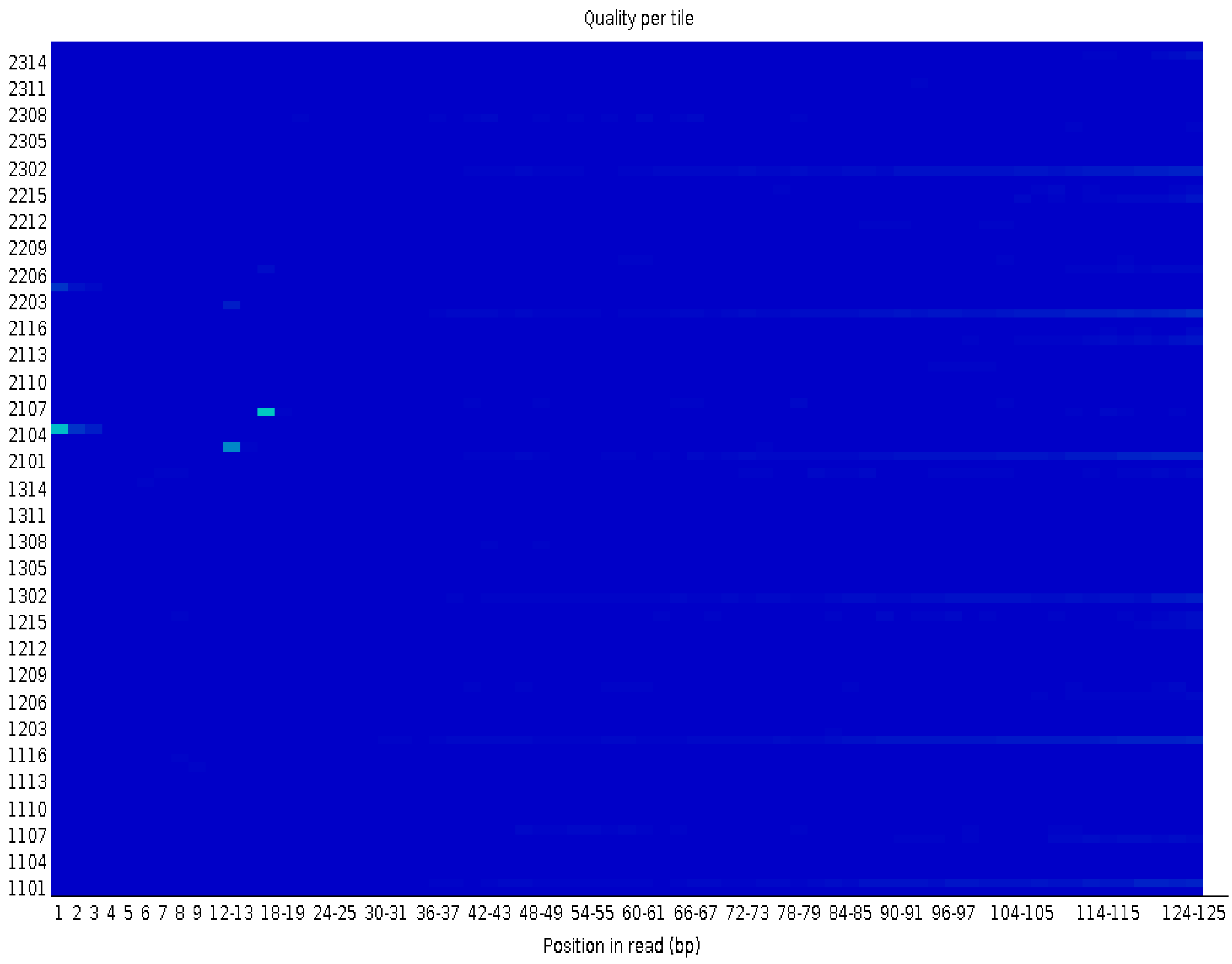

Per sequence quality scores

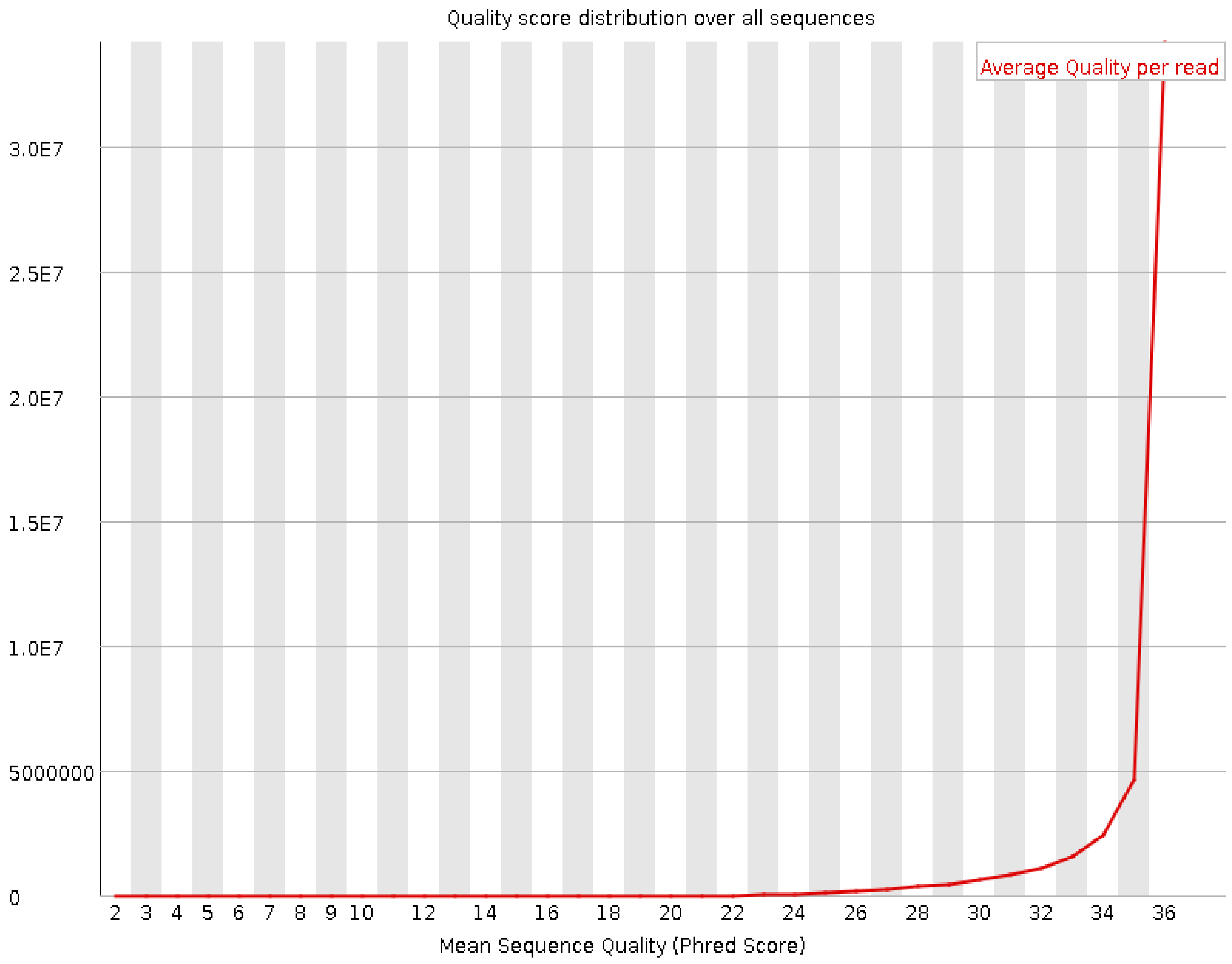

Per base sequence content

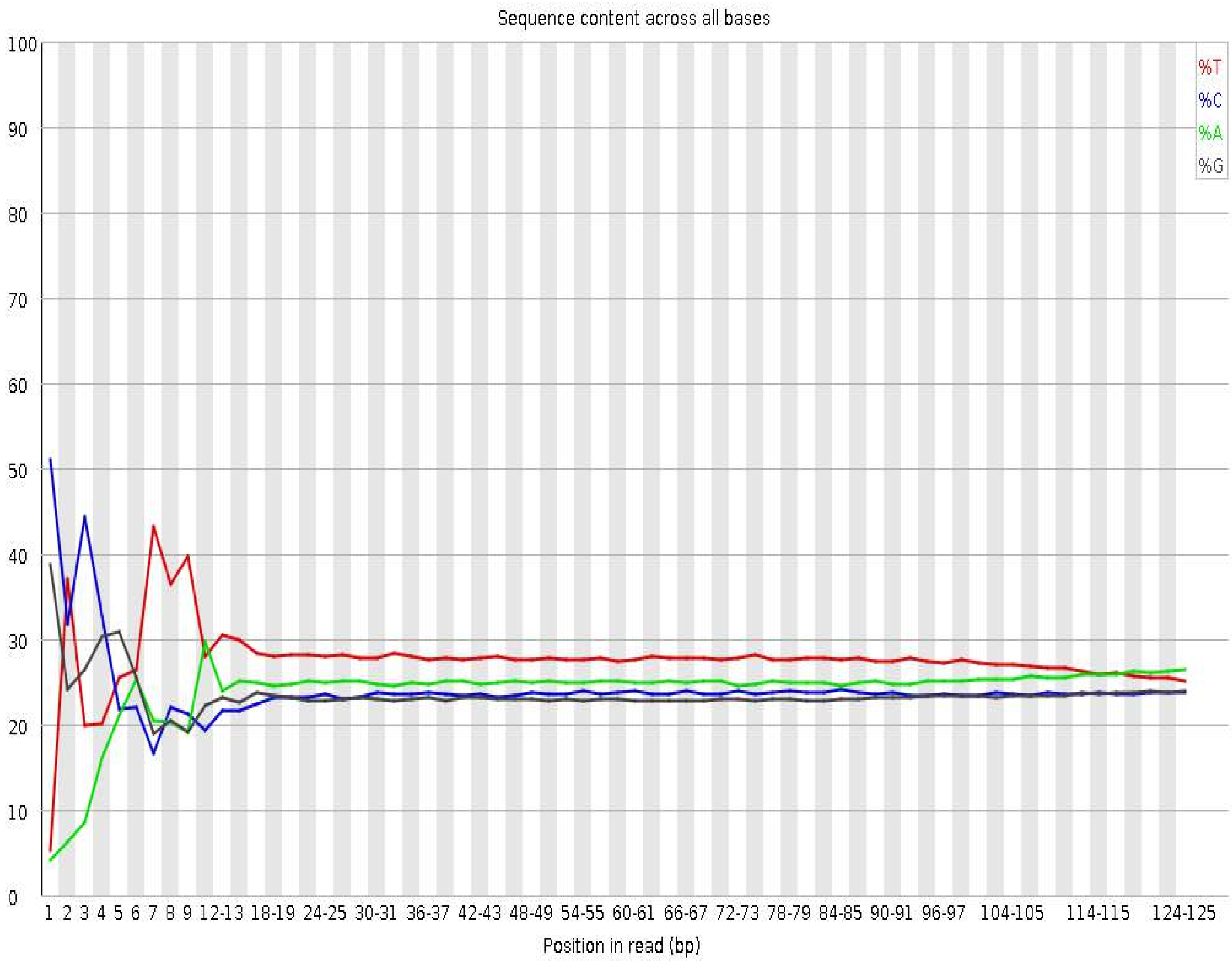

Per sequence GC content

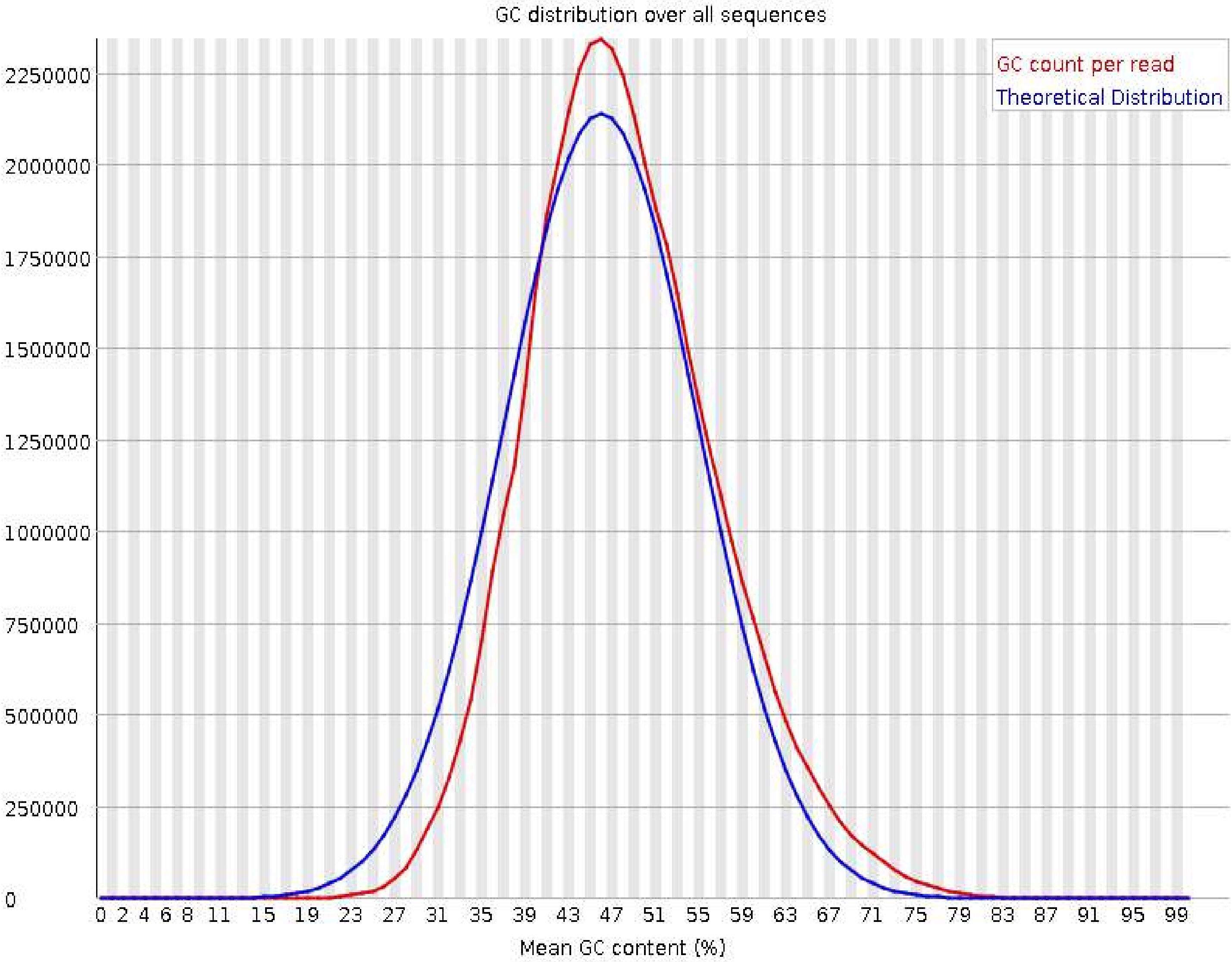

Per base N content

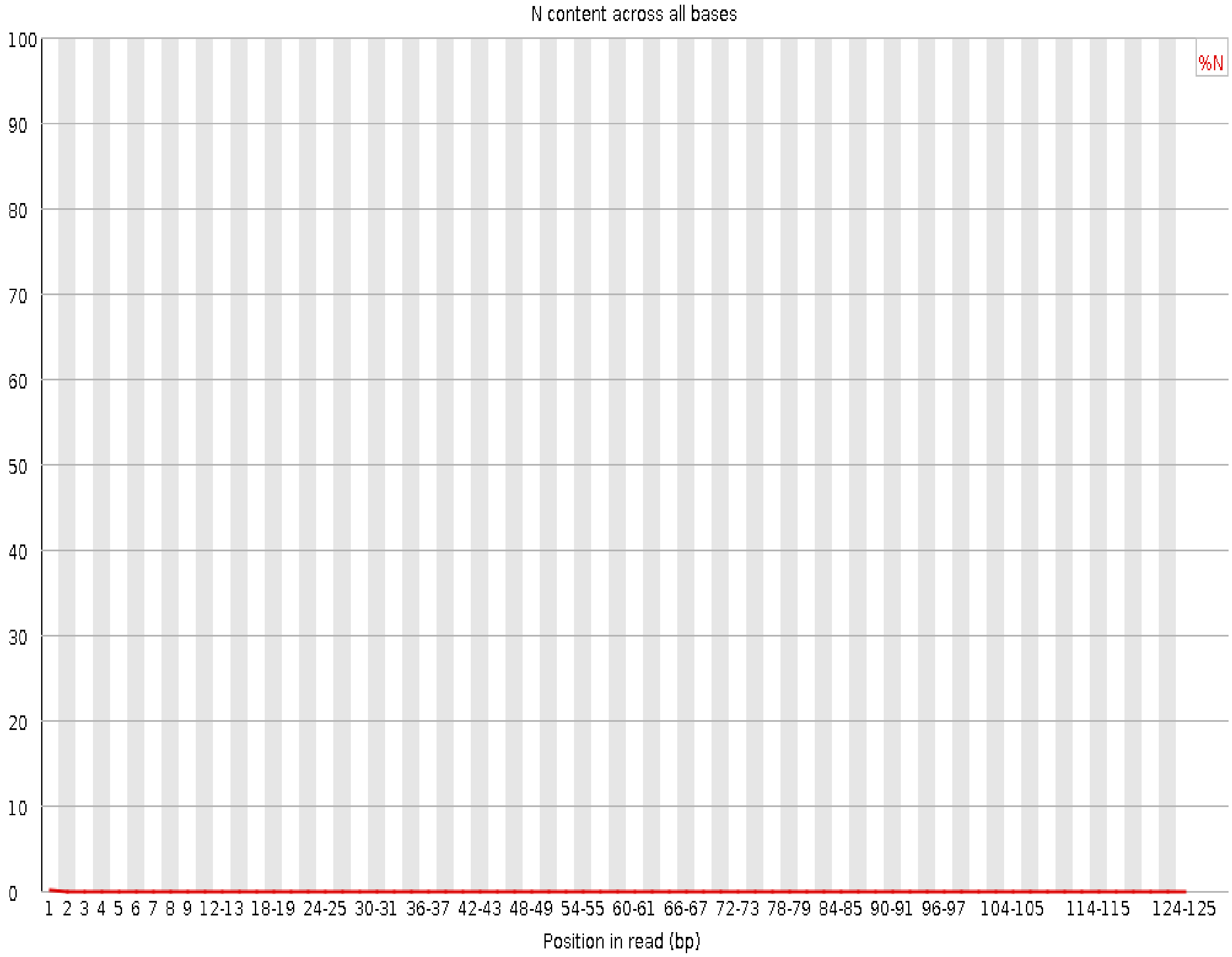

Sequence Length Distribution

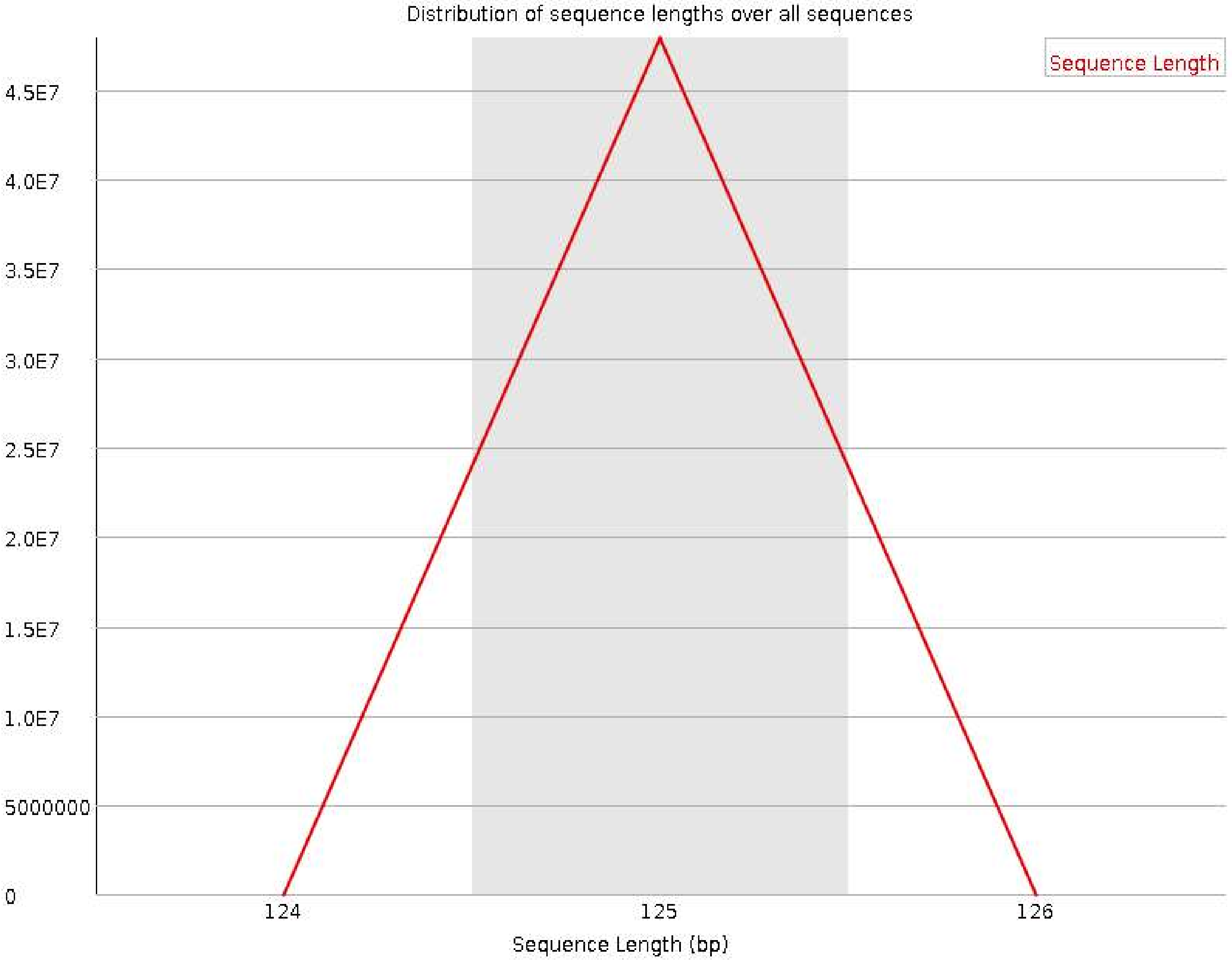

Sequence Duplication Levels

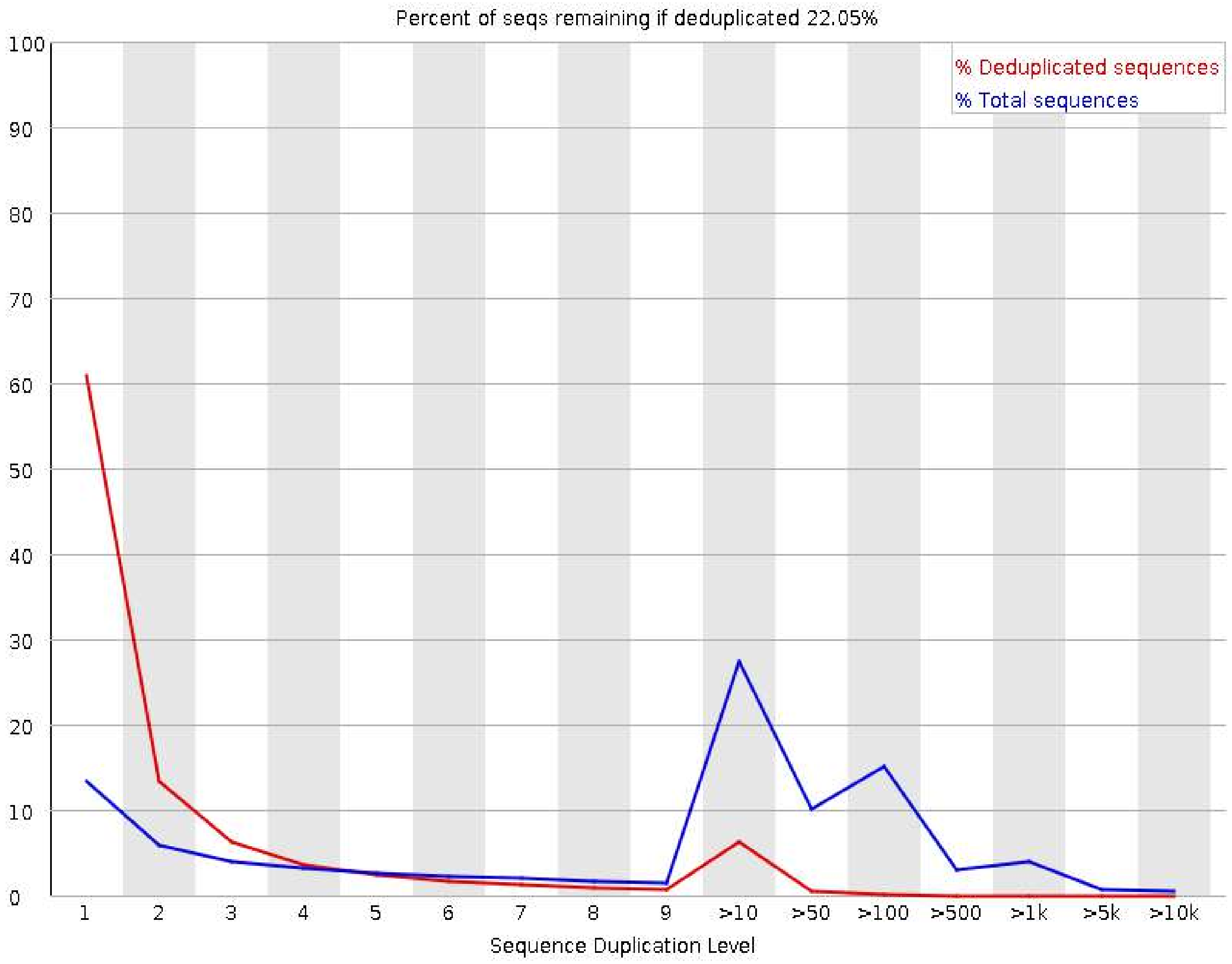

Overrepresented sequences

No overrepresented sequences

Adapter Content

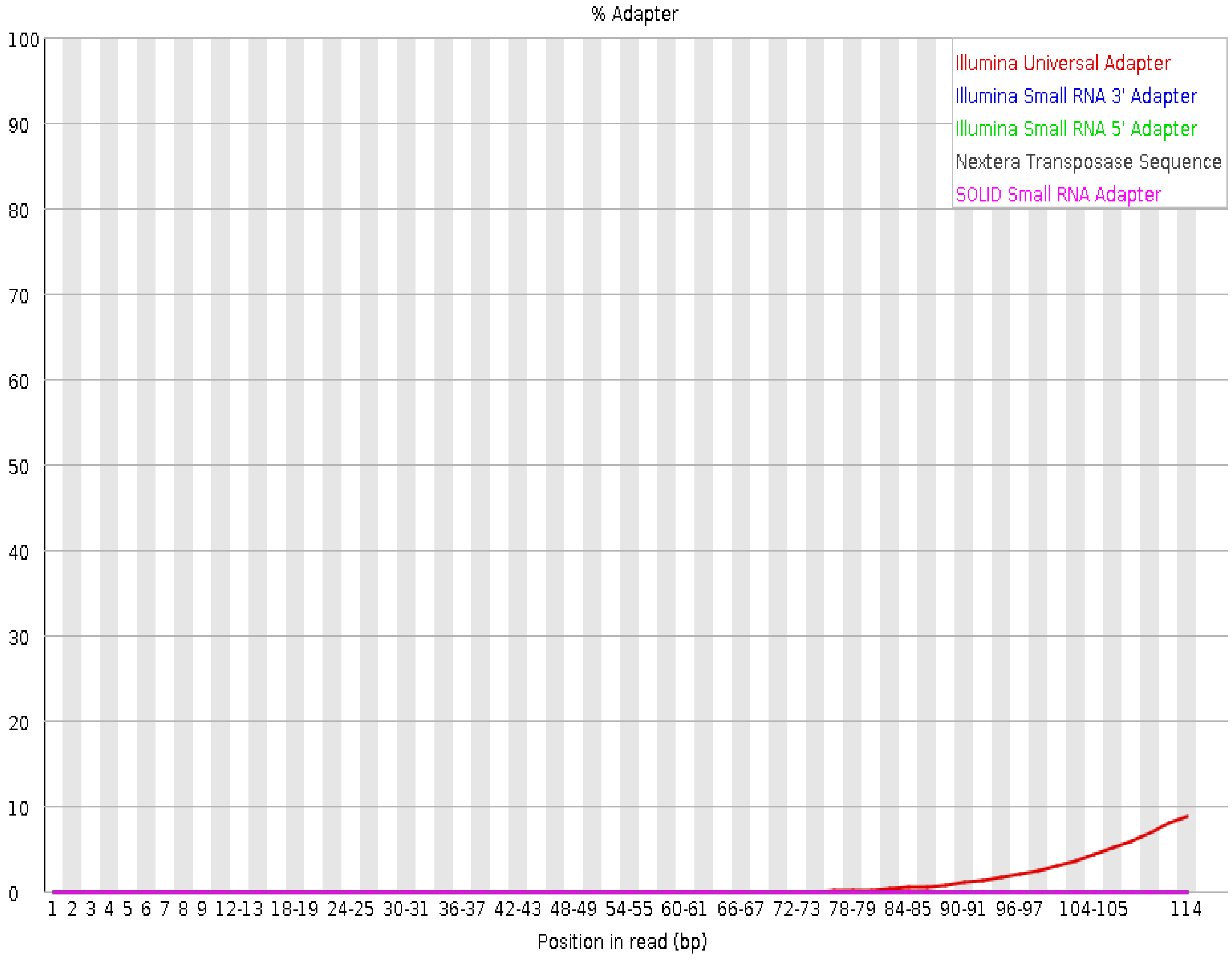

Produced by FastQC (version 0.11.8)

FastQC Report

Wed 9 Oct 2019
Eulamprus.Female.Ovary_R2.fastq.gz

Summary

- Basic Statistics
- Per base sequence quality
- Per tile sequence quality
- Per sequence quality scores
- Per base sequence content
- Per sequence GC content
- Per base N content
- Sequence Length Distribution
- Sequence Duplication Levels
- Overrepresented sequences
- Adapter Content

Basic Statistics

Measure	Value
Filename	Eulamprus.Female.Ovary_R2.fastq.gz
File type	Conventional base calls
Encoding	Sanger / Illumina 1.9
Total Sequences	47862449
Sequences flagged as poor quality	0
Sequence length	125
%GC	47

Per base sequence quality

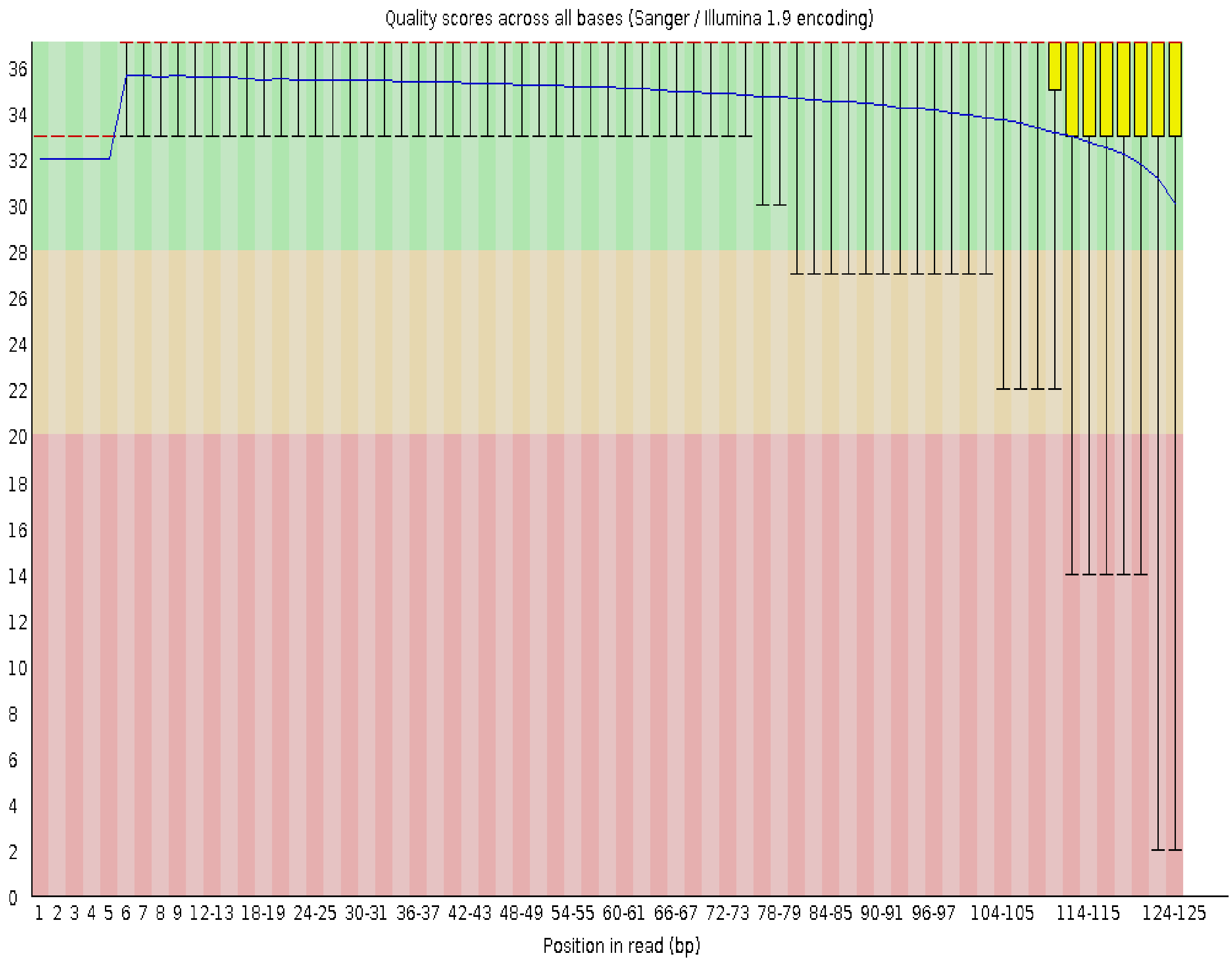

Per tile sequence quality

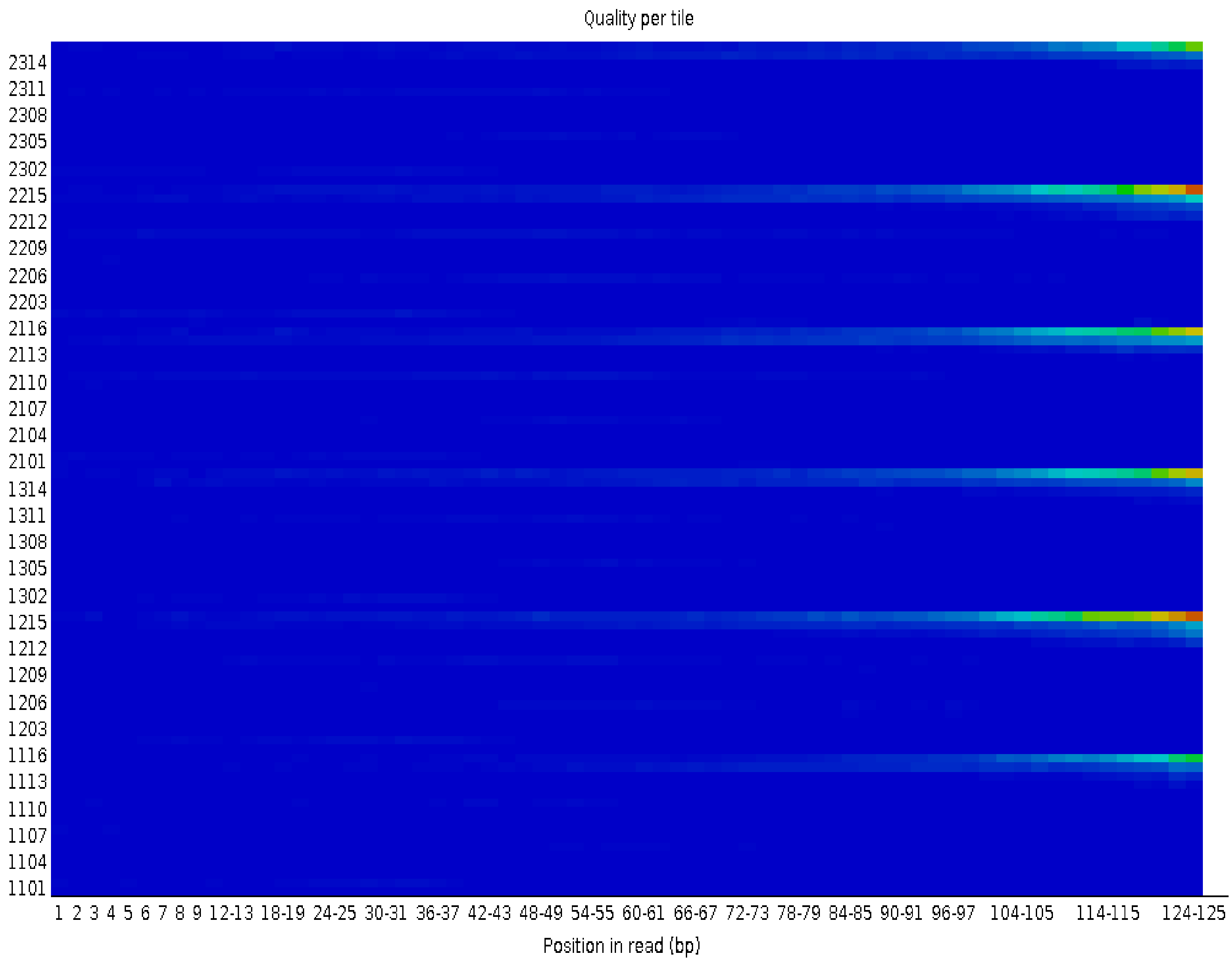

Per sequence quality scores

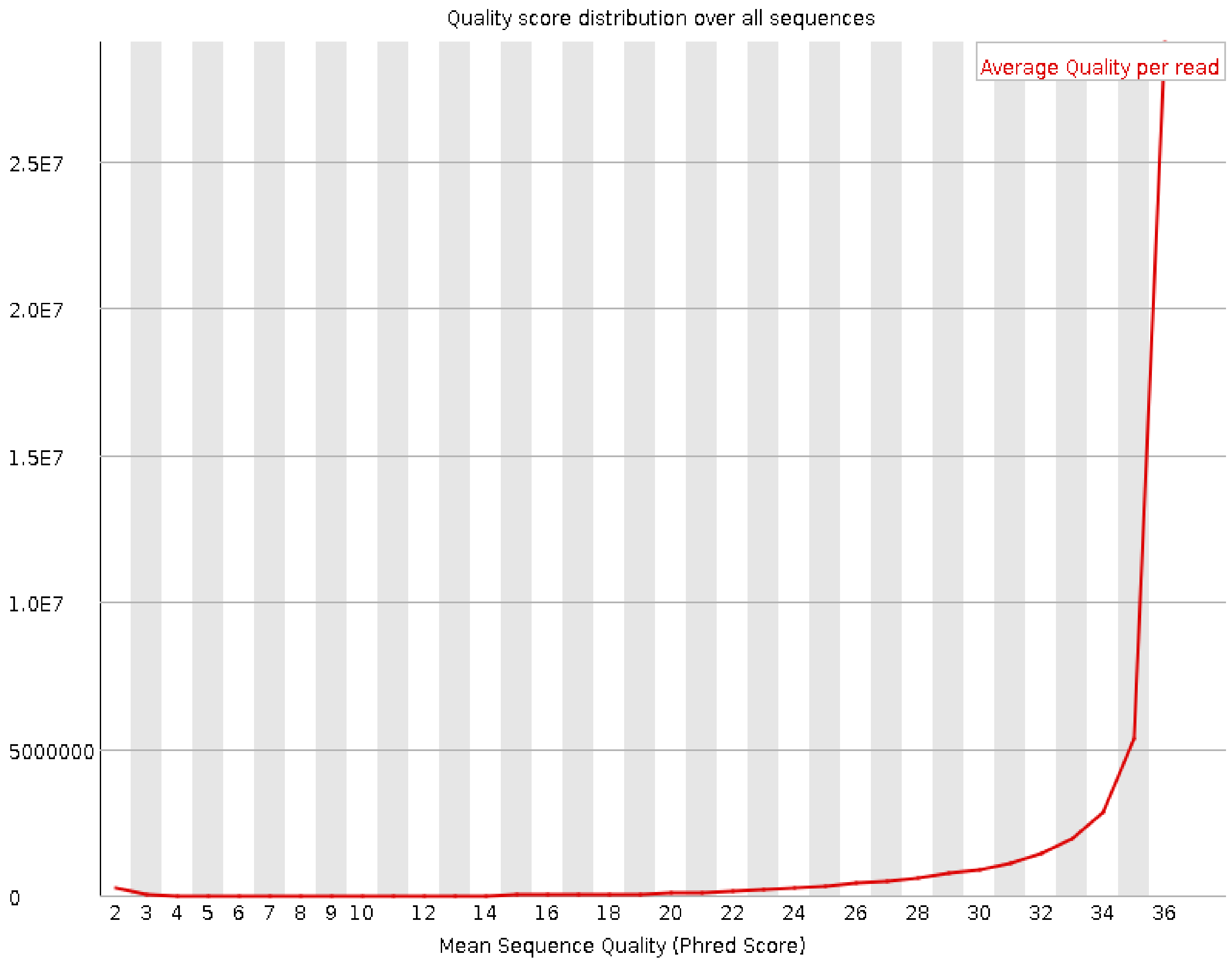

Per base sequence content

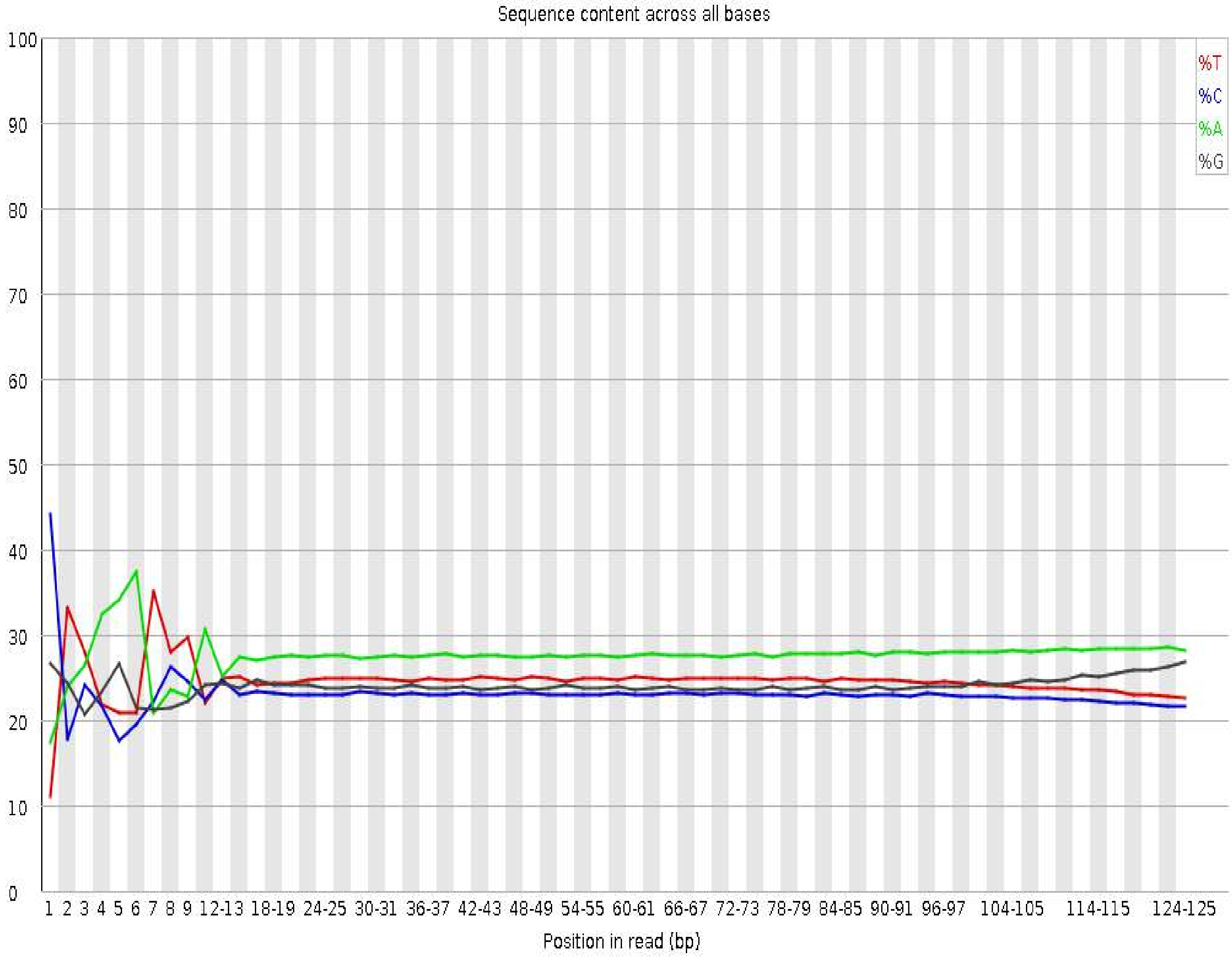

Per sequence GC content

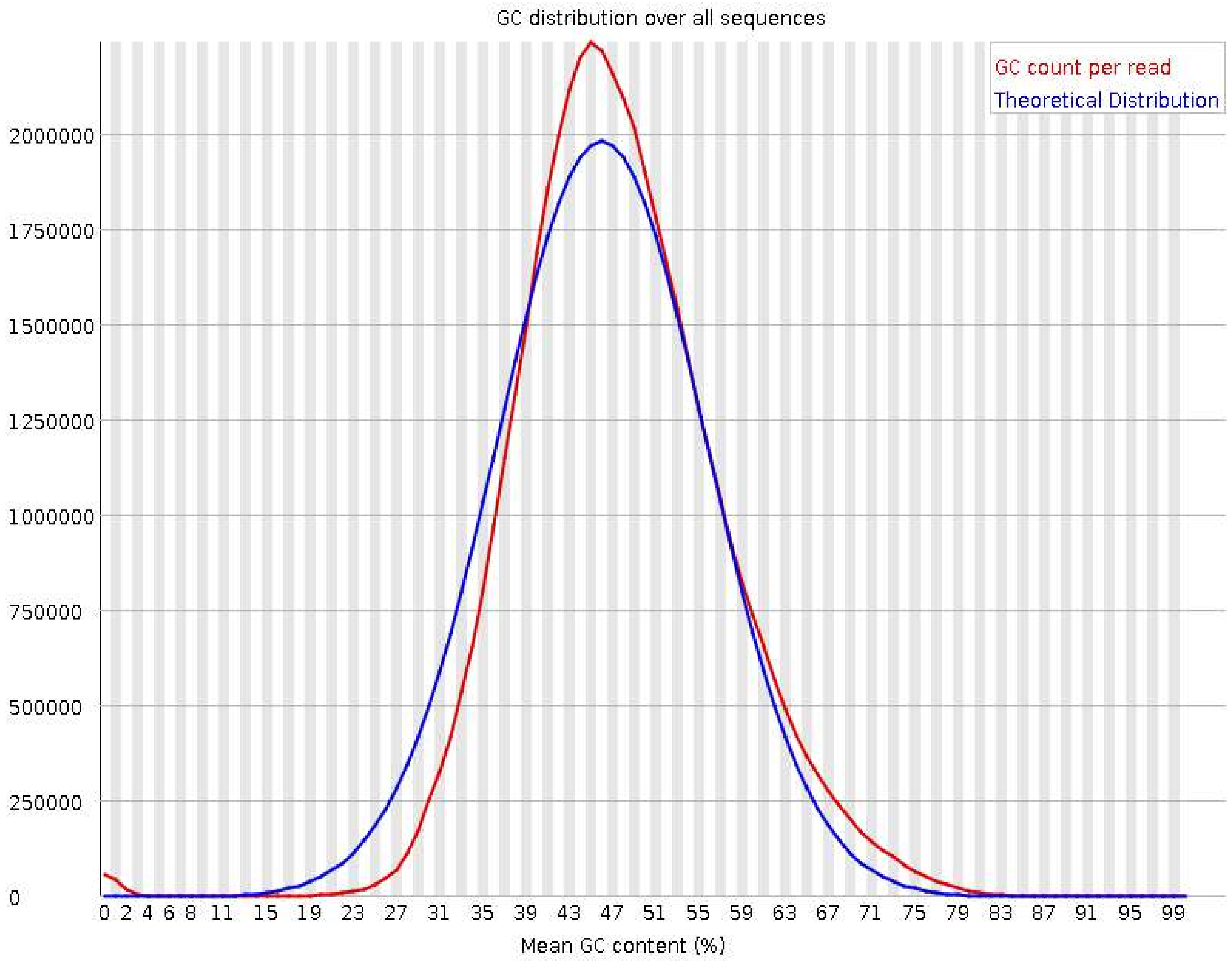

Per base N content

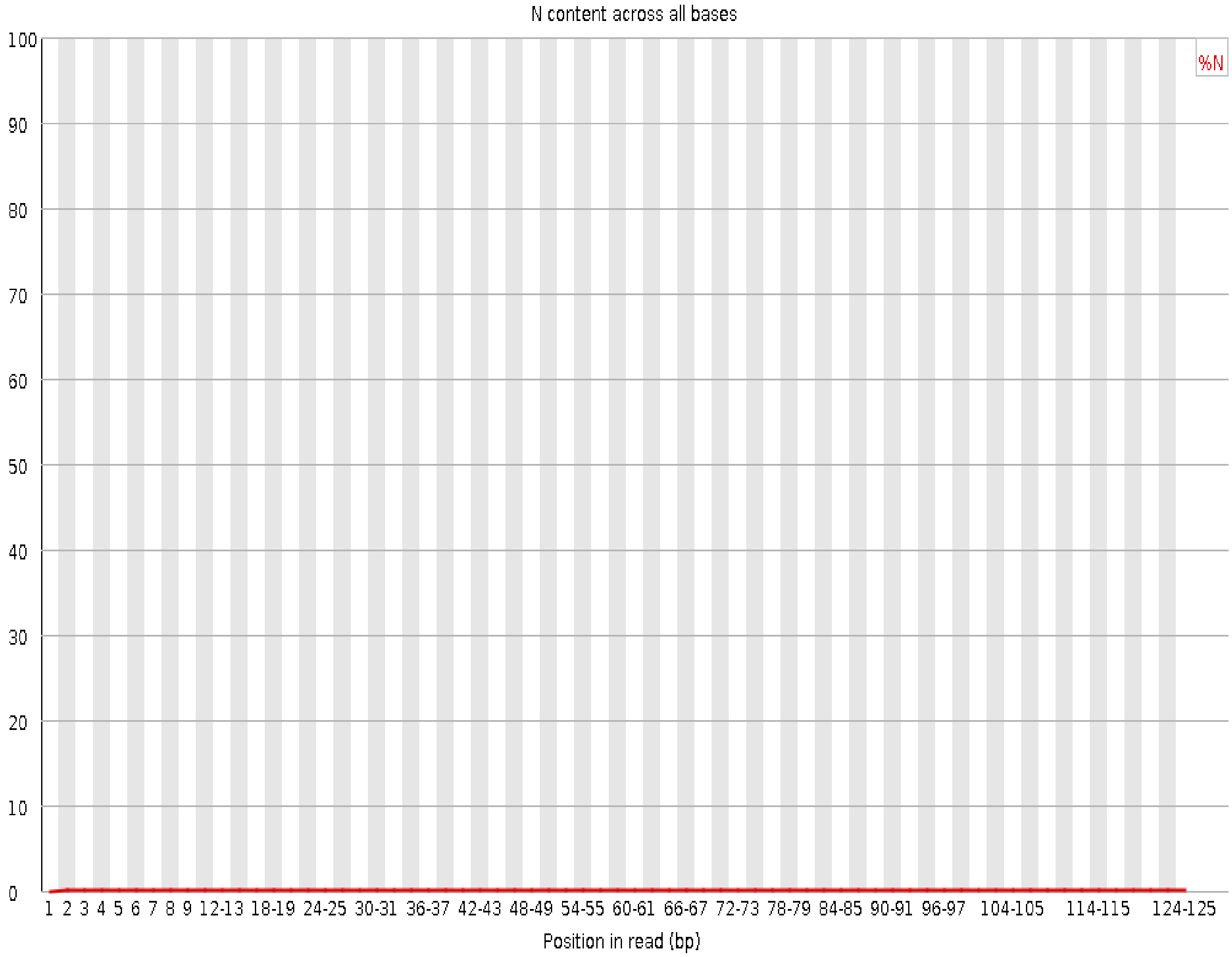

Sequence Length Distribution

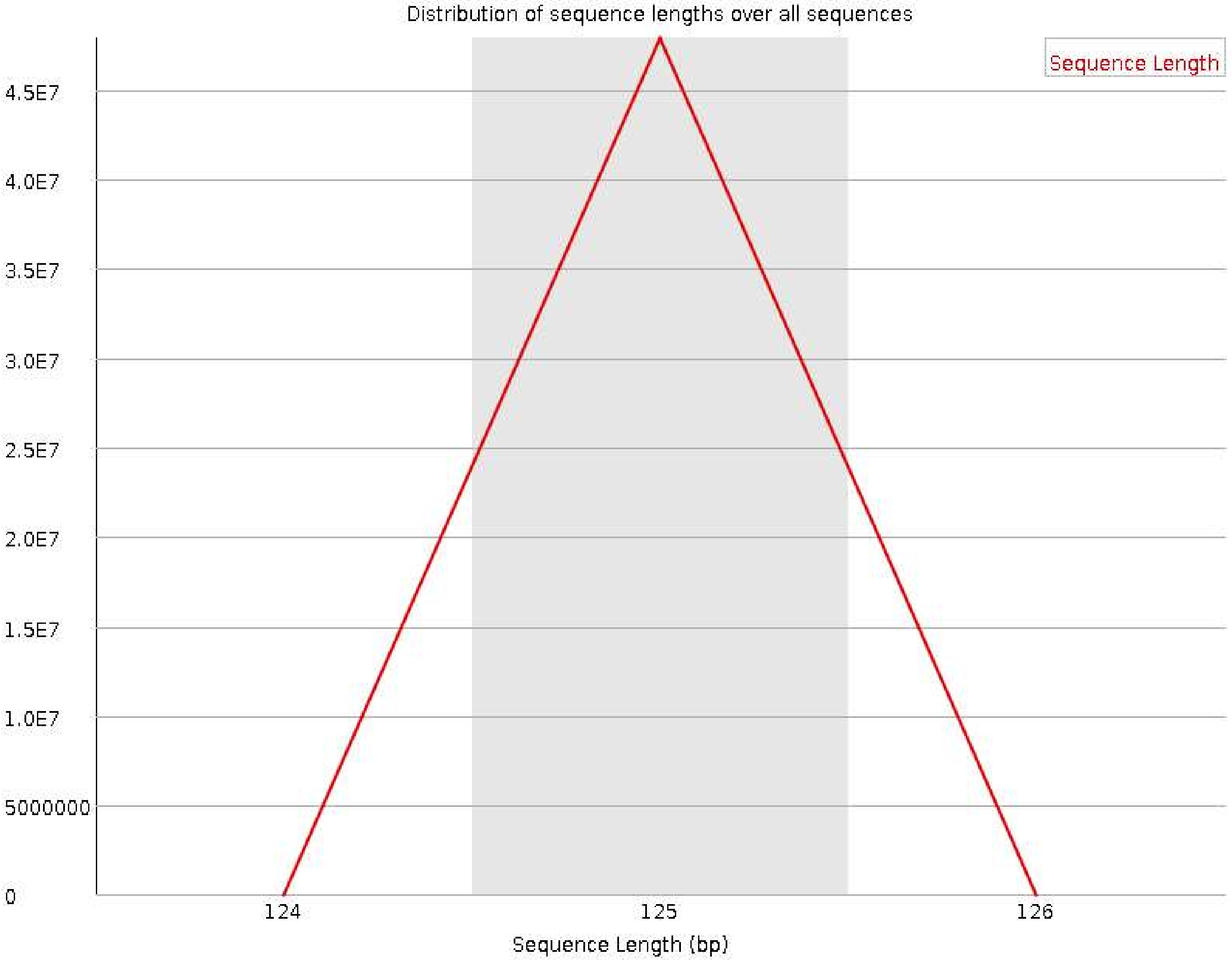

Sequence Duplication Levels

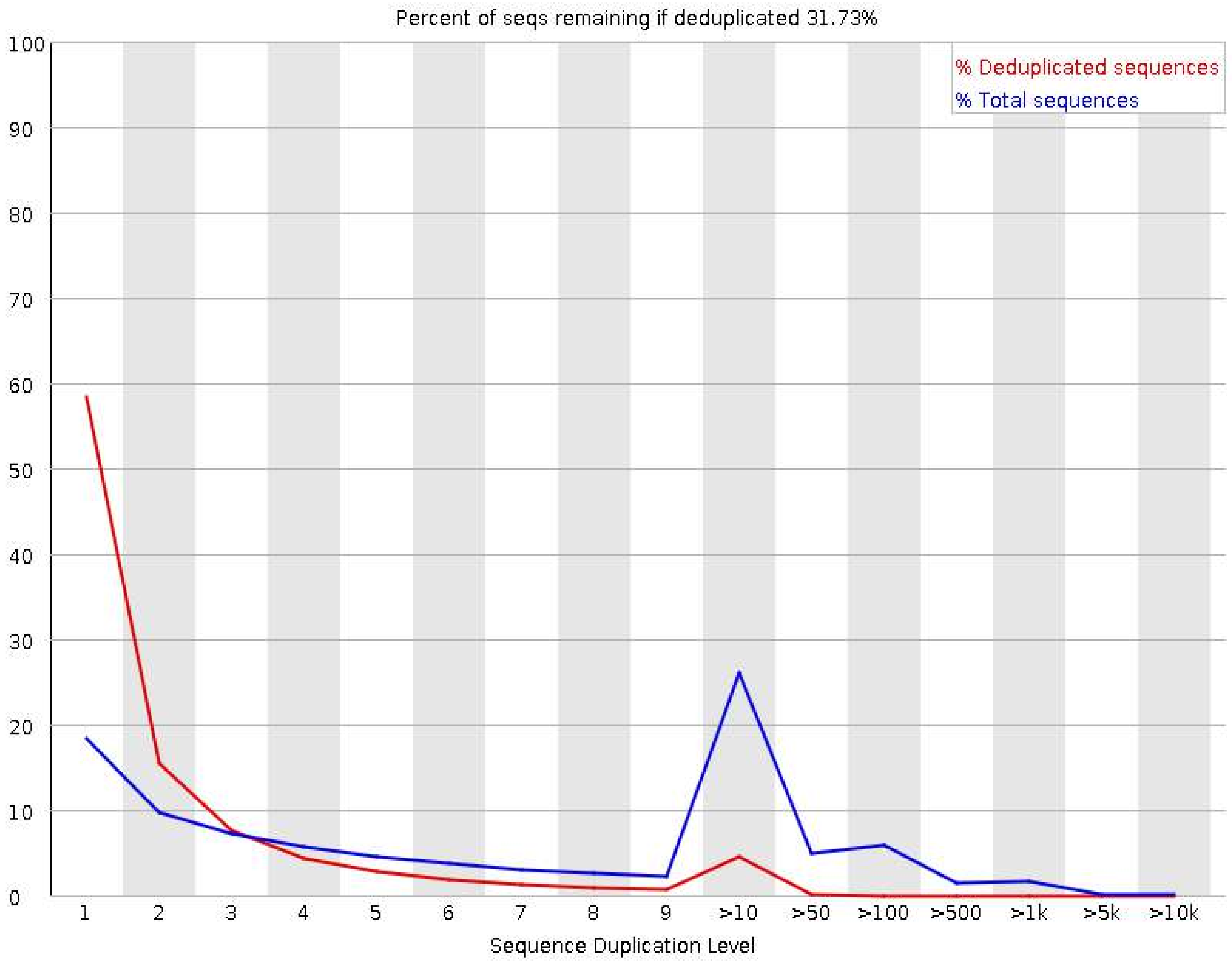

Overrepresented sequences

No overrepresented sequences

Adapter Content

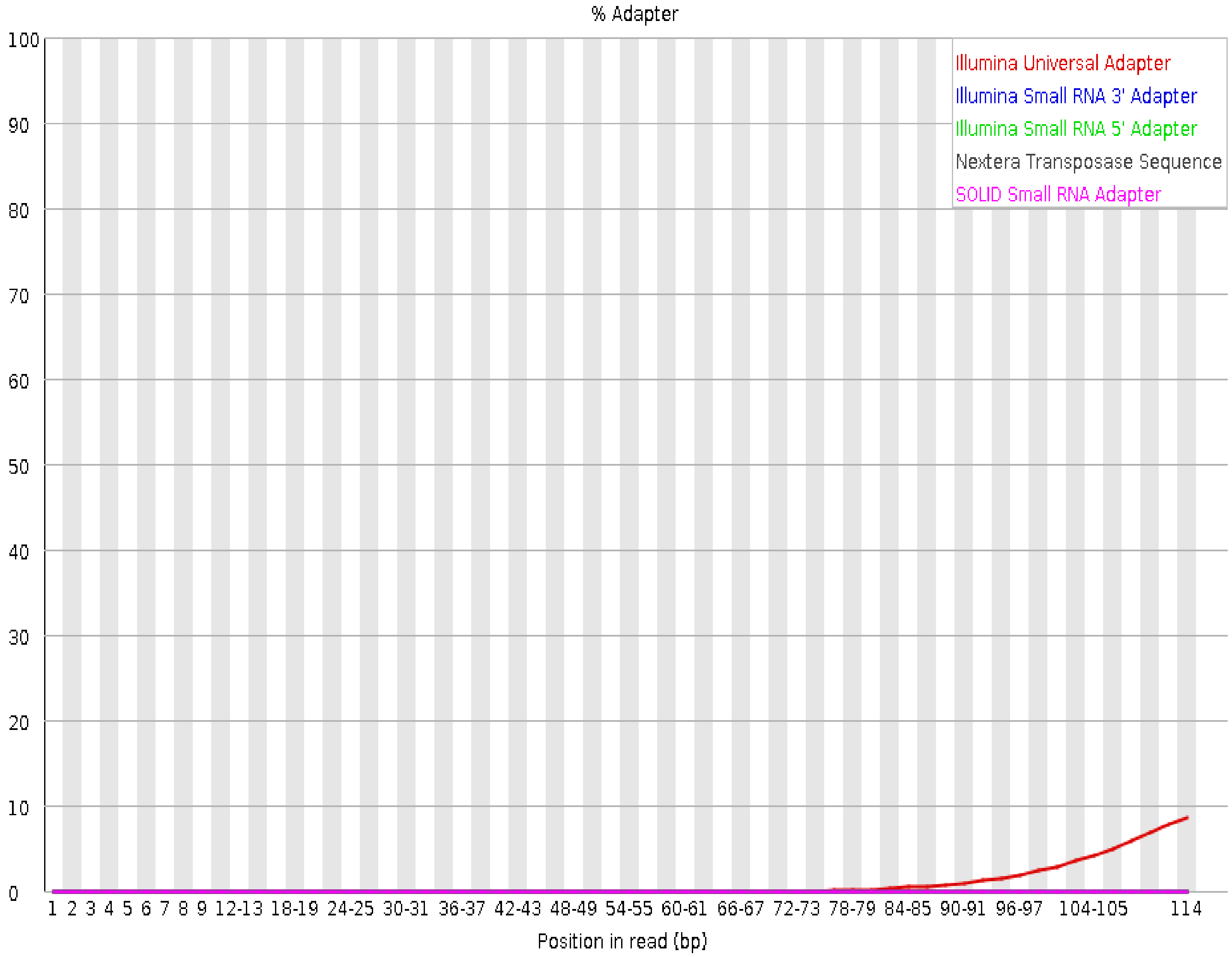

Produced by FastQC (version 0.11.8)

FastQC Report

Wed 9 Oct 2019
Eulamprus.Male.Brain_R1.fastq.gz

Summary

- Basic Statistics
- Per base sequence quality
- Per tile sequence quality
- Per sequence quality scores
- Per base sequence content
- Per sequence GC content
- Per base N content
- Sequence Length Distribution
- Sequence Duplication Levels
- Overrepresented sequences
- Adapter Content

Basic Statistics

Measure	Value
Filename	Eulamprus.Male.Brain_R1.fastq.gz
File type	Conventional base calls
Encoding	Sanger / Illumina 1.9
Total Sequences	45518999
Sequences flagged as poor quality	0
Sequence length	125
%GC	46

Per base sequence quality

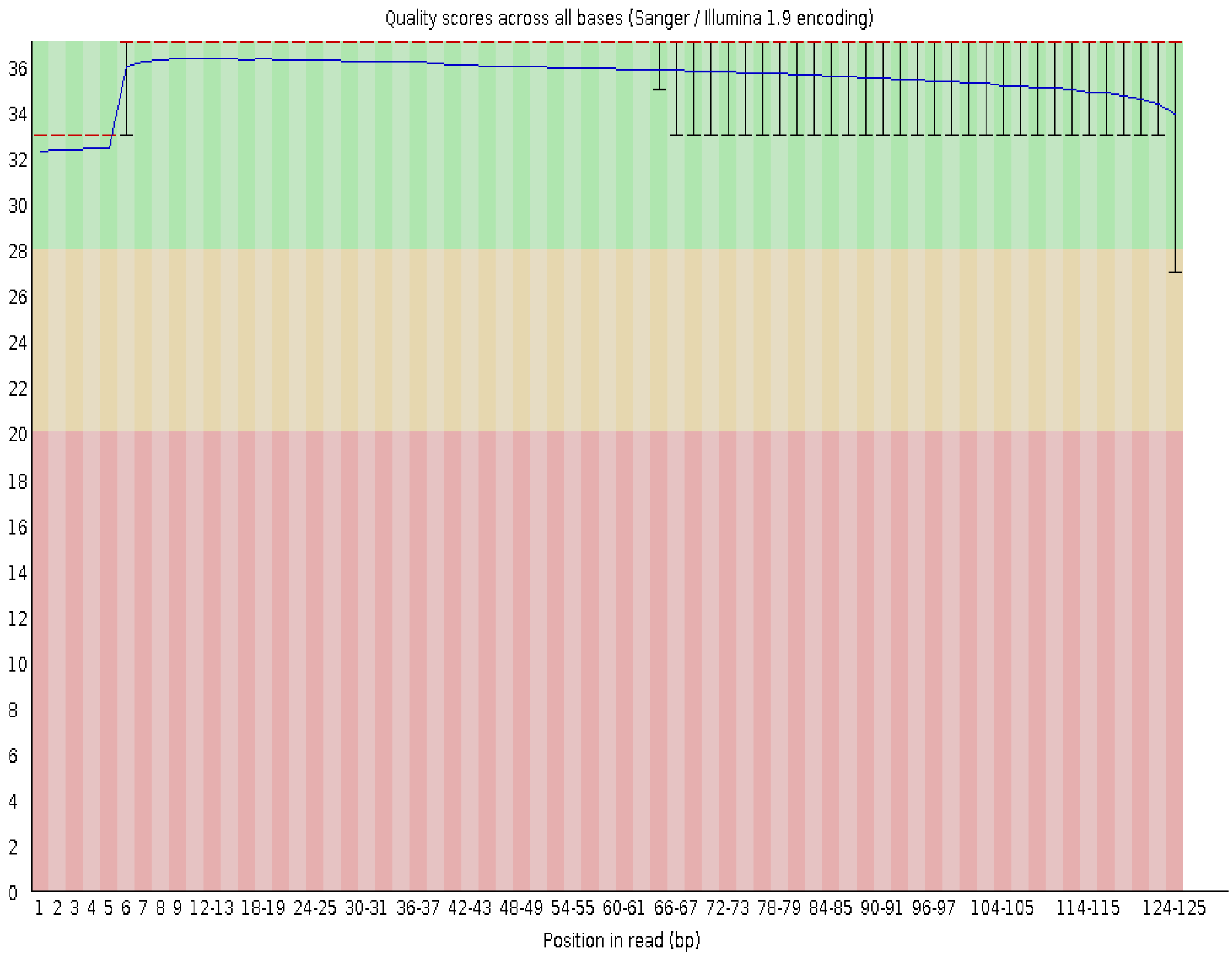

Per tile sequence quality

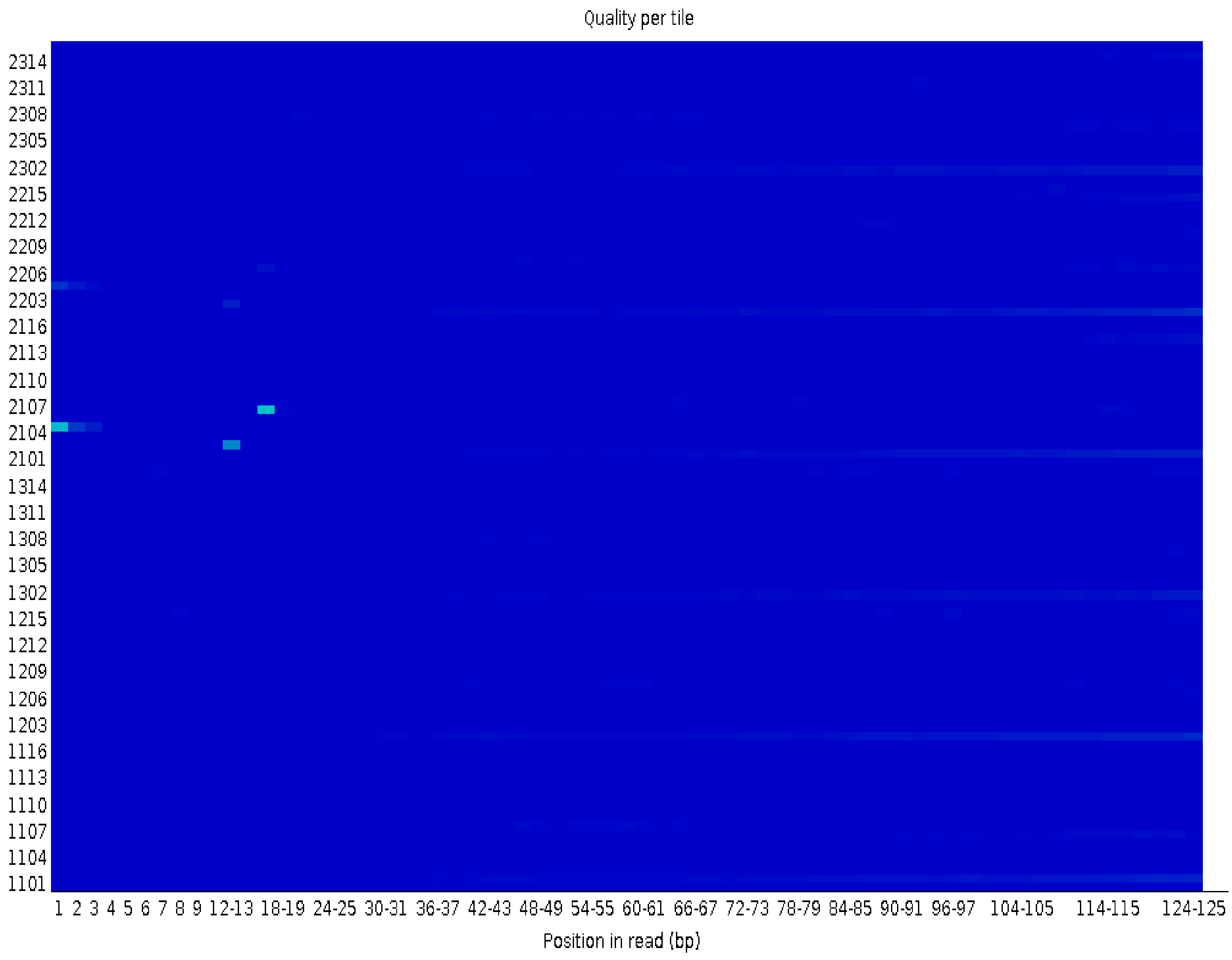

Per sequence quality scores

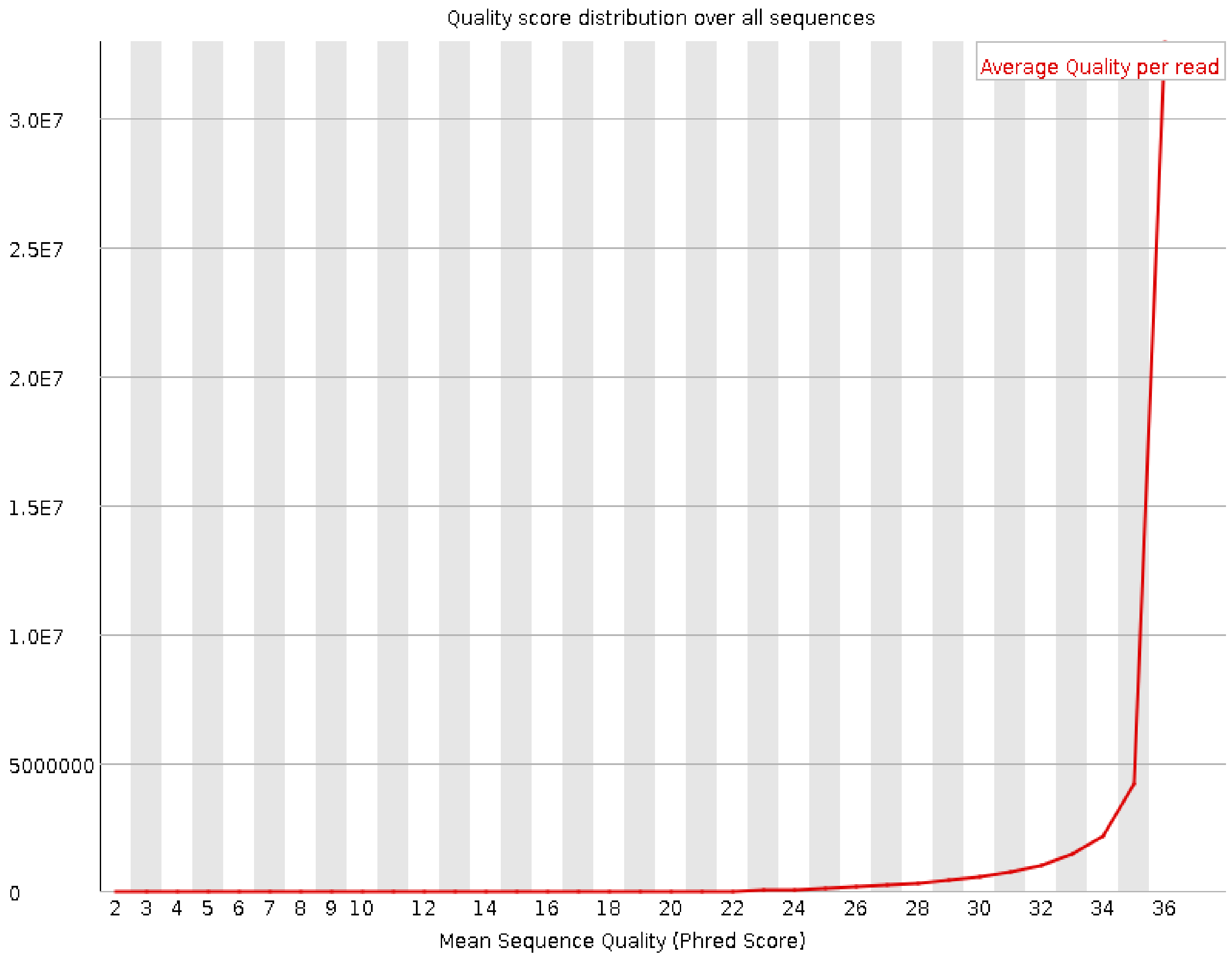

Per base sequence content

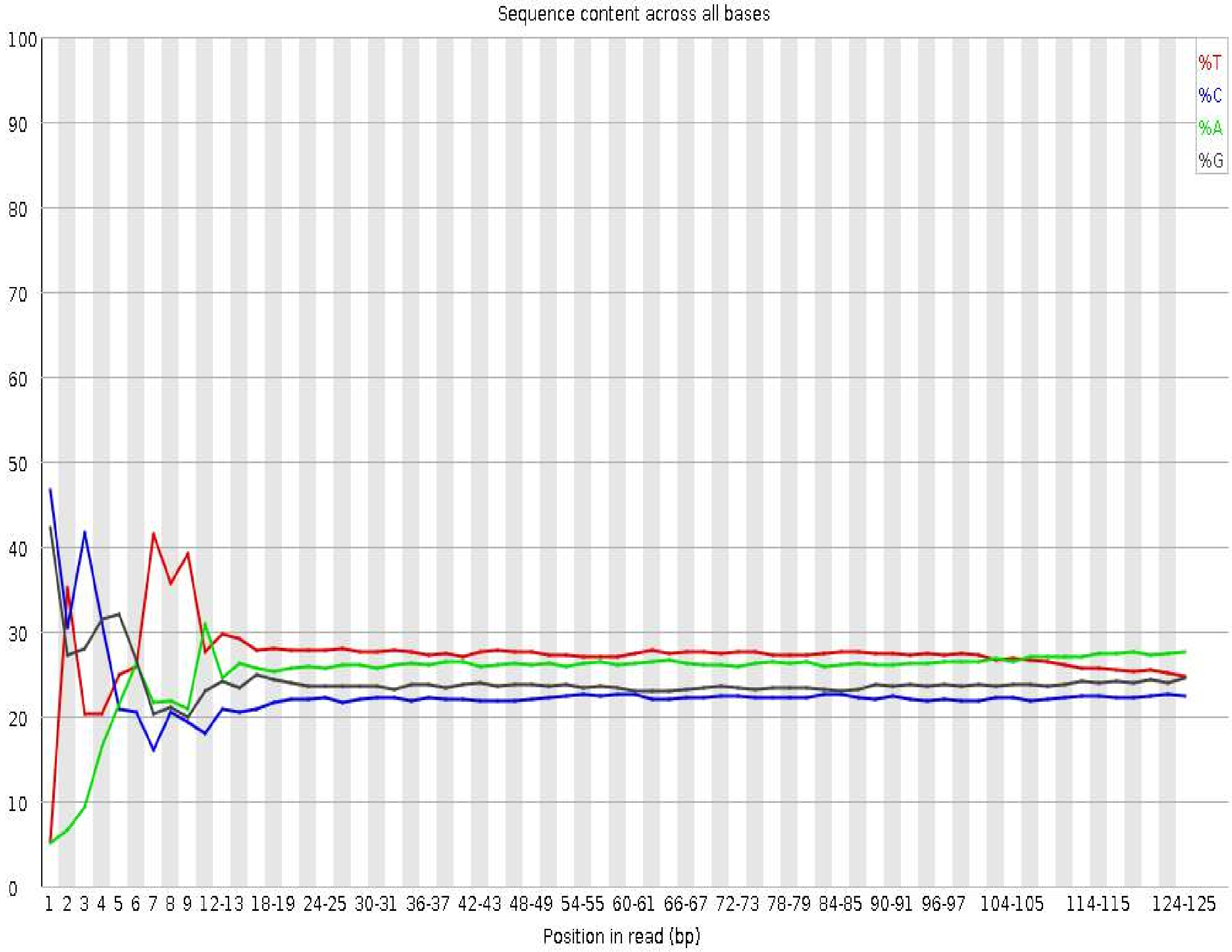

Per sequence GC content

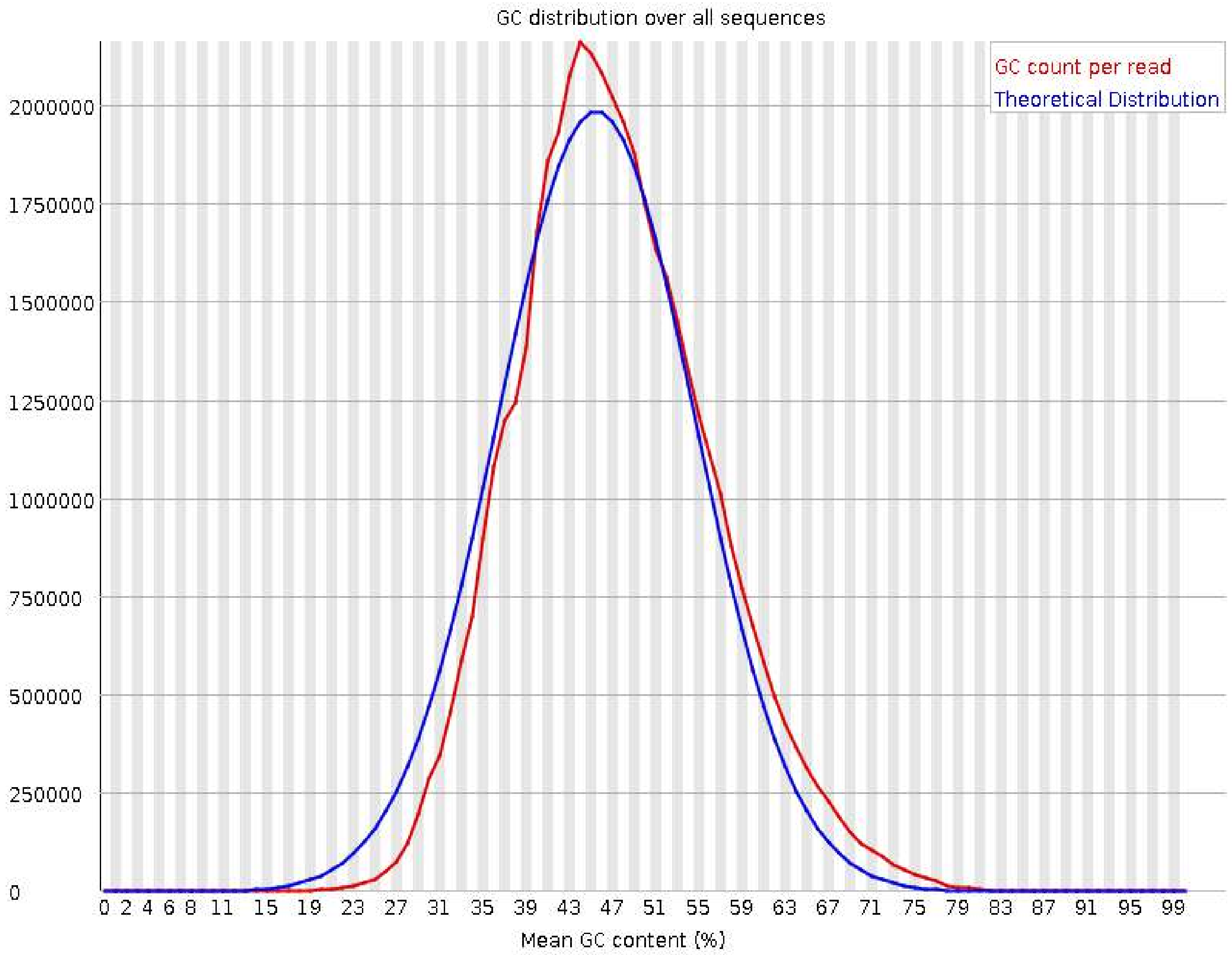

Per base N content

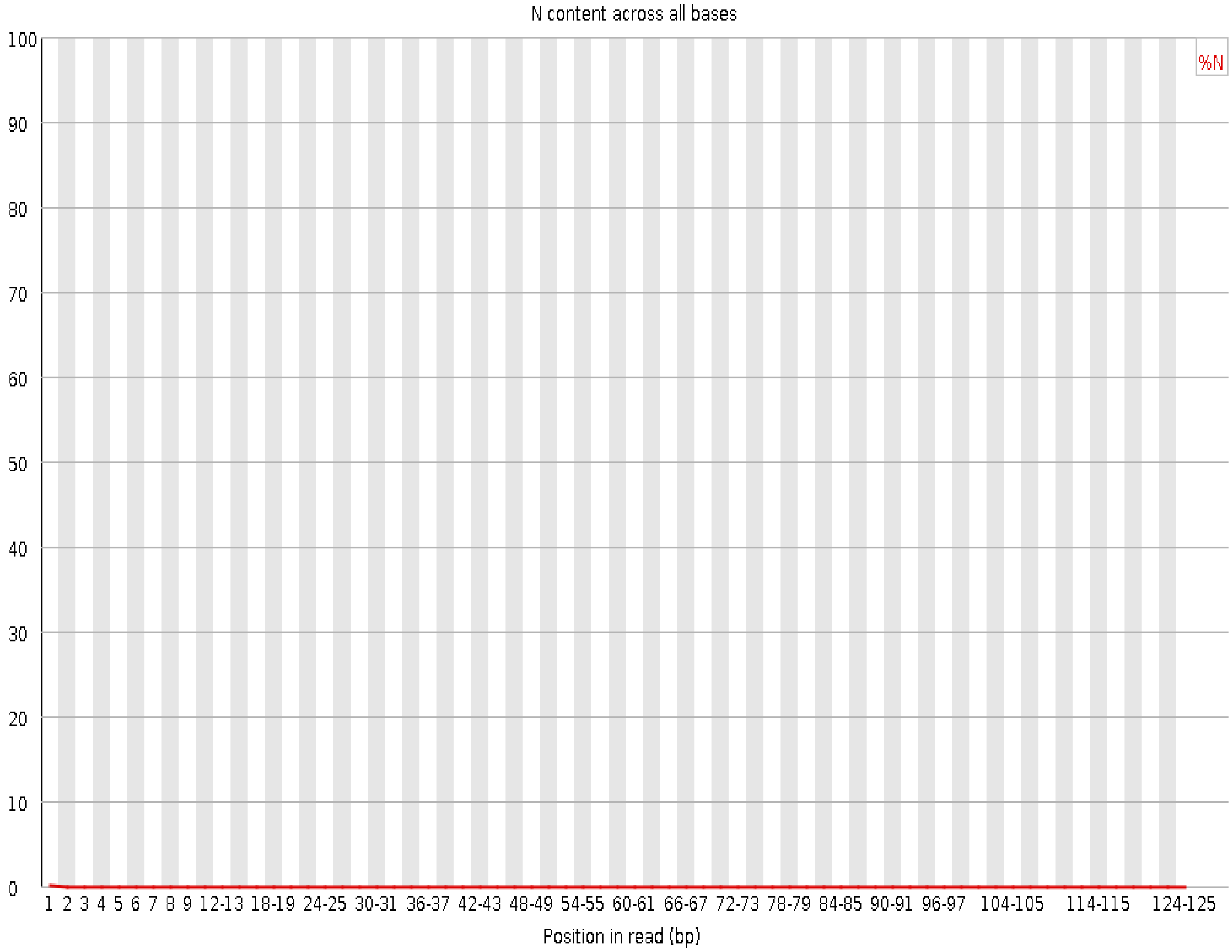

Sequence Length Distribution

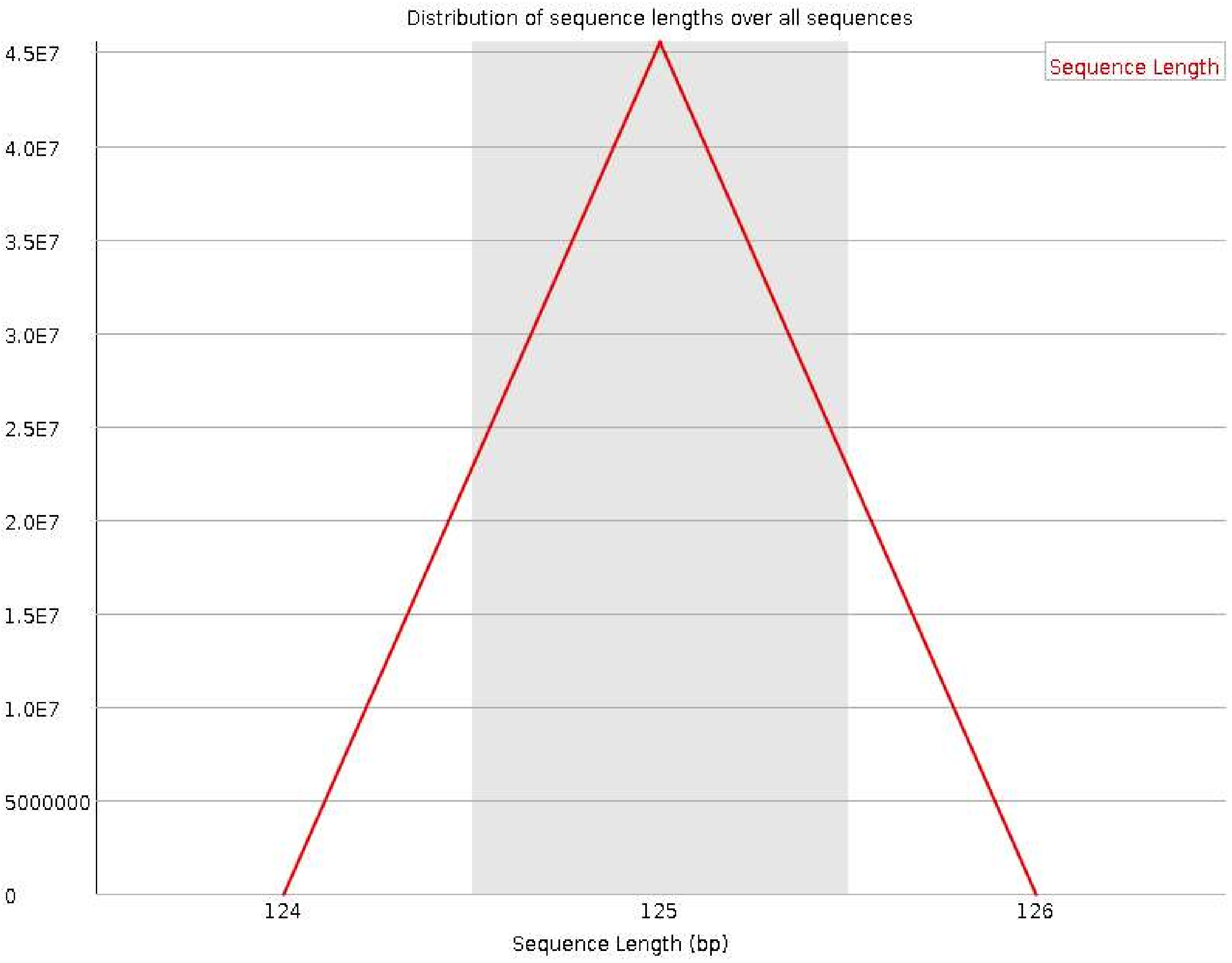

Sequence Duplication Levels

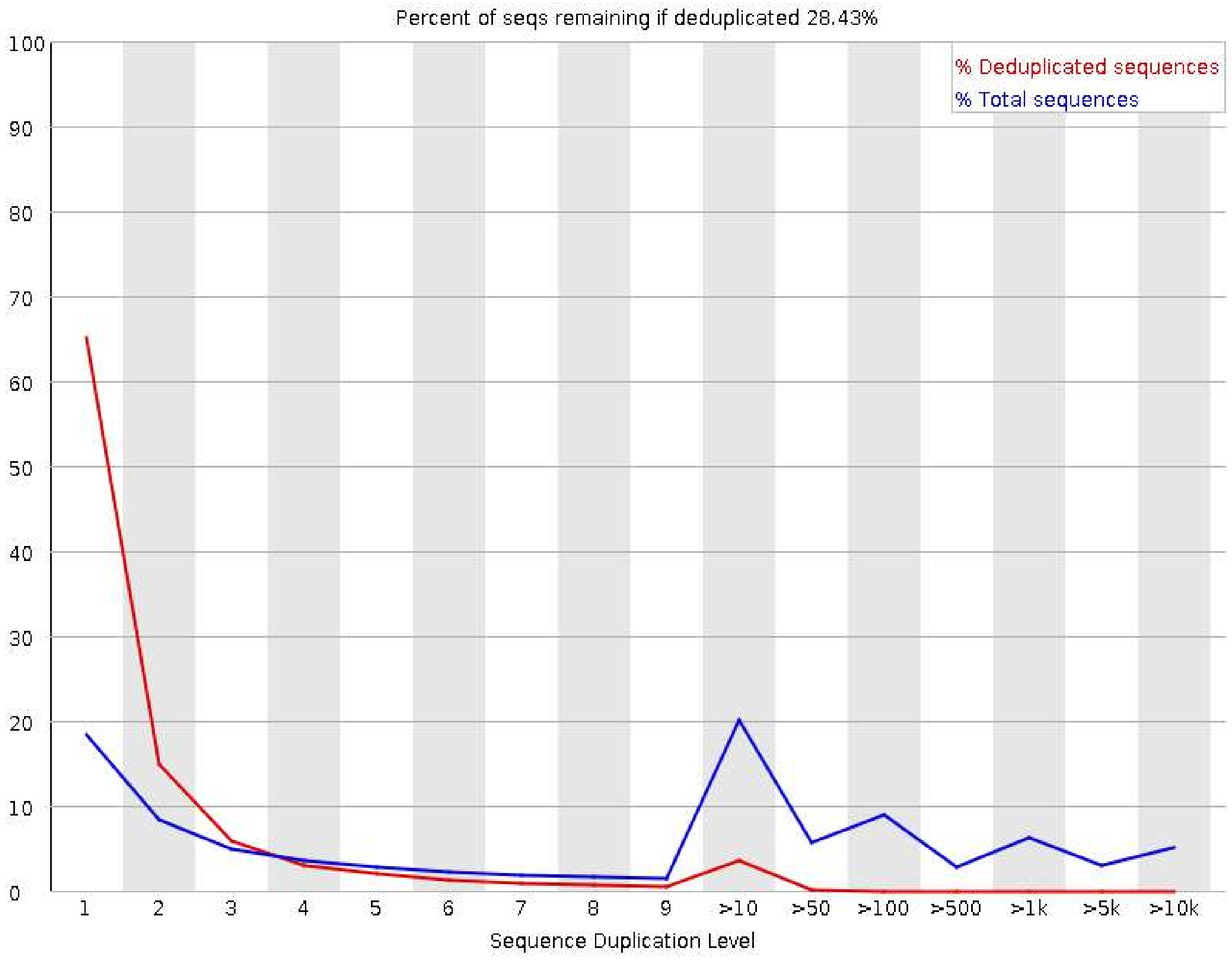

Overrepresented sequences

Sequence	Count	Percentage	Possible Source
GTCGGTTGTAAAGTGGTATAAGACTTGTCGTAATAGGCAAATAATTAAGA	85860	0.18862453455973405	No Hit
GTCGTCTTTGTCGTGGCCGGGACTAGGTTATCTAAGTAAGTATCGTTCGG	61466	0.13503372514848141	No Hit
GTAAGGATTTAAGAAAGGTTCTTATGAGGATGGTAAGGTAGGGTAGGGTA	56483	0.12408664786323619	No Hit
TAAGGATTTAAGAAAGGTTCTTATGAGGATGGTAAGGTAGGGTAGGGTAA	46472	0.1020936334737941	No Hit

Adapter Content

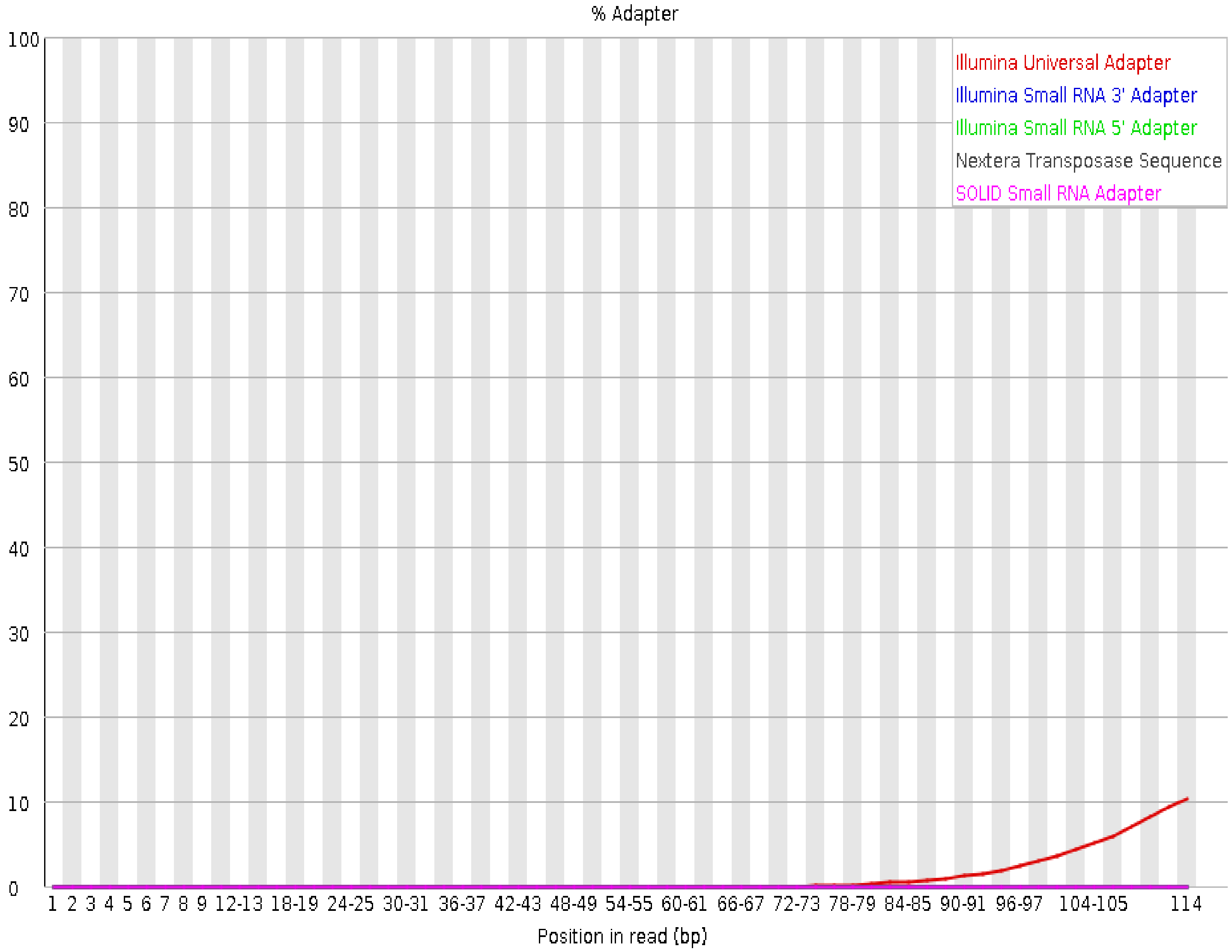

Produced by FastQC (version 0.11.8)

FastQC Report

Wed 9 Oct 2019
Eulamprus.Male.Brain_R2.fastq.gz

Summary

- Basic Statistics
- Per base sequence quality
- Per tile sequence quality
- Per sequence quality scores
- Per base sequence content
- Per sequence GC content
- Per base N content
- Sequence Length Distribution
- Sequence Duplication Levels
- Overrepresented sequences
- Adapter Content

Basic Statistics

Measure	Value
Filename	Eulamprus.Male.Brain_R2.fastq.gz
File type	Conventional base calls
Encoding	Sanger / Illumina 1.9
Total Sequences	45518999
Sequences flagged as poor quality	0
Sequence length	125
%GC	46

Per base sequence quality

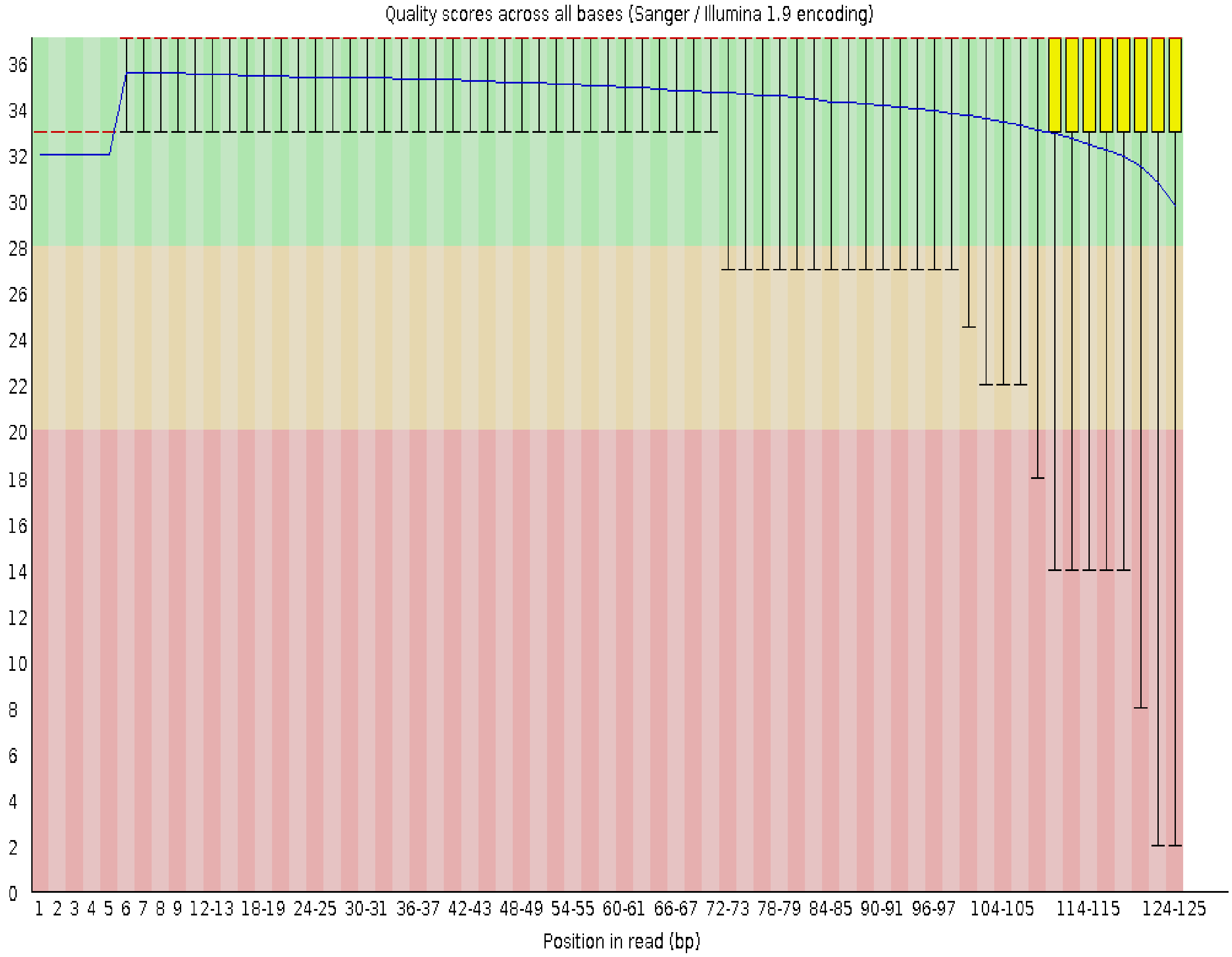

Per tile sequence quality

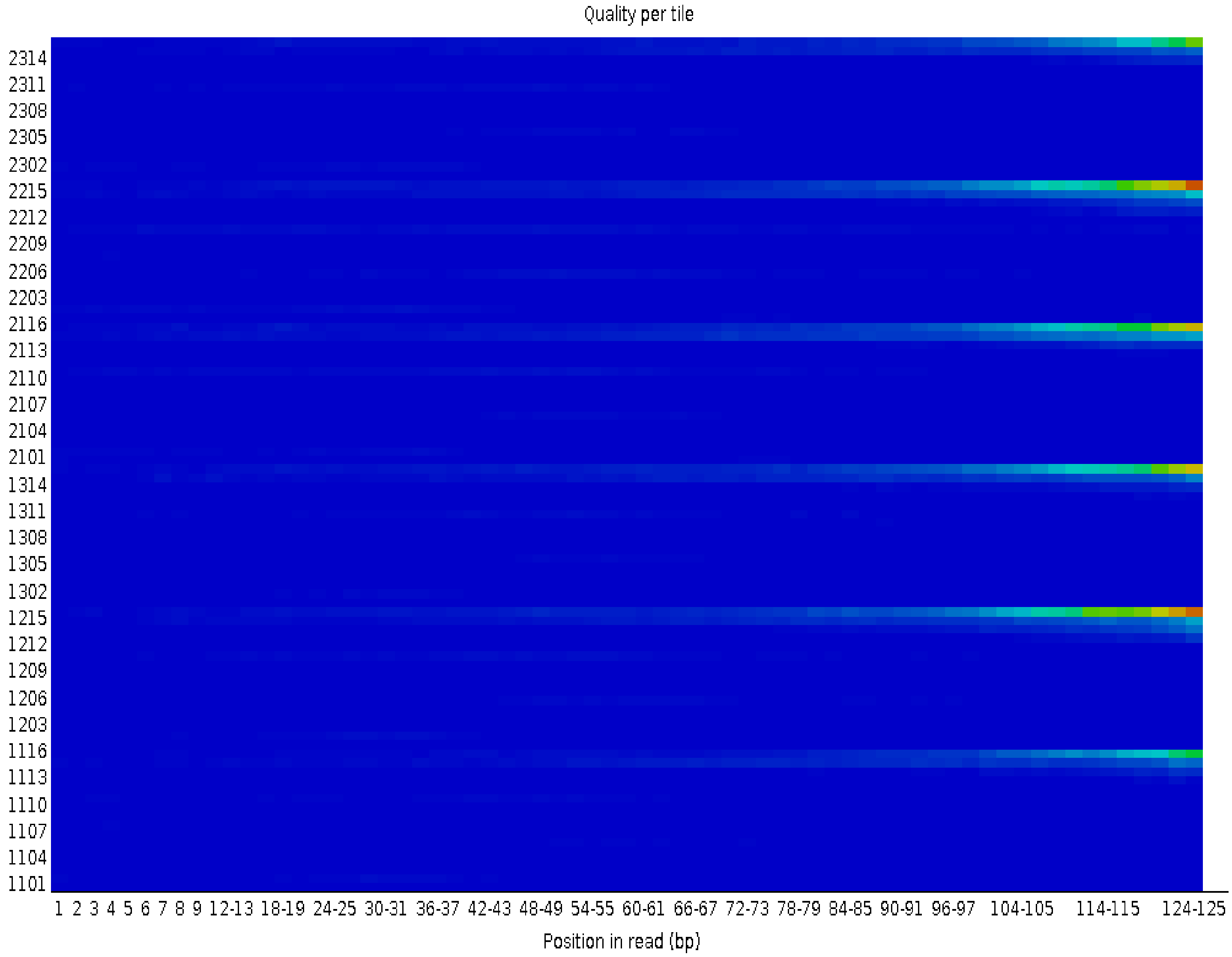

Per sequence quality scores

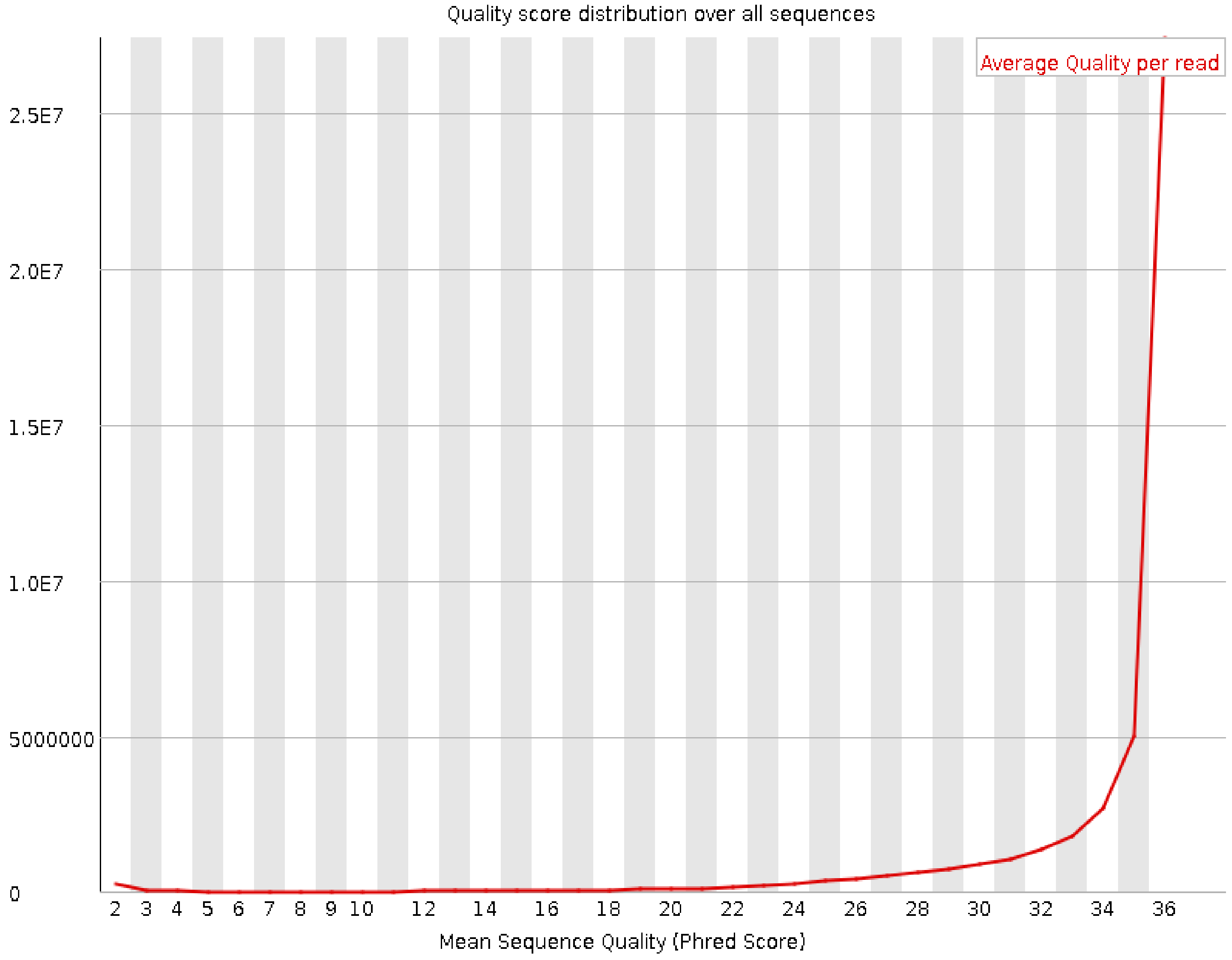

Per base sequence content

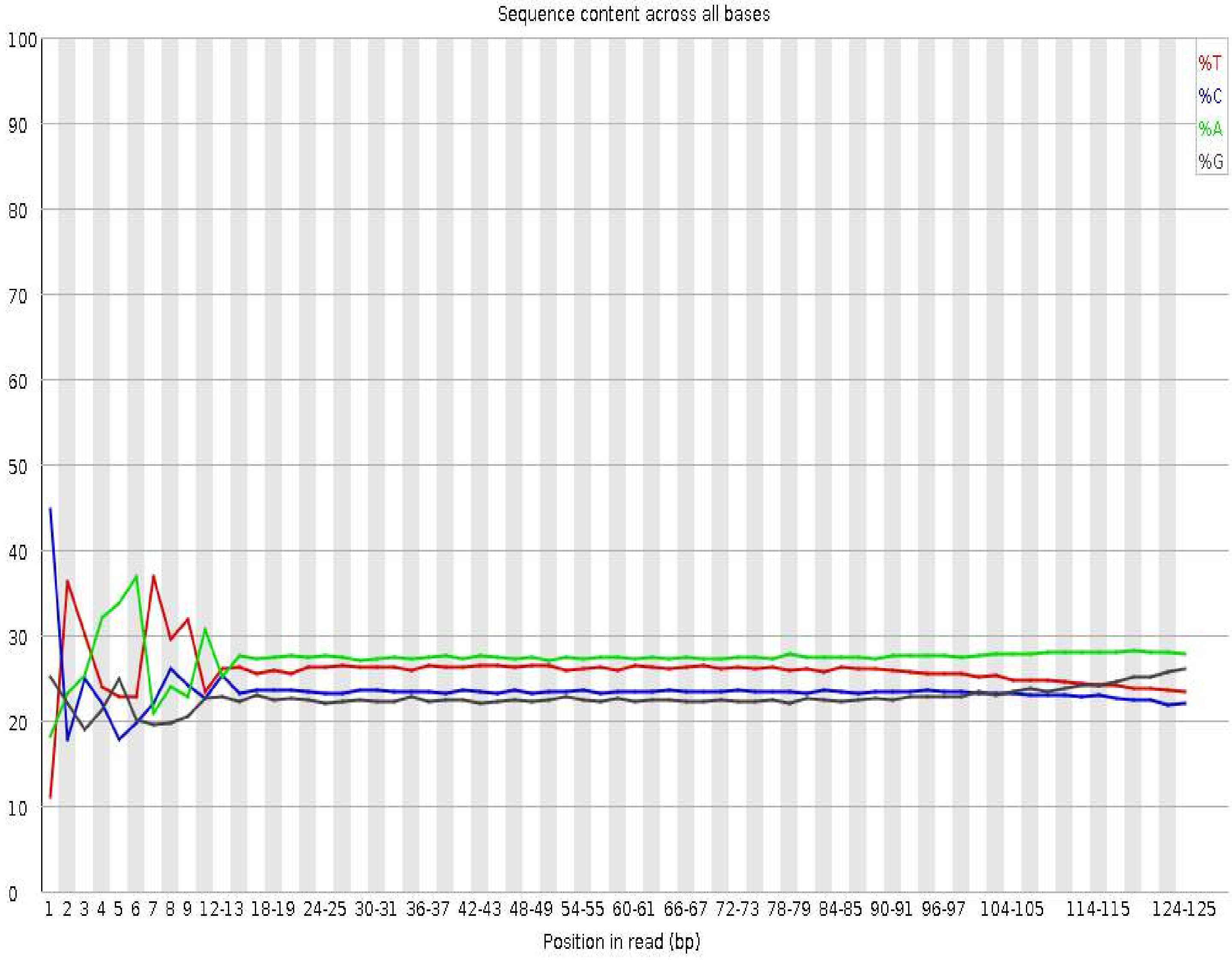

Per sequence GC content

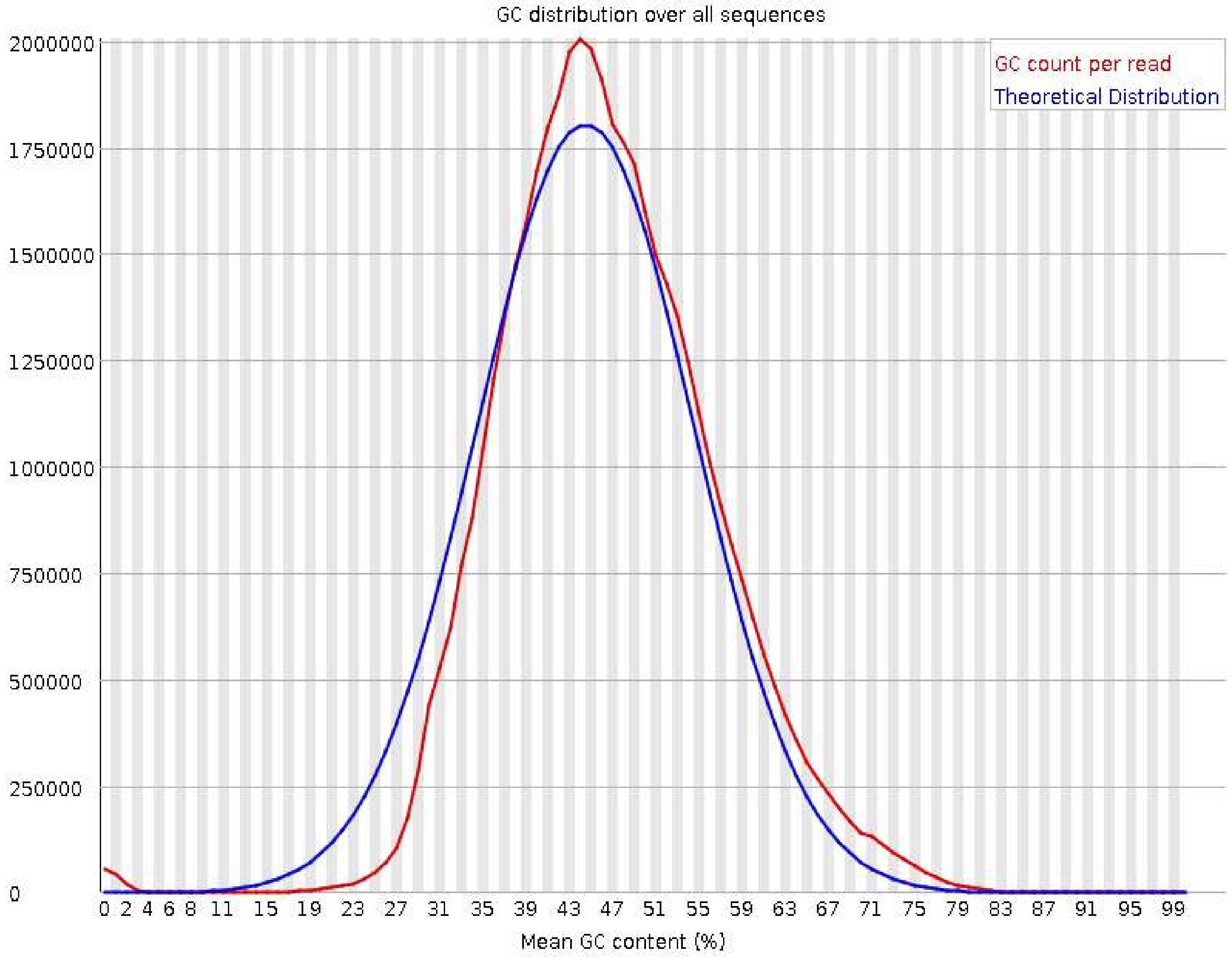

Per base N content

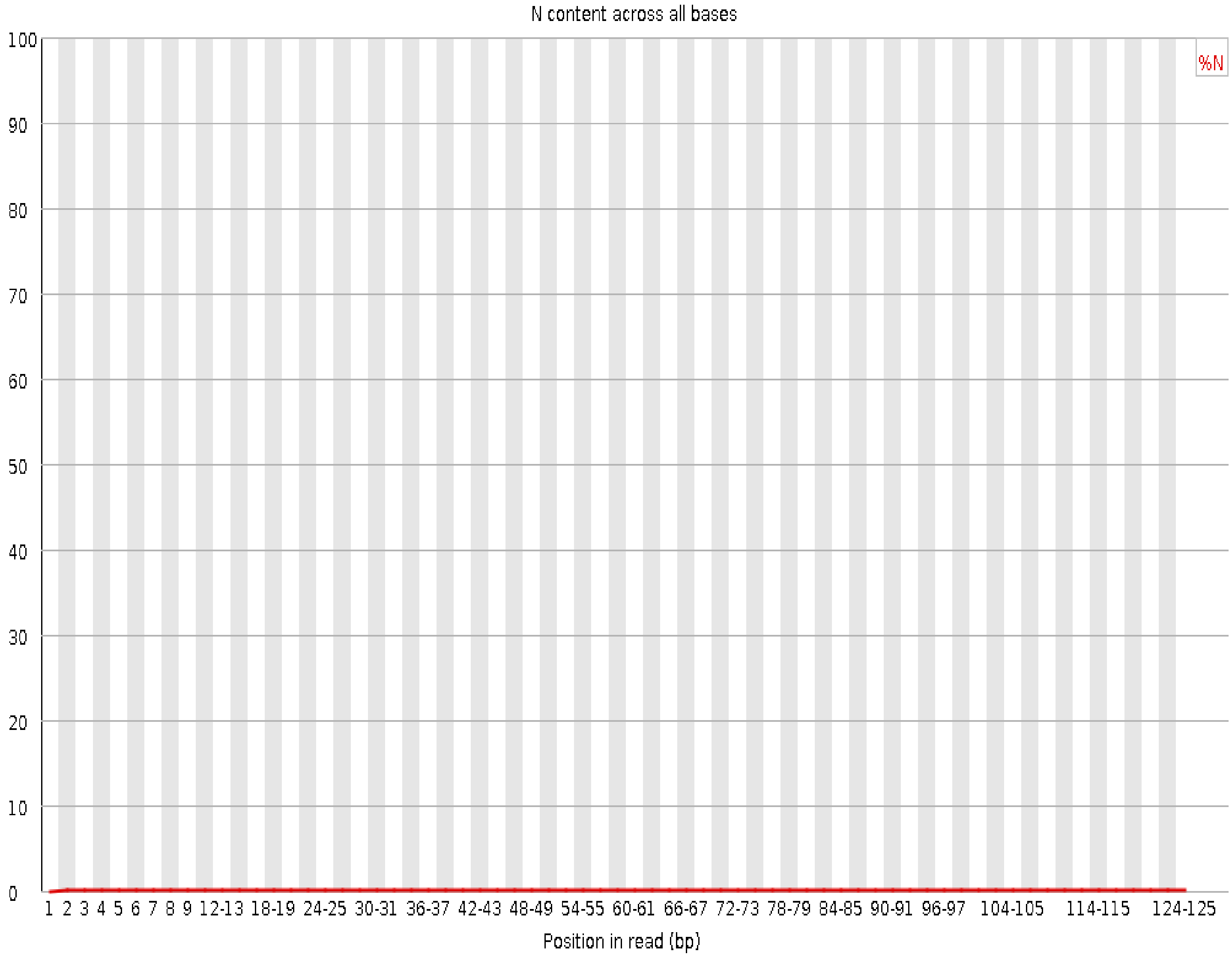

Sequence Length Distribution

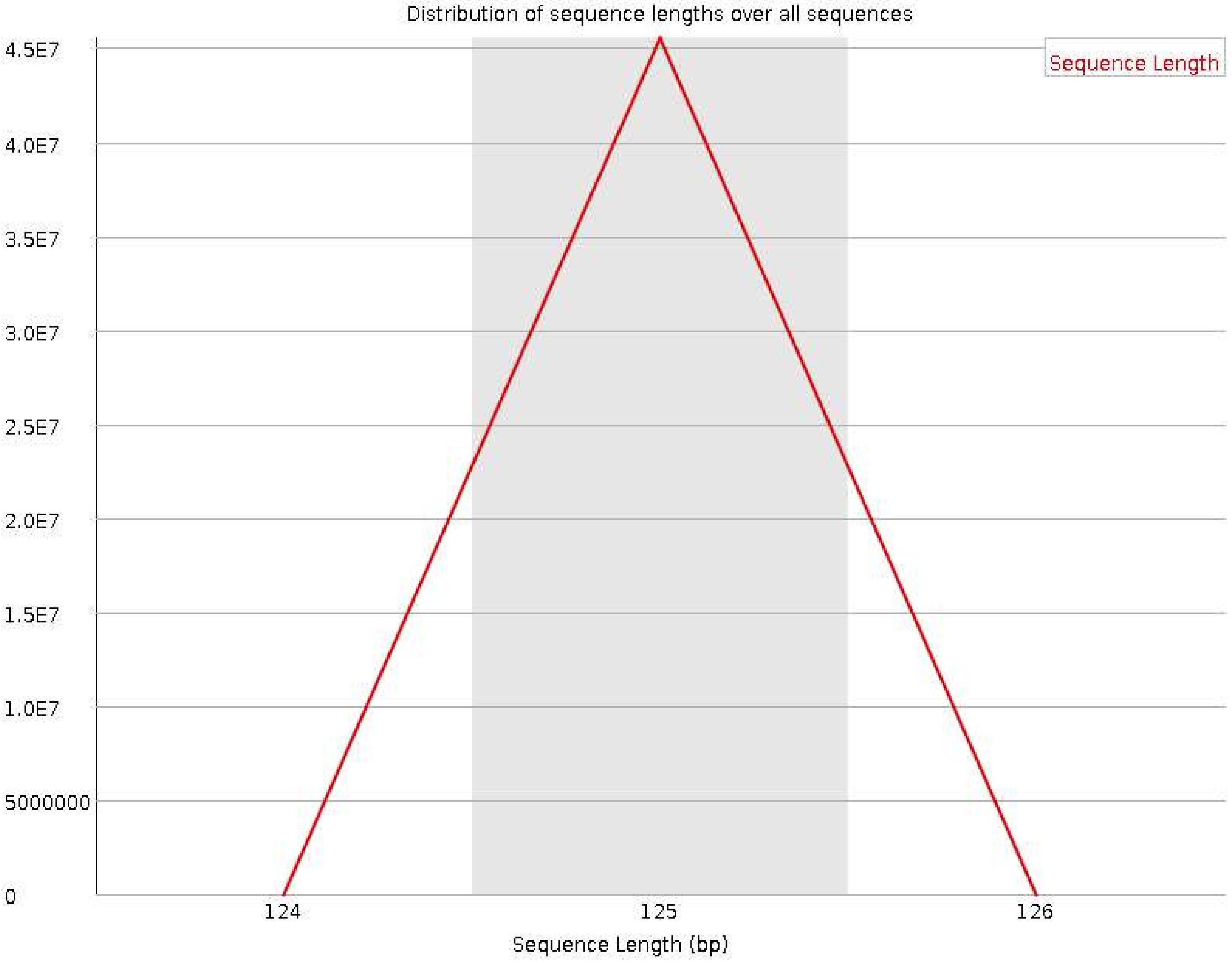

Sequence Duplication Levels

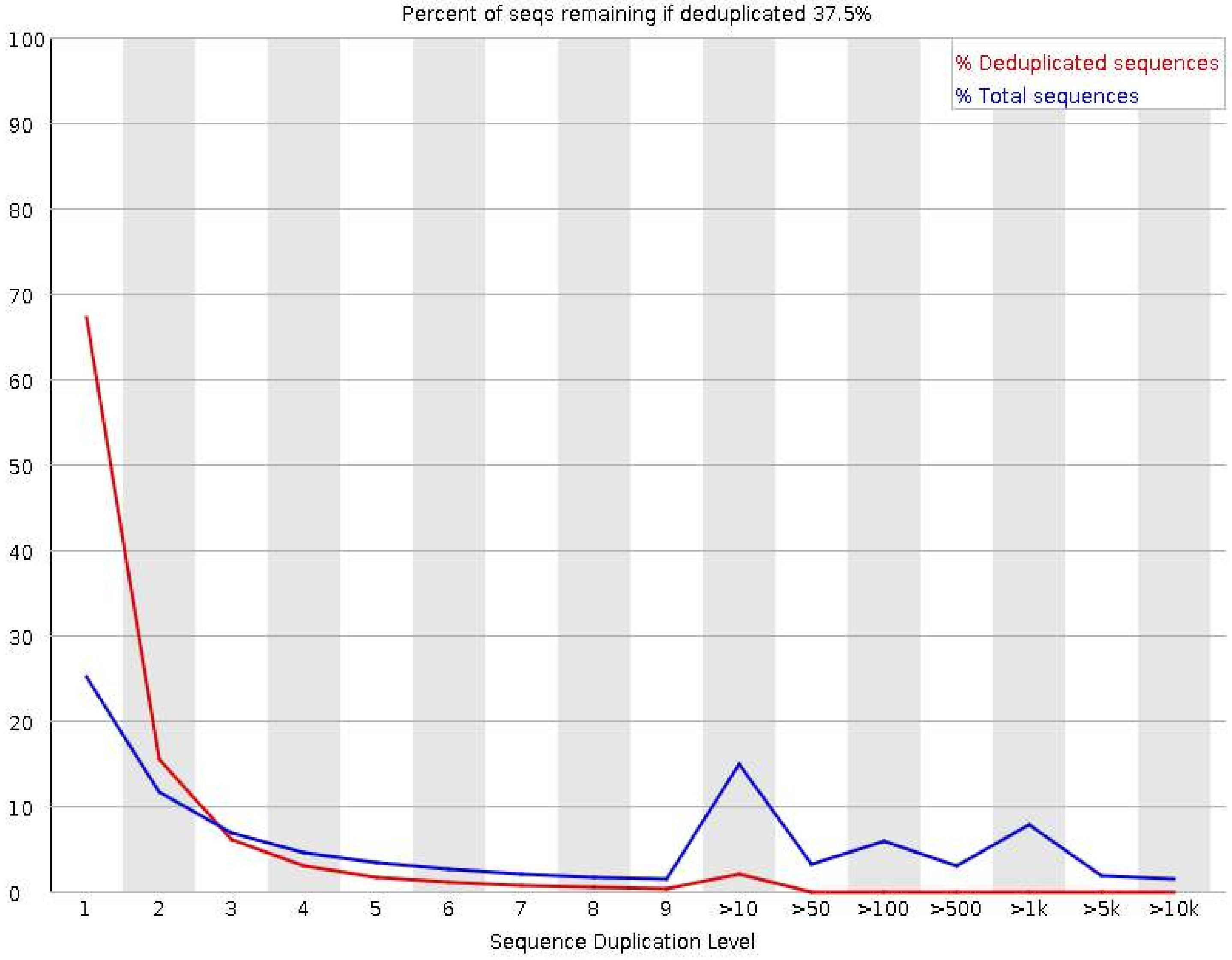

Overrepresented sequences

Sequence	Count	Percentage	Possible Source
CTCAGTTCTCTTGAAACCTACATAAATTCGCAATTATGACAATATATTAC	128192	0.281623064689977	No Hit

Adapter Content

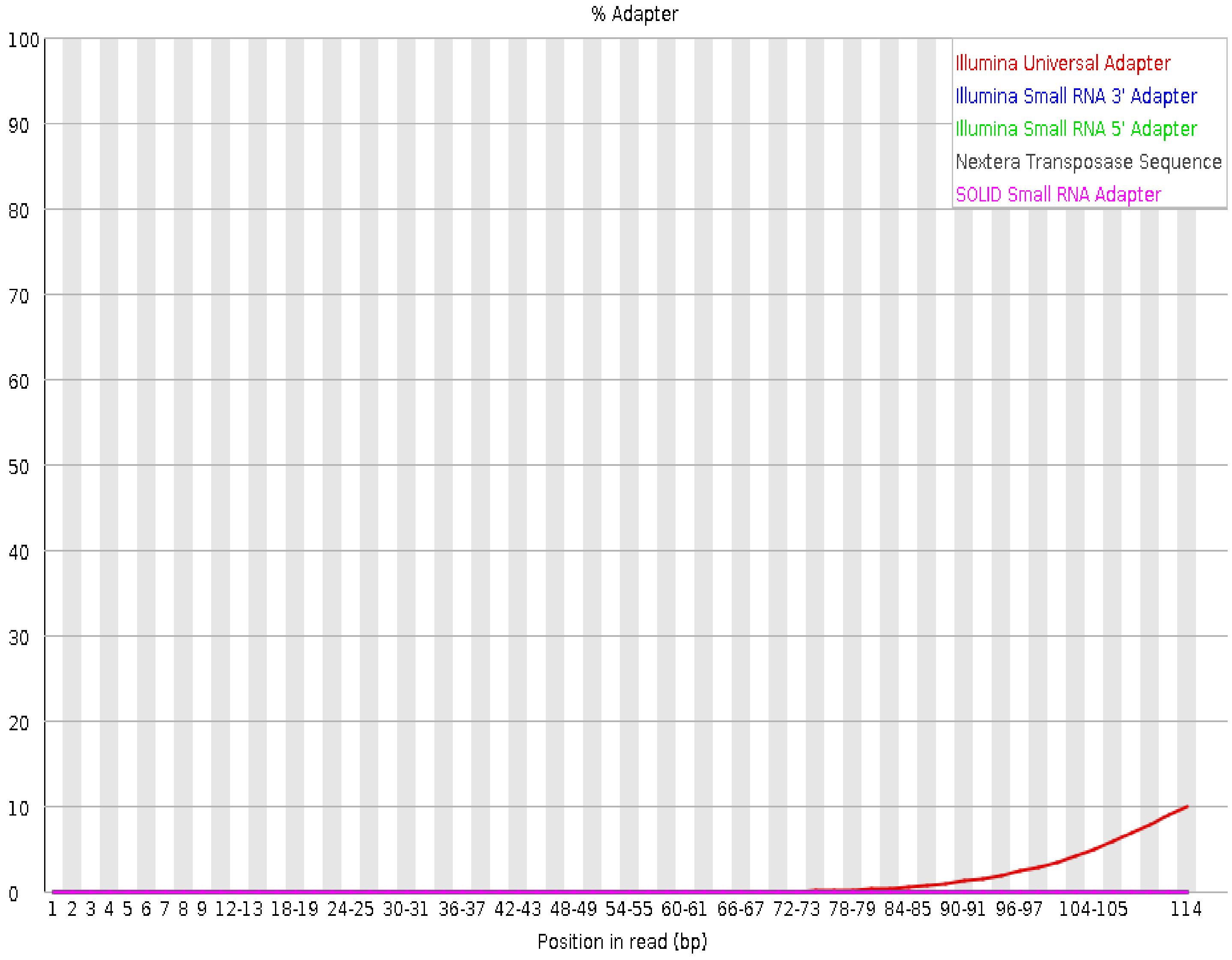

Produced by FastQC (version 0.11.8)

FastQC Report

Wed 9 Oct 2019
Eulamprus.Male.Liver_R1.fastq.gz

Summary

- Basic Statistics
- Per base sequence quality
- Per tile sequence quality
- Per sequence quality scores
- Per base sequence content
- Per sequence GC content
- Per base N content
- Sequence Length Distribution
- Sequence Duplication Levels
- Overrepresented sequences
- Adapter Content

Basic Statistics

Measure	Value
Filename	Eulamprus.Male.Liver_R1.fastq.gz
File type	Conventional base calls
Encoding	Sanger / Illumina 1.9
Total Sequences	42406849
Sequences flagged as poor quality	0
Sequence length	125
%GC	44

Per base sequence quality

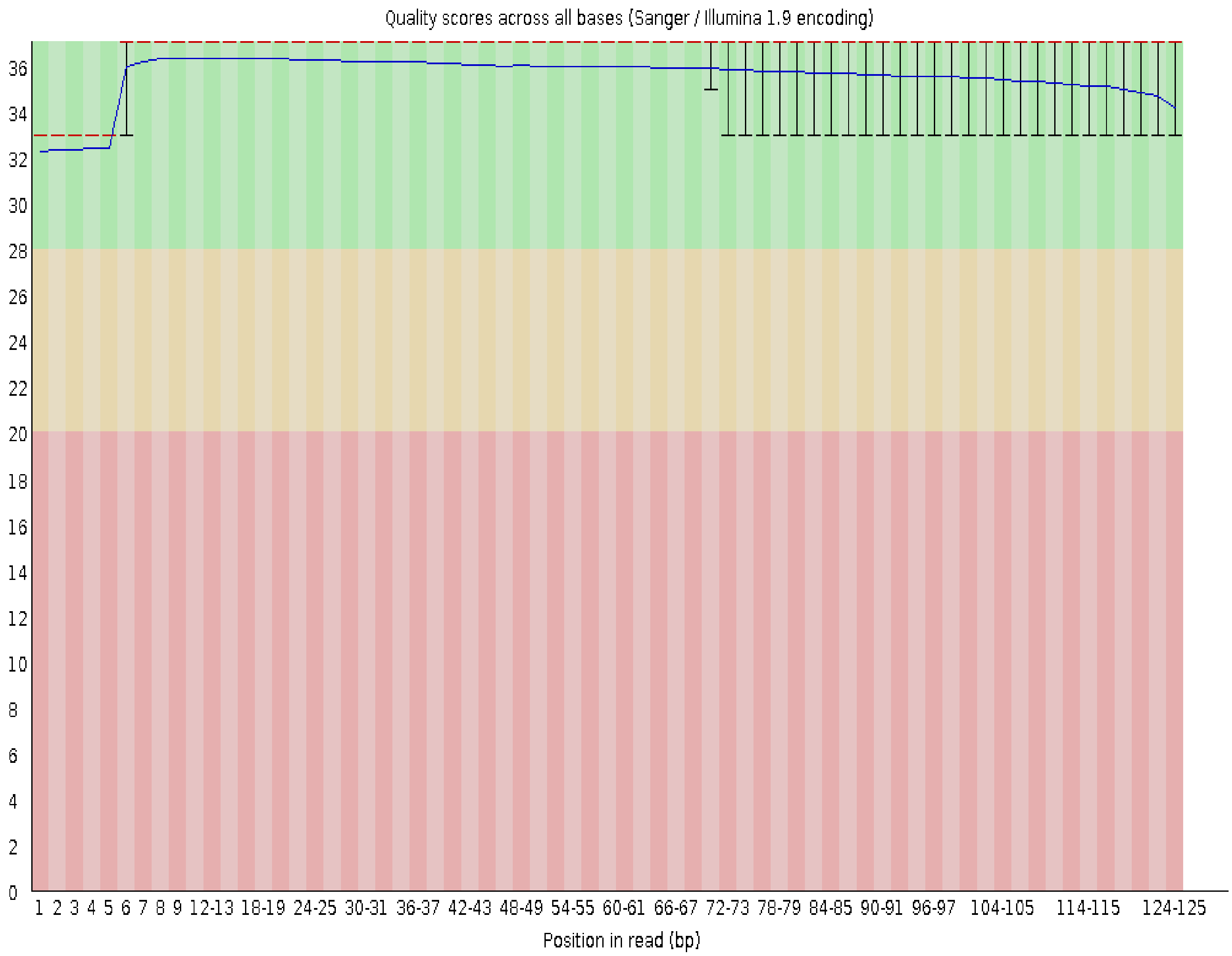

Per tile sequence quality

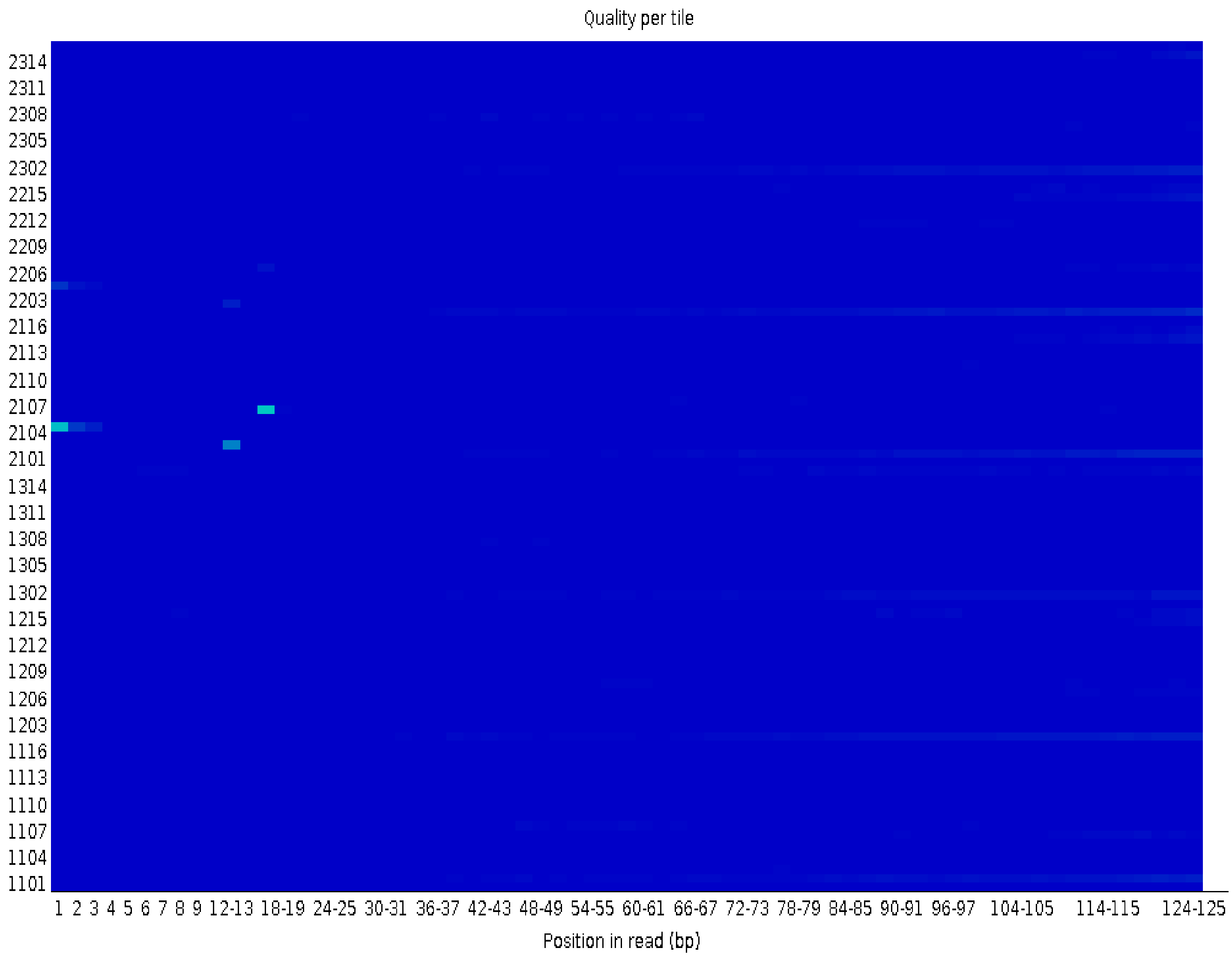

Per sequence quality scores

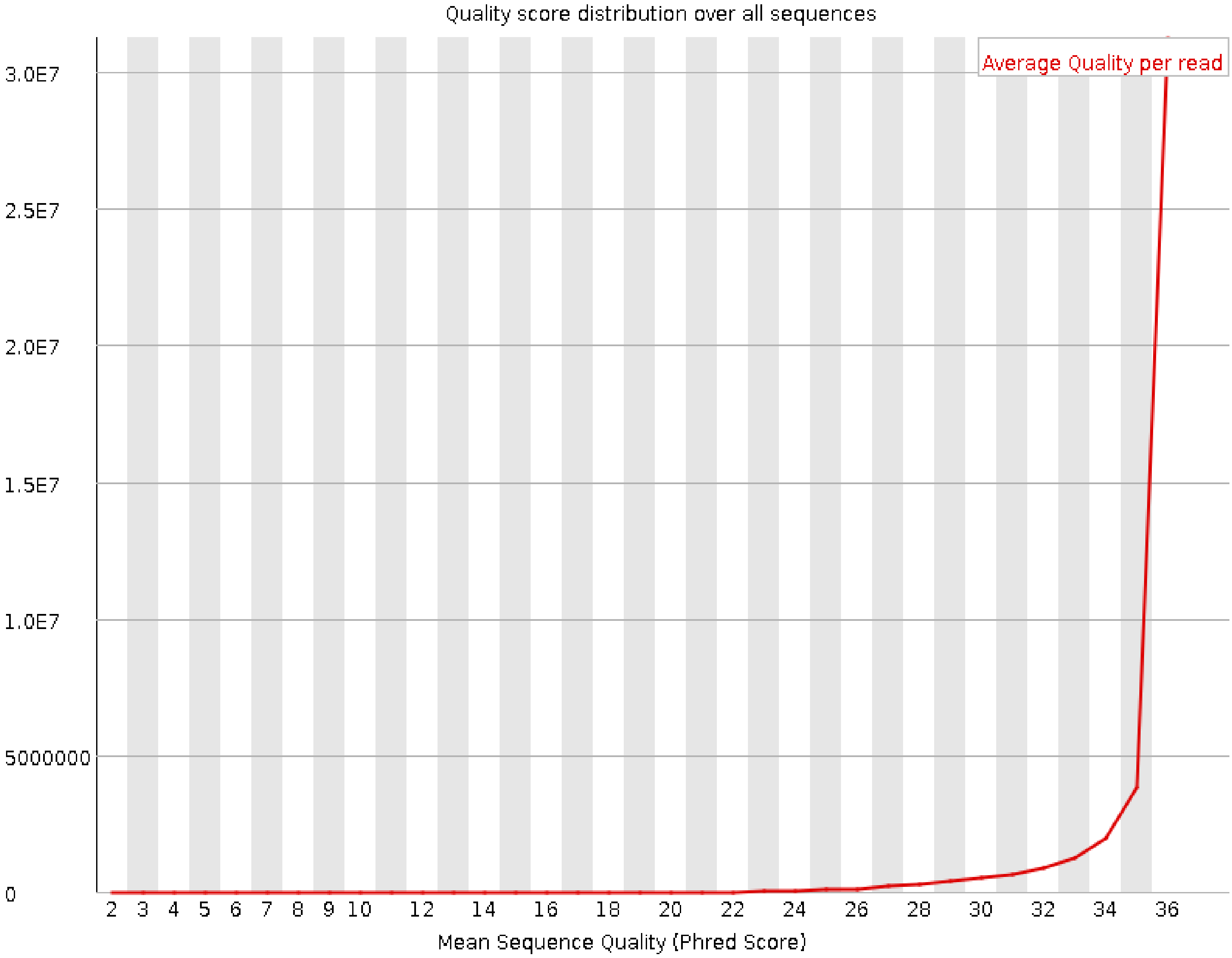

Per base sequence content

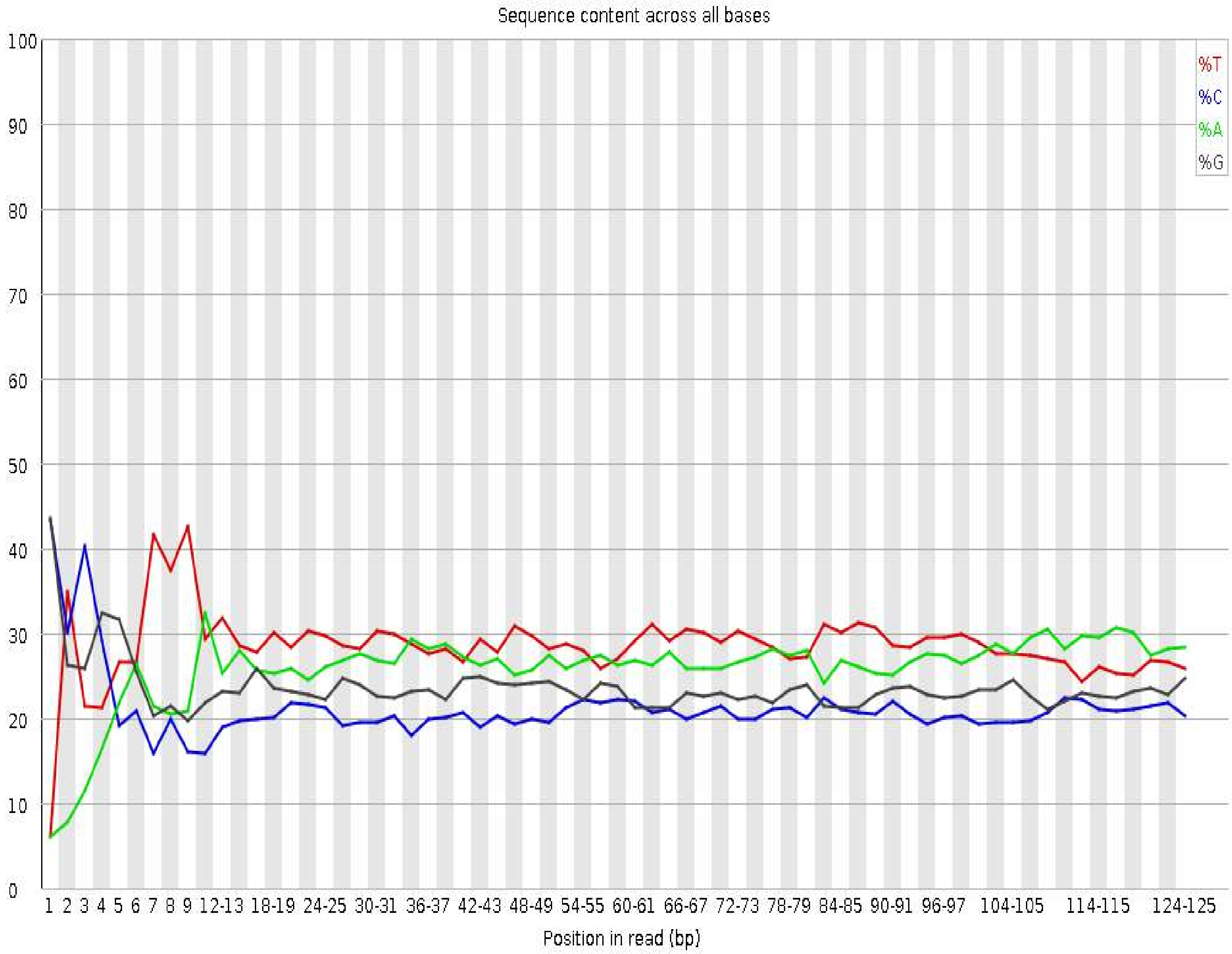

Per sequence GC content

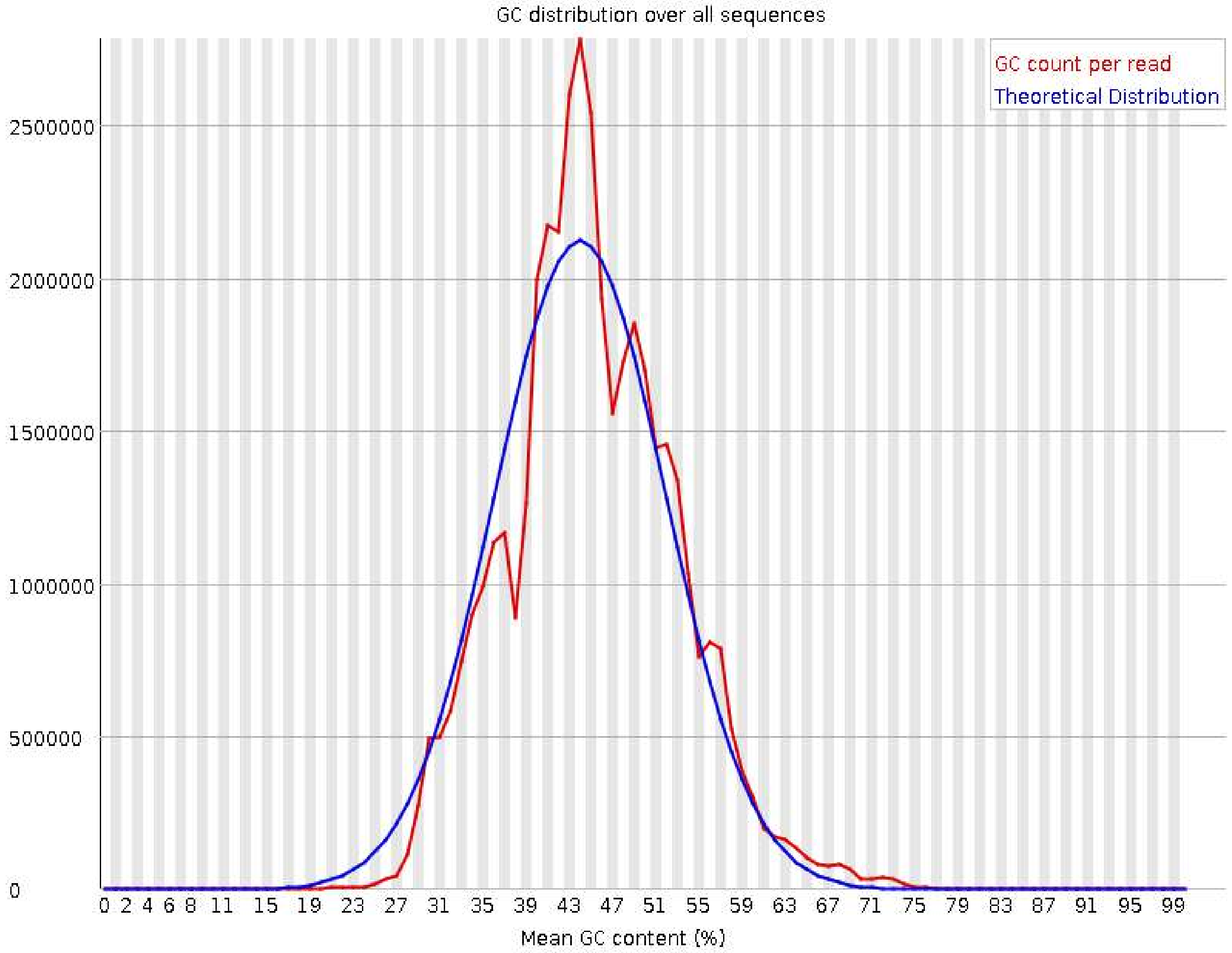

Per base N content

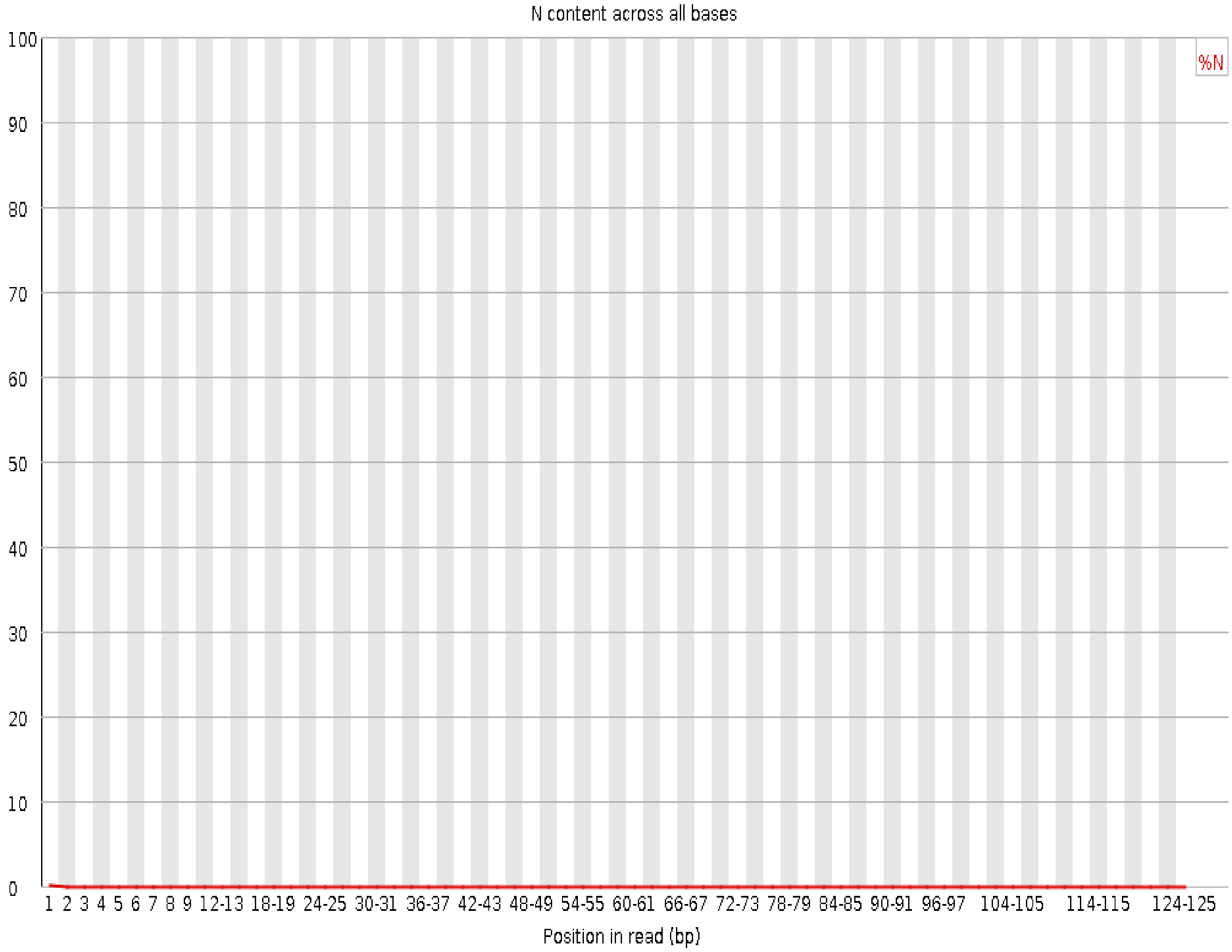

Sequence Length Distribution

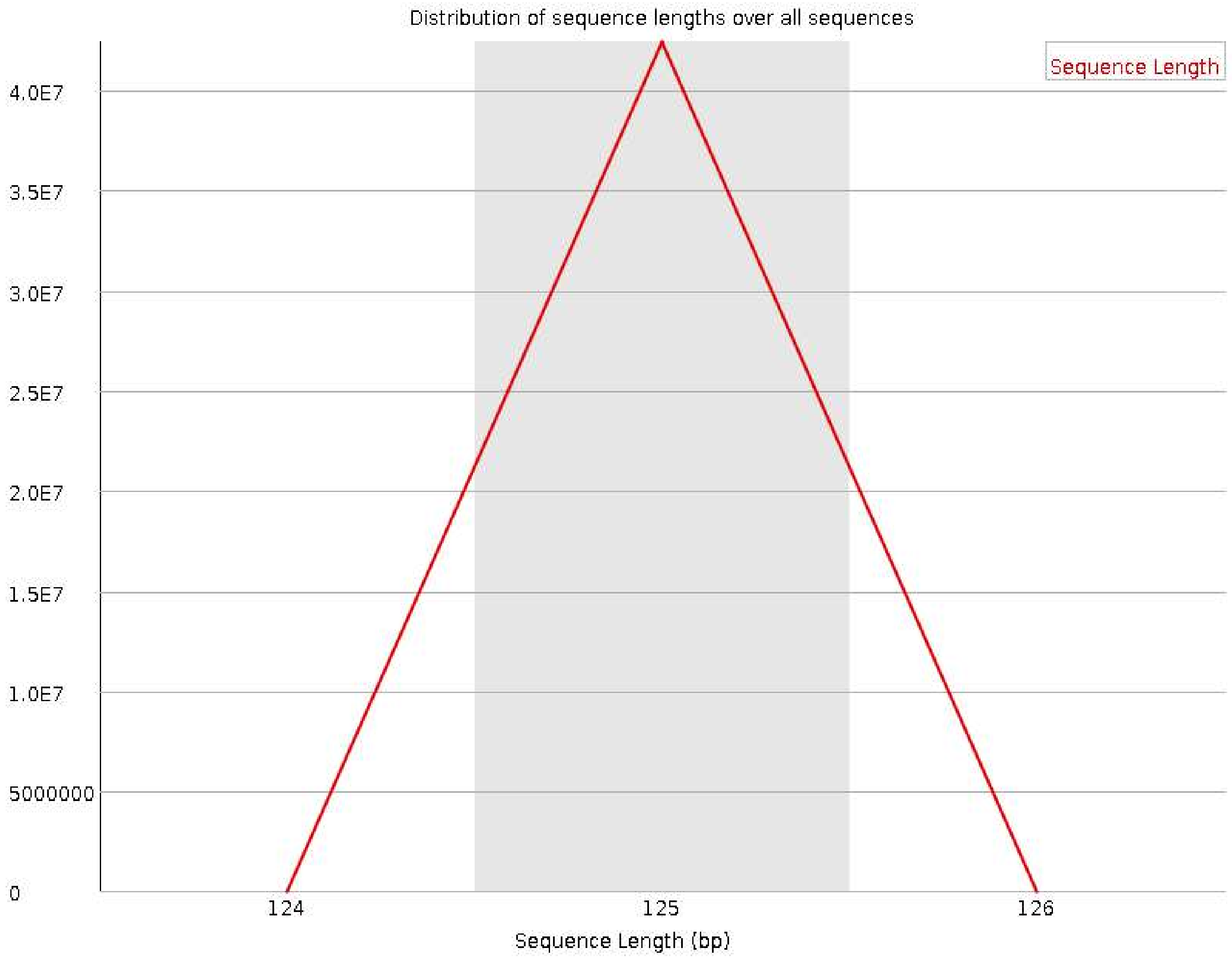

Sequence Duplication Levels

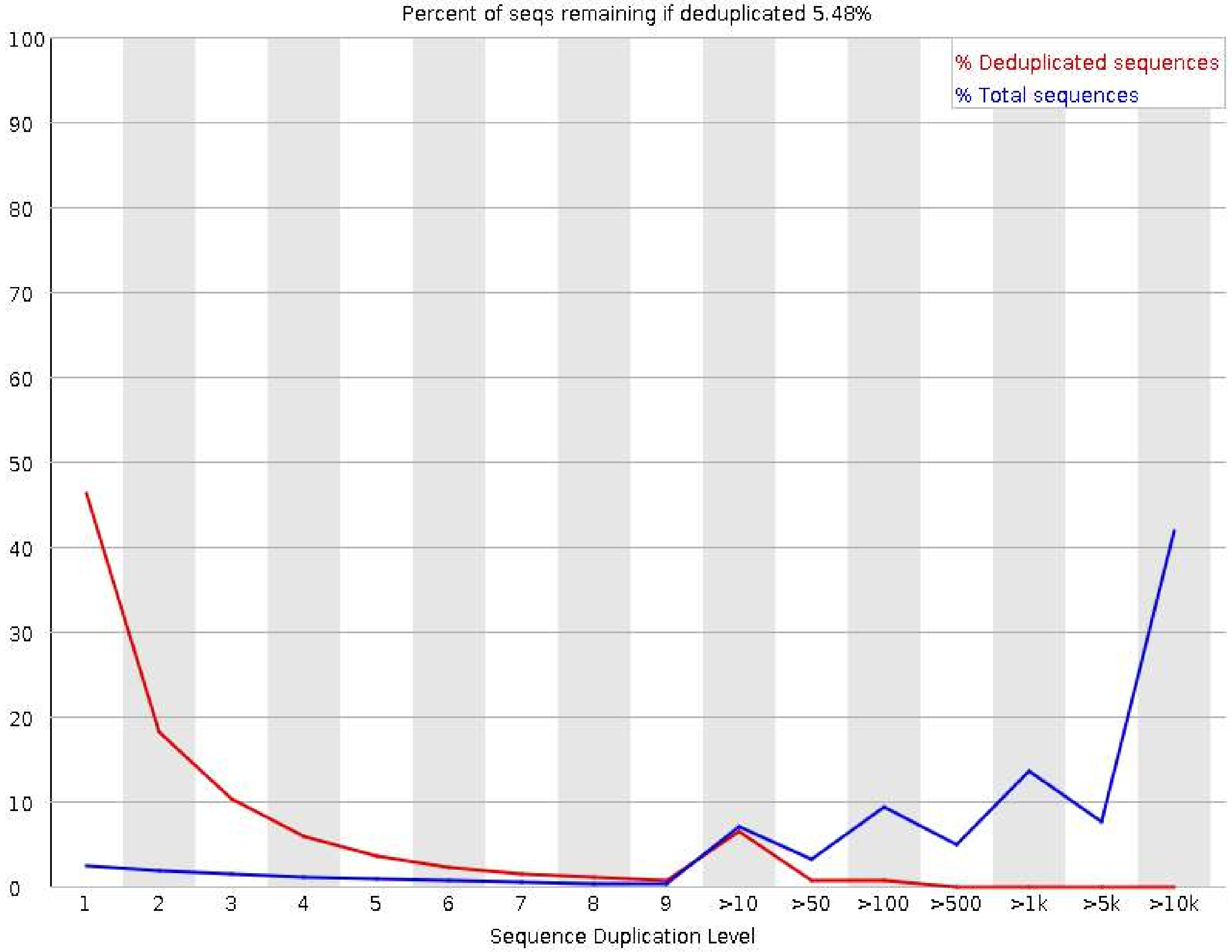

Overrepresented sequences

Sequence	Count	Percentage	Possible Source
GTCGTCTTTGTCGTGGCCGGGACTAGGTTATCTAAGTAAGTATCGTTCGG	477190	1.125266345537722	No Hit
GTAAGGATTTAAGAAAGGTTCTTATGAGGATGGTAAGGTAGGGTAGGGTA	431540	1.017618639856972	No Hit
TAAGGATTTAAGAAAGGTTCTTATGAGGATGGTAAGGTAGGGTAGGGTAA	336058	0.7924616139246752	No Hit
GGGTAGGGTAACTCCAGGCACTTACTAGCTGTTGTCCTGAATTAATTTGT	295707	0.697309531297645	No Hit
GCTGGTGCTAAGTGCATGTTGGGCGAAGGTAAGTGGTATGGAGATGATAG	295364	0.6965006996865059	No Hit
GCTTGGTGTTTTGGTGTATTCGAGCTGCTTTAATAAACTTCTTTATGAGA	260467	0.6142097471094822	No Hit
GGGGGATTAGAAAAAGTATAACTCTTTACTTTGTGTCATCCTAGGCTGTC	233501	0.5506209621941022	No Hit
CTCTGCCTTTTTTCCTCTATACTCCTATCTCTTTAACAGCACTCATTGAT	197776	0.46637749482400825	No Hit
CCTCTGCCTTTTTTCCTCTATACTCCTATCTCTTTAACAGCACTCATTGA	185449	0.43730907712572564	No Hit
GTCGTTTGTAGTTCCTTTAAGGTCTTAACAGAAGTTCGGTCGTCTTTGTC	185197	0.436714833493052	No Hit
GTTGCCGAGAGCGAGTGTGATTGATGTAGGAATCGCTGCACGTTTAGATG	169339	0.3993199306083789	No Hit
GTCAGATCTGTATCTATTATTGTTGAAGATATCTAGGGAGTGAGTGCATA	169282	0.3991855183581312	No Hit

Sequence	Count	Percentage	Possible Source
CCCGCCCGTACCATATTAGCCGTTTTATTGACAATTCTATAAAGAGCGAT	165830	0.3910453238343646	No Hit
GGGGATTAGAAAAAGTATAACTCTTTACTTTGTGTCATCCTAGGCTGTCT	164604	0.38815428139921454	No Hit
GGCCACTTTTCCTGAACAAAGCTCAAGGATACAGAGATTATGTCAATACA	156744	0.36961953952296717	No Hit
GTCCTGAATTAATTTGTGGTTTGATTGAGCATCCAAGCCATTAGCCGTCT	154151	0.36350496119152825	No Hit
GCCACTTTTCCTGAACAAAGCTCAAGGATACAGAGATTATGTCAATACAT	152731	0.3601564454836057	No Hit
CCCGTAGTTCACCTACCAGAACCTTTTCACTGGCTATACAGCAACACTCT	151003	0.35608163200241544	No Hit
CTCCTTCCTAATATCTTTCGTTTATGTAGTCGATTTAAGAGTTAGATACC	150925	0.35589769944944505	No Hit
GGCTTGGTGTTTTGGTGTATTCGAGCTGCTTTAATAAACTTCTTTATGAG	138651	0.3269542615627961	No Hit
CGGGCTTGGTGTTTTGGTGTATTCGAGCTGCTTTAATAAACTTCTTTATG	135626	0.3198209798610597	No Hit
GTTTGATTGAGCATCCAAGCCATTAGCCGTCTAGTTGGTCAGATCTGTAT	134876	0.31805239762095977	No Hit
CTGGTGCTAAGTGCATGTTGGGCGAAGGTAAGTGGTATGGAGATGATAGT	132144	0.31161004204768905	No Hit
CCTGAATTAATTTGTGGTTTGATTGAGCATCCAAGCCATTAGCCGTCTAG	126711	0.29879843230040504	No Hit
CTTGGTGTTTTGGTGTATTCGAGCTGCTTTAATAAACTTCTTTATGAGAC	124246	0.2929856920046099	No Hit
GGGGTCGTTTGTAGTTCCTTTAAGGTCTTAACAGAAGTTCGGTCGTCTTT	123775	0.29187502235782714	No Hit
GTCGATTTAAGAGTTAGATACCTAAACGGGCTTGGTGTTTTGGTGTATTC	120891	0.28507423411722954	No Hit
GTGGTGTAAGGATTTAAGAAAGGTTCTTATGAGGATGGTAAGGTAGGGTA	117071	0.27606625524098716	No Hit
CCCCGCCCGTACCATATTAGCCGTTTTATTGACAATTCTATAAAGAGCGA	115330	0.2719607863343018	No Hit
GCCTCTGCCTTTTTTCCTCTATACTCCTATCTCTTTAACAGCACTCATTG	112958	0.26636735023627905	No Hit
GTGCTGGTGCTAAGTGCATGTTGGGCGAAGGTAAGTGGTATGGAGATGAT	111966	0.2640281054600402	No Hit
GGTGTAAGGATTTAAGAAAGGTTCTTATGAGGATGGTAAGGTAGGGTAGG	110777	0.26122431308206845	No Hit
CCCTGAGTTAGTTGGGATATACTCCACAAGCTAGAGCTTAATTCCTTCTA	108406	0.2556332350936991	No Hit
GCACGTTTAGATGTGGATAGTGGTGTAAGGATTTAAGAAAGGTTCTTATG	106583	0.25133440119542955	No Hit
GCCGTCTAGTTGGTCAGATCTGTATCTATTATTGTTGAAGATATCTAGGG	103044	0.2429890511318113	No Hit
CTGGTAATCAAATACGAATGCCCCGGTATTATCCTATCCGTAGCCCTTGT	102853	0.24253865218799917	No Hit
CCGTAGTTCACCTACCAGAACCTTTTCACTGGCTATACAGCAACACTCTA	102372	0.24140440144468173	No Hit
GTGTAAGGATTTAAGAAAGGTTCTTATGAGGATGGTAAGGTAGGGTAGGG	95561	0.2253433165949208	No Hit
CCTAGAGGGACCACAGCATGGCCTCCAATAATTAATGCTCGCTGGCCGCC	94990	0.22399683598279138	No Hit
GTGGATAGTGGTGTAAGGATTTAAGAAAGGTTCTTATGAGGATGGTAAGG	94310	0.22239332141843407	No Hit
GGGCTTGGTGTTTTGGTGTATTCGAGCTGCTTTAATAAACTTCTTTATGA	93233	0.21985363732165056	No Hit
GTCGGTTGTAAAGTGGTATAAGACTTGTCGTAATAGGCAAATAATTAAGA	93001	0.2193065558820463	No Hit
AAGGATTTAAGAAAGGTTCTTATGAGGATGGTAAGGTAGGGTAGGGTAAC	92988	0.21927590045655124	No Hit
CGTCTTTGTCGTGGCCGGGACTAGGTTATCTAAGTAAGTATCGTTCGGGT	92236	0.21750260199714438	No Hit
GCCCATCCTAGATAAGTGGCCCAACTTATGTGCAACTTACATGGAATTTG	91031	0.2146610798647171	No Hit
GTCGGCAGAAGTTGTGAACGACGAAGCAGCCGGGTATTCGTCACTTAACG	90626	0.21370604545506316	No Hit
GTGTTGCCGAGAGCGAGTGTGATTGATGTAGGAATCGCTGCACGTTTAGA	87071	0.20532296563698943	No Hit
CTACGAGTTTGTATCTGTGCTGGTGCTAAGTGCATGTTGGGCGAAGGTAA	86350	0.20362276857684003	No Hit
CTCCATTTCCCTGAGTTAGTTGGGATATACTCCACAAGCTAGAGCTTAAT	85501	0.20162073348104687	No Hit
CCCCTCCTGGCAGCAGGCCCCGCTCCGACCACGTGGAAAGCGCATAGCTC	85163	0.20082369241817521	No Hit

Sequence	Count	Percentage	Possible Source
CGCTGCACGTTTAGATGTGGATAGTGGTGTAAGGATTTAAGAAAGGTTCT	82141	0.19369748504539916	No Hit
CCCGCACATCTATGAGTTTAGGCTTGCATTACCCACCCGTTGTTTCCCTT	78365	0.1847932629939093	No Hit
CTGTGCTGGTGCTAAGTGCATGTTGGGCGAAGGTAAGTGGTATGGAGATG	75498	0.1780325626174206	No Hit
CCCGTTGTTTCCCTTAGTATTCAGAATAGCCGACATACTTTATCTATCTT	75015	0.17689359565479623	No Hit
GTTTTGGTGTATTCGAGCTGCTTTAATAAACTTCTTTATGAGACATTATA	72206	0.17026966563820858	No Hit
CTCCTATCTCTTTAACAGCACTCATTGATGTTGAAAGTTTTAAAGGTTTA	69132	0.1630208365634523	No Hit
GGATAGTGGTGTAAGGATTTAAGAAAGGTTCTTATGAGGATGGTAAGGTA	68399	0.16129234218746127	No Hit
CCTGGATTATATAGCGCCCAATTTAGGGTAAGACCGCCACACCAGTGTGC	67458	0.1590733610035492	No Hit
GGCGAAGGTAAGTGGTATGGAGATGATAGTATAATGAACCTTTAGTAGAT	66926	0.157818846667905	No Hit
GCCGTCAGTAATCATTAGTAACAAAAATAGGGAACTTTACAGAATAAACA	66371	0.15651009581023104	No Hit
TGTAAGGATTTAAGAAAGGTTCTTATGAGGATGGTAAGGTAGGGTAGGGT	65802	0.15516833141740854	No Hit
GGCAGAAGTTGTGAACGACGAAGCAGCCGGGTATTCGTCACTTAACGATA	65306	0.1539987090292891	No Hit
GTTTGTAGTTCCTTTAAGGTCTTAACAGAAGTTCGGTCGTCTTTGTCGTG	64886	0.15300830297483314	No Hit
CCCCCTCCTGGCAGCAGGCCCCGCTCCGACCACGTGGAAAGCGCATAGCT	63086	0.14876370559859328	No Hit
GCCTAGAGGGACCACAGCATGGCCTCCAATAATTAATGCTCGCTGGCCGC	62767	0.14801146861913744	No Hit
CCCCACTAAGATGAAGTTCCGGTTGACACATTCCATCTTCATTACAAAAC	61883	0.14592689968547298	No Hit
CGGTCGTCTTTGTCGTGGCCGGGACTAGGTTATCTAAGTAAGTATCGTTC	61828	0.1457972036545323	No Hit
GTGTGTGTTGCCGAGAGCGAGTGTGATTGATGTAGGAATCGCTGCACGTT	61156	0.14421255396740276	No Hit
CTGCCTTTTTTCCTCTATACTCCTATCTCTTTAACAGCACTCATTGATGT	60964	0.14375979691393717	No Hit
CCGTTGTTTCCCTTAGTATTCAGAATAGCCGACATACTTTATCTATCTTT	60650	0.14301935048274866	No Hit
CCGTCTAGTTGGTCAGATCTGTATCTATTATTGTTGAAGATATCTAGGGA	60314	0.1422270256391839	No Hit
GGGGGTCGTTTGTAGTTCCTTTAAGGTCTTAACAGAAGTTCGGTCGTCTT	59222	0.13965196989759837	No Hit
GTCTAGTTGGTCAGATCTGTATCTATTATTGTTGAAGATATCTAGGGAGT	58335	0.13756032663497353	No Hit
CTGCACGTTTAGATGTGGATAGTGGTGTAAGGATTTAAGAAAGGTTCTTA	56763	0.13385337825972404	No Hit
CTTCCTAATATCTTTCGTTTATGTAGTCGATTTAAGAGTTAGATACCTAA	56210	0.132549343621357	No Hit
CTGAATTAATTTGTGGTTTGATTGAGCATCCAAGCCATTAGCCGTCTAGT	55636	0.1311957886802672	No Hit
CCCCGGTATTATCCTATCCGTAGCCCTTGTGAGGTCTACAGCGTGACGTT	55325	0.1304624165780391	No Hit
CCGAGAGCGAGTGTGATTGATGTAGGAATCGCTGCACGTTTAGATGTGGA	54618	0.12879523305303822	No Hit
CTGGATTATATAGCGCCCAATTTAGGGTAAGACCGCCACACCAGTGTGCA	54198	0.12780482699858223	No Hit
GCTGCACGTTTAGATGTGGATAGTGGTGTAAGGATTTAAGAAAGGTTCTT	53720	0.1266776505842252	No Hit
GTCGCGGTATTCCAGCTAAGCCTAGGAAATGTTGTGGAAAAAAAGTCATA	53599	0.12639231931615574	No Hit
GGATTTAAGAAAGGTTCTTATGAGGATGGTAAGGTAGGGTAGGGTAACTC	52979	0.12493029133100646	No Hit
CCCAGTTGGAATATCCAGAAGAATTACGATCAATGTAAGCCTCTTTGTTG	52665	0.12418984489981795	No Hit
CCCCGCTCCGACCACGTGGAAAGCGCATAGCTCGAGATCATTTAAATATT	52659	0.12417569624189714	No Hit
GTTCGGGTAGCCTGACTCTCGGAAGACTTGGAGGAAAGGCGTGGCCTGGT	52127	0.12292118190625292	No Hit
CACGTTTAGATGTGGATAGTGGTGTAAGGATTTAAGAAAGGTTCTTATGA	52112	0.12288581026145093	No Hit
GCTGTCTACTCGCGCTGTGTGCTTTCAGGGGTATTCGTTGAGGCTGCAGC	52001	0.12262406008991614	No Hit
GTCCGCGGATCCTTAGGGGGTCGTTTGTAGTTCCTTTAAGGTCTTAACAG	50970	0.12019284903719209	No Hit

Sequence	Count	Percentage	Possible Source
CCGCACATCTATGAGTTTAGGCTTGCATTACCCACCCGTTGTTTCCCTTA	50686	0.11952314589560757	No Hit
TGCACGTTTAGATGTGGATAGTGGTGTAAGGATTTAAGAAAGGTTCTTAT	50682	0.1195137134569937	No Hit
CCCGTACCATATTAGCCGTTTTATTGACAATTCTATAAAGAGCGATTGCA	50137	0.11822854369585442	No Hit
CGGCACTCGAAAAATAATGGACCCCACTAAGATGAAGTTCCGGTTGACAC	49130	0.11585392727481356	No Hit
CCCGGTATTATCCTATCCGTAGCCCTTGTGAGGTCTACAGCGTGACGTTG	49030	0.11561811630946689	No Hit
CCTCCCTGGATTATATAGCGCCCAATTTAGGGTAAGACCGCCACACCAGT	48647	0.1147149603121892	No Hit
GCCCGTACCATATTAGCCGTTTTATTGACAATTCTATAAAGAGCGATTGC	48139	0.11351704060822818	No Hit
GGTTTGATTGAGCATCCAAGCCATTAGCCGTCTAGTTGGTCAGATCTGTA	47625	0.11230497224634634	No Hit
CCCTATTCTTTGATGATGTAAACGAGTGCAACATGAATGGTAATAACAAT	47419	0.11181920165773222	No Hit
GGATGGTAAGGTAGGGTAGGGTAACTCCAGGCACTTACTAGCTGTTGTCC	46314	0.10921349049065165	No Hit
GCCGGGACTAGGTTATCTAAGTAAGTATCGTTCGGGTAGATCTCGATCGG	45546	0.1074024622767893	No Hit
CCCGGACAGAGGGTTCATCCACGATGTACAAACGTGCGTACCCAAAACTA	44612	0.1051999878604515	No Hit
CACCTCTTCATGGGCCCGTCCGTGCCTCATTCCCCCTCCTGGCAGCAGGC	44472	0.10486985250896619	No Hit
CCTGAGTTAGTTGGGATATACTCCACAAGCTAGAGCTTAATTCCTTCTAG	44150	0.10411054120054994	No Hit
GGCCTCTCAAATAATGAACATTATTATAATTACGGCTACAAGGGAGATTA	43747	0.10316022301020289	No Hit
GTACGGAGGTGGGCAGCCGTGAAGTCATTCAATATTAGTACTTGTAAGTT	43247	0.10198116818346961	No Hit
CTCGTATTGAATGGAATTATTCCCCCTCTTGTGAGCAGAAGGGGAGGAAG	42969	0.10132561369980589	No Hit
GGTAGGGTAACTCCAGGCACTTACTAGCTGTTGTCCTGAATTAATTTGTG	42953	0.10128788394535043	No Hit

Adapter Content

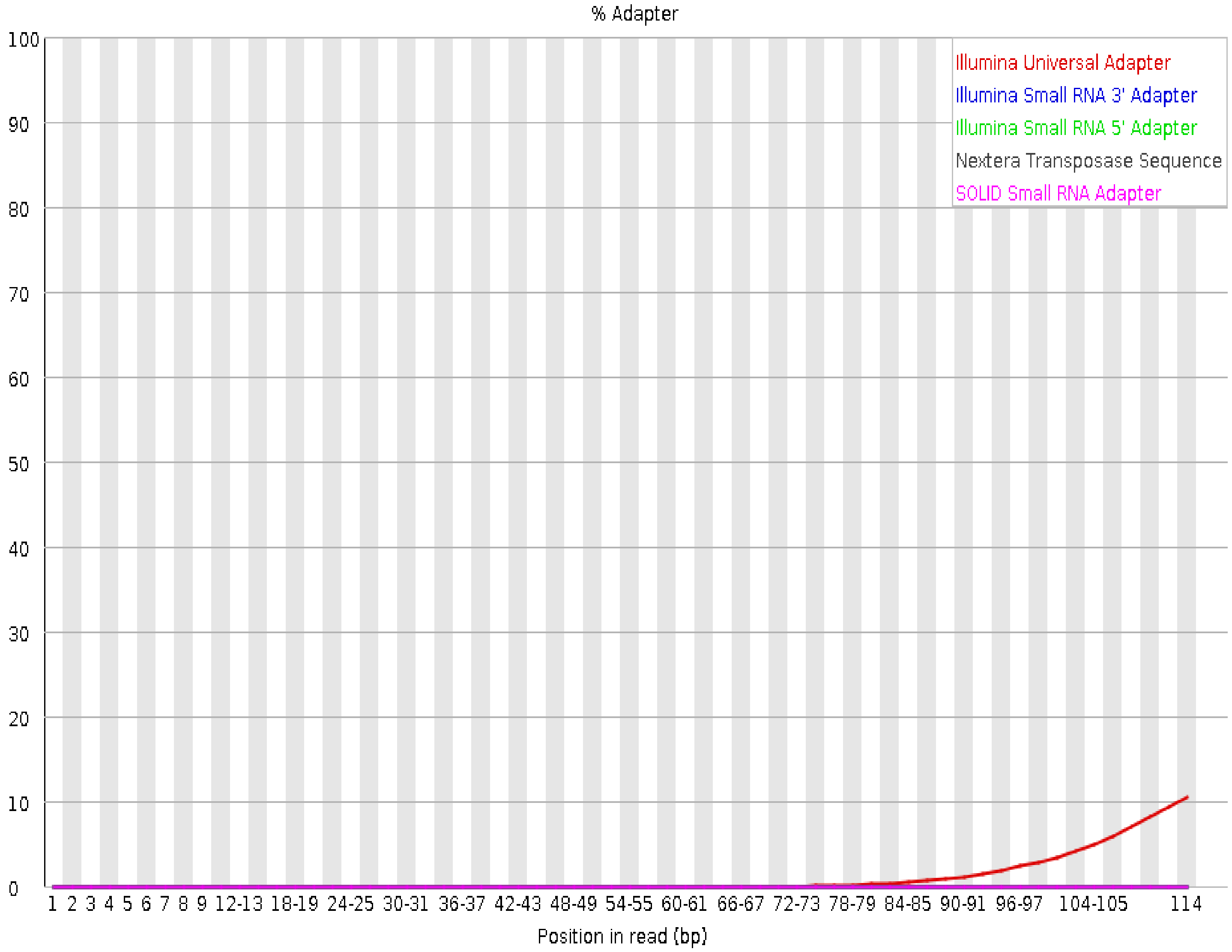

Produced by FastQC (version 0.11.8)

FastQC Report

Wed 9 Oct 2019
Eulamprus.Male.Liver_R2.fastq.gz

Summary

- Basic Statistics
- Per base sequence quality
- Per tile sequence quality
- Per sequence quality scores
- Per base sequence content
- Per sequence GC content
- Per base N content
- Sequence Length Distribution
- Sequence Duplication Levels
- Overrepresented sequences
- Adapter Content

Basic Statistics

Measure	Value
Filename	Eulamprus.Male.Liver_R2.fastq.gz
File type	Conventional base calls
Encoding	Sanger / Illumina 1.9
Total Sequences	42406849
Sequences flagged as poor quality	0
Sequence length	125
%GC	44

Per base sequence quality

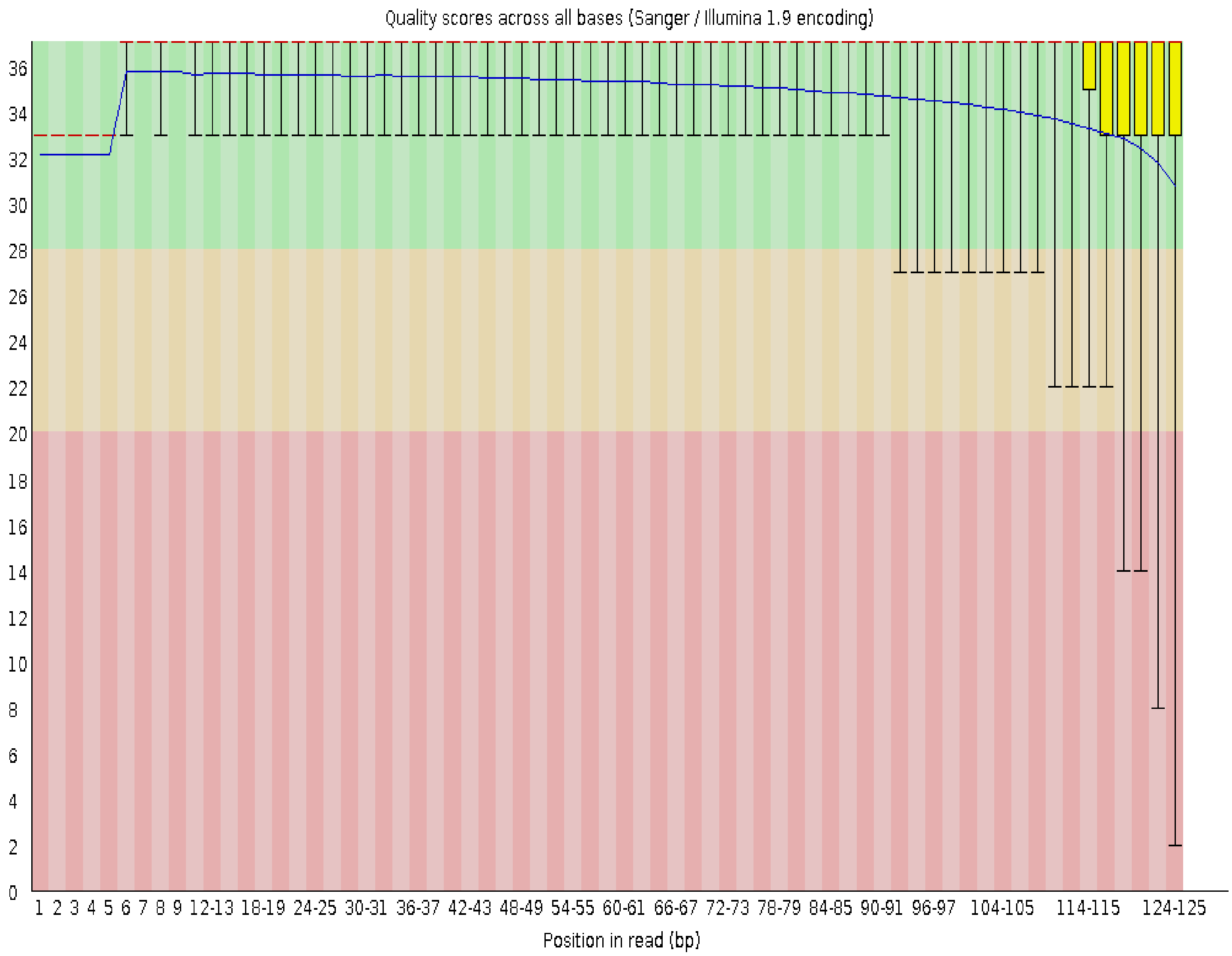

Per tile sequence quality

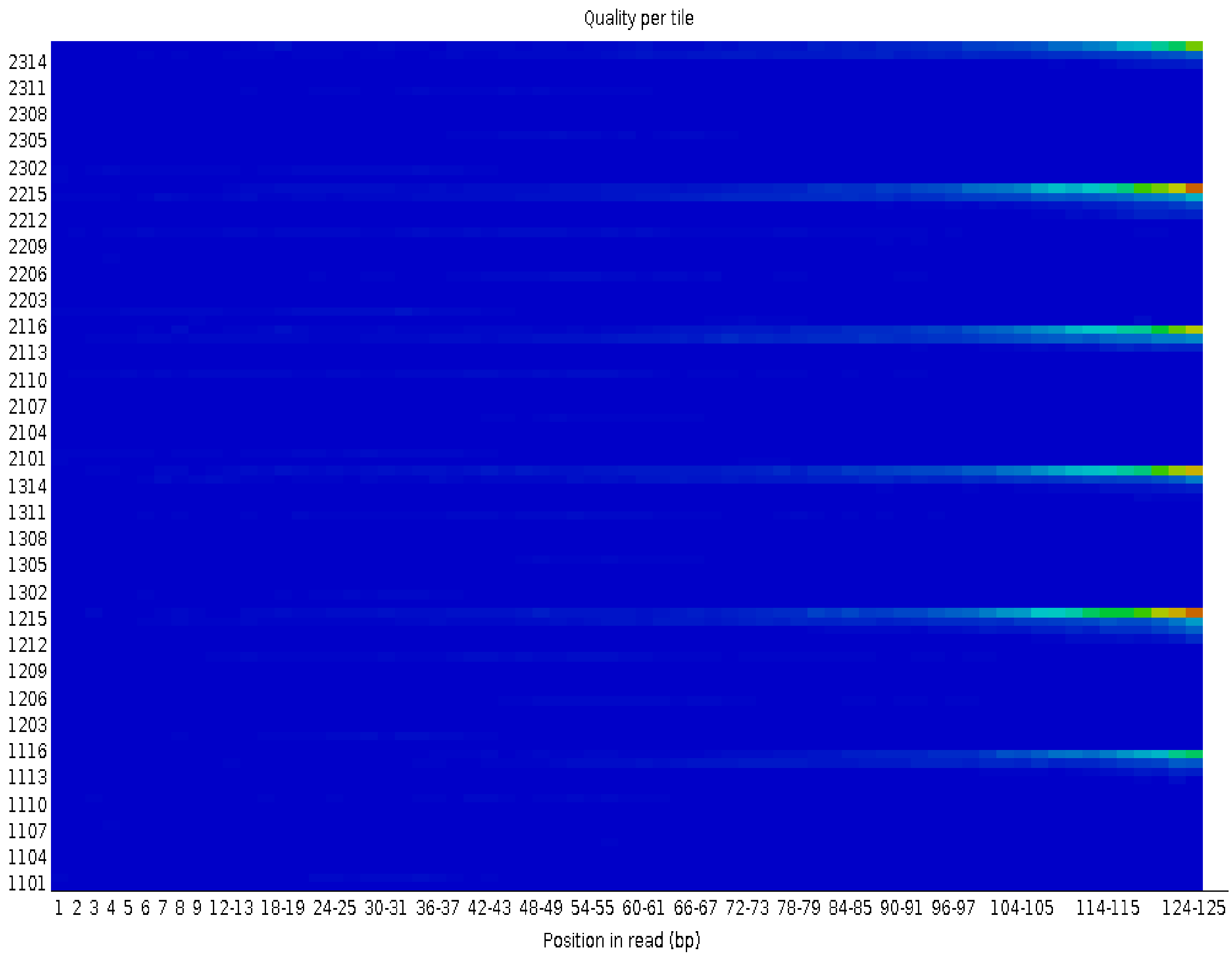

Per sequence quality scores

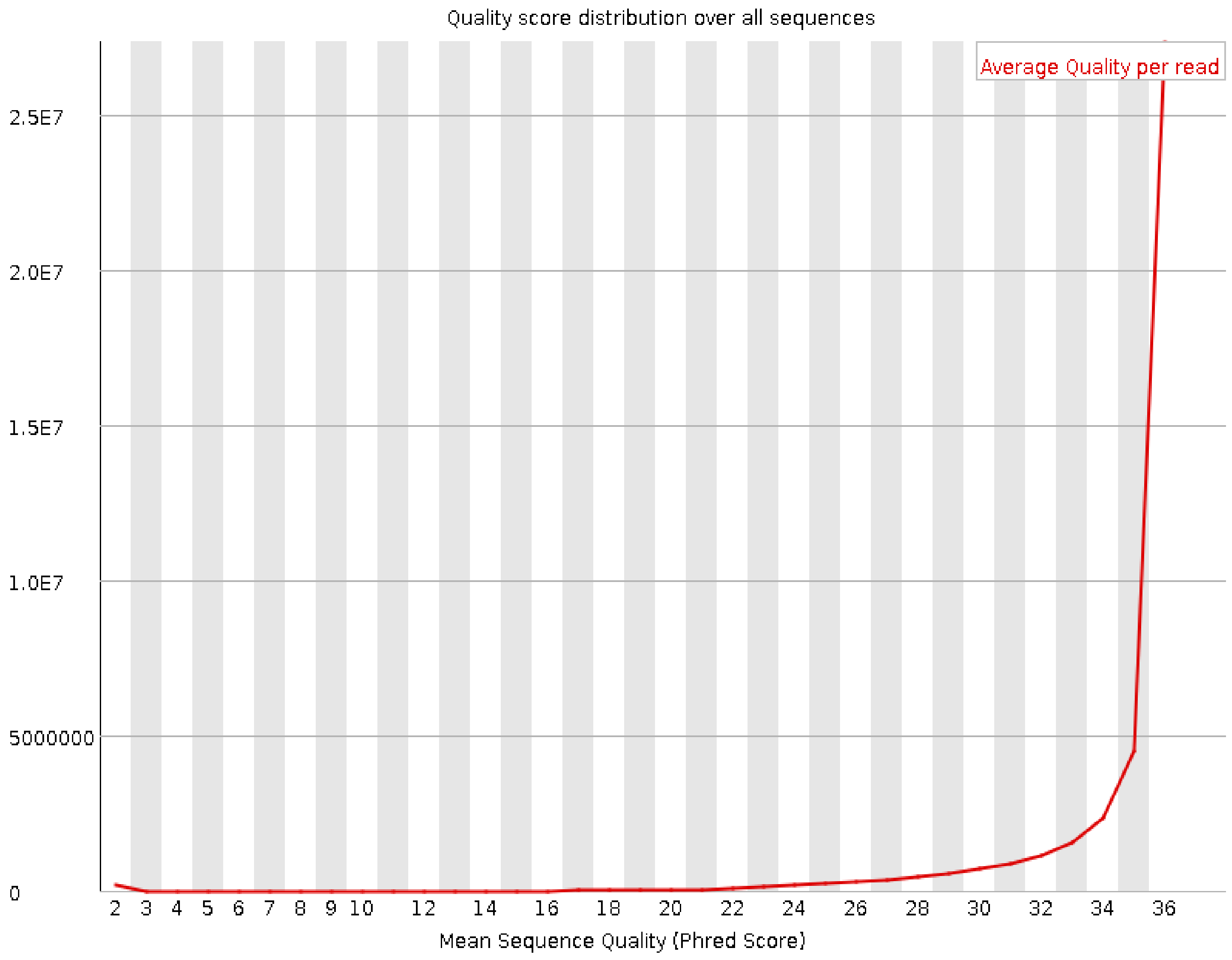

Per base sequence content

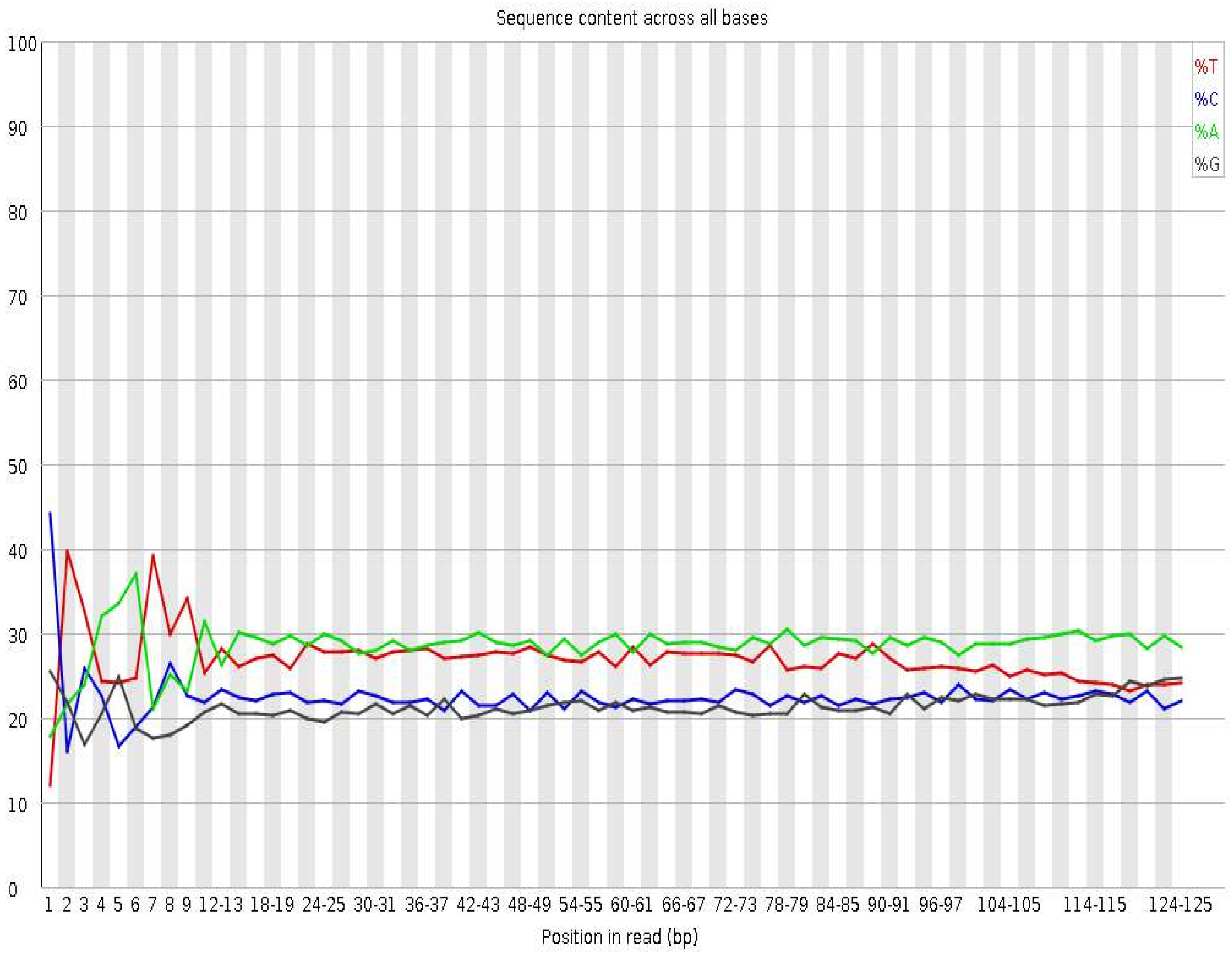

Per sequence GC content

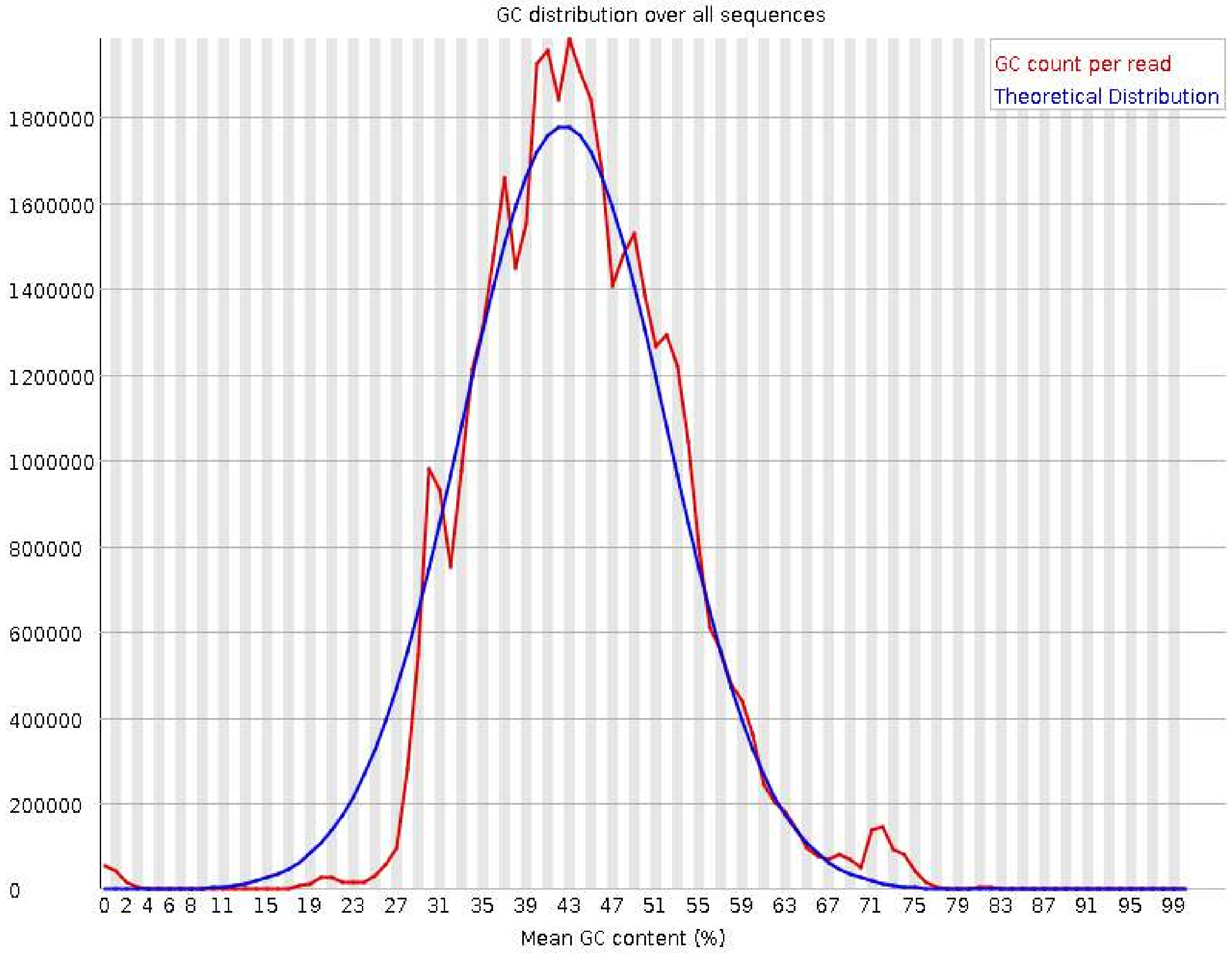

Per base N content

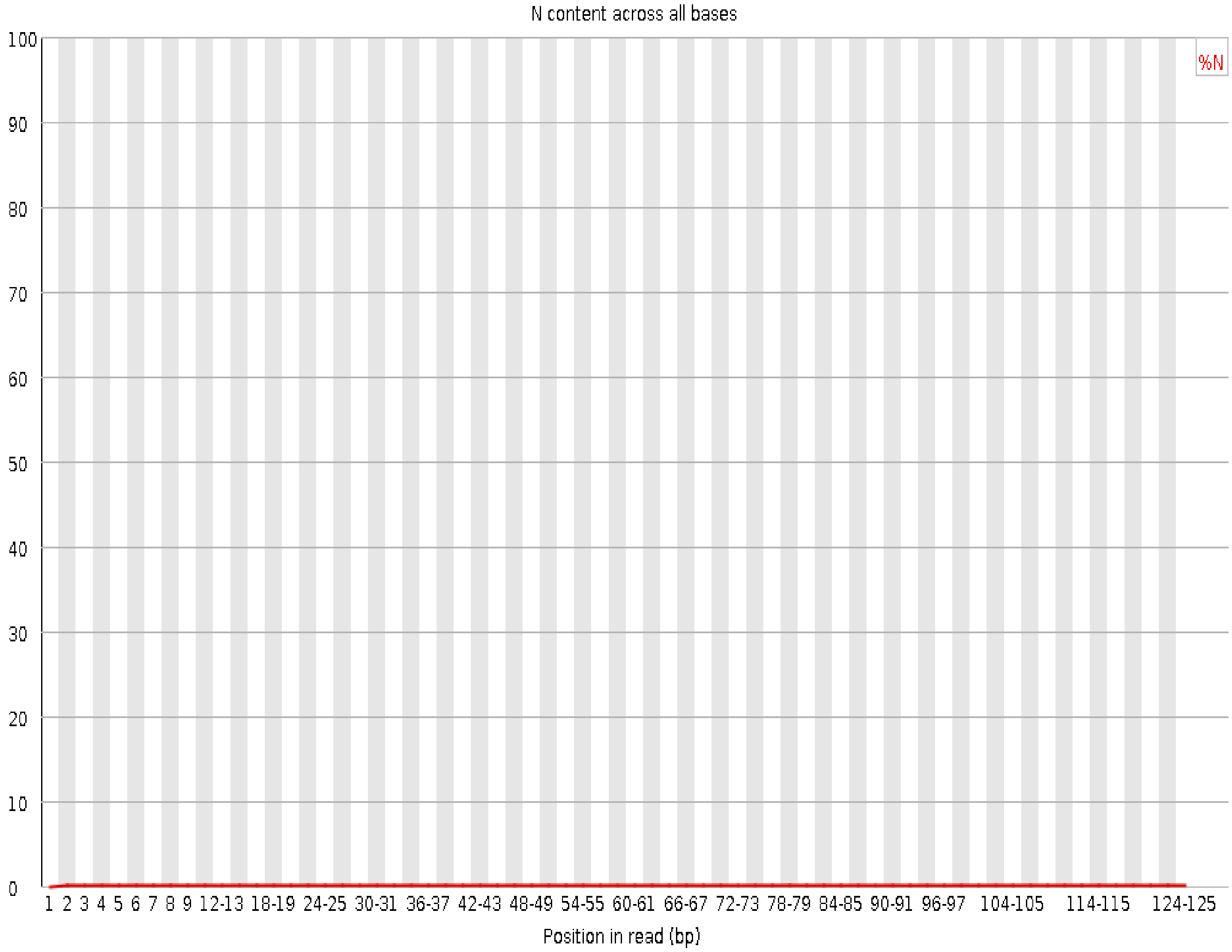

Sequence Length Distribution

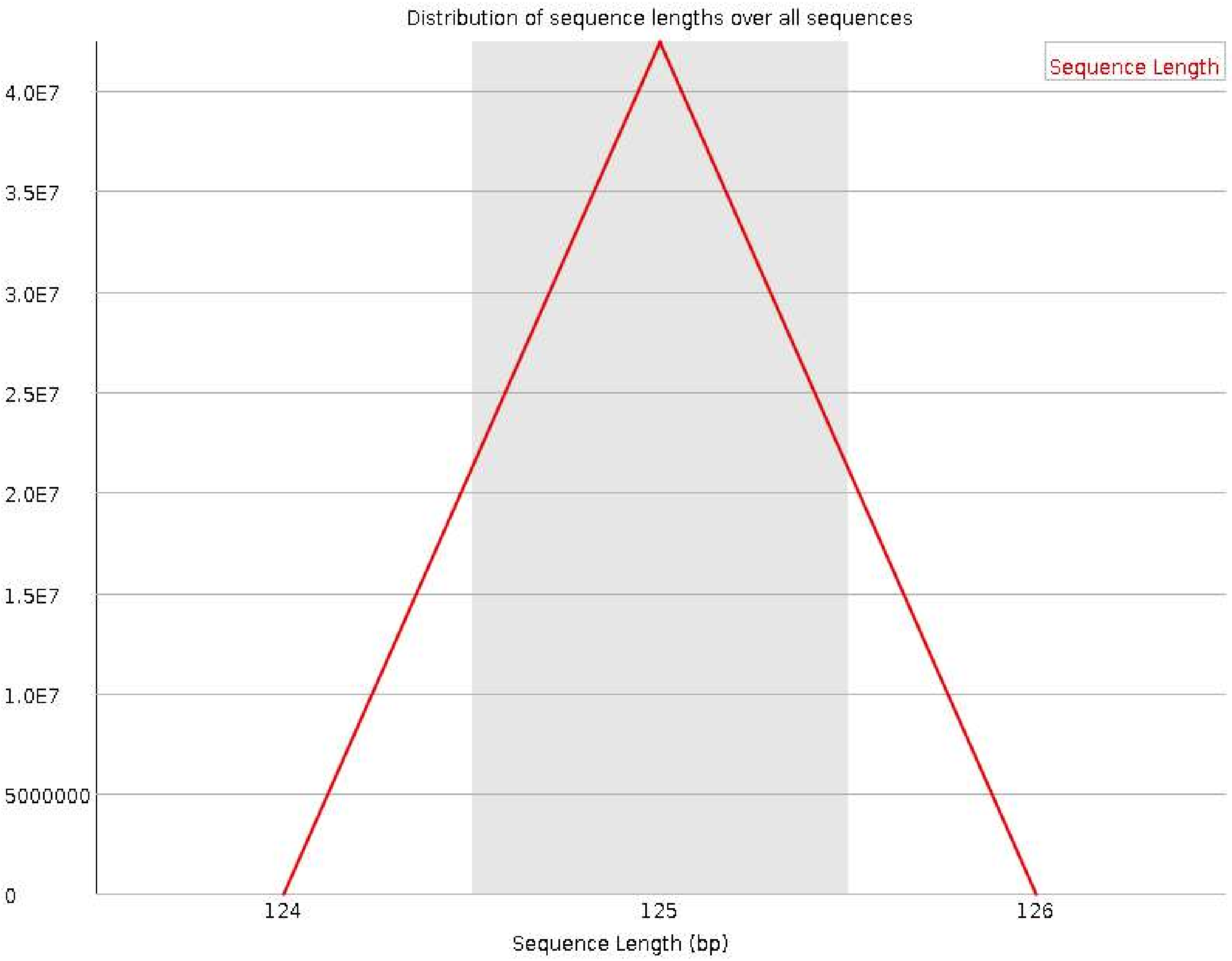

Sequence Duplication Levels

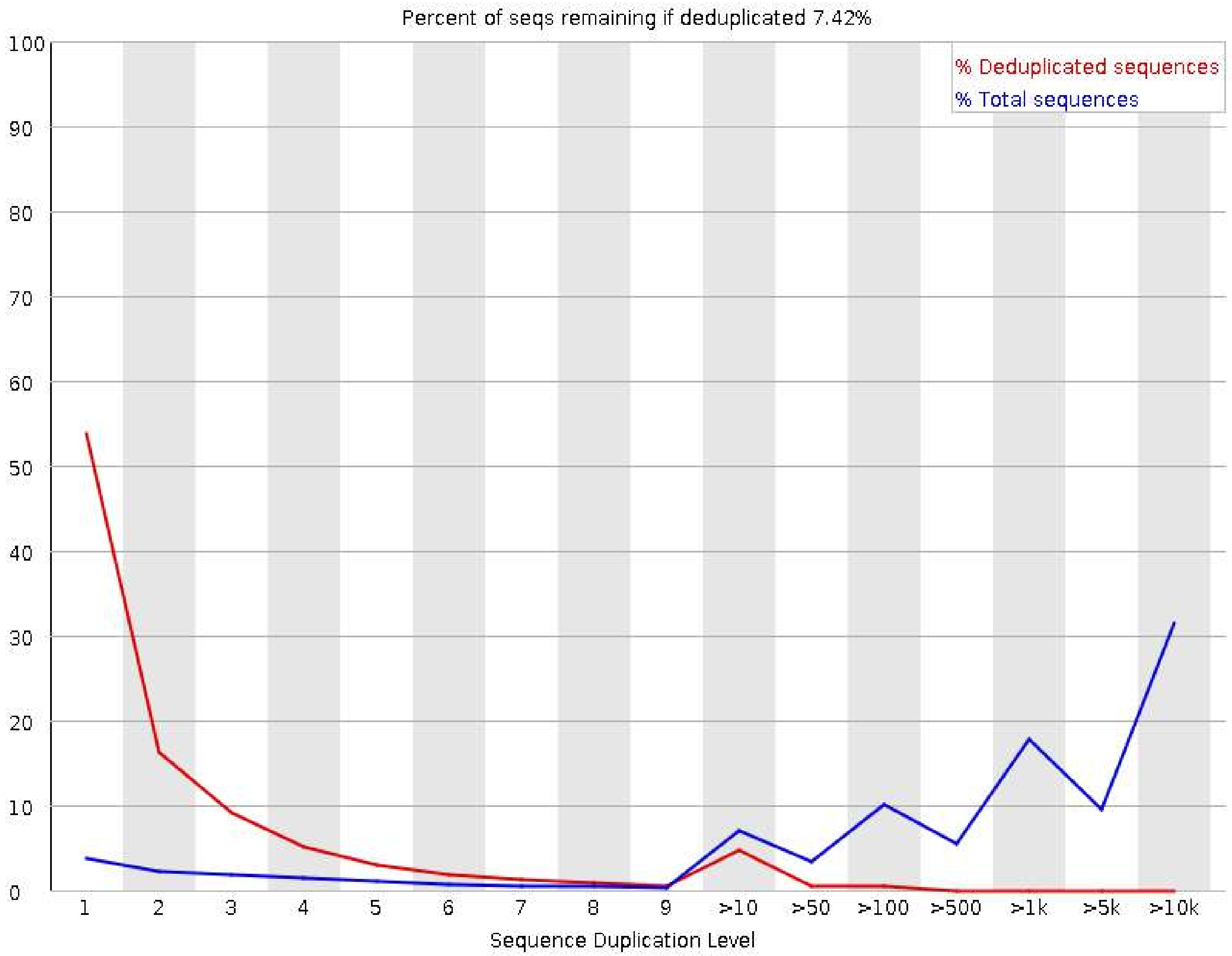

Overrepresented sequences

Sequence	Count	Percentage	Possible Source
CTCAGTTCTCTTGAAACCTACATAAATTCGCAATTATGACAATATATTAC	837587	1.9751219903181203	No Hit
CTTCAACAATAATAGATACAGATCTGACCAACTAGACGGCTAATGGCTTG	352073	0.8302267400249426	No Hit
CTTTTATATTGTTCAACTTCTCTCTTAATAGCTATGCACTCACTCCCTAG	174098	0.41054217444922636	No Hit
GTTCTCTTGAAACCTACATAAATTCGCAATTATGACAATATATTACTAGC	142999	0.33720732233606887	No Hit
GTCGATTTTCACAATATACTTTTATATTGTTCAACTTCTCTCTTAATAGC	139223	0.32830310028457904	No Hit
CTCGTTTACATCATCAAAGAATAGGGGTACTTCCTATTGAAGAGCTGTAT	125787	0.2966195389806019	No Hit
GTCTAGTTCTTTATATAATCACATTCTTTTTCGTCCTTTATATACGCTAC	121224	0.28585948463183386	No Hit
GTTCTTTATATAATCACATTCTTTTTCGTCCTTTATATACGCTACCGTGC	99135	0.23377120049641037	No Hit
CTTGAAACCTACATAAATTCGCAATTATGACAATATATTACTAGCTAAAG	97461	0.22982372493650732	No Hit
TGGAGCTAGAAGGAATTAAGCTCTAGCTTGTGGAGTATATCCCAACTAAC	91429	0.21559960750679683	No Hit
CTAGTTCTTTATATAATCACATTCTTTTTCGTCCTTTATATACGCTACCG	91010	0.21461155956199432	No Hit
CAAAGAATAGGGGTACTTCCTATTGAAGAGCTGTATGTGTCGATTTTCAC	90292	0.21291843683080536	No Hit

Sequence	Count	Percentage	Possible Source
CTTCTCTCTTAATAGCTATGCACTCACTCCCTAGATATCTTCAACAATAA	89598	0.2112819087312995	No Hit
CTCACTCCCTAGATATCTTCAACAATAATAGATACAGATCTGACCAACTA	88154	0.20787679839169376	No Hit
CCTAGATATCTTCAACAATAATAGATACAGATCTGACCAACTAGACGGCT	86358	0.20364163345406777	No Hit
GGTCCACTCAGTTCTCTTGAAACCTACATAAATTCGCAATTATGACAATA	84172	0.1984868057515898	No Hit
GTTCAACTTCTCTCTTAATAGCTATGCACTCACTCCCTAGATATCTTCAA	81607	0.192438254490448	No Hit
GTGGGAATGGCATCCGGCCAACTGTAGTCGGGTTCTCTTGACTCGGAGGA	77500	0.1827534981436607	No Hit
TGTTGGCCTAACTACGAACACATCACTCATCATCAGCGCTCAGTAAAAGG	74885	0.17658704139984557	No Hit
GTTGGCCTAACTACGAACACATCACTCATCATCAGCGCTCAGTAAAAGGG	71139	0.16775356263795974	No Hit
CGGCTAATGGCTTGGATGCTCAATCAAACCACAAATTAATTCAGGACAAC	70585	0.16644716988993924	No Hit
CTAATGGCTTGGATGCTCAATCAAACCACAAATTAATTCAGGACAACAGC	70197	0.1655322233443942	No Hit
CAGTTCTCTTGAAACCTACATAAATTCGCAATTATGACAATATATTACTA	69477	0.16383438439389825	No Hit
CAGCTAGTAAGTGCCTGGAGTTACCCTACCCTACCTTACCATCCTCATAA	67116	0.15826688750206364	No Hit
TACAGATCTGACCAACTAGACGGCTAATGGCTTGGATGCTCAATCAAACC	63189	0.14900659089290036	No Hit
GTCCACTCAGTTCTCTTGAAACCTACATAAATTCGCAATTATGACAATAT	61665	0.14541283178101724	No Hit
CATCAATGAGTGCTGTTAAAGAGATAGGAGTATAGAGGAAAAAAGGCAGA	60886	0.14357586436096678	No Hit
GGGAAATGGAGAACCATAATGTAAACCTTTAAAACTTTCAACATCAATGA	58935	0.13897519242705347	No Hit
GGCTTGGATGCTCAATCAAACCACAAATTAATTCAGGACAACAGCTAGTA	55479	0.13082556546467294	No Hit
CCATAATGTAAACCTTTAAAACTTTCAACATCAATGAGTGCTGTTAAAGA	54633	0.1288306046978402	No Hit
CCCAACTAACTCAGGGAAATGGAGAACCATAATGTAAACCTTTAAAACTT	54615	0.12878815872407778	No Hit
ATCTGACCAACTAGACGGCTAATGGCTTGGATGCTCAATCAAACCACAAA	54368	0.12820570563967154	No Hit
GTATATCCCAACTAACTCAGGGAAATGGAGAACCATAATGTAAACCTTTA	54147	0.12768456340625545	No Hit
ATCCCAACTAACTCAGGGAAATGGAGAACCATAATGTAAACCTTTAAAAC	53697	0.1266234140621955	No Hit
CAACAATAATAGATACAGATCTGACCAACTAGACGGCTAATGGCTTGGAT	53580	0.12634751523273988	No Hit
CTTCGATGCCCACGCGGCTAGTCCCTGGAACACCCAGAGGACTGCCCCCA	51931	0.12245899241417348	No Hit
GGGGTACTTCCTATTGAAGAGCTGTATGTGTCGATTTTCACAATATACTT	51434	0.12128701191640057	No Hit
CTCTTCACCAAACCTGAACAAAAGTCCACTTTGGTGTAATGTTTGCAGGT	50457	0.11898313878496374	No Hit
TGTCGATCTACTCGGTGCATGGAGCTAGAAGGAATTAAGCTCTAGCTTGT	49372	0.11642458981095247	No Hit
CGCCTTTCCTCCAAGTCTTCCGAGAGTCAGGCTACCCGAACGATACTTAC	47025	0.11089010645426638	No Hit
TGGAGAACCATAATGTAAACCTTTAAAACTTTCAACATCAATGAGTGCTG	46558	0.10978886924609749	No Hit
GATATCTTCAACAATAATAGATACAGATCTGACCAACTAGACGGCTAATG	46385	0.10938091627604776	No Hit
CACTCAGTTCTCTTGAAACCTACATAAATTCGCAATTATGACAATATATT	46299	0.10917811884584964	No Hit
CTTCTGGTGGATCTCGATATATCATATATTATCTCAAGTGATGTAGTAGT	46209	0.10896588897703764	No Hit
CACAAATTAATTCAGGACAACAGCTAGTAAGTGCCTGGAGTTACCCTACC	45180	0.10653939414362053	No Hit
CTCACAAGGGCTACGGATAGGATAATACCGGGGCATTCGTATTTGATTAC	45094	0.1063365967134224	No Hit
TATCAATGTCGATCTACTCGGTGCATGGAGCTAGAAGGAATTAAGCTCTA	44848	0.10575650173866961	No Hit
CGTTAAGTGACGAATACCCGGCTGCTTCGTCGTTCACAACTTCTGCCGAC	44830	0.10571405576490721	No Hit
GCTCAATCAAACCACAAATTAATTCAGGACAACAGCTAGTAAGTGCCTGG	43890	0.10349743269064862	No Hit
GTAGTATCAATGTCGATCTACTCGGTGCATGGAGCTAGAAGGAATTAAGC	43665	0.10296685801861866	No Hit

Sequence	Count	Percentage	Possible Source
GCTGTATGTGTCGATTTTCACAATATACTTTTATATTGTTCAACTTCTCT	42660	0.10059695781688473	No Hit

Adapter Content

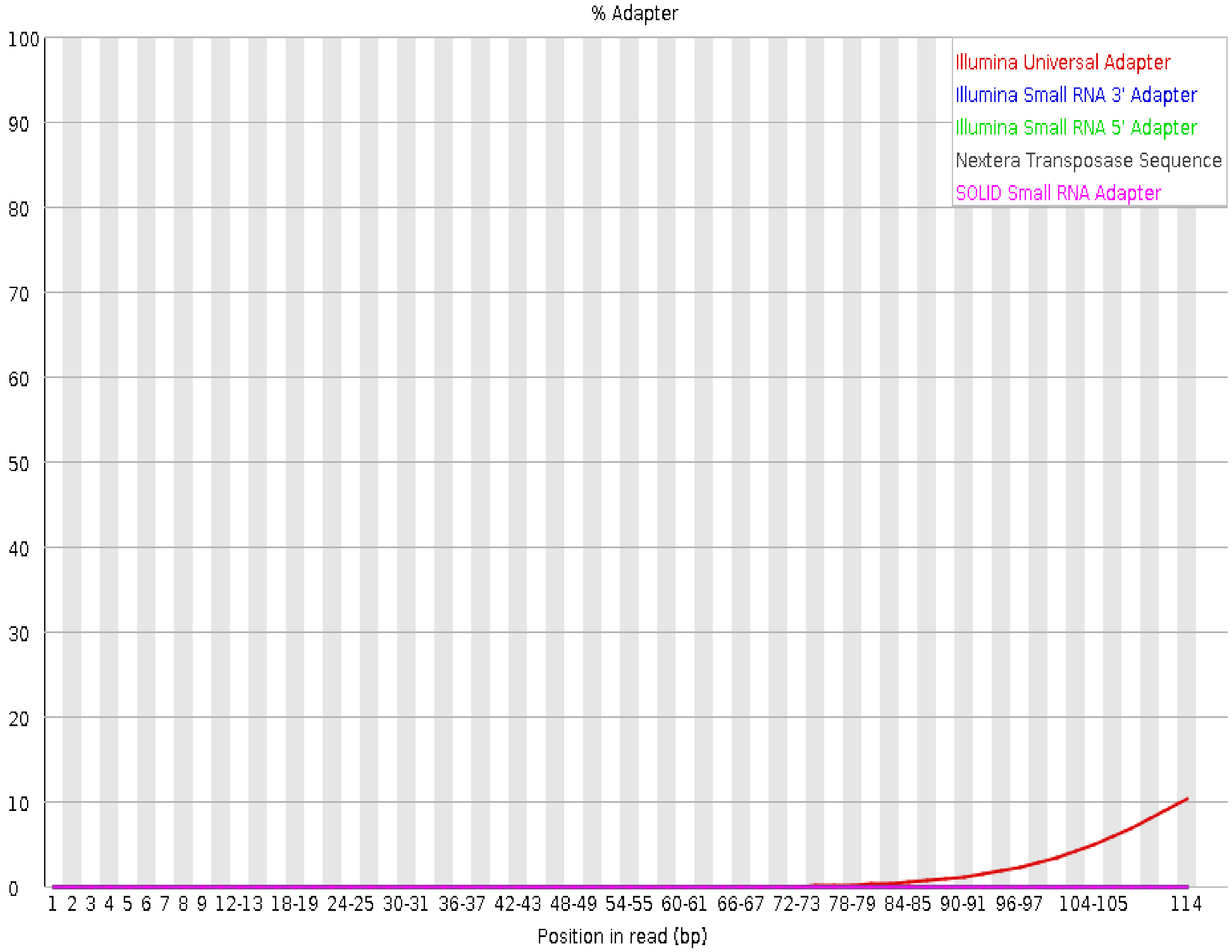

Produced by FastQC (version 0.11.8)

FastQC Report

Wed 9 Oct 2019
Eulamprus.Male.Testis_R1.fastq.gz

Summary

- Basic Statistics
- Per base sequence quality
- Per tile sequence quality
- Per sequence quality scores
- Per base sequence content
- Per sequence GC content
- Per base N content
- Sequence Length Distribution
- Sequence Duplication Levels
- Overrepresented sequences
- Adapter Content

Basic Statistics

Measure	Value
Filename	Eulamprus.Male.Testis_R1.fastq.gz
File type	Conventional base calls
Encoding	Sanger / Illumina 1.9
Total Sequences	43748899
Sequences flagged as poor quality	0
Sequence length	125
%GC	46

Per base sequence quality

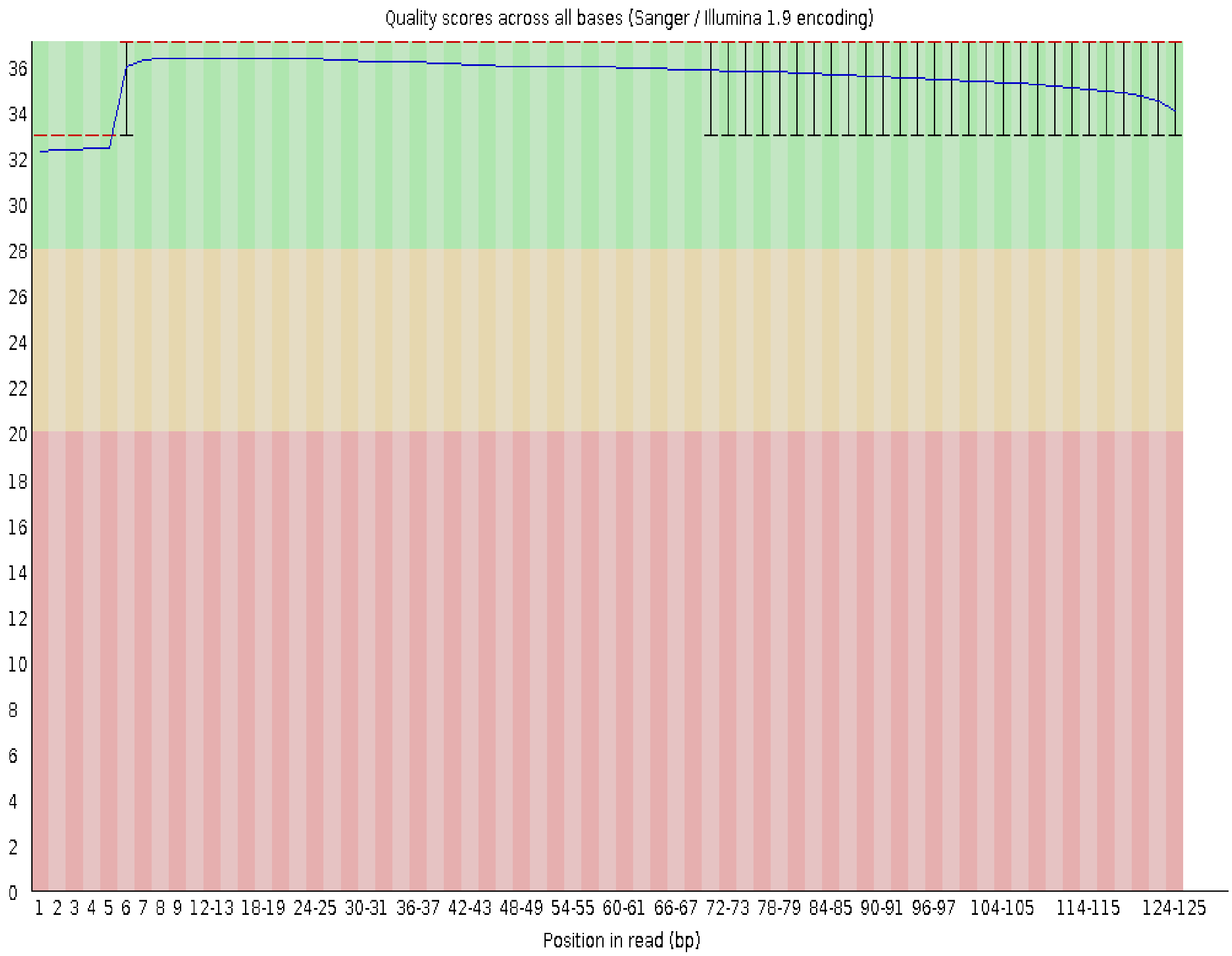

Per tile sequence quality

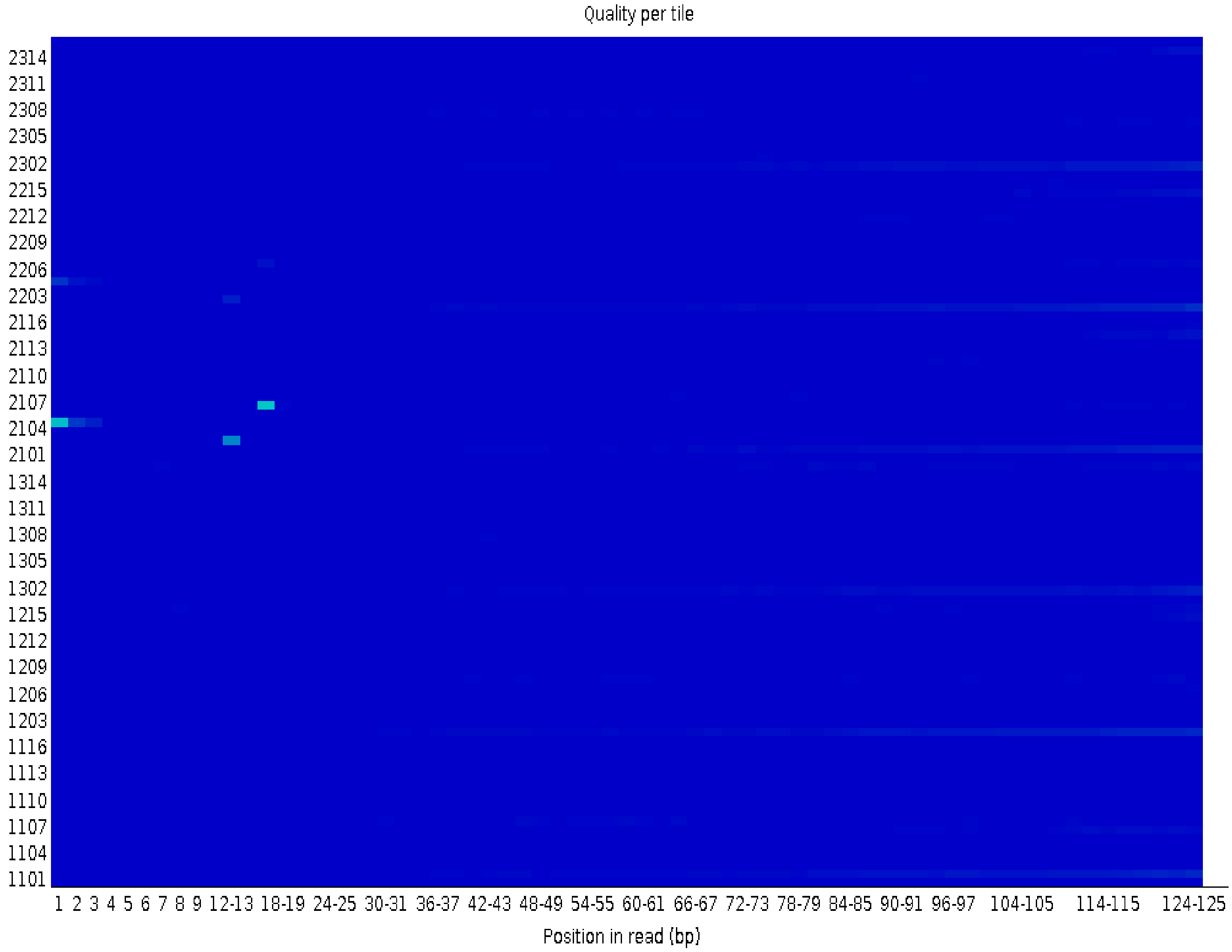

Per sequence quality scores

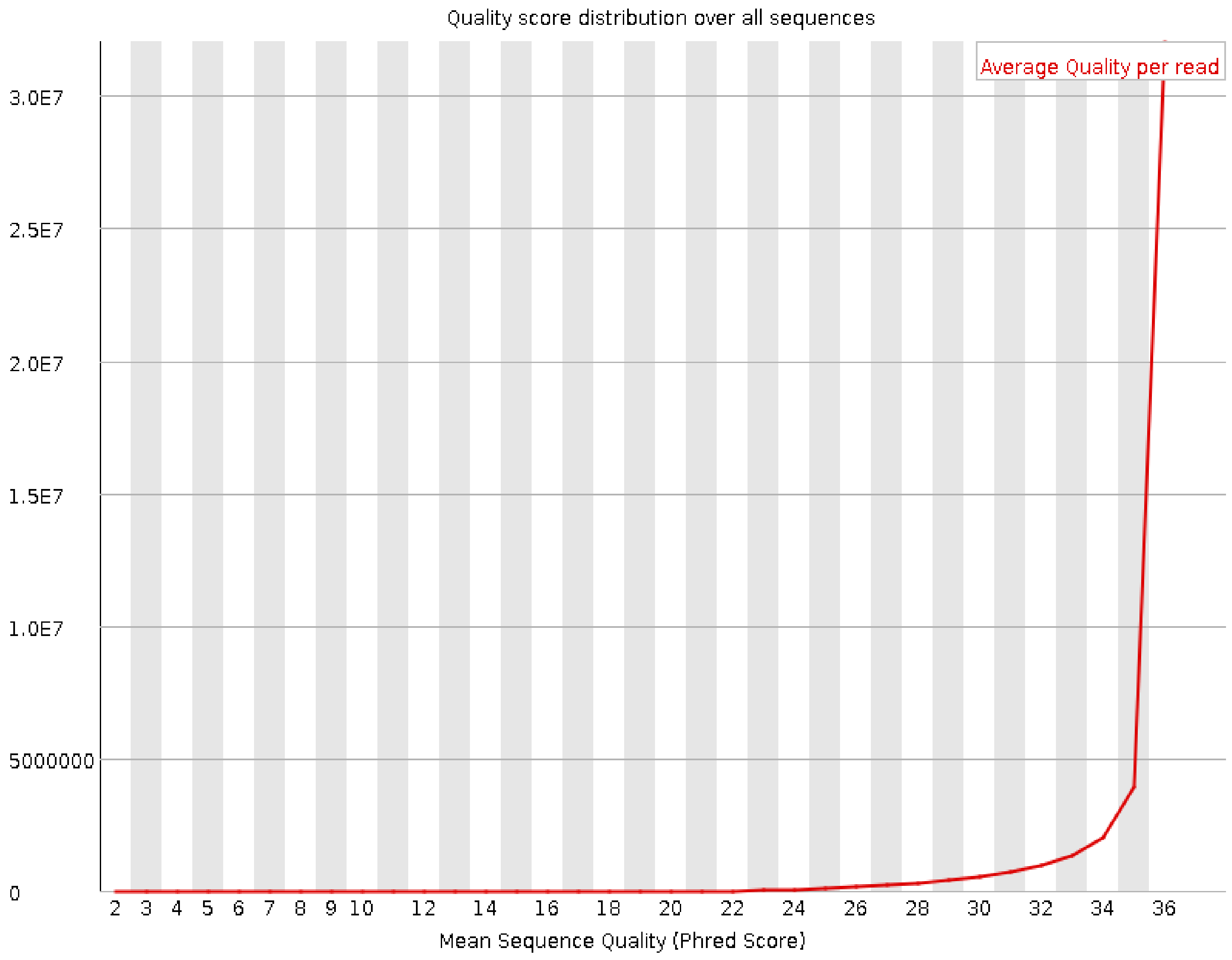

Per base sequence content

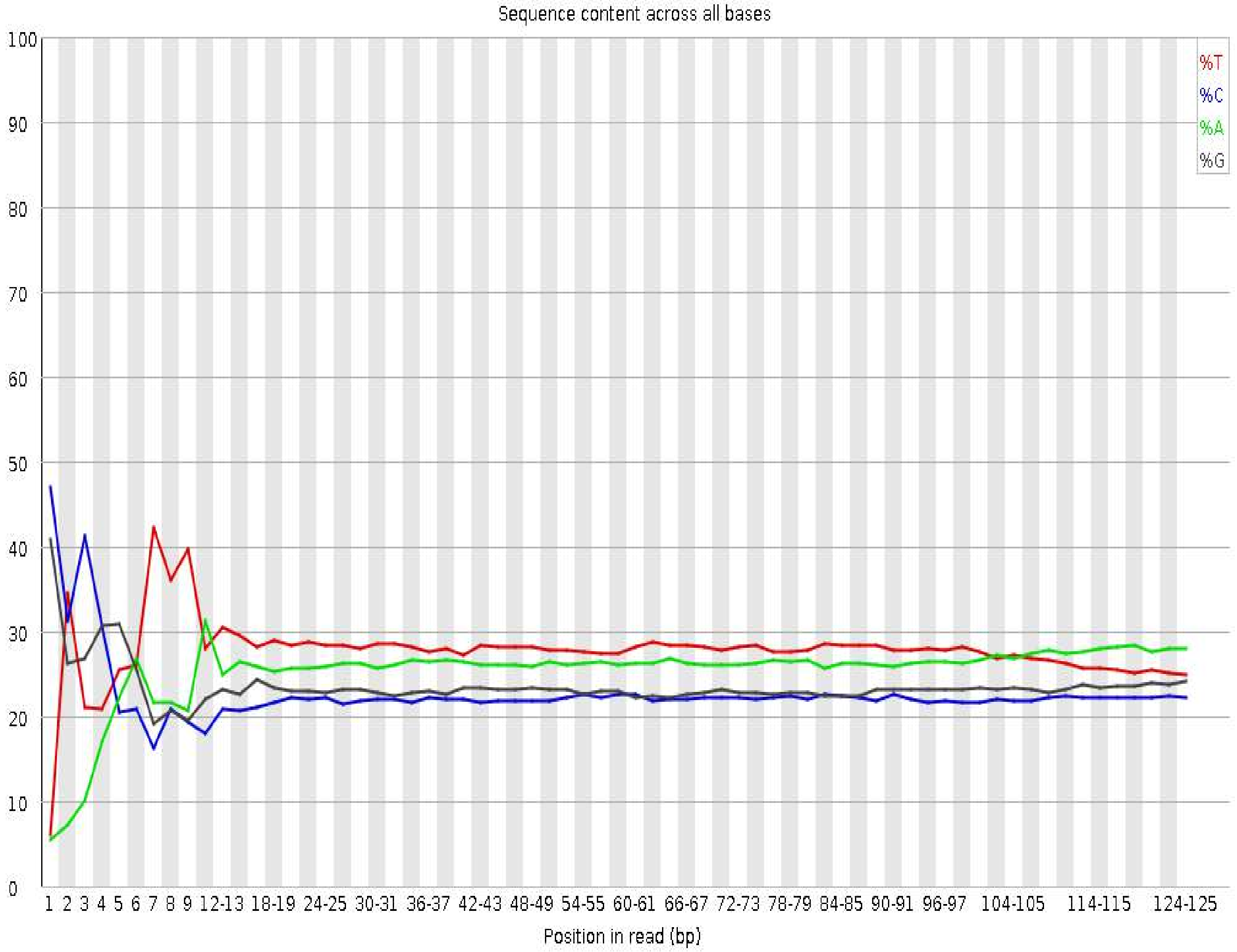

Per sequence GC content

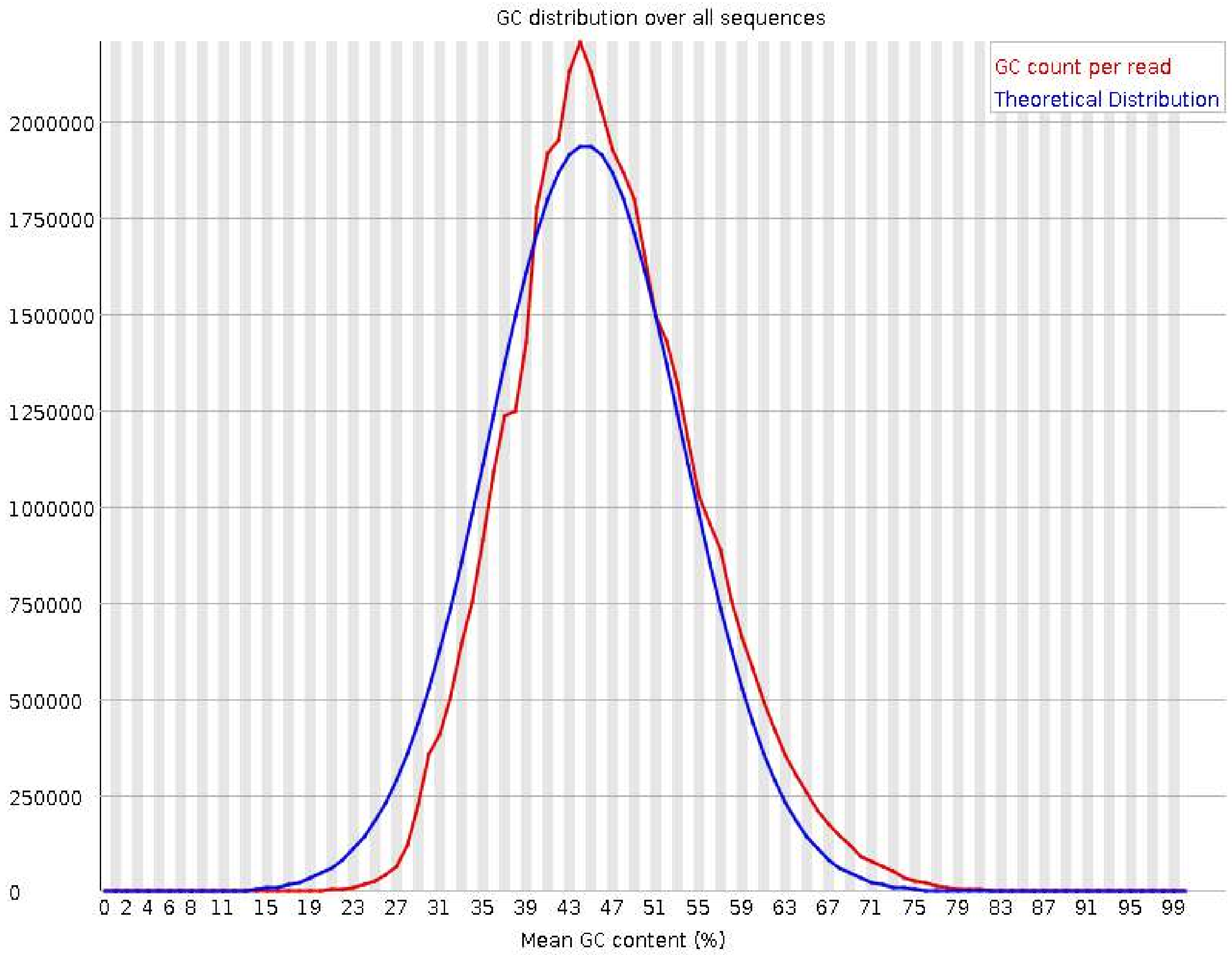

Per base N content

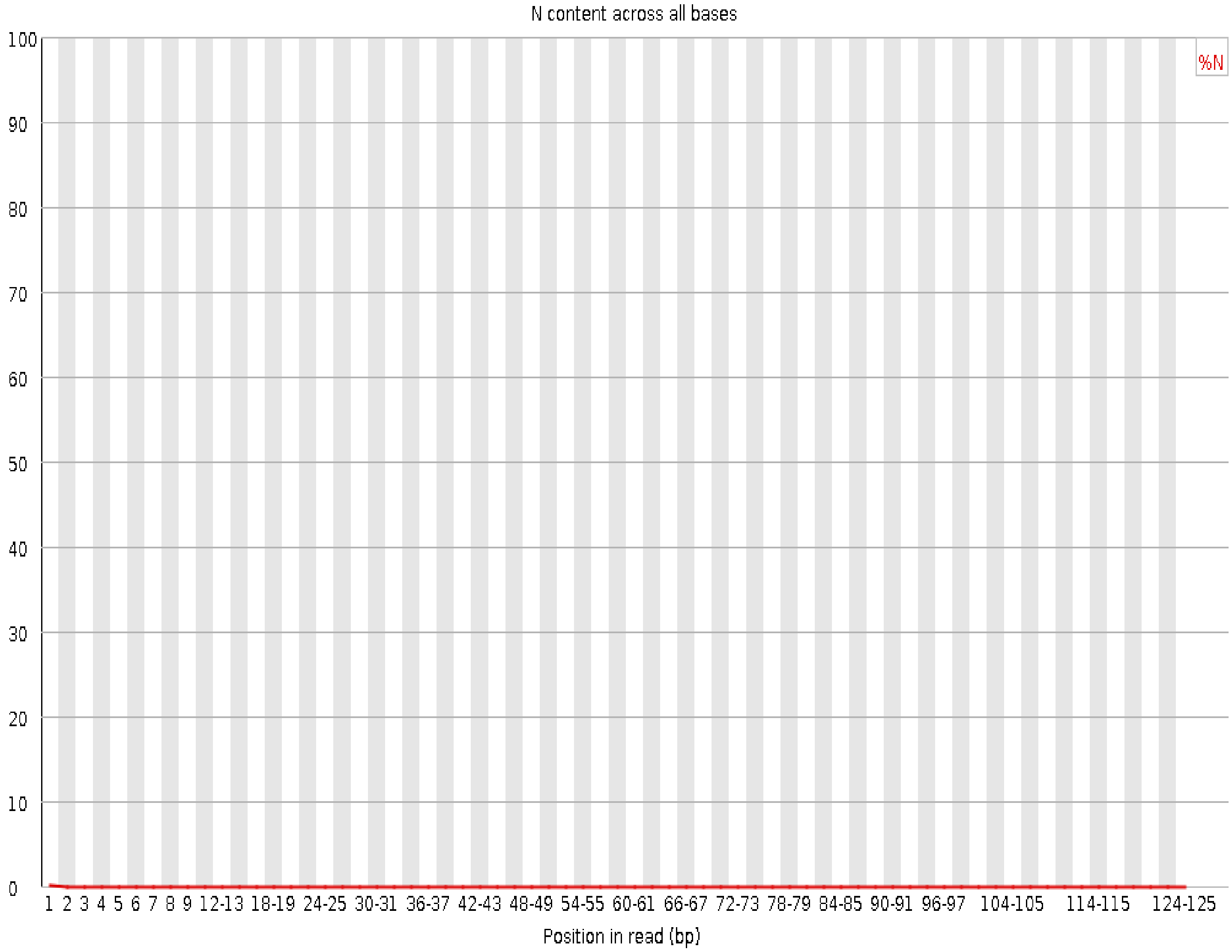

Sequence Length Distribution

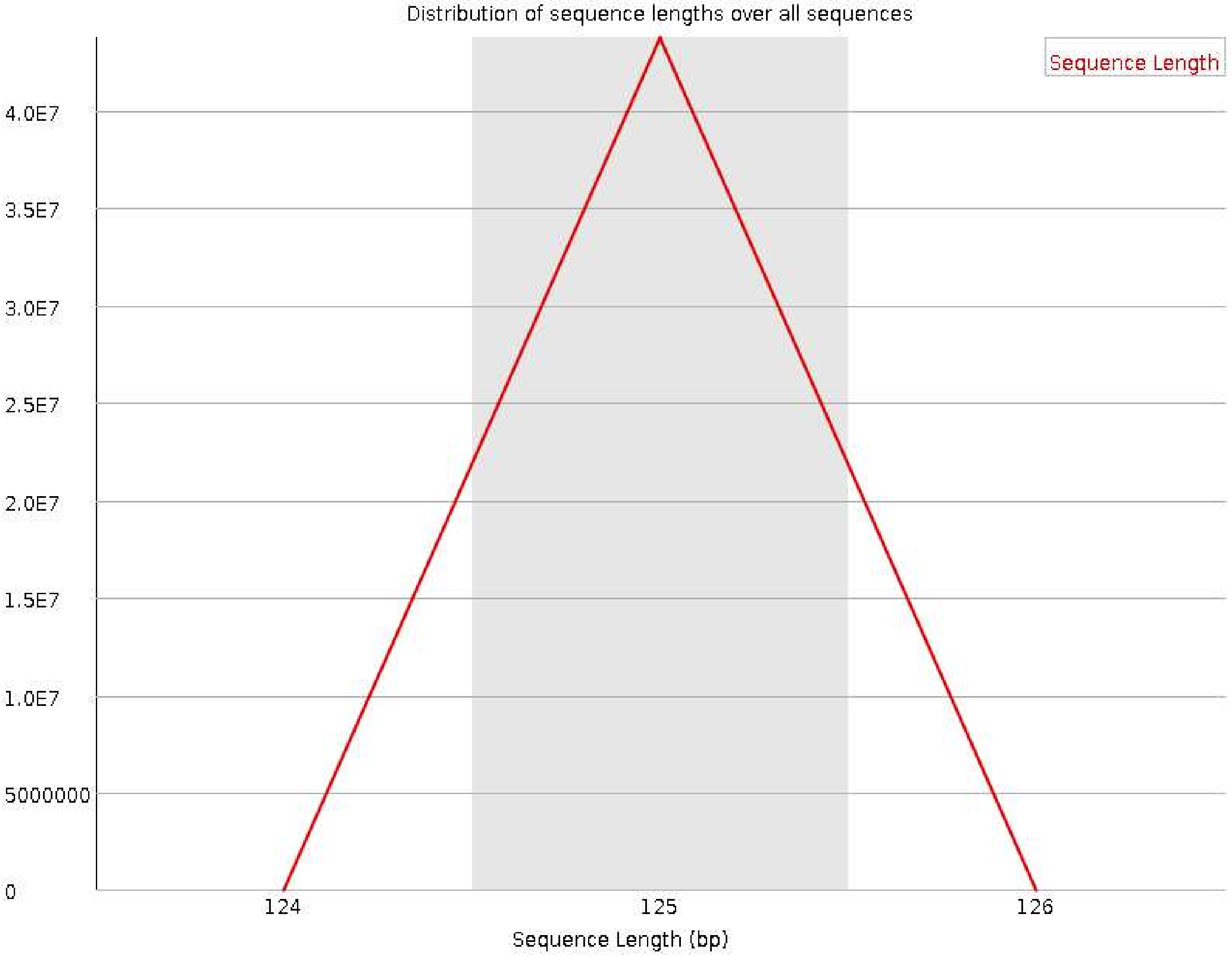

Sequence Duplication Levels

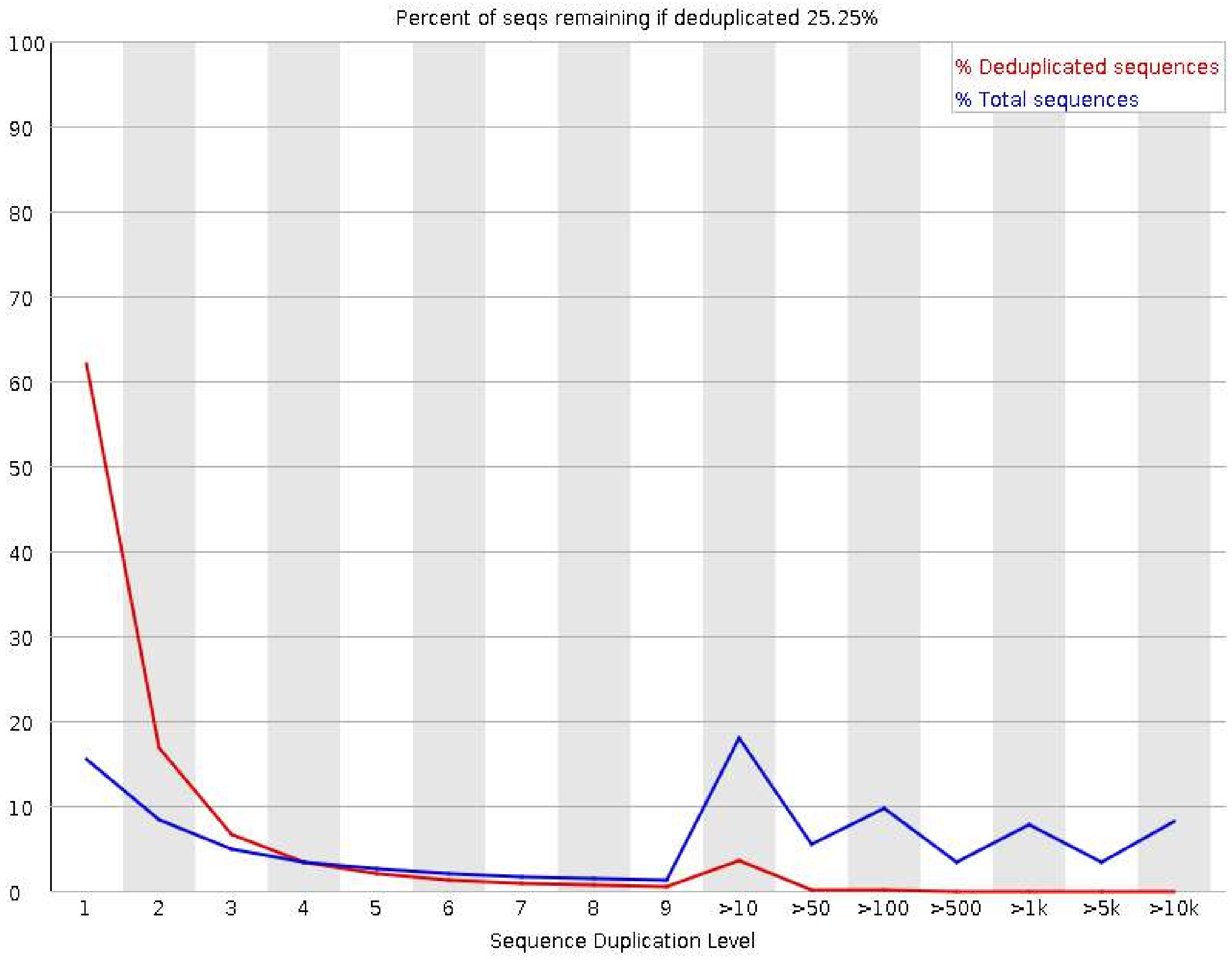

Overrepresented sequences

Sequence	Count	Percentage	Possible Source
GTAAGGATTTAAGAAAGGTTCTTATGAGGATGGTAAGGTAGGGTAGGGTA	112857	0.2579653490251263	No Hit
GCTTGGTGTTTTGGTGTATTCGAGCTGCTTTAATAAACTTCTTTATGAGA	103640	0.23689739026346696	No Hit
GTCGTCTTTGTCGTGGCCGGGACTAGGTTATCTAAGTAAGTATCGTTCGG	102813	0.2350070569775939	No Hit
TAAGGATTTAAGAAAGGTTCTTATGAGGATGGTAAGGTAGGGTAGGGTAA	99872	0.2282846020879291	No Hit
GGGTAGGGTAACTCCAGGCACTTACTAGCTGTTGTCCTGAATTAATTTGT	84024	0.19205969046215313	No Hit
GCTGGTGCTAAGTGCATGTTGGGCGAAGGTAAGTGGTATGGAGATGATAG	77797	0.17782618940878947	No Hit
GTCGGTTGTAAAGTGGTATAAGACTTGTCGTAATAGGCAAATAATTAAGA	60960	0.13934064946411567	No Hit
GTCAGATCTGTATCTATTATTGTTGAAGATATCTAGGGAGTGAGTGCATA	59568	0.13615885510627365	No Hit
GGGGGATTAGAAAAAGTATAACTCTTTACTTTGTGTCATCCTAGGCTGTC	52673	0.12039845848463523	No Hit
CTTGGTGTTTTGGTGTATTCGAGCTGCTTTAATAAACTTCTTTATGAGAC	50171	0.11467945741903127	No Hit
CTCTGCCTTTTTTCCTCTATACTCCTATCTCTTTAACAGCACTCATTGAT	49899	0.11405772748703917	No Hit
GGCTTGGTGTTTTGGTGTATTCGAGCTGCTTTAATAAACTTCTTTATGAG	49690	0.1135800011790011	No Hit

Sequence	Count	Percentage	Possible Source
CGGGCTTGGTGTTTTGGTGTATTCGAGCTGCTTTAATAAACTTCTTTATG	49019	0.11204624829529997	No Hit
CTCCTTCCTAATATCTTTCGTTTATGTAGTCGATTTAAGAGTTAGATACC	47491	0.10855358897146189	No Hit
CCTCTGCCTTTTTTCCTCTATACTCCTATCTCTTTAACAGCACTCATTGA	45934	0.10499464226516877	No Hit
CCCGCCCGTACCATATTAGCCGTTTTATTGACAATTCTATAAAGAGCGAT	45346	0.1036506084415976	No Hit

Adapter Content

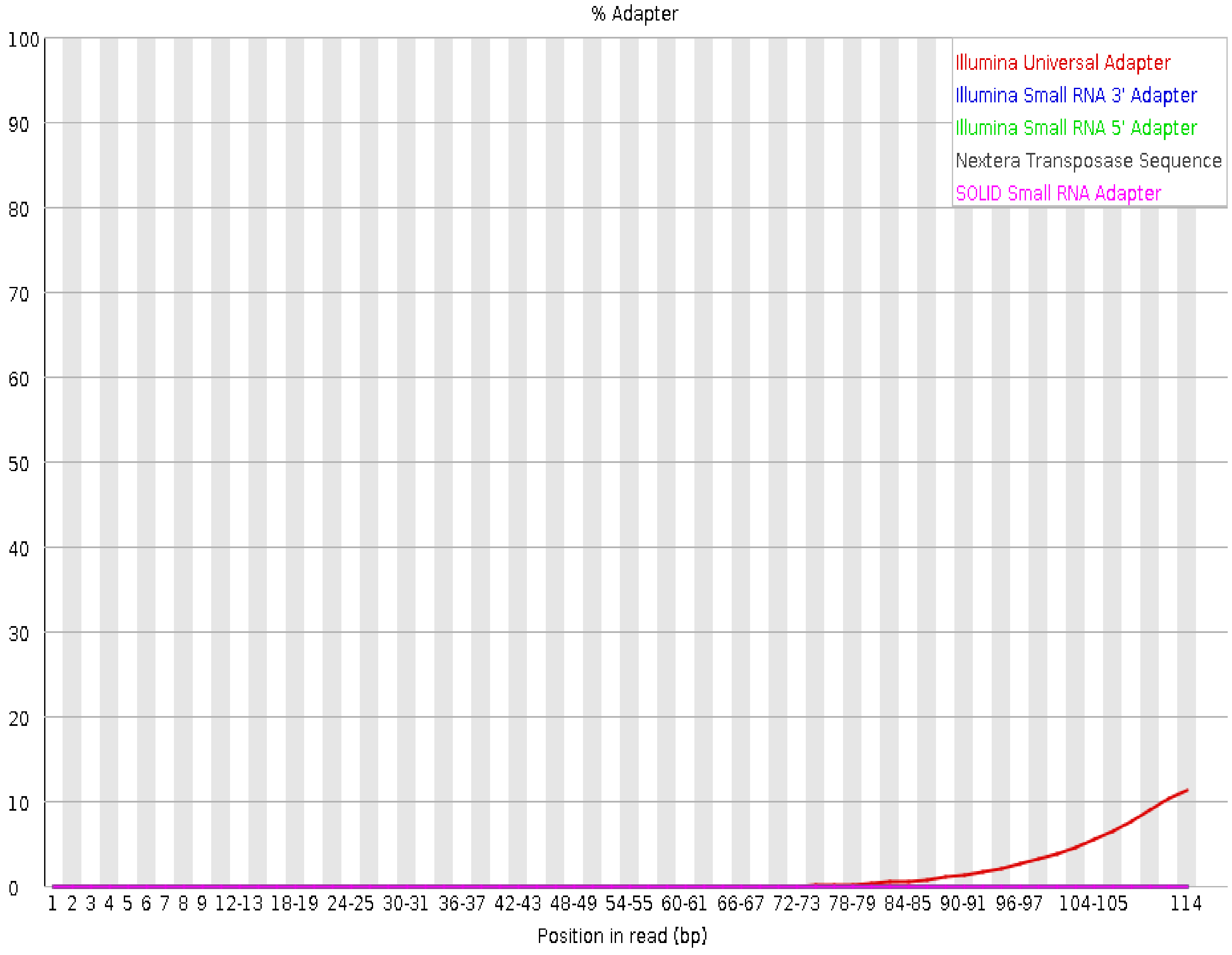

Produced by FastQC (version 0.11.8)

FastQC Report

Wed 9 Oct 2019
Eulamprus.Male.Testis_R2.fastq.gz

Summary

- Basic Statistics
- Per base sequence quality
- Per tile sequence quality
- Per sequence quality scores
- Per base sequence content
- Per sequence GC content
- Per base N content
- Sequence Length Distribution
- Sequence Duplication Levels
- Overrepresented sequences
- Adapter Content

Basic Statistics

Measure	Value
Filename	Eulamprus.Male.Testis_R2.fastq.gz
File type	Conventional base calls
Encoding	Sanger / Illumina 1.9
Total Sequences	43748899
Sequences flagged as poor quality	0
Sequence length	125
%GC	45

Per base sequence quality

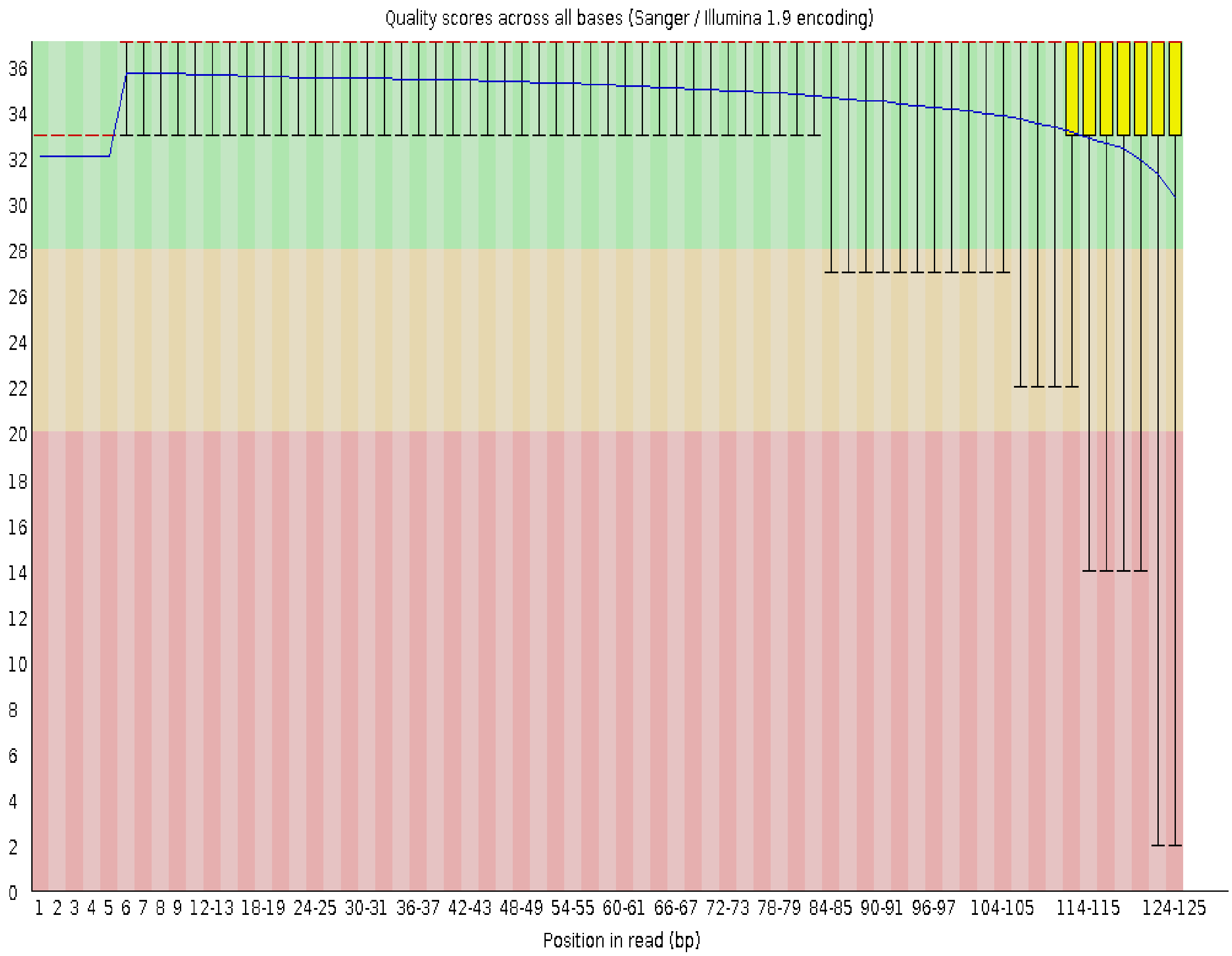

Per tile sequence quality

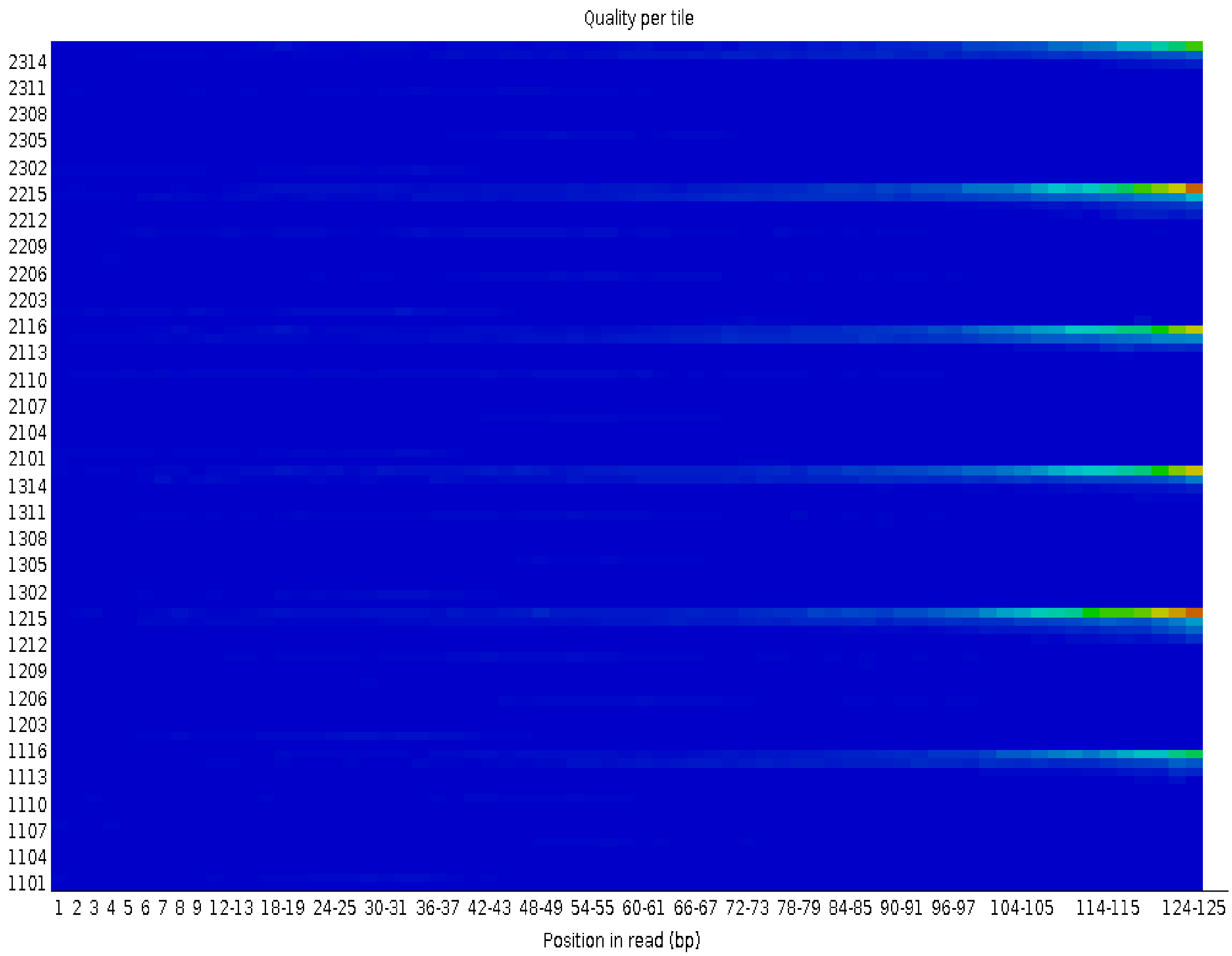

Per sequence quality scores

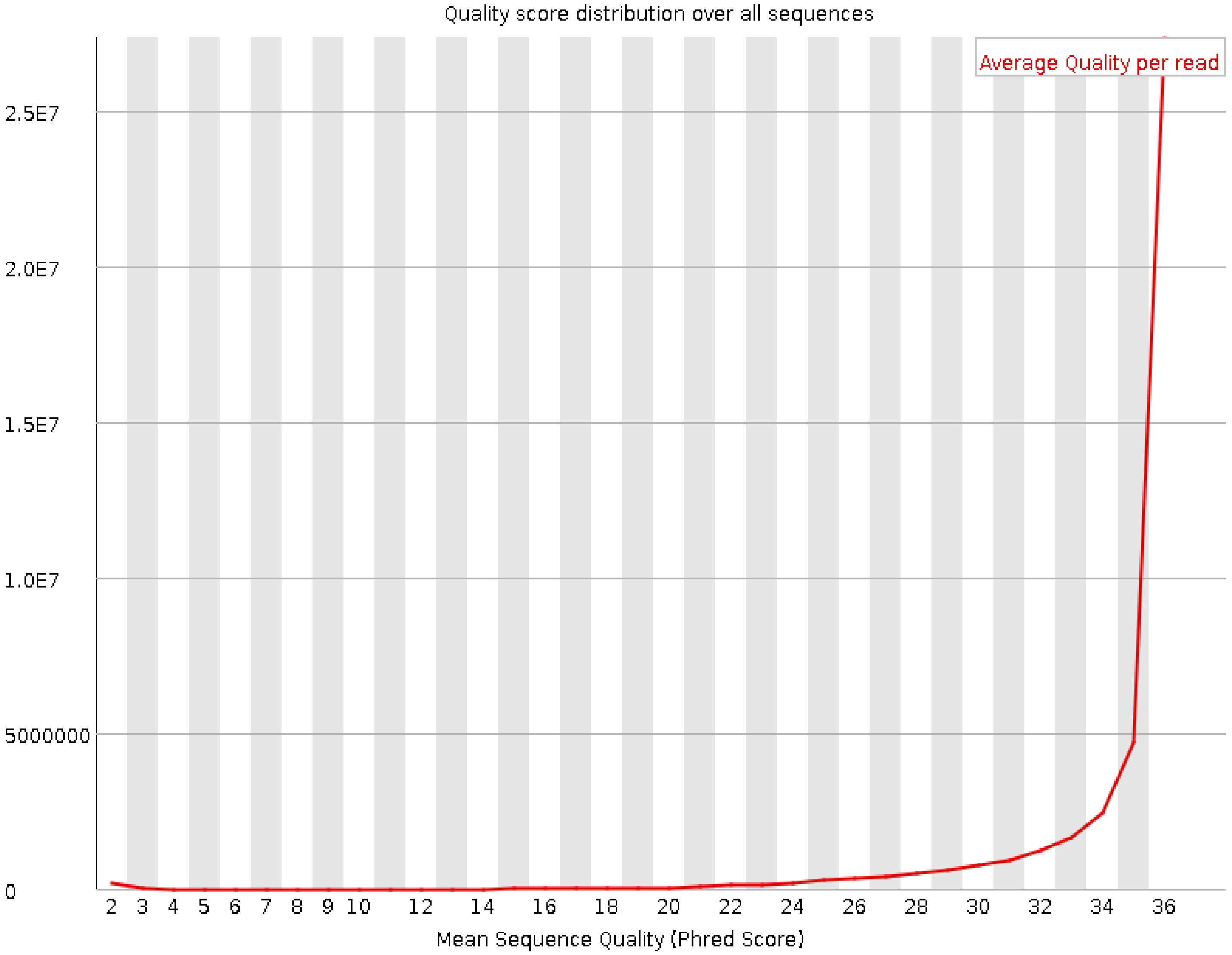

Per base sequence content

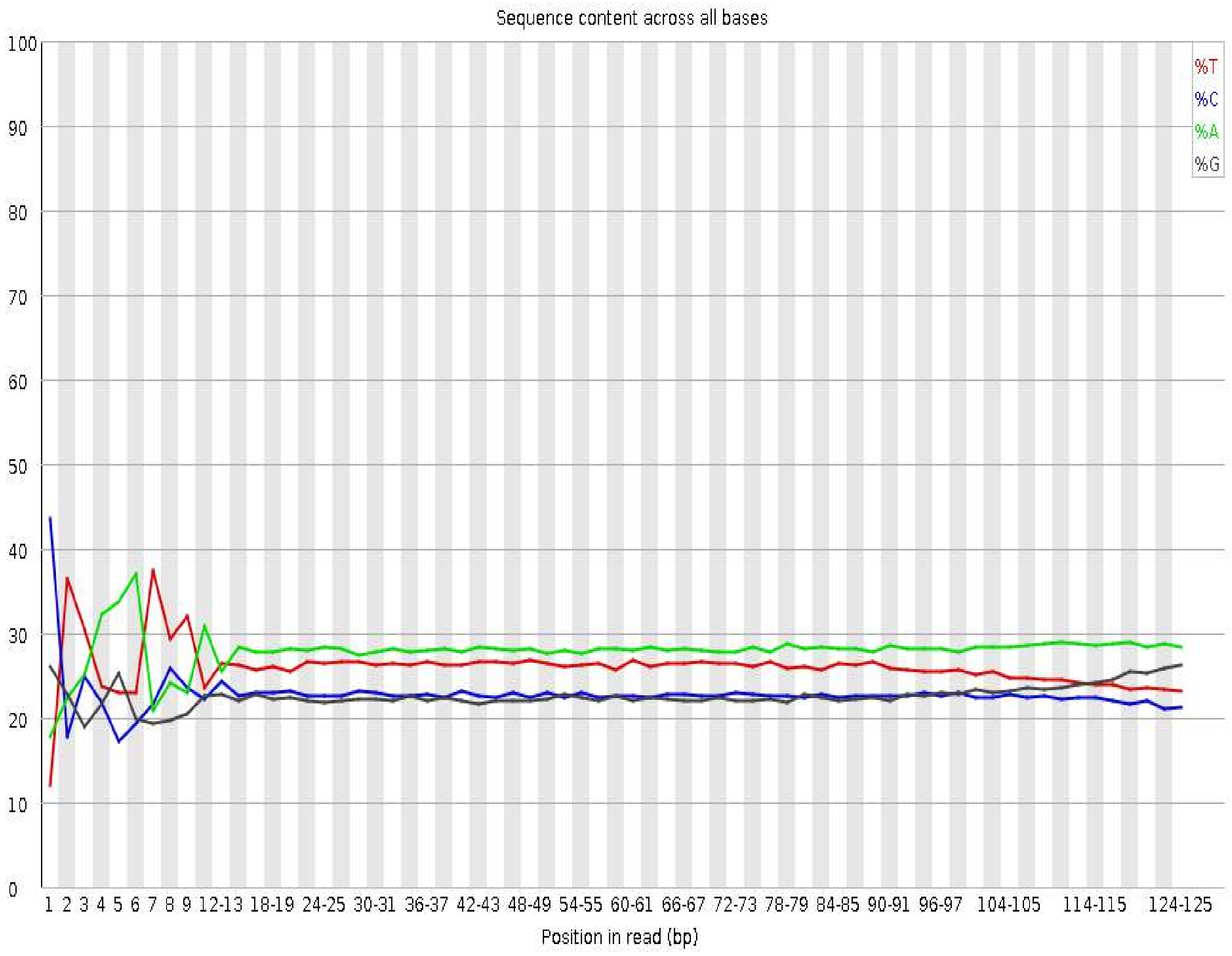

Per sequence GC content

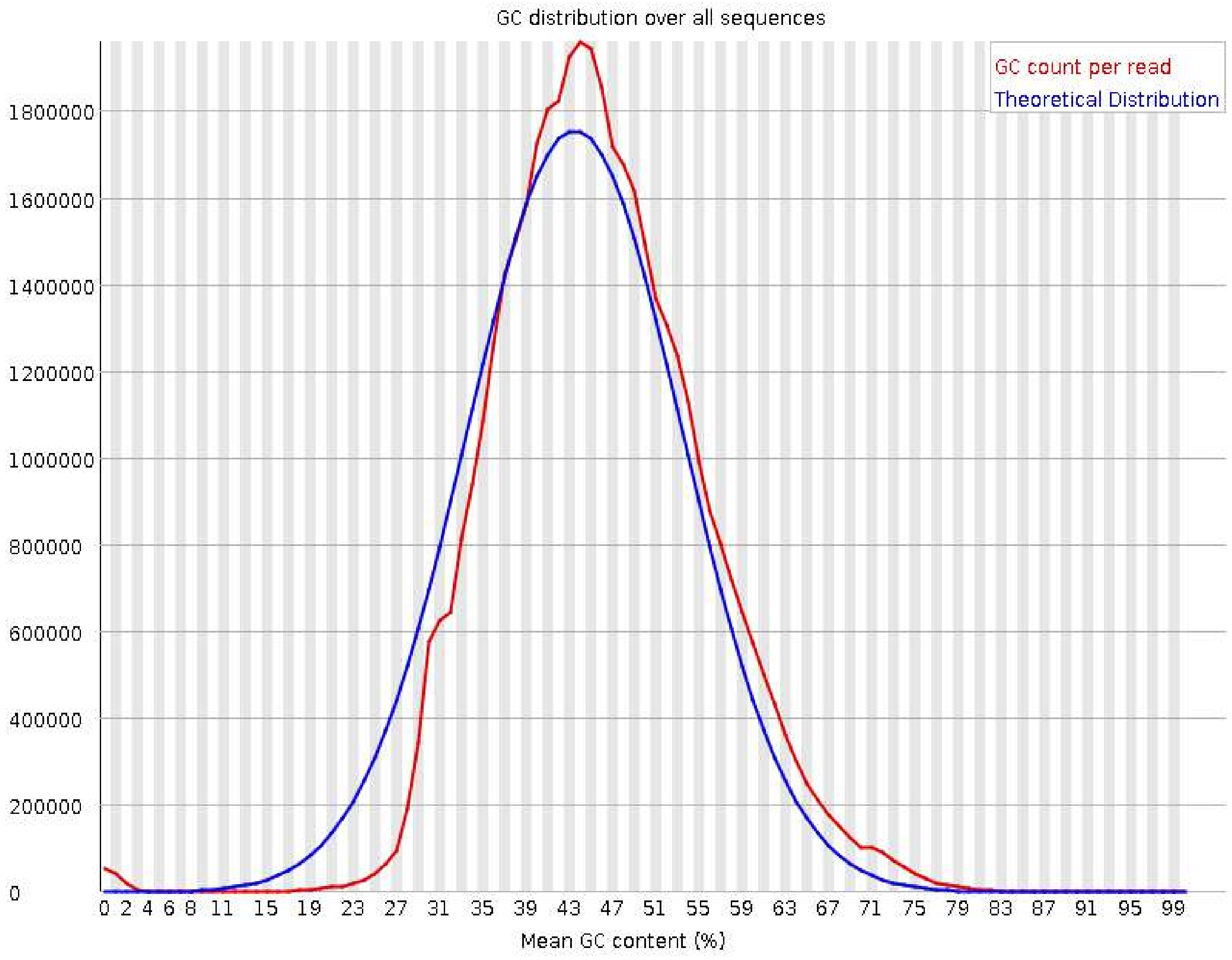

Per base N content

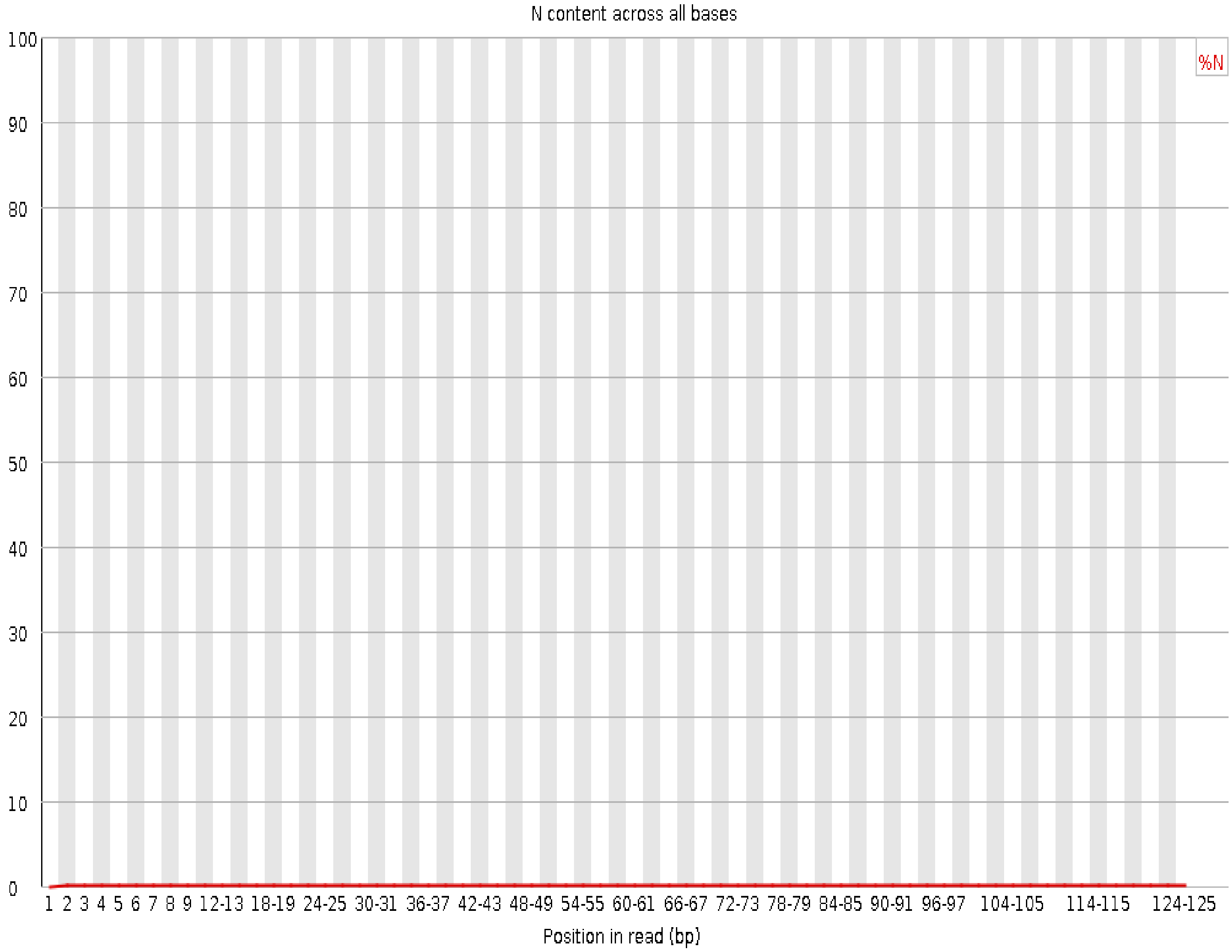

Sequence Length Distribution

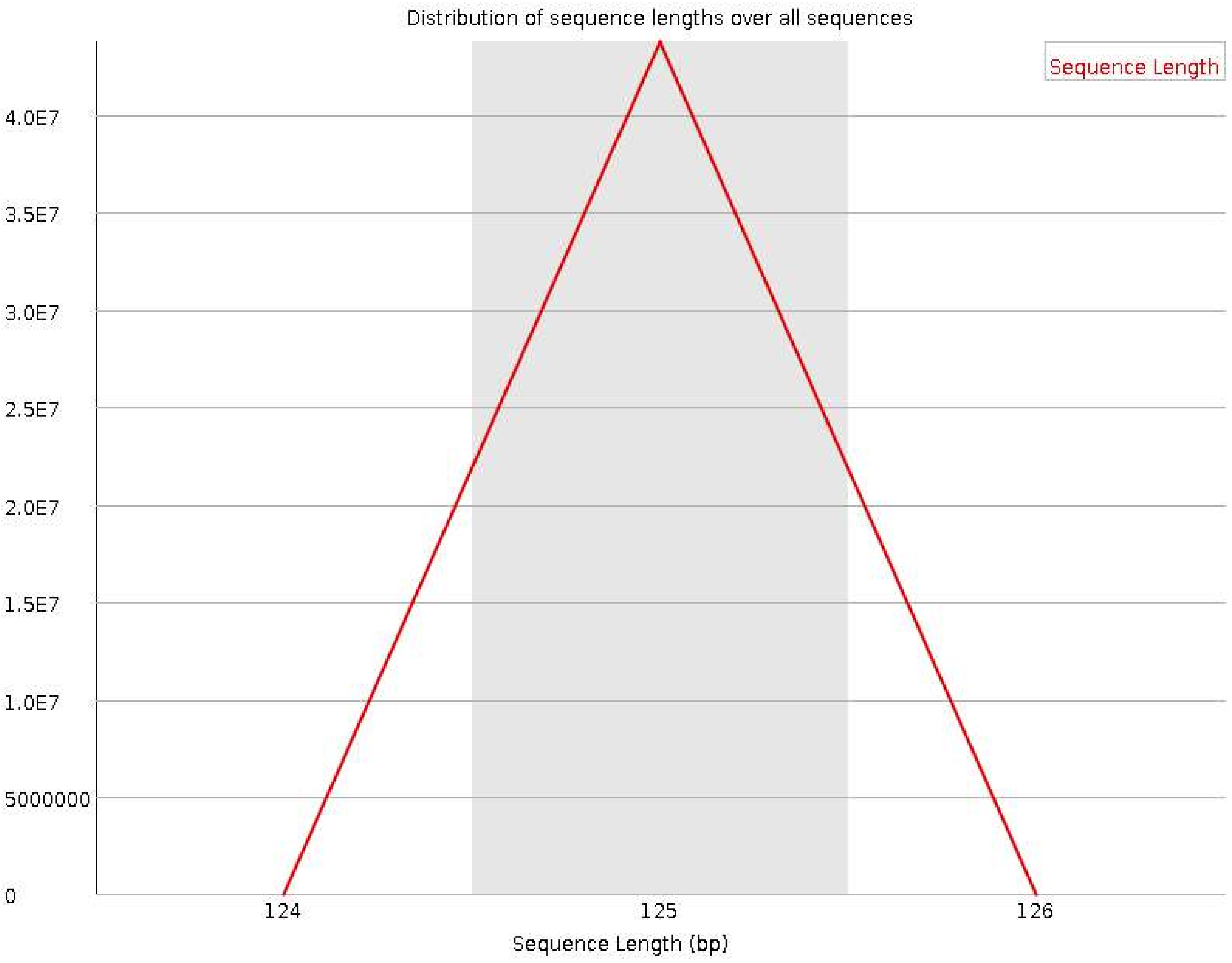

Sequence Duplication Levels

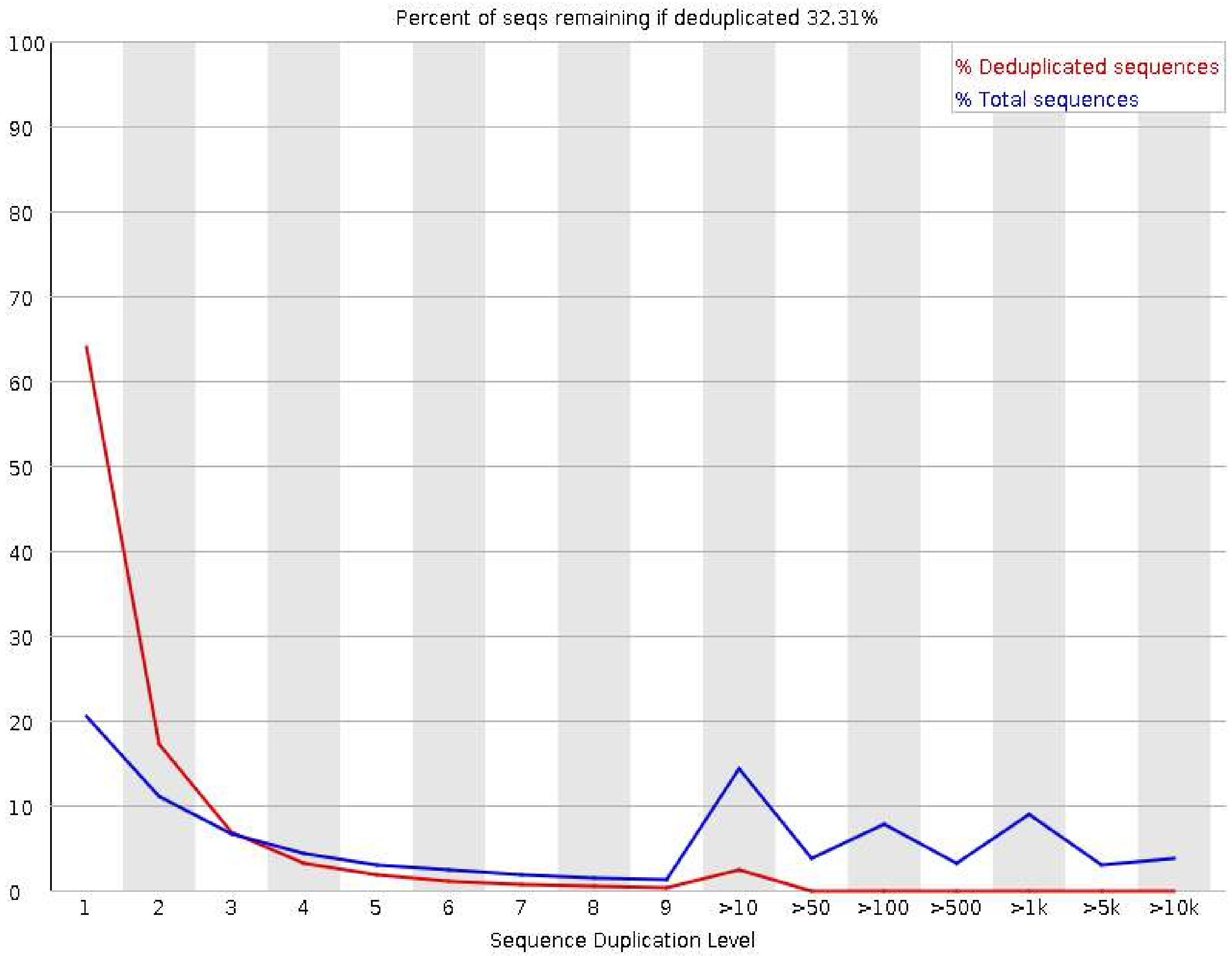

Overrepresented sequences

Sequence	Count	Percentage	Possible Source
CTCAGTTCTCTTGAAACCTACATAAATTCGCAATTATGACAATATATTAC	308527	0.7052223188519555	No Hit
CTTCAACAATAATAGATACAGATCTGACCAACTAGACGGCTAATGGCTTG	96746	0.22113927941363734	No Hit
GTTCTCTTGAAACCTACATAAATTCGCAATTATGACAATATATTACTAGC	54398	0.12434141485480583	No Hit
CTTTTATATTGTTCAACTTCTCTCTTAATAGCTATGCACTCACTCCCTAG	50360	0.11511146829089344	No Hit

Adapter Content

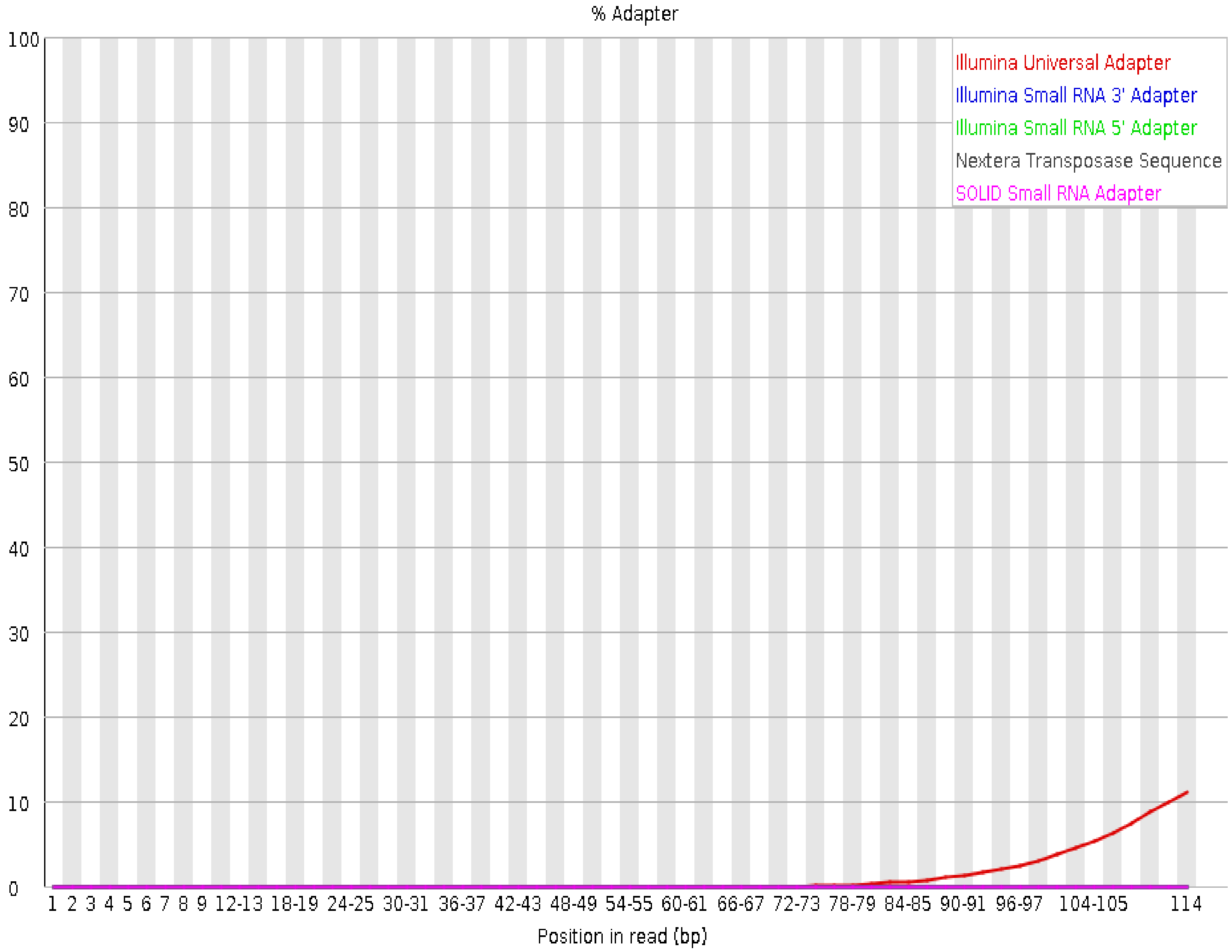

Produced by FastQC (version 0.11.8)
