# Supplementary material for: Viviparous Reptile Regarded to Have Temperature-Dependent Sex Determination Has Old XY Chromosomes
Source: Genome Biol Evol. 2020 May 20;12(6):924–30. doi: 10.1093/gbe/evaa104 (PMC7313667; doi:10.1093/gbe/evaa104)
Supplement: evaa104_Supplementary_Data [file evaa104_supplementary_data.zip › Supp_Figures1-5_compressed.pdf]

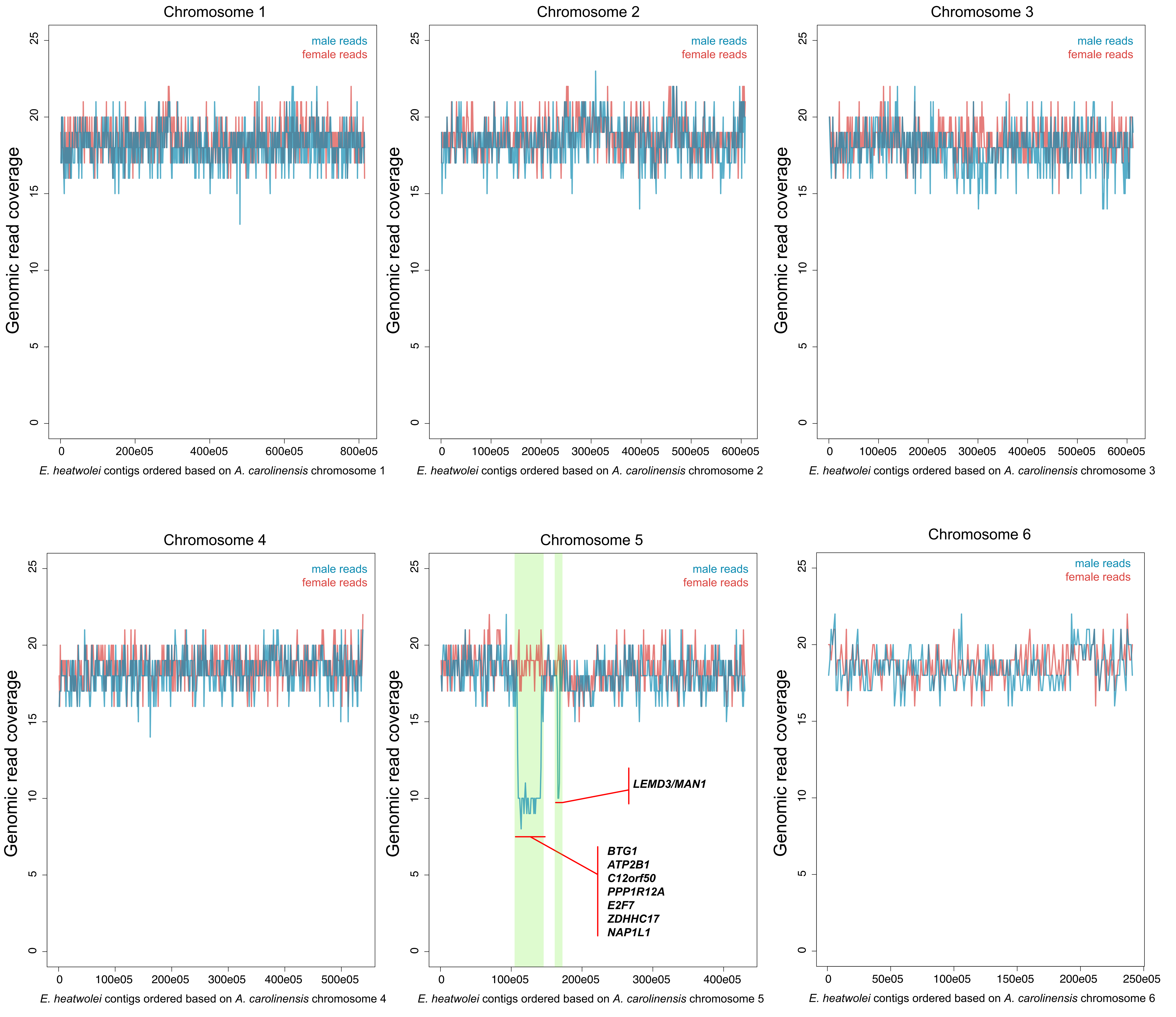

Supp. Figure 1. Male (blue lines) and female (red lines) coverage of genomic reads for six reconstructed chromosomes in eulamprus. Chromosome 5 shows a region with half the coverage in males (only one copy), that is exactly where the X gametologs are located.

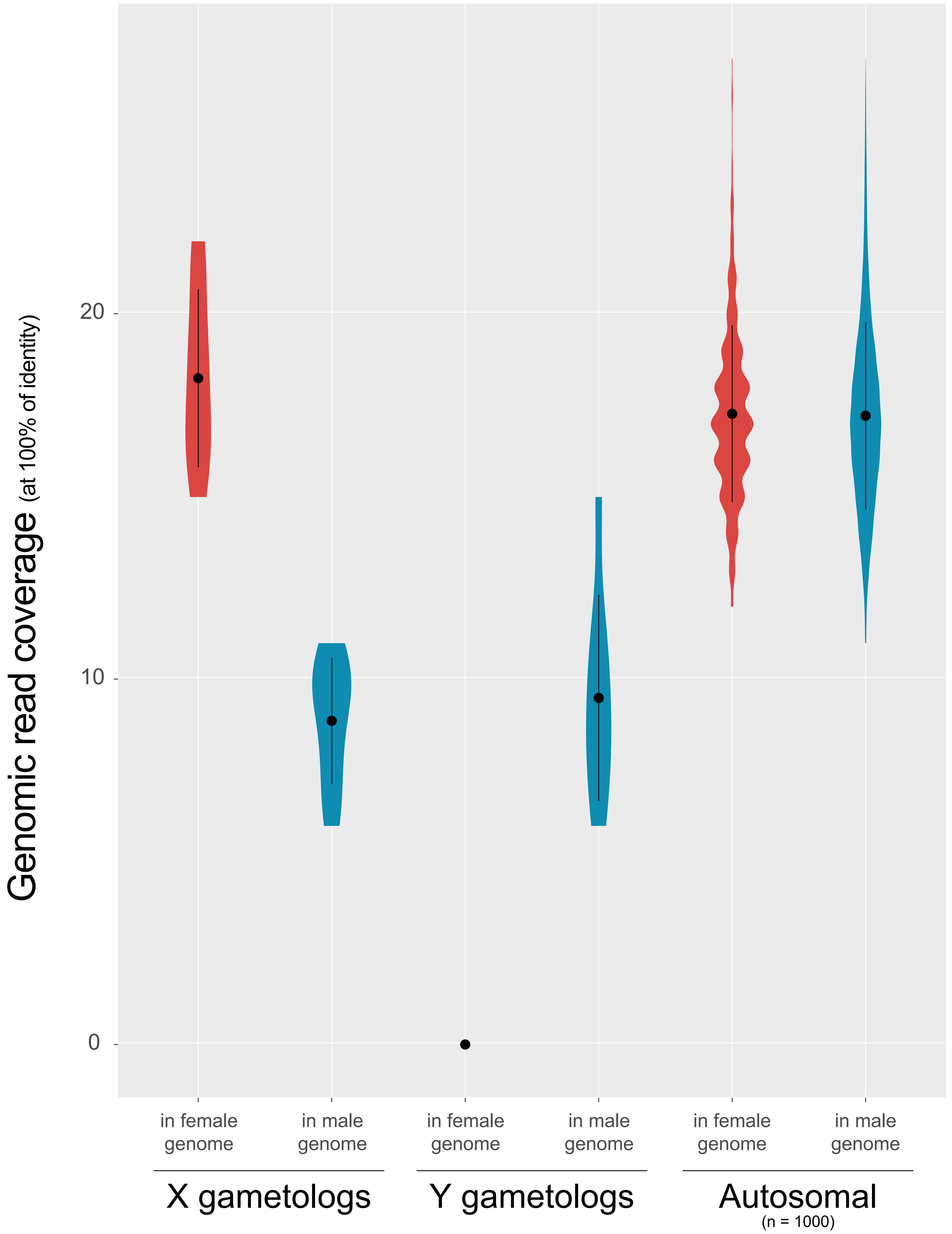

Supp. Figure 2. Violin plots showing male (blue) and female (red) median coverage for the 14 XY gametologs and 100 autosomal genes in eulamprus. X gametologs in females show the same coverage as autosomal genes. X and Y gametologs in males show half of this coverage. Y gametologs in females show zero coverage.

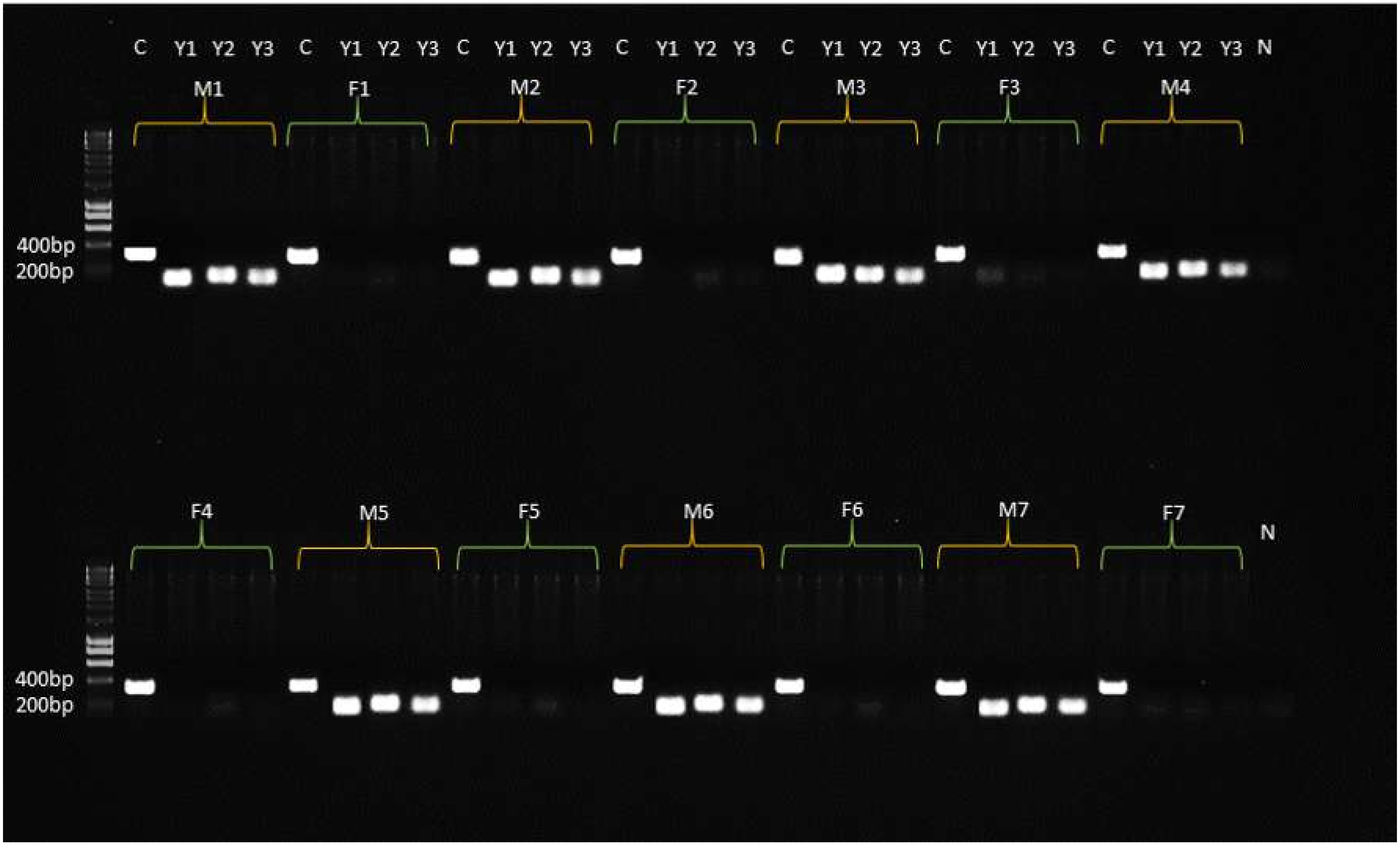

Supp. Figure 3. Validation of three Y-specific markers in *E. heatwolei* by screenings by standard PCR using genomic DNA from 7 males (M1-7) and 7 females (F1-7). Male specificity was defined by the present of an amplicon in males but not in females. C: positive control, autosomal gene *COL1A1,* it was amplified in both males and females. Y1: *BTG1*, amplification only in males; Y2: *PPP1R12A*, amplification only in males; Y3: *TNPO3*, amplification only in males. Lane N was a negative control without genomic DNA.

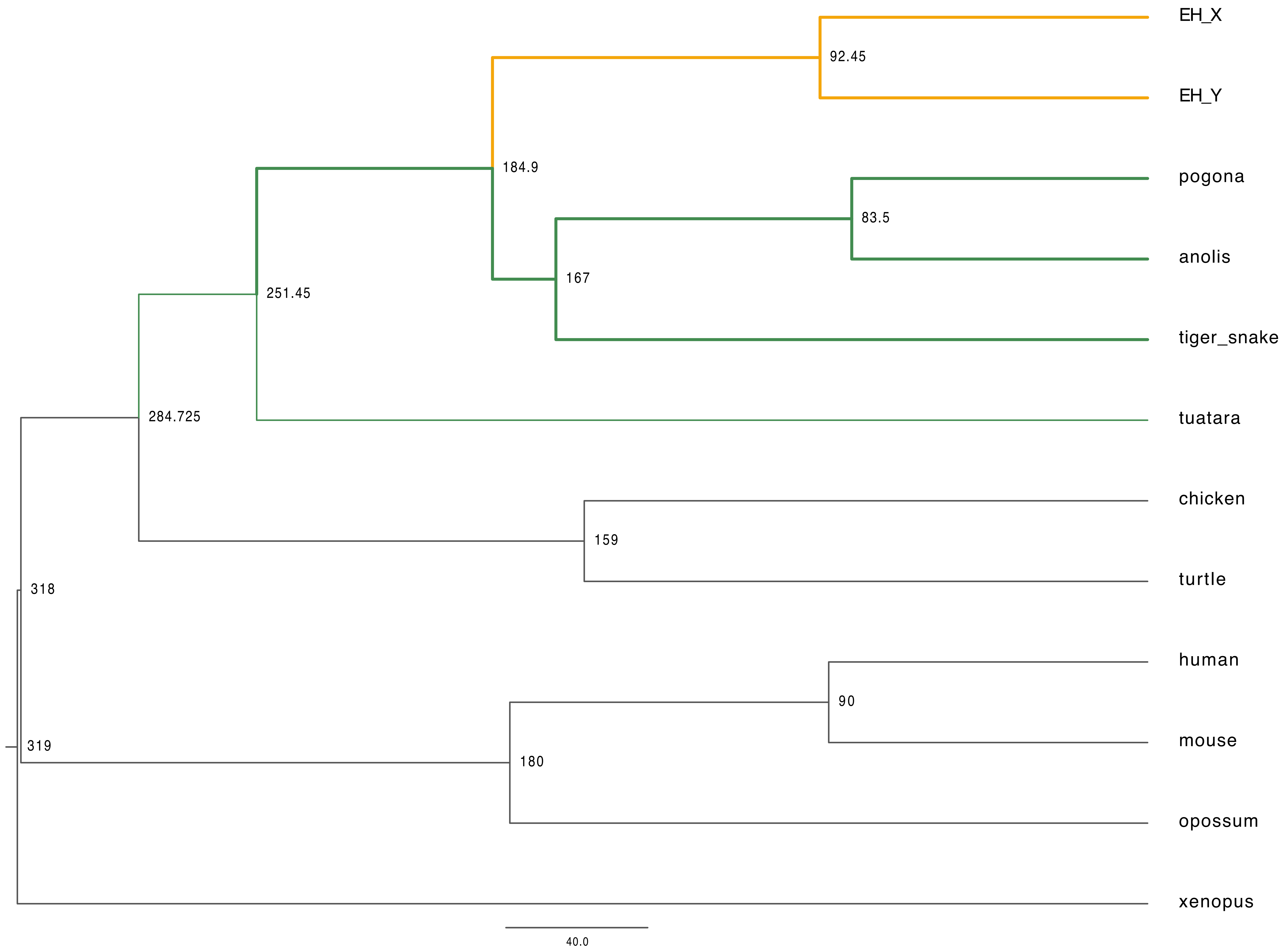

Supp. Figure 4. Average age estimate of two independent runs to calculate the age of the sex chromosomes using BEAST v1.10.4 (http:/beast.bio.ed.ac.uk/). For this analysis, we used the relaxed clock and calibrated the tree based on the reptile/mammalian divergence time, GTR+I+G for the substitution model (estimated for the dataset), and a normal distribution with SD of 1 as priors on the calibration nodes to accommodate calibration uncertainty. The analyses were run two independent times for 100,000,000 generations, sampling every 1,000 generations.

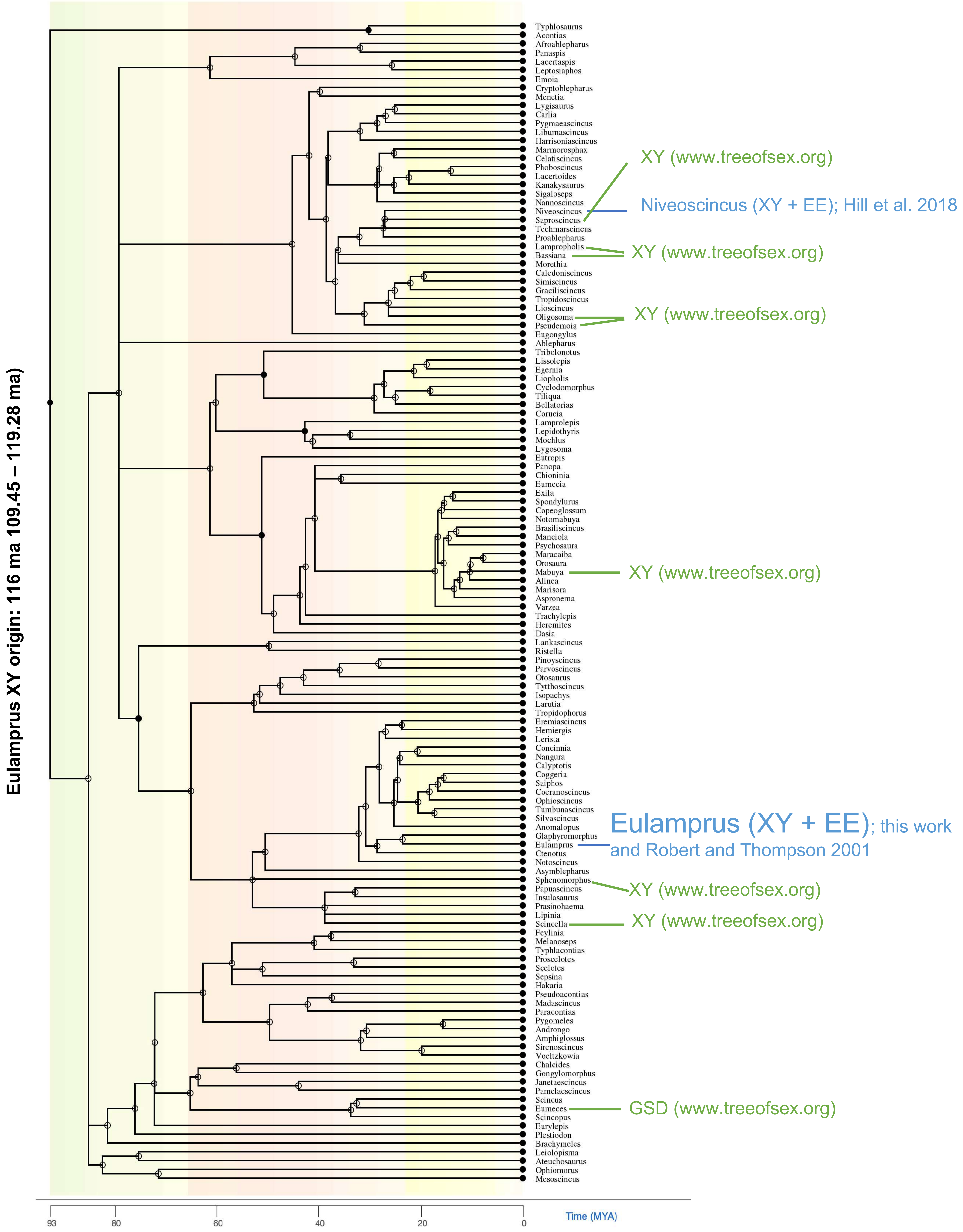

Supp. Figure 5. Phylogenetic tree of 132 genera of the *Scincidae* familiy. Known genus with XY or XY + EE (Environmental Effects) are indicated. The XY systems could share a common origin.
